# Safety and Efficacy of Different Stent Strategies in Percutaneous Coronary Intervention: A Network Meta-Analysis

**DOI:** 10.1016/j.jacadv.2025.101600

**Published:** 2025-02-18

**Authors:** Waqas Ullah, Harigopal Sandhyavenu, Syeda Ramsha Zaidi, Mileydis Alonso, Muhammad Atif Khan, Irfan Ullah, Farah Yasmin, Aravind Reddy Polam, Salman Zahid, Arnav Kumar, Ewout W. Steyerberg, Mohammad Murtaza, Chadi Alraiesm, Jeffrey J. Rade

**Affiliations:** a Department of Cardiology, University of Massachusetts Chan Medical School, Worcester, Massachusetts, USA; b Department of Cardiology, UT Health San Antonio, San Antonio, Texas, USA; c Department of Pulmonology and Critical Care, Marshall University School of Medicine, Huntington, West Virginia, USA; d Department of Cardiology, Cleveland Clinic Florida, Weston, Florida, USA; e Department of Internal Medicine, University of Kansas Medical Center, Kansas City, Kansas, USA; f Department of Internal Medicine, Khyber Teaching Hospital, Peshawar, Pakistan; g Department of Internal Medicine, Yale School of Medicine, New Haven, Connecticut, USA; h Department of Internal Medicine, Weiss Memorial Hospital, Chicago, Illinois, USA; i Department of Cardiology, Oregon Health and Science University, Portland, Oregon, USA; j Department of Cardiology, HCA Houston Heart Medical Center, Houston, Texas, USA; k Department of Biomedical Data Sciences, Leiden University Medical Center, Leiden, the Netherlands; l Department of Cardiology, Thomas Jefferson University Hospitals, Philadelphia, Pennsylvania, USA; m Department of Cardiology, Detroit Medical Center, Detroit, Michigan, USA

**Keywords:** drug-eluting stents, everolimus, polymer-absorbable, polymer-free, polymer-permanent, sirolimus, titanium-nitride-oxide, zotarolimus

## Abstract

**Background:**

The utility of polymer-permanent, polymer-absorbable, and polymer-free drug-eluting stents (DES) in the context of different eluting drugs in patients undergoing percutaneous coronary intervention (PCI) remains controversial.

**Objectives:**

The purpose of this study was to compare the efficacy of different DES strategies in post-PCI patients.

**Methods:**

Digital databases were searched to select all randomized control trials. Different combinations of DES were directly compared with permanent-polymer sirolimus-eluting stents. The primary outcome was major adverse cardiovascular events; a composite of cardiovascular mortality, myocardial infarction (MI), and target vessel revascularization. A network meta-analysis was performed to determine the net relative risk (RR) and its 95% CI.

**Results:**

A total of 314 randomized control trials comprising 345,749 patients with coronary artery disease undergoing PCI were included. Compared with polymer-permanent sirolimus-eluting stent, polymer-free titanium-nitride-oxide (RR: 0.68; 95% CI: 0.53-0.87), polymer-permanent everolimus (RR: 0.89; 95% CI: 0.82-0.96), and zotarolimus stents (RR: 0.89; 95% CI: 0.79-0.99) had a lower risk of major adverse cardiovascular events at 5 years. Polymer-free titanium-nitride-oxide stents also had a significantly lower incidence of stent thrombosis (RR: 0.28; 95% CI: 0.13-0.59) and MI (RR: 0.41; 95% CI: 0.27-0.62) at 1 year. Bare metal stents had a significantly higher 1-year risk of MI (RR: 1.52; 95% CI: 1.25-1.86), and need for target vessel revascularization (RR: 2.26; 95% CI: 1.93-2.64).

**Conclusions:**

In comparison with polymer-permanent sirolimus, the newer stents including polymer-free titanium-nitride-oxide, polymer-permanent everolimus, and zotarolimus stents significantly reduce the risk of long-term ischemic events.

Bare-metal stents (BMS) demonstrated superiority over balloon angioplasty in preventing restenosis by addressing vascular recoil and remodeling.^1^ However, their effectiveness was limited by neointimal hyperplasia. The development of drug-eluting stents (DES), which incorporate antiproliferative agents like sirolimus and paclitaxel within a permanent polymer coating, marked a significant advancement, reducing tissue proliferation and in-stent restenosis.^2^, ^3^, ^4^, ^5^ Despite these benefits, polymer-coated DES posed risks such as delayed endothelialization by the antirestenotic drugs and hypersensitivity reaction to the polymer coating.^6^, ^7^, ^8^, ^9^, ^10^, ^11^, ^12^ To address these challenges, DES generations introduced biodegradable polymer and polymer-free coatings alongside highly potent antiproliferative drugs. Additionally, newer generation titanium-nitride-oxide (TiNO)–coated stents emerged with improved biocompatibility. These stents, often referred to as *bioactive stents*, evolved as a cobalt-chromium, thin-strutted coated with an ultrathin metallic layer that exposes nitric oxide molecules toward the vascular lumen.^13^, ^14^, ^15^, ^16^, ^17^, ^18^

While numerous randomized controlled trials and meta-analyses have examined DES and polymer types, a comprehensive evaluation comparing different DES generations across polymer types, factoring in antiplatelet therapy duration, clinical presentation, and follow-up, remains lacking.

## Methods

The current network meta-analysis (NMA) was conducted in adherence to the Preferred Reporting Items for Systematic Reviews and Meta-Analyses (PRISMA) checklist (Supplemental Table 1).

### Search strategy

PubMed, Cochrane, and Embase databases were searched up to January 2024 using Boolean operators and MeSH terms. Screening was conducted at the title and abstract level, with full-text appraisal and data extraction for relevant studies. References of included randomized control trials (RCTs) were assessed via backward snowballing. Details of the search strategy are provided in the supplementary appendix. This study was exempted from Institutional Review Board/ethics committee approval as data were obtained from previously published studies.

### Selection criteria and study subjects

Included RCTs compared DES and BMS, focused on post-percutaneous coronary intervention (PCI) all-comers with stable angina or acute coronary syndrome (ACS), reported at least one efficacy outcome and had follow-up durations of 6 months to 5 years. Studies with duplicate populations, incomplete data on stent type or follow-up, reviews, conference papers, and case reports were excluded.

### Comparison strategies

The included RCTs were categorized into 4 major strategies: BMS, polymer-free, polymer-absorbable, and polymer-permanent DES. The latter 3 were further divided into 18 subgroups based on the eluting drug. The common control arm was polymer permanent sirolimus-eluting stents.

### Study outcomes

The primary outcome was major adverse cardiovascular events (MACE); a composite of cardiovascular mortality, myocardial infarction (MI), and target vessel revascularization (TVR). Secondary endpoints included individual components of MACE, all-cause mortality, stent thrombosis (ST), and target lesion revascularization (TLR).

### Statistical analysis

NMA was performed using a frequentist random effects model to analyze mixed treatment comparisons. Network geometry illustrated comparison strategies, with open and closed loops indicating indirect and direct comparisons, respectively. The prerequisites of NMA (similarity and transitivity) were satisfied by detailed scrutinization of trial-level methodology. Loop and global consistency of the summary estimates was statistically validated and a *P* value >0.05 suggested no evidence of inconsistency. The design- and study-level estimates were illustrated using intervals and the network forest plots. Minimal parallelism and mean path length visualized net effect size and indirect evidence, respectively. Treatment ranking was determined using surface under the cumulative ranking, where values closer to 1 indicated superior performance. Subgroup analyses were conducted by clinical presentation, follow-up duration, and dual antiplatelet therapy (DAPT) duration. Sensitivity analysis using the “leave-one-out” method evaluated the impact of individual studies.

Trial sequential analysis (TSA) evaluated the power and summary estimate stability. The former was confirmed when the accrued cumulative effect size (Z-curve) reached either the O'Brien–Fleming monitoring boundary or the required information size. The trend of evidence was unlikely to alter, if the *z*-score curve crossed the futility boundary. Required information size was calculated for 80% power (β = 0.20) and a *P* value of 0.05, based on MACE incidences of 13.77% and 14.83% and all-cause mortality incidences of 8.26% and 9.28% for intervention and control groups, respectively.

Higgins's I-squared statistics and L'Abbé plots were used to assess heterogeneity in the pairwise estimates, while Egger's regression equation and funnel plots were used for publication bias. Analysis was performed using Copenhagen Trial Unit, STATA v16 (STATACORP LLC v16), and R v4.01 (R Foundation v4.01).

## Results

### Search results

**Figure 1:**
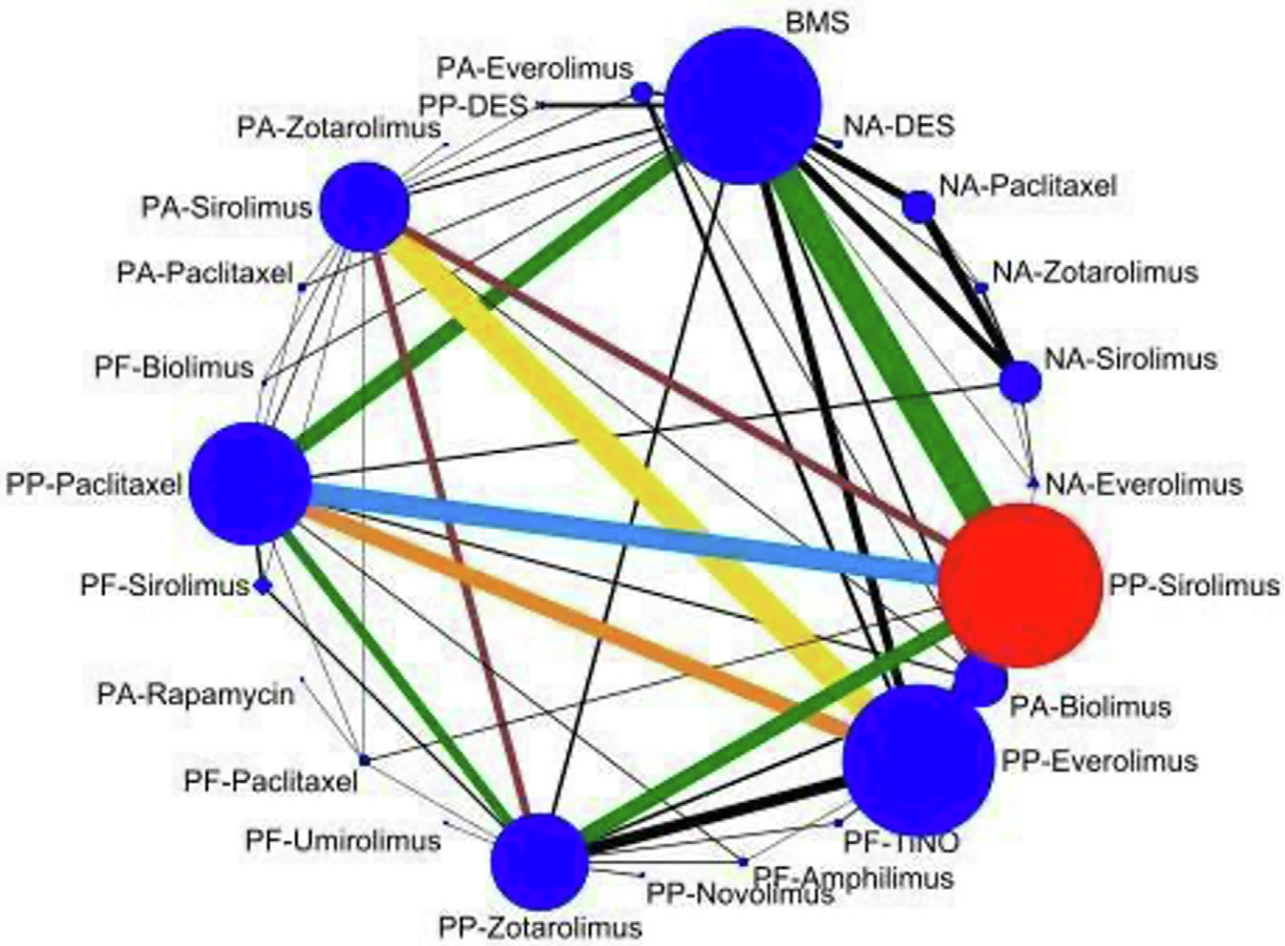
Network of Treatments Indicating Direct and Indirect Treatment Comparisons The size of the node represents the number of studies and thickness of line represent the amount of relevant data. BMS = bare metal stent; DES = drug-eluting stent; NA = not available; PA = polymer-absorbable; PF = polymer-free; PP = polymer-permanent; TiNO = titanium-nitride-oxide.

A comprehensive literature search identified 11,716 items, reduced to 9,460 after duplicates removal. 8,326 records that did not qualify the selection criteria were removed after screening the title and abstracts. 1,134 full-text articles were reviewed, of which 314 (196 primary, 118 secondary) RCTs qualified for quantitative analysis (Supplemental Figures 1 and 2). The relative contribution and relevance of treatment regimens are illustrated in the network geometry (Figure 1).

### Study characteristics

A total of 345,749 patients (176,989 in the experimental group and 168,760 in the control group) across 314 RCTs and 382 stent comparisons were included in the analysis. Among the experimental group, the proportions of major stent types were as follows: polymer-permanent everolimus (26%), polymer-permanent zotarolimus (22%), BMS (7.5%), polymer-permanent paclitaxel (4.3%), and polymer-free TiNO (1.9%). In the control arm, sirolimus-eluting stents accounted for 55% of patients (n = 93,616). The mean age of the study population was 62 ± 10 years, with 72% males. The prevalence of hypertension and other traditional risk factors of CAD were frequent in comparison groups. Most RCTs included all comers, with the highest clinical presentation of the ACS (Table 1). The detailed study-level demographics, baseline characteristics, and clinical presentation are given in Supplemental Table 2, 3, and 4.

**Table 1:** Pooled Baseline Characteristics of the Major Stent Types Directly Compared With the Control Arm (Permanent-Polymer Sirolimus-Eluting Stents)

	PP-ZES (n = 7,946)	PP-SES (n = 7,378)	PP-PES (n = 2,730)	PP-SES (n = 2,756)	PP-EES (n = 5,385)	PP-SES (n = 4,473)	PA-SES (n = 1,917)	PP-SES (n = 1,039)	PA-BES (n = 2,305)	PP-SES (n = 2,245)	BMS (n = 1,802)	PP-SES (n = 1,792)
Demographics												
Age, y	62.5 ± 10.8	62.2 ± 10.8	63.5 ± 10.7	63.8 ± 10.5	64.4 ± 10.2	64.7 ± 10.3	60.1 ± 10.6	60.0 ± 10.9	65.6 ± 10.5	65.8 ± 10.1	62.6 ± 11.2	62.0 ± 10.7
Male	5,872 (73.9)	5,740 (77.8)	2,064 (75.6)	2,075 (75.3)	3,845 (71.4)	3,216 (71.9)	1,365 (71.2)	766 (73.7)	1,699 (73.7)	1,659 (73.9)	1,350 (74.9)	1,342 (74.9)
Comorbidities												
Diabetes mellitus	1,961 (25.5)	1,932 (23.9)	546 (24.7)	557 (24.6)	1,637 (41.3)	1,368 (40.5)	457 (24.4)	248 (27.0)	483 (26.6)	432 (25.7)	435 (27.4)	466 (28.5)
Hypertension	4,820 (58.6)	4,532 (56.8)	1,098 (64.5)	1,154 (65.1)	3,285 (60.8)	2,723 (62.6)	1,245 (60.4)	646 (56.7)	1,461 (69.4)	1,382 (70.6)	1,013 (59.1)	981 (57.5)
Hyperlipidemia	3,944 (56.1)	3,784 (54.5)	1,075 (61.4)	1,039 (58.9)	2,407 (56.6)	1,874 (58.7)	521 (35.3)	259 (27.6)	710 (71.3)	688 (75.0)	962 (67.0)	965 (68.2)
Current smoker	2,334 (38.1)	2,214 (39.7)	905 (32.6)	949 (34.1)	1,402 (31.3)	1,130 (34.0)	793 (51.7)	437 (48.8)	641 (27.8)	619 (25.5)	621 (37.6)	607 (36.3)
Prior PCI	959 (14.4)	894 (13.3)	354 (22.1)	355 (23.9)	1,258 (18.8)	1,254 (21.7)	142 (5.3)	68 (4.8)	580 (28.7)	559 (30.6)	86 (10.6)	78 (10.1)
Prior MI	1,371 (15.4)	1,388 (16.3)	820 (32.8)	770 (29.5)	976 (25.0)	943 (16.8)	310 (11.0)	158 (10.4)	525 (23.5)	511 (23.7)	429 (29.3)	437 (28.1)
Prior CABG	338 (5.3)	345 (5.3)	157 (12.6)	138 (9.6)	249 (4.3)	283 (5.3)	10 (0.8)	6 (0.4)	187 (6.4)	182 (7.2)	21 (3.1)	20 (3.0)
CHF	9 (1.0)	4 (0.5)	25 (3.7)	18 (2.7)	217 (6.9)	208 (6.7)						
PAD	15 (1.7)	21 (2.4)			34 (9.4)	23 (9.2)	23 (1.3)	4 (0.4)				
Prior smoker	204 (4.8)	201 (5.2)	29 (4.2)	30 (4.5)	256 (8.7)	216 (8.9)	171 (9.4)	92 (10.1)			15 (3.7)	12 (2.7)
EF	56.3 ± 10.3	55.2 ± 10.5	35.7 ± 6.8	26.5 ± 7.0	57.8 ± 10.0	58.3 ± 9.9	53.7 ± 7.8	52.4 ± 7.5	55.9 ± 11.3	55.4 ± 12.4	52.6 ± 10.7	53.4 ± 10.0

Values are mean ± SD or n (%). BES = biolimus-eluting stent; BMS = bare metal stent; CABG = coronary artery bypass graft; CHF = congestive heart failure; EES = everolimus-eluting stent; EF = ejection fraction; MI = myocardial infarction; PA = polymer absorbable; PAD = peripheral arterial disease; PCI = percutaneous coronary intervention; PES = paclitaxel-eluting stent; PP = polymer permanent; SES = sirolimus-eluting stent; ZES = zotarolimus-eluting stent.

### Primary endpoint

**Figure 2:**
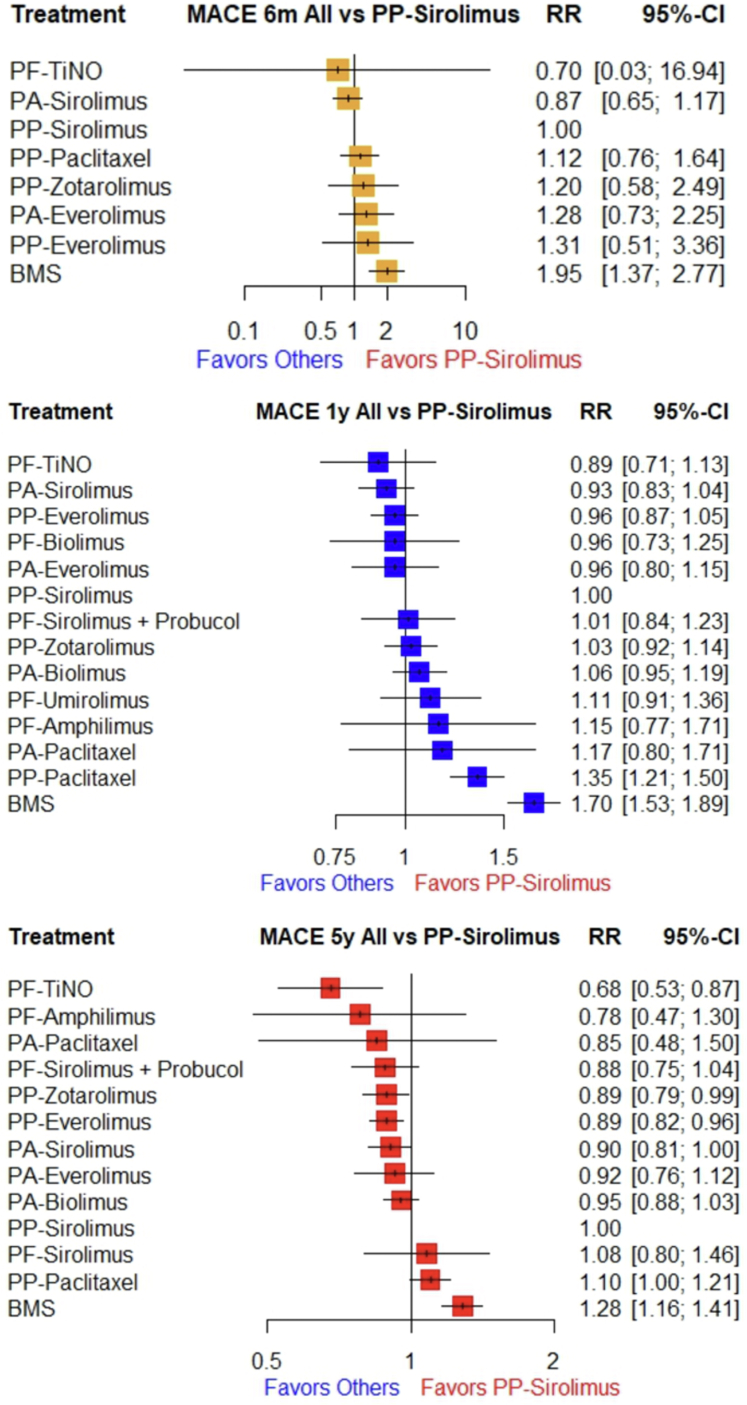
Network Forest Plot for MACE at 6 Months (Yellow), 1 Year (Blue), and 5 Years (Red) for Experimental Vs Control Arms MACE = major adverse cardiovascular outcomes; other abbreviations as in Figure 1.

Compared with polymer-permanent sirolimus stents, the risk of MACE was significantly lower with polymer-free TiNO (RR: 0.68; 95% CI: 0.53-0.87), polymer-permanent everolimus (RR: 0.89; 95% CI: 0.82-0.96), and polymer-permanent zotarolimus stents (RR: 0.89; 95% CI: 0.79-0.99) at 5 years (Central Illustration). The 6-month, 1-year, and 5-year risk of MACE remained higher in patients receiving polymer-permanent paclitaxel and BMS. There was no significant difference in the incidence of MACE between all other DES types at all timepoints (Figure 2, Table 2). The splitwise interval analysis (direct and indirect comparisons) showed similar results (Supplemental Figures 3 and 4).

**Central Illustration:**
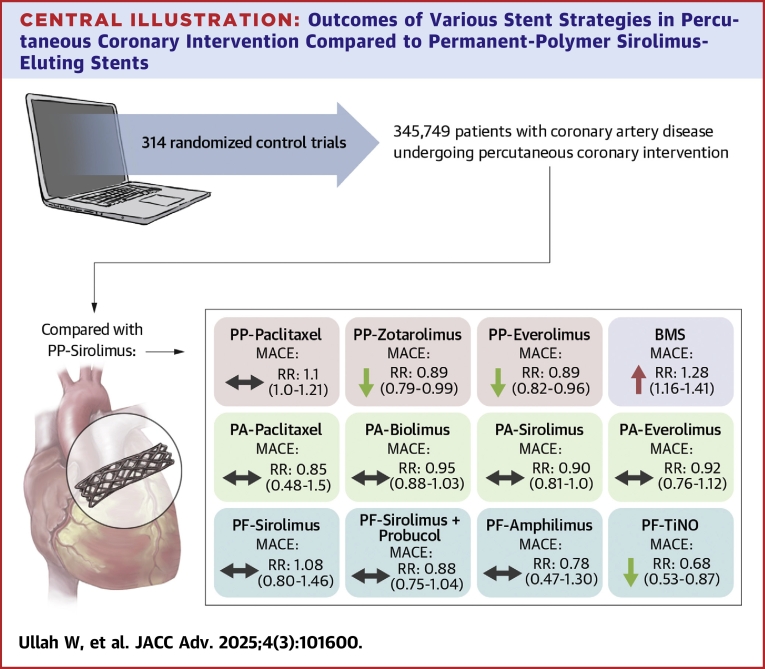
Outcomes of Various Stent Strategies in Percutaneous Coronary Intervention Compared to Permanent-Polymer Sirolimus-Eluting Stents NS = nonsignificant; RR = relative risk ratio; other abbreviations as in Figures 1 and 2.

**Table 2:** Net-League Estimates of MACE Over 1 and 5 Year Across Different Treatment Strategies Showing Risk Ratio With Its 95% CI

1-Year MACE											
BMS	1.60 (1.41-1.81)	1.77 (1.49-2.12)	1.46 (1.01-2.10)	1.84 (1.63-2.07)	1.48 (0.99-2.21)	1.68 (1.39-2.02)	1.90 (1.50-2.41)	1.78 (1.60-1.97)	1.26 (1.13-1.41)	1.70 (1.53-1.89)	1.66 (1.49-1.85)
1.34 (1.21-1.50)	PA-BES	1.11 (0.94-1.31)	0.91 (0.62-1.34)	1.15 (1.02-1.29)	0.93 (0.62-1.38)	1.05 (0.87-1.27)	1.19 (0.94-1.51)	1.11 (1.01-1.23)	0.79 (0.70-0.89)	1.06 (0.95-1.19)	1.03 (0.92-1.16)
1.39 (1.14-1.69)	1.03 (0.85-1.25)	PA-EES	0.82 (0.55-1.23)	1.04 (0.87-1.23)	0.84 (0.55-1.27)	0.95 (0.75-1.19)	1.07 (0.82-1.41)	1.00 (0.85-1.18)	0.71 (0.59-0.85)	0.96 (0.80-1.14)	0.93 (0.78-1.11)
1.51 (0.86-2.65)	1.12 (0.63-1.99)	1.09 (0.60-1.97)	PA-PES	1.26 (0.86-1.85)	1.02 (0.59-1.75)	1.15 (0.76-1.74)	1.31 (0.84-2.02)	1.22 (0.83-1.78)	0.87 (0.59-1.27)	1.17 (0.80-1.71)	1.14 (0.78-1.66)
1.42 (1.25-1.61)	1.05 (0.94-1.18)	1.02 (0.84-1.24)	0.94 (0.53-1.67)	PA-SES	0.81 (0.54-1.20)	0.91 (0.76-1.10)	1.04 (0.82-1.31)	0.97 (0.89-1.05)	0.69 (0.61-0.78)	0.93 (0.83-1.04)	0.90 (0.81-1.00)
1.64 (0.99-2.73)	1.22 (0.73-2.04)	1.18 (0.69-2.02)	1.09 (0.51-2.32)	1.16 (0.69-1.94)	PF-AES	1.13 (0.75-1.72)	1.28 (0.82-2.00)	1.20 (0.81-1.78)	0.85 (0.57-1.27)	1.15 (0.77-1.71)	1.12 (0.76-1.65)
1.45 (1.22-1.71)	1.08 (0.92-1.27)	1.04 (0.83-1.31)	0.96 (0.53-1.73)	1.02 (0.86-1.21)	0.88 (0.52-1.49)	PF-SES + Probucol	1.13 (0.86-1.49)	1.06 (0.89-1.26)	0.75 (0.62-0.91)	1.01 (0.84-1.22)	0.99 (0.85-1.15)
1.89 (1.47-2.43)	1.40 (1.09-1.81)	1.36 (1.01-1.83)	1.25 (0.68-2.32)	1.33 (1.03-1.72)	1.15 (0.66-2.01)	1.30 (1.00-1.70)	PF-TiNO	0.93 (0.75-1.17)	0.66 (0.52-0.84)	0.89 (0.71-1.13)	0.87 (0.69-1.09)
1.44 (1.31-1.59)	1.07 (0.99-1.17)	1.04 (0.87-1.24)	0.96 (0.54-1.69)	1.02 (0.93-1.12)	0.88 (0.53-1.46)	1.00 (0.86-1.15)	0.76 (0.60-0.97)	PP-EES	0.71 (0.64-0.78)	0.96 (0.87-1.05)	0.93 (0.85-1.02)
1.17 (1.06-1.29)	0.87 (0.78-0.97)	0.84 (0.69-1.02)	0.77 (0.44-1.37)	0.82 (0.73-0.93)	0.71 (0.43-1.17)	0.81 (0.69-0.94)	0.62 (0.48-0.79)	0.81 (0.74-0.88)	PP-PES	1.35 (1.21-1.50)	1.31 (1.18-1.46)
1.28 (1.16-1.41)	0.95 (0.88-1.03)	0.92 (0.76-1.12)	0.85 (0.48-1.50)	0.90 (0.81-1.00)	0.78 (0.47-1.30)	0.88 (0.75-1.04)	0.68 (0.53-0.87)	0.89 (0.82-0.96)	1.10 (1.00-1.21)	PP-SES	0.97 (0.88-1.08)
1.44 (1.28-1.64)	1.07 (0.96-1.21)	1.04 (0.86-1.26)	0.96 (0.54-1.70)	1.02 (0.90-1.16)	0.88 (0.53-1.47)	1.00 (0.89-1.12)	0.76 (0.60-0.97)	1.00 (0.92-1.10)	1.24 (1.11-1.38)	1.13 (1.01-1.26)	PP-ZES
5-Year MACE											

AES = amphilimus-eluting stent; MACE = major adverse cardiovascular events; PF = polymer free; TiNO = titanium-nitride-oxide; other abbreviations as in Table 1.

### Secondary endpoints

The network estimates in comparison to polymer-permanent sirolimus stents of secondary endpoints are presented in Supplemental Tables 4 to 15. At 6 months, polymer-permanent everolimus stents (RR: 0.34; 95% CI: 0.13-0.88) had a significantly lower risk of ST. BMS had a higher risk of MI, and risk for TLR and TVR (Supplemental Figure 5). At 1-year, polymer-free TiNO, polymer-permanent everolimus stents, and polymer-absorbable sirolimus stents had a significantly lower, while BMS had a significantly higher risk of ST, and MI compared with polymer-permanent sirolimus stents. Patients undergoing PCI with BMS continued to have a higher risk of TLR (RR: 3.02; 95% CI: 2.34-3.89) and TVR (RR: 2.26; 95% CI: 1.93-2.64) (Supplemental Figure 6). At 5 years, polymer-free TiNO (RR: 0.56; 95% CI: 39-0.82) and polymer-absorbable sirolimus stents (RR: 0.82; 95% CI: 0.68-0.98) had a significantly lower risk of MI, while polymer-permanent everolimus stents had a significantly lower risk of ST (RR: 0.44; 95% CI: 0.30-0.67), risk of TLR (RR: 0.85; 95% CI: 0.76-0.97) and TVR (RR: 0.87; 95% CI: 0.77-0.99). Similarly, polymer-absorbable sirolimus stents had a lower risk of ST (RR: 0.49; 95% CI: 0.30-0.80) and BMS remained to have a higher risk of TLR (RR: 2.09; 95% CI: 1.79-2.44) and TVR (RR: 1.59; 95% CI: 1.38-1.82) (Supplemental Figure 7). Nonetheless, the risk of stroke, CVD, and all-cause mortality remained nonsignificantly different in all stent comparisons at all timepoints (Supplemental Figures 5 to 7). The contribution of direct and indirect evidence to the net estimates for primary and secondary endpoints at all timepoints is given in Supplemental Tables 5 to 17.

### Subgroup analysis

A stratified analysis of 28 RCTs (24,320 patients; 12,251 experimental, 12,069 control) of all comers based on the duration of DAPT (6 months vs 1 year) was also performed compared to polymer-permanent sirolimus stents. At 1 year, there was a similar risk of ST, MI, TVR, TLR, CVD, and all-cause mortality among all stent comparisons with polymer-permanent sirolimus stents, irrespective of DAPT duration (6 and 12 months), except that the risk of TLR and TVR was significantly higher with BMS and polymer-permanent paclitaxel stents (Supplemental Figures 8 and 9).

In patients with ACS, a total of 18 RCTs comprising 15,783 (7,390 experimental, 8,393 control) were included. In concordance with the net results, the risk of MACE in all types of stents compared with polymer-permanent sirolimus stents was similar, except for patients undergoing PCI with polymer-free TiNO, who had a lower 1-year risk of MI (RR: 0.34; 95% CI: 0.14-0.81) and ST (RR: 0.17; 95% CI: 0.07-0.42). Compared to polymer-permanent sirolimus stents, the relative risk of ST was lower with the use of polymer-permanent everolimus stents (RR: 0.45; 95% CI: 0.23-0.88) and the incidence of TLR higher with BMS (RR: 2.57; 95% CI: 1.33-4.99) and TVR (RR: 1.88; 95% CI: 1.44-2.46) (Supplemental Figure 10).

### Ranking of treatment strategies

**Figure 3:**
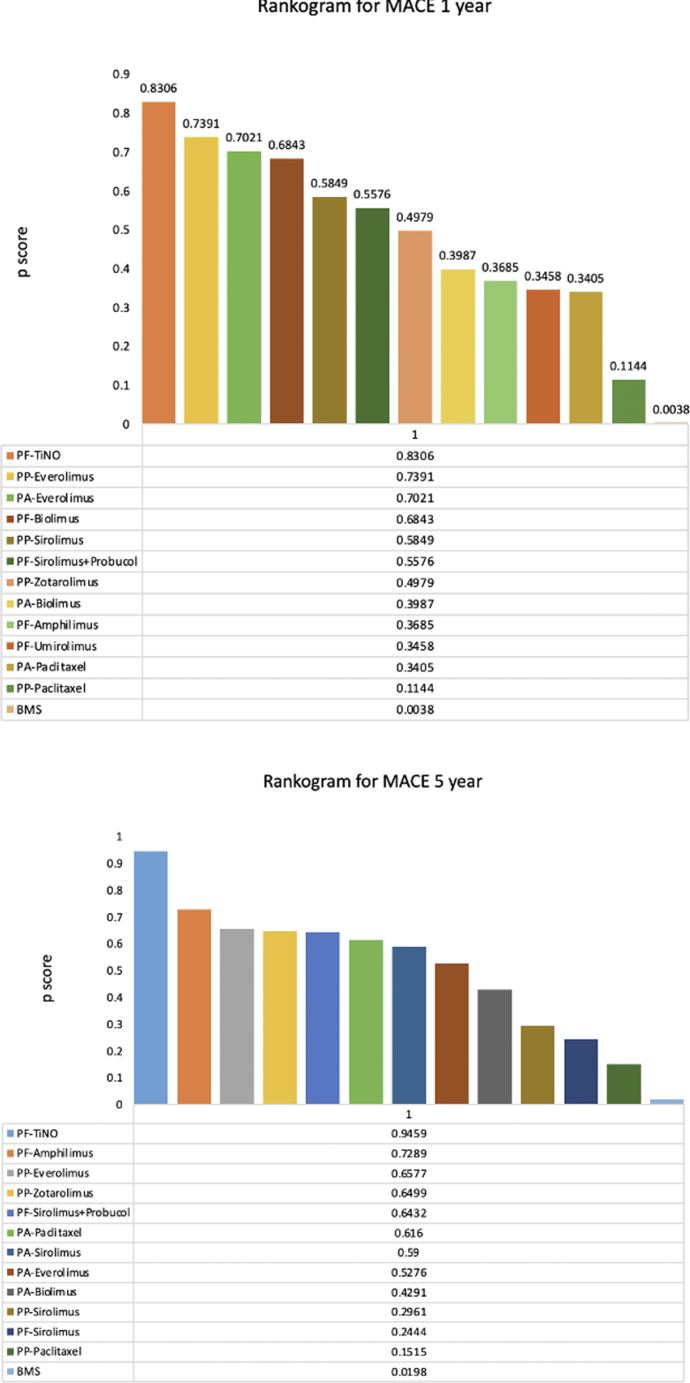
Rankogram for MACE for Experimental Vs Control Arms Maximum *P*-score is 1 and scores closer to 1 represent the highest performance. Abbreviations as in Figures 1 and 2.

Polymer-free TiNO was associated with the lowest risk of MACE at 1- (*P*-score 0.8306) and 5-year (*P*-score 0.9459). Polymer-free TiNO was also ranked the best for reducing the 1-year risk of MI (*P*-score 0.9641), ST (*P*-score 0.9655), and all-cause mortality (*P*-score 0.7275) followed by polymer-permanent everolimus stents. BMS and polymer-permanent paclitaxel stents had the worst ranking for all these outcomes (Figure 3, Supplemental Figures 1 and 12).

### Network consistency and heterogeneity

On NMA, there was no evidence of total (global), within-, and between-design (loop) inconsistency in MACE and all secondary outcomes (ST, MI, TLR, TVR, mortality, and CVD) (*P* > 005) at all timepoints (6 months, 1 and 5 years), except for TLR at 1 year that showed a significant within-design (*P* = 0.0002), loop (*P* = 0.036), and global inconsistency (*P* < 0.0001) (Supplemental Table 18). The within-design inconsistency was driven by the significant heterogeneity in the pairwise comparison of polymer-permanent everolimus, paclitaxel, and zotarolimus stents. L'abbe plot showed a symmetric distribution of studies on each side of the equality line, validating no heterogeneity (Supplemental Figure 13). The heatmap also showed minimal design-level inconsistency for MACE (Supplemental Figure 14).

### Sensitivity analyses

There was no major influence of any single RCT on net estimates of MACE and all-cause mortality (Supplemental Figure 15).

### Trial sequential analysis

**Figure 4:**
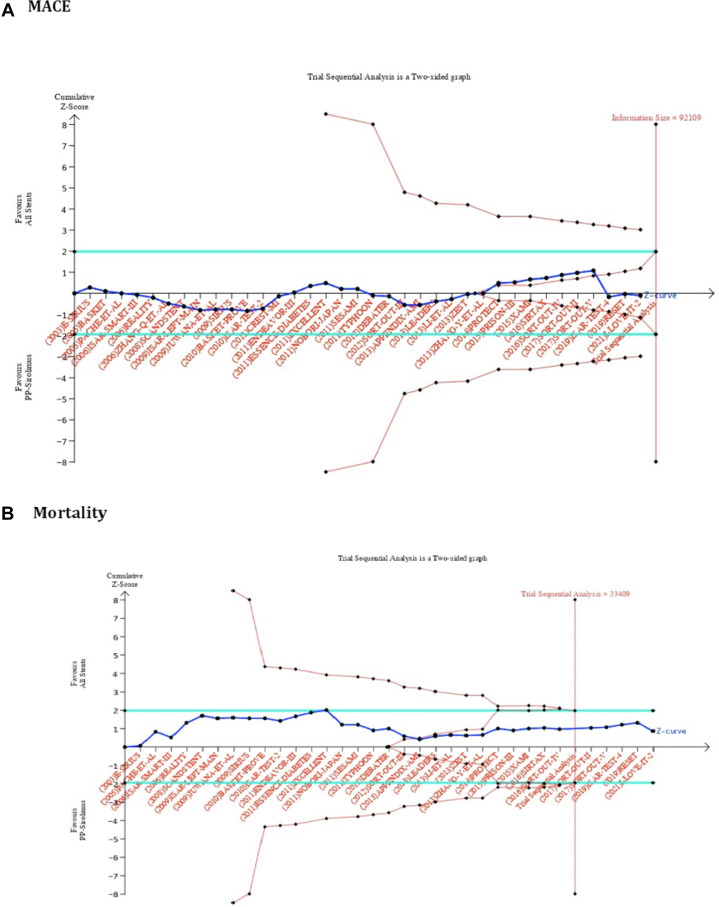
Red Vertical Line at the Right End Indicates the Required Information Size (Total Population), to Detect a Relative Risk Difference (RRD) of Direct Comparison Strategies With PP-SES at an Allowable Alpha Error of 5% and Power of 80% The black vertical line on the left side shows the scale of the cumulative z-score, where |Z| = 1.96 corresponds to a *P* = 0.05. The black central horizontal line represents the interim addition of RCTs in a chronological fashion. The green horizontal lines on each side signify the conventional boundaries for measured 95% CIs. The red dotted lines on each outer side of the conventional boundaries that taper toward the axis of the information size represent the monitoring boundaries of TSA (inward sloping), while the red dotted lines inside the conventional boundaries (outward sloping) represent the futility boundaries. The solid blue line represents the calculated cumulative point estimate (Z-curve) with each dot indicating a change in the net estimate with the addition of the new RCT. (A) The required information size to detect or reject a 1.96% RRD with a major adverse cardiovascular events (MACE) incidence of 14.83% in the PP-SES was 92,109. The TSA crossed the area of futility (outward red line slope), suggesting that the addition of future trials on pp-ses will unlikely reach the threshold of statistical significance. (B) The required information size to detect or reject the RRD with a mortality incidence of 9.28% in PP-SES was 33,409. With the addition of the SORT-OUT-II trial, the cumulative z line surpassed the required information size suggesting that sufficient evidence on mortality has been achieved. RCTs = randomized control trials; SES = sirolimus-eluting stent; TSA = trial sequential analysis; other abbreviation as in Figure 1.

TSA further validated the nonsignificant differences in the risk of MACE and all-cause mortality between the experimental and polymer-permanent sirolimus stents direct comparison groups at the interim analyses of the included trials. TSA also elucidated that statistically significant differences in the incidence of MACE between the comparison strategies are not expected with future RCTs as the cumulative Z-line crossed the futility area (Figure 4A). Similarly, the all-cause mortality findings were conclusive because the calculated cumulative estimates surpassed the information size (Figure 4B).

### Publication bias

The risk of publication bias for both MACE and mortality was low both by the visual criterion of symmetry of the funnel plots and by nonsignificant ERE (*P* = 0.80 and *P* = 0.99, respectively) (Supplemental Figure 16).

## Discussion

The current network demonstrates the following. 1) Compared with the control group, permanent-polymer everolimus and polymer-free TiNO stents were associated with 11%, and 22% lower 5-year risk of MACE, respectively. 2) The former was also associated with a lower 5-year risk of ST and the need for revascularization. 3) Polymer-free TiNO had the highest efficacy in terms of the lowest risk of ST and MI. 4) BMS had a greater risk of ST and MI, and a higher need for TLR and TVR irrespective of clinical presentation, DAPT duration, and follow-up periods. 5) A sequential analysis suggested that future RCTs on direct comparisons of polymer-permanent sirolimus stents are not required; both for incidence of MACE (as it is implausible that the aggregated estimates could become statistically significant) and all-cause mortality (because the required sample size for superiority is already obtained).

Prior studies on the utility of different stent designs have shown that delayed vascular healing is the major determinant of ST, contributing to the overall risk of adverse events after stent implantation.^19^^,^^20^ Recent advancements in DES were focused on the reduction of implantation-related vascular injury, polymer-associated inflammation, and stent-induced thrombosis. In our study, different stent strategies were directly or indirectly compared with each other and with the control group (polymer-permanent sirolimus stents). On a weighted estimate of 125 RCTs, polymer-permanent everolimus stents showed an impressive performance in terms of lowering the incidence of ST. A significant reduction in the risk of ST was evident as early as within 6 months, with no thrombotic late catch-up phenomenon thereafter up to 5 years. These benefits ultimately translated into a substantially lower incidence of pooled ischemic events, and need for revascularization at 5 years, not only compared with BMS but also with first-generation DES such as polymer-permanent sirolimus and paclitaxel stents. This suggests that a durable and relatively thinner fluoropolymer used in polymer-permanent everolimus stents might be “thromboresistant” and more biocompatible than first-generation polymers.^21^^,^^22^ Apart from this, better stent geometry and uniform coating of drugs presumably enabled a sustained release of antiproliferative drugs, favoring polymer-permanent everolimus stents over older-generation stents.^22^

In our study, the lower incidence of MACE and secondary efficacy endpoints at long-term follow-up with the next-generation polymer-free TiNO-coated stents appeared to be driven by the TIDE-ACS (Thin-strut DES in Extended duration dual antiplatelet therapy in Acute Coronary Syndrome), TiNOX (Titanium-Nitride-Oxide-coated stents in ACS), and TiTAX (Titanium-Nitride-Oxide Coated Stent versus TAXUS Drug-Eluting Stent) trials.^13^, ^14^, ^15^, ^16^, ^17^, ^18^ Mechanistically, these benefits could be explained by the use of a cobalt-chromium platform with ultrathin struts that have improved radial strength, lower risk of platelet adhesion, and reduced fibrinogen binding.^13^ The other plausible explanation for better outcomes in polymer-free TiNO would be the use of polymer-free instead of polymer-coated surface for drug adherence, allowing for earlier endothelialization and decreased inflammatory and thrombogenic responses to the polymer.^23^ In TIDES-ACS trials, intravascular imaging of patients treated with TiNO-coated stents showed a lower percentage of uncovered and malapposed struts but had more risk of intimal hyperplasia. The latter findings might be responsible for the relatively higher risk of revascularization with polymer-free TiNO. Nevertheless, the higher rate of revascularization could be considered a trade-off for the substantially lower incidence of ST and repeat MI events seen with these stents. Overall, these findings should be interpreted with caution, as only 6 trials contributed to the comparison, the included trials did not perform routine angiographic follow-up, and were underpowered to assess individual efficacy endpoints, potentially overestimating the net results.

Similar to polymer-free TiNO and polymer-permanent everolimus stents, sirolimus stents coated on bioabsorbable polymers showed a substantial reduction in the ST and MI events at both 1 and 5 years, compared with a similar eluting drug (sirolimus) coated on nondegradable polymer. These benefits clearly indicate that polymer-coating has a crucial role in the pathogenesis of ST and subsequent hard clinical outcomes. This attests that bioabsorbable polymer coatings preserve the efficacy of permanent coatings during the initial phase, providing higher antirestenotic properties, whereas simultaneously offering the safety of BMS (lower thrombogenicity) after the polymer dissolves.^24^ Although our net estimates favored polymer absorbable sirolimus stents over polymer-permanent sirolimus stents, the significance of *P* values was only modest in its statistical power, resulting in a nonsignificant difference in the primary composite endpoint, and the need for revascularization between the 2 stent designs.

The other 16 stent strategies showed a similar risk of primary ischemic endpoint and most of the secondary endpoints on all different comparisons. Lastly, the BMS and polymer-permanent paclitaxel stents persistently ranked the worst strategies due to a 1- to 3-fold higher incidence of ST, MI, and the need for revascularization at all timepoints. The results of the current study were concordant based on several subgroups and sensitivity analyses.

Our study expands the findings of previous meta-analyses that had conflicting findings. Importantly, previous analyses lacked stratification of outcomes based on clinical presentation, DAPT duration, and polymer type (Supplemental Table 19). By contrast, using 19 different stent types, our study is the most comprehensive analysis of 3 major polymer types, BMS and a myriad of DES. In a nutshell, we found that new-generation antiproliferative drug coating on the durable polymer (everolimus stents) or potentially on a polymer-free surface (TiNO) run in parallel with a reduction of definite ST rates, compared with old-generation polymer-permanent sirolimus stents and BMS.

### Study Limitations

The findings of our study should be interpreted with consideration of certain limitations. The inherent methodological variations across the included RCTs, such as differences in patient populations, timing of randomization, medication compliance, and outcome measurement may introduce bias in the pooled analysis. Although larger-scale trials were selected for subgroup analyses, the risk of type 1 error remains, as multiple comparisons increase the likelihood of false positive findings. Due to a limited number of studies, we couldn't determine the safety of novel stents in the setting of de-escalation therapies (<6-month DAPT). There were some sample size differences in the experimental and control arms of some of the included trials resulting in wide credibility intervals and imprecise estimates that should be interpreted with caution. Due to the lack of head-to-head comparisons of polymer-free TiNO with most of the older generation DES, we had to rely on our indirect estimates; however, the lack of significant inconsistency supports the reliability of our net results. While the new-generation polymer-free TiNO stents seem to have promising results in the initial trials, large-scale confirmatory trials are needed to validate these findings.

## Conclusions

Among 19 different stent designs, permanent-polymer everolimus and zotarolimus use followed by DAPT for 12 months might be the best strategy to reduce the long-term incidence of ischemic events and the need for revascularizations in all comers. Polymer-free TiNO is emerging as a potentially safer and more efficacious strategy, particularly in ACS patients. BMS and paclitaxel-eluting stents might be considered outdated since they carry a higher overall risk of complications irrespective of DAPT duration and clinical presentation of patients.

Perspectives **COMPETENCY IN MEDICAL KNOWLEDGE:** In post-PCI patients, permanent-polymer everolimus and zotarolimus stents appear to have the highest efficacy due to a significantly lower risk of ischemic events compared with older-generation stents.

**TRANSLATIONAL OUTLOOK:** Further studies are needed to determine the efficacy of TiNO stents.

## Funding support and author disclosures

The authors have reported that they have no relationships relevant to the contents of this paper to disclose.

## Supplementary data

Supplemental Tables 1, 2, 3, 4, 5, 6, 7, 8, 9, 10, 11, 12, 13, 14, 15, 16, 17, 18, and 19, and Figures 1, 2, 3, 4, 5, 6, 7, 8, 9, 10, 11, 12, 13, 14, 15, and 16
