# Supplementary material for: Safety and Efficacy of Different Stent Strategies in Percutaneous Coronary Intervention: A Network Meta-Analysis
Source: JACC Adv. 2025 Feb 18;4(3):101600. doi: 10.1016/j.jacadv.2025.101600 (PMC11883374; doi:10.1016/j.jacadv.2025.101600)
Supplement: Supplemental Tables 1, 2, 3, 4, 5, 6, 7, 8, 9, 10, 11, 12, 13, 14, 15, 16, 17, 18, and 19, and Figures 1, 2, 3, 4, 5, 6, 7, 8, 9, 10, 11, 12, 13, 14, 15, and 16 [file mmc1.docx]

**SUPPLEMENTAL APPENDIX**

**Additional text:**

**PICO:**

Problem/Population: Patients undergoing PCI for ACS or stable angina or silent ischemia

Intervention: Polymer-free, polymer coated or baremetal stent

Control: Sirolimus eluting permanent polymer coated stent

Outcomes: MACE, cardiovascular and all-cause mortality, stroke, stent thrombosis, myocardial infarction, target-lesion and target-vessel revascularization

**Search Map:**

(((((((((((((((((((((((((drug eluting stent AND ACS)) OR (drug eluting stent AND acute coronary syndrome)) OR (drug eluting stent AND CAD)) OR (zotarolimus stent AND randomized trials)) OR (everolimus stent AND randomized trials)) OR (paclitaxel stent AND randomized trials)) OR (sirolimus stent AND randomized trials)) OR (endeavor zotarolimus stent AND randomized trials)) OR (r solute zotarolimus stent AND randomized trials)) OR (biodegradable polymer stent AND randomized trials)) OR (bioabsorbable stent AND randomized trials)) OR (polymer free stent AND randomized trials)) OR (biolimus stent AND randomized trials)) OR biolimus stent) OR (umirolimus stent AND randomized trials)) OR umirolimus stent) OR sirolimus stent) OR paclitaxel stent) OR everolimus stent OR everolimus stent) OR zotarolimus stent) OR endeavor zotarolimus-stent) OR r solute zotarolimus stent) OR biodegradable polymer stent

**Supplemental Table 1: Preferred Reporting Items for Systematic Reviews and Meta‐Analyses (PRISMA) Network Meta-analysis (NMA) Checklist Items**

| **Section/Topic** | **Item #** | **Checklist Item** | **Reported on Page #** |
| --- | --- | --- | --- |
| **TITLE** |  |  |  |
| Title | 1 | Identify the report as a systematic review *incorporating a network meta-analysis (or related form of meta-analysis).* | 1 |
|  |  |  |  |
| **ABSTRACT** |  |  |  |
| Structured summary | 2 | Provide a structured summary including, as applicable:  **Background:** main objectives  **Methods:** data sources; study eligibility criteria, participants, and interventions; study appraisal; and *synthesis methods, such as network meta-analysis.*  **Results:** number of studies and participants identified; summary estimates with corresponding confidence/credible intervals; *treatment rankings may also be discussed. Authors may choose to summarize pairwise comparisons against a chosen treatment included in their analyses for brevity.*  **Discussion/Conclusions:** limitations; conclusions and implications of findings.  **Other:** primary source of funding; systematic review registration number with registry name. | 2 |
|  |  |  |  |
| **INTRODUCTION** |  |  |  |
| Rationale | 3 | Describe the rationale for the review in the context of what is already known*, including mention of why a network meta-analysis has been conducted.* | 4 |
| Objectives | 4 | Provide an explicit statement of questions being addressed, with reference to participants, interventions, comparisons, outcomes, and study design (PICOS). | 4 |
|  |  |  |  |
| **METHODS** |  |  |  |
| Protocol and registration | 5 | Indicate whether a review protocol exists and if and where it can be accessed (e.g., Web address); and, if available, provide registration information, including registration number. | 4 |
| Eligibility criteria | 6 | Specify study characteristics (e.g., PICOS, length of follow-up) and report characteristics (e.g., years considered, language, publication status) used as criteria for eligibility, giving rationale. *Clearly describe eligible treatments included in the treatment network, and note whether any have been clustered or merged into the same node (with justification).* | **5** |
| Information sources | 7 | Describe all information sources (e.g., databases with dates of coverage, contact with study authors to identify additional studies) in the search and date last searched. | 4 |
| Search | 8 | Present full electronic search strategy for at least one database, including any limits used, such that it could be repeated. | 4 |
| Study selection | 9 | State the process for selecting studies (i.e., screening, eligibility, included in systematic review, and, if applicable, included in the meta-analysis). | 5 |
| Data collection process | 10 | Describe method of data extraction from reports (e.g., piloted forms, independently, in duplicate) and any processes for obtaining and confirming data from investigators. | 4 |
| Data items | 11 | List and define all variables for which data were sought (e.g., PICOS, funding sources) and any assumptions and simplifications made. | 4 |
| **Geometry of the network** | **S1** | Describe methods used to explore the geometry of the treatment network under study and potential biases related to it. This should include how the evidence base has been graphically summarized for presentation, and what characteristics were compiled and used to describe the evidence base to readers. | 5-6 |
| Risk of bias within individual studies | 12 | Describe methods used for assessing risk of bias of individual studies (including specification of whether this was done at the study or outcome level), and how this information is to be used in any data synthesis. | 5-6 |
| Summary measures | 13 | State the principal summary measures (e.g., risk ratio, difference in means). *Also describe the use of additional summary measures assessed, such as treatment rankings and surface under the cumulative ranking curve (SUCRA) values, as well as modified approaches used to present summary findings from meta-analyses.* | 5-6 |
| Planned methods of analysis | 14 | Describe the methods of handling data and combining results of studies for each network meta-analysis. This should include, but not be limited to:  · *Handling of multi-arm trials;*  · *Selection of variance structure;*  · *Selection of prior distributions in Bayesian analyses; and*  ·  *Assessment of model fit.* | 5-6 |
| **Assessment of Inconsistency** | **S2** | Describe the statistical methods used to evaluate the agreement of direct and indirect evidence in the treatment network(s) studied. Describe efforts taken to address its presence when found. | 5-6 |
| Risk of bias across studies | 15 | Specify any assessment of risk of bias that may affect the cumulative evidence (e.g., publication bias, selective reporting within studies). | 5-6 |
| Additional analyses | 16 | Describe methods of additional analyses if done, indicating which were pre-specified. This may include, but not be limited to, the following:  · Sensitivity or subgroup analyses;  · Meta-regression analyses;  · *Alternative formulations of the treatment network; and*  · *Use of alternative prior distributions for Bayesian analyses (if applicable).* | 6 |
|  |  |  |  |
| **RESULTS** |  |  |  |
| Study selection | 17 | Give numbers of studies screened, assessed for eligibility, and included in the review, with reasons for exclusions at each stage, ideally with a flow diagram. | 6 |
| **Presentation of network structure** | **S3** | Provide a network graph of the included studies to enable visualization of the geometry of the treatment network. | 6 |
| **Summary of network geometry** | **S4** | Provide a brief overview of characteristics of the treatment network. This may include commentary on the abundance of trials and randomized patients for the different interventions and pairwise comparisons in the network, gaps of evidence in the treatment network, and potential biases reflected by the network structure. | 6 |
| Study characteristics | 18 | For each study, present characteristics for which data were extracted (e.g., study size, PICOS, follow-up period) and provide the citations. | 6-7 |
| Risk of bias within studies | 19 | Present data on risk of bias of each study and, if available, any outcome level assessment. | 6-7 |
| Results of individual studies | 20 | For all outcomes considered (benefits or harms), present, for each study: 1) simple summary data for each intervention group, and 2) effect estimates and confidence intervals. *Modified approaches may be needed to deal with information from larger networks.* | 7-9 |
| Synthesis of results | 21 | Present results of each meta-analysis done, including confidence/credible intervals. *In larger networks, authors may focus on comparisons versus a particular comparator (e.g. placebo or standard care), with full findings presented in an appendix. League tables and forest plots may be considered to summarize pairwise comparisons.* If additional summary measures were explored (such as treatment rankings), these should also be presented. | 7-9 |
| **Exploration for inconsistency** | **S5** | Describe results from investigations of inconsistency. This may include such information as measures of model fit to compare consistency and inconsistency models, *P* values from statistical tests, or summary of inconsistency estimates from different parts of the treatment network. | 7-9 |
| Risk of bias across studies | 22 | Present results of any assessment of risk of bias across studies for the evidence base being studied. | 10 |
| Results of additional analyses | 23 | Give results of additional analyses, if done (e.g., sensitivity or subgroup analyses, meta-regression analyses*, alternative network geometries studied, alternative choice of prior distributions for Bayesian analyses,* and so forth). | 8-9 |
|  |  |  |  |
| **DISCUSSION** |  |  |  |
| Summary of evidence | 24 | Summarize the main findings, including the strength of evidence for each main outcome; consider their relevance to key groups (e.g., healthcare providers, users, and policy-makers). | 10 |
| Limitations | 25 | Discuss limitations at study and outcome level (e.g., risk of bias), and at review level (e.g., incomplete retrieval of identified research, reporting bias). *Comment on the validity of the assumptions, such as transitivity and consistency. Comment on any concerns regarding network geometry (e.g., avoidance of certain comparisons).* | 12 |
| Conclusions | 26 | Provide a general interpretation of the results in the context of other evidence, and implications for future research. | 13 |
|  |  |  |  |
| **FUNDING** |  |  |  |
| Funding | 27 | Describe sources of funding for the systematic review and other support (e.g., supply of data); role of funders for the systematic review. This should also include information regarding whether funding has been received from manufacturers of treatments in the network and/or whether some of the authors are content experts with professional conflicts of interest that could affect use of treatments in the network. | 1 |

PICOS = population, intervention, comparators, outcomes, study design.

**Supplemental Table 2: Detailed trial-level study characteristics and demographics of the included population in intervention (i) and control (c) groups.**

| STUDY | Author | year | Design | Country | FU | Total | **Stent Comparison** | | **Age (Mean±SD** | | **Male n(%)** | |
| --- | --- | --- | --- | --- | --- | --- | --- | --- | --- | --- | --- | --- |
|  |  |  |  |  |  |  | **Intervention** | **Comparison** | **Intervention** | **Comparison** | **Intervention** | **Comparison** |
| ACTION [1] | Serruys | 2004 | RCT | Multiple | 12 | 360 | PF-DES | BMS | 60.5±11 | 60±10 | 79(66) | 78(66) |
| APPENDIX-AMI [2] | Velders | 2013 | RCT | Netherlands | 24 | 977 | PP-EES | PP-SES | 65.3±11.3 | 65.0±11.2 | 353(70.9) | 347(72.4) |
| BASKET [3] | Kiaser | 2005 | RCT | Switzerland | 6 | 826 | PP-SES | BMS | 64±12 | 64±11 | 209(79) | 223(79) |
| BASKET | Kiaser | 2005 | RCT | Switzerland | 6 | 826 | PP-PES | BMS | 64±11 | 64±11 | 218(78) | 223(79) |
| BASKET-PROVE [4] | Kiaser | 2010 | RCT | Multiple | 24 | 2323 | PP-SES | BMS | 66±11 | 67±11 | 576(74) | 586(77) |
| BASKET-PROVE | Kiaser | 2010 | RCT | Multiple | 24 | 2323 | PP-EES | BMS | 66±11 | 67±11 | 587(76) | 586(77) |
| BASKET-PROVE II [5] | Kiaser | 2014 | RCT | Multiple | 24 | 2291 | PA-BES | BMS | 62±11 | 63±11 | 600(78) | 570(75) |
| BASKET-PROVE II | Kiaser | 2014 | RCT | Multiple | 24 | 2291 | PP-EES | BMS | 62±11 | 63±11 | 610(80) | 570(75) |
| BASKET-SMALL [6] | Jeger | 2016 | RCT | Switzerland | 24 | 191 | PP-ZES | PP-PES | 64±11.9 | 65.2±10.7 | 72(79.1) | 79(79) |
| BIODEGRADE [7] | Yoon | 2020 | RCT | South Korea | 18 | 2327 | PA-BES | PA-SES | 63.6±11 | 63.4±10.7 | 838(72.2) | 835(71.6) |
| BIOFLOW II [8] | Windecker | 2015 | RCT | Multiple | 12 | 452 | PA-SES | PP-EES | 62.7±10.4 | 64.8±9.2 | 233(78.2) | 115(74.7) |
| BIOFLOW IV [9] | Saito | 2019 | RCT | Multiple | 12 | 575 | PA-SES | PP-EES | 64.8±9.6 | 64.4±9.8 | 280(72.7) | 146(76.8) |
| BIOFLOW V [10] | Kandzari | 2017 | RCT | Multiple | 12 | 1334 | PA-SES | PP-EES | 64.5±10.3 | 64.6±10.7 | 660(74.7) | 328(72.9) |
| BIOFLOW VI [11] | Li | 2020 | RCT | China | 12 | 440 | PA-SES | PP-EES | 59.1±8.5 | 58.4±8.6 | 160(72.7) | 142(64.5) |
| BIOFREEDOM FIM [12] | Cost | 2016 | RCT | Germany | 60 | 182 | PF-BES | PP-PES | 66.8±9.2 | 67.9±8 | 43.5(71.3) | 40(66.7) |
| BIOSTEMI [13] | Iglesias | 2019 | RCT | Switzerland | 12 | 1300 | PA-SES | PP-EES | 62.2±11.8 | 63.2±11.8 | 513(79) | 477(73) |
| BIONICS [14] | Kandzari | 2017 | RCT | Multiple | 12 | 1919 | PP-RES | PP-ZES | 63.7±10.2 | 63.1±10.3 | 750(78.3) | 787(81.9) |
| BIONYX [15] | Birgelen | 2018 | RCT | Multiple | 12 | 2488 | PP-ZES | PA-SES | 64.1±10.9 | 63.9±11.2 | 946(76.1) | 948(76.1) |
| BIO-RESORT [16] | Birgelen | 2016 | RCT | Netherlands | 12 | 3514 | PP-EES | PA-ZES | 64±10.7 | 63.6±10.9 | 845(72) | 848(72) |
| BIO-RESORT | Birgelen | 2016 | RCT | Netherlands | 12 | 3514 | PA-SES | PA-ZES | 64.2±10.7 | 63.6±10.9 | 854(73) | 848(72) |
| BIOSCIENCE [17] | Pilgrim | 2014 | RCT | Switzerland | 12 | 2119 | PA-SES | PP-EES | 66.1±11.6 | 65.9±11.4 | 818(77) | 816(77.3) |
| CATOS [18] | Park | 2012 | RCT | South Korea | 12 | 160 | PP-ZES | PP-SES | 62.7±12.3 | 63±11.7 | 52(65) | 61(76) |
| CERVINKA ET AL [19] | Cervinka | 2006 | RCT | Czech Republic | 6 | 70 | PP-SES | PP-PES | 56±10.2 | 55±13.3 | 27(74) | 24(72) |
| CENTURY II [20] | Saito | 2014 | RCT | Multiple | 9 | 1101 | PA-SES | PP-EES | 65±11 | 66±11 | 433(78.6) | 453(82.4) |
| CHOICE [21] | Youn | 2021 | RCT | South Korea | 24 | 1911 | PP-EES | PA-BES | 65.6±11 | 65±11.6 | 422(66.1) | 445(70.2) |
| CHOICE | Youn | 2021 | RCT | South Korea | 24 | 1911 | PA-BES | PP-ZES | 65±11.6 | 64±11.3 | 445(70.2) | 429(67.1) |
| CORACTO [22] | Reifart | 2010 | RCT | Germany | 24 | 91 | BMS | PA-SES | 64.8±8.9 | 64.7±9.9 | 37(82.2) | 32(69.6) |
| COMFORTABLE AMI [23] | Raber | 2012 | RCT | Multiple | 12 | 1161 | PA-BES | BMS | 60.7±11.6 | 60.4±11.9 | 463(80.5) | 455(78.2) |
| COMPARE [24] | Kedhi | 2010 | RCT | Netherlands | 12 | 1800 | PP-EES | PP-PES | 62.9 | 63.6 | 619(69) | 654(72) |
| COMPARE II [25] | Smits | 2013 | RCT | Multiple | 12 | 2707 | PA-BES | PP-EES | 63±11.1 | 62.7±11 | 1336(74.4) | 678(74.3) |
| COSTAR II [26] | Krucoff | 2008 | RCT | Multiple | 8 | 1700 | PA-PES | PP-PES | 63.5±10.8 | 63.7±10.6 | 723(73.1) | 488(71.1) |
| CREST MI [27] | Wang | 2011 | RCT | China | 6 | 875 | PP-ZES | PP-SES | 59.6 | 60.2 | 366(81.6) | 348(81.8) |
| CRE8 (NEXT) [28] | Carrie | 2012 | RCT | Multiple | 12 | 323 | PF-AES | PP-PES | 64.9±10.2 | 64.39±10.45 | 124(76.5) | 109(67.7) |
| C-SIRIUS [29] | Schampaert | 2004 | RCT | Canada | 9 | 100 | PP-SES | BMS | 60.3±10.6 | 60.7±9.1 | 35(70) | 34(68) |
| Dang et al [30] | Dang | 2012 | RCT | China | 12 | 105 | PP-SES | PF-PES | 67.1±12.5 | 65.2±13.8 | 39(70.9) | 34(68) |
| DEBATER [31] | Wijnbergen | 2012 | RCT | Netherlands | 12 | 870 | PP-SES | BMS | 60±11 | 61±11 | 332(78) | 336(76) |
| DEBFIRST [32] | Chae | 2017 | RCT | South Korea | 12 | 180 | PF-PES+BMS | PP-ZES | 61.2±11.1 | 62.4±11.9 | 68(75.6) | 63(70) |
| DECODE [33] | Chan | 2008 | RCT | Multiple | 12 | 83 | NA-SES | BMS | 58.7±9.7 | 62.5±10.3 | 37(69) | 19(66) |
| DEDICATION [34] | Kelbaek | 2008 | RCT | Denmark | 8 | 626 | NA-DES | BMS | 61.8 | 62.6 | 228(72.8) | 230(73.5) |
| DES-DIABETES [35] | Lee | 2008 | RCT | South Korea | 9 | 400 | NA-SES | NA-PES | 61.1±8.9 | 60.7±8.8 | 122(61) | 110(55) |
| DESSERT [36] | Maresta | 2008 | RCT | Italy | 12 | 150 | PP-SES | BMS | 71±9 | 69±9 | 47(63) | 37(49) |
| DESSOLVE II [37] | Wijns | 2015 | RCT | Belgium | 9 | 184 | PA-SES | PP-ZES | 65±10.4 | 65.1±10.5 | 85(69.1) | 45(73.8) |
| DESSOLVE III [38] | DeWinter | 2017 | RCT | Multiple | 12 | 1398 | PA-SES | PP-EES | 66.4±10.7 | 66.3±10.7 | 494(70) | 513(74) |
| DIABEDES [39] | Maeng | 2009 | RCT | Denmark | 8 | 153 | PP-SES | PP-PES | 66±8 | 65±10 | 64(84) | 57(74) |
| DIABETES [40] | Sabate | 2005 | RCT | Spain | 9 | 160 | PP-SES | BMS | 65.9±9 | 67.2±10 | 50(62.5) | 50(62.5) |
| Diaz de la llera et al [41] | Diaz de la llera | 2007 | RCT | Spain | 12 | 120 | BMS | PP-SES | 65±13 | 64±12 | 47(78.3) | 48(80) |
| DIRECT II [42] | Verheye | 2016 | RCT | Multiple | 12 | 159 | PA-SES | PP-ZES | 62.7±9.9 | 64.2±12.4 | 82(75.9) | 34(66.7) |
| ENDEAVOR II [43] | Fajadet | 2006 | RCT | Multiple | 9 | 1197 | PP-ZES | BMS | 61.6±10.5 | 61.9±10.5 | 460(77) | 449(75) |
| ENDEAVOR III [44] | Kandzari | 2006 | RCT | USA | 9 | 436 | PP-ZES | PP-SES | 61.42±10.58 | 61.73±11.59 | 211(65.3) | 92(81.4) |
| ENDEAVOR IV [45] | Leon | 2010 | RCT | USA | 12 | 1548 | PP-ZES | PP-PES | 63.5±11.1 | 63.6±11.0 | 517(66.9) | 531(68.5) |
| Erglis et al [46] | Erglis | 2007 | RCT | Australia | 6 | 103 | BMS | NA-PES | 61.08±10.28 | 62.56±11.45 | 45(85) | 41(42) |
| ESSENCE-DIABETES [47] | Kim | 2011 | RCT | South Korea | 12 | 300 | PP-EES | PP-SES | 63.2±8.3 | 63.5±8.1 | 78(52.3) | 99(65.6) |
| EUCATAX [48] | Rodriguez | 2011 | RCT | Argentina | 12 | 422 | PA-PES | BMS | 63.8±10.2 | 64.7±12.2 | 176(83.4) | 167(79.1) |
| EVERBIO II [49] | Puricel | 2015 | RCT | Switzerland | 9 | 240 | PA-EES | PA-BES | 65±11 | 65±10 | 64(80) | 64(80) |
| EVOLVE [50] | Meredith | 2012 | RCT | Multiple | 6 | 192 | PP-EES | PA-EES | 62.1±10 | 64.9±11 | 78(79.6) | 66(69.9) |
| EVOLVE II [51] | Kereiakes | 2015 | RCT | Multiple | 12 | 1684 | PA-EES | PP-EES | 63.5±10.4 | 63.9±10.5 | 597(70.6) | 609(72.7) |
| EXAMINATION [52] | Brugaletta | 2021 | RCT | Spain | 120 | 1498 | PP-EES | BMS | 60.8±12 | 61.6±13 | 634(84) | 610(82) |
| EXCELLA II [53] | Serruys | 2010 | RCT | Multiple | 9 | 210 | PP-NES | PP-ZES | 64.7±9.6 | 62.7±9.7 | 106(76.3) | 56(78.9) |
| EXCELLENT [54] | Park | 2011 | RCT | South Korea | 12 | 1443 | PP-EES | PP-SES | 62.5±10.1 | 63.4±9.9 | 703(65.2) | 228(62.6) |
| E-SIRIUS [55] | Schofer | 2003 | RCT | Europe | 9 | 352 | PP-SES | BMS | 62.0±11.4 | 62.6±10.3 | 123(70) | 126(71) |
| FIBISTEMI [56] | Gao | 2007 | RCT | China | 6 | 156 | NA-SES | BMS | 57.8±11.7 | 59.7±11.1 | 84(83.1) | 42(76) |
| GARA-GARA [57] | Fukumoto | 2011 | RCT | Japan | 8 | 800 | PP-SES | PP-PES | 69±9 | 69±9 | 324(71) | 334(71) |
| GENESIS [58] | Verheye | 2009 | RCT | Multiple | 6 | 249 | PA-PES | NA-Pim | 64.4±9.6 | 64.1±10.0 | 35(71.4) | 80(80) |
| GENESIS | Verheye | 2009 | RCT | Multiple | 6 | 249 | PA-PES | NA-DES | 64.4±9.6 | 59.9±10.1 | 35(71.4) | 76(78.4) |
| GRACIA-3 [59] | Sanchez | 2010 | RCT | Spain | 12 | 433 | PP-PES | BMS | 62 | 61.5 | 173(79.7) | 185(85.6) |
| GISSOC II-GISE [60] | Rubartelli | 2010 | RCT | Italy | 24 | 152 | PP-SES | BMS | 63.9±9.6 | 63.9±9.8 | 58(78.3) | 68(87.1) |
| HARMONEE [61] | Saito l | 2018 | RCT | Multiple | 12 | 572 | PA-SES | PP-EES | 67.6±9.6 | 66.5±10.4 | 211(73.5) | 212(74.3) |
| Herdeg C et al. [62] | Herdeg | 2009 | RCT | Germany | 6 | 134 | PP-PES | BMS | 65.7±8.4 | 64.7±8.8 | 45(67) | 59(87) |
| Hong et al [63] | Hong | 2010 | RCT | South Korea | 36 | 169 | PP-SES | PP-PES | 65.9±8.0 | 64.5±8.9 | 61(71.8) | 64(76.2) |
| HORIZONS-AMI [64] | Stone | 2009 | RCT | USA | 12 | 3006 | PP-PES | BMS | 59.9 | 59.3 | 1738(77.0) | 569(76.0) |
| HOST-ASSURE [65] | Park | 2014 | RCT | South Korea | 12 | 3755 | NA-EES | NA-ZES | 63.1±10.8 | 63.5±10.7 | 1746(69.8) | 820(65.6) |
| INSPIRON I [66] | Oliviera | 2015 | RCT | Brazil | 48 | 57 | NA-SES | BMS | 58.9 | 62.8 | 24(63.2) | 9(47.4) |
| ISAR-DIABETES [67] | Dibra | 2005 | RCT | Germany | 9 | 250 | PP-SES | PP-PES | 67.7±10.2 | 68.3±9.6 | 93(74.4) | 89(71.2) |
| ISAR-LEFT-MAIN [68] | Mehilli | 2009 | RCT | Germany | 12 | 607 | PP-SES | PP-PES | 69.3±9.34 | 68.8±10.1 | 243(80) | 225(75) |
| ISAR-LEFT-MAIN II [69] | Mehilli | 2013 | RCT | Germany | 12 | 650 | PP-ZES | PP-EES | 69.4±10.4 | 70.2±9.4 | 236(72) | 252(77.3) |
| ISAR-SMART III [70] | Mehilli | 2006 | RCT | Germany | 12 | 360 | PP-PES | PP-SES | 65.7±10.4 | 67.4±10.9 | 135(75) | 125(69) |
| ISAR-TEST [71] | Mehilli | 2006 | RCT | Germany | 9 | 450 | PF-SES | PP-PES | 66.8±10.5 | 66.6±10.2 | 169(75) | 177(79) |
| ISAR-TEST-2 [72] | Byrne | 2009 | RCT | Germany | 12 | 1007 | PF-RP | PP-SES | 67.0±11.2 | 66.6±11.1 | 257(77.2) | 259(77.3) |
| ISAR-TEST-2 | Byrne | 2009 | RCT | Germany | 12 | 1007 | PP-ZES | PP-SES | 67.2±10.9 | 66.6±11.1 | 256(75.5) | 259(77.3) |
| ISAR-TEST-4 [73] | Byrne | 2009 | RCT | Germany | 12 | 2603 | PA-SES | PP-DES | 66.7±10.7 | 66.8±11.1 | 978(75.3) | 1002(76.8) |
| ISAR-TEST-5 [74] | Massberg | 2011 | RCT | Germany | 12 | 3002 | PF-SES+Probucol | PP-ZES | 67.7±11.2 | 68.1±10.8 | 1532(76.5) | 763(76.3) |
| I-LOVE-IT 2 [75] | Han | 2014 | RCT | China | 12 | 2737 | PA-SES | PP-SES | 60.2±10.1 | 60.2±10 | 1243(68) | 636(70) |
| J-DESSERT [76] | Nakamura | 2014 | RCT | Japan | 12 | 3348 | NA-SES | NA-PES | 69.5±9.0 | 69.3±9.4 | 1201(72.2) | 1223(72.6) |
| Juwana et al [77] | Juwana | 2009 | RCT | Netherlands | 12 | 397 | PP-SES | PP-PES | 61±12 | 61±11 | 135(69) | 149(74) |
| Kamoi et al [78] | Kamoi | 2011 | RCT | Japan | 8 | 100 | PP-SES | PP-PES | 65±9 | 64±9 | 39(78) | 35(70) |
| Kim et al [79] | Kim | 2008 | RCT | South Korea | 6 | 169 | PP-SES | PP-PES | 62.9±8.0 | 61.5±8.9 | 61(71.8) | 64(76.2) |
| KOMER [80] | Kang | 2011 | RCT | South Korea | 18 | 611 | NA-ZES | NA-SES | 60±13 | 59±12 | 156(76) | 166(81) |
| KOMER | Kang | 2011 | RCT | South Korea | 18 | 611 | NA-ZES | NA-PES | 60±13 | 60±13 | 156(76) | 160(79) |
| Laarman et al [81] | Laarman | 2006 | RCT | Netherlands | 12 | 619 | NA-PES | BMS | 61±12 | 61±13 | 229(73.9) | 241(78) |
| Lansky et al [82] | Lansky | 2000 | RCT | USA | 12 | 755 | BMS | BMS | 61±11 | 61±11 | 261(68.7) | 268(71.5) |
| LEADERS [83] | Windecker | 2008 | RCT | Multiple | 9 | 1707 | PA-BES | PP-SES | 64.6±10.8 | 64.5±10.7 | 643(75.0) | 634(74.6) |
| LEADERS FREE [84] | Urban | 2015 | RCT | Multiple | 13 | 2466 | PF-BES | BMS | 75.7±9.4 | 75.7±9.3 | 857(70.2) | 837(69.1) |
| Li et al [85] | Li | 2013 | RCT | China | 36 | 338 | PA-SES | PP-SES | 59.95±11.19 | 59.77±11.79 | 122(74.4) | 130(77.4) |
| LIPSIA YUKON [86] | Desch | 2011 | RCT | Germany | 9 | 240 | PF-SES | PP-PES | 67.0±9.5 | 67.3±9.1 | 83(69) | 79(68) |
| LISAII [87] | Shiratori | 2014 | RCT | Spain | 24 | 164 | PP-PES | PF-PES | 67.2±10.5 | 65.9±8 | 55(68.8) | 64(76.2) |
| LONG-DES II [88] | Kim | 2006 | RCT | South Korea | 9 | 500 | NA-SES | NA-PES | 61.4±9.0 | 60.7±9.0 | 168(67.2) | 153(61.2) |
| LONG-DES III [89] | Park | 2011 | RCT | South Korea | 12 | 450 | NA-EES | NA-SES | 62.9±9.9 | 63.0±9.7 | 165(73.7) | 149(65.9) |
| LONG-DES IV [90] | Ahn | 2012 | RCT | South Korea | 12 | 500 | NA-ZES | NA-SES | 62.8±9.7 | 62.7±9.8 | 184(73.6) | 181(72.4) |
| LONG-DES V [91] | Lee | 2014 | RCT | South Korea | 12 | 500 | PA-BES | PP-EES | 63.1±10.5 | 63.5±10.6 | 167(68.2) | 184(72.2) |
| MASTER [92] | Stone | 2012 | RCT | Multiple | 1 | 433 | BMS | NA-D/BMS | 60(52-68) | 58(51-67) | 163(75.1) | 166(76.9) |
| MASTER STUDY [93] | Valdes-chavarri | 2019 | RCT | Spain | 12 | 500 | BMS | PA-SES | 60±11 | 62±11 | 304(81.1) | 100(80.1) |
| MERIT-V [94] | Abizaid | 2018 | RCT | Multiple | 9 | 256 | PA-SES | PP-EES | 64.3±9.5 | 64.7±8.9 | 111(65.3) | 53(61.6) |
| MISSION [95] | Van der Hieven | 2008 | RCT | Netherlands | 12 | 310 | PP-SES | BMS | 59.2±11.2 | 59.1±11.6 | 118(74.7) | 123(80.9) |
| MULTISTRATEGY [96] | Valgimigli | 2008 | RCT | Multiple | 8 | 745 | NA-SES | BMS | 62.7±11.2 | 63.9±11.7 | 281(75.5) | 284(76.3) |
| NAPLES-DIABETES [97] | Briguori | 2011 | RCT | Italy | 36 | 226 | NA-SES | NA-PES | 64±8 | 64±10 | 43(57) | 44(59) |
| NAPLES-DIABETES | Briguori | 2011 | RCT | Italy | 36 | 226 | NA-SES | NA-EES | 64±8 | 65±8 | 43(57) | 42(56) |
| NEVO RES-I [98] | Ormiston | 2010 | RCT | Multiple | 6 | 394 | PA-SES | PP-PES | 63±10 | 64.4±9.9 | 158(78) | 143(74) |
| NEXT [99] | Natsuaki | 2013 | RCT | Japan | 12 | 3241 | PA-BES | PP-EES | 69.1±9.8 | 69.3±9.8 | 1245(77) | 1253(77) |
| NOBORI I [100] | Chevalier | 2015 | RCT | Multiple | 60 | 363 | PA-BES | PP-PES | 63.2±10.6 | 62.9±10 | 173(72.7) | 85(68) |
| NOBORI JAPAN [101] | Kadota | 2011 | RCT | Japan | 9 | 335 | PA-BES | PP-SES | 67.1±10.3 | 67.6±9.3 | 139(71.6) | 95(72) |
| NORSTENT [102] | Bonna | 2016 | RCT | Norway | 72 | 9013 | NA-EES/ZES | BMS | 62.6±10.8 | 62.6±10.9 | 3377(75) | 3380(75) |
| OCT DES [103] | Hamshere | 2018 | RCT | UK | 6 | 60 | PP-EES | PP-ZES | 59.2±9.8 | 62.6±12 | 22(73.3) | 23(76.6) |
| OCTDESI [104] | Guagliumi | 2010 | RCT | Italy | 12 | 60 | PP-PES | PP-PES | 65.05±7.65 | 63.1±8.2 | 34(82.9) | 15(78.9) |
| ONYX ONE [105] | Windecker | 2020 | RCT | Multiple | 12 | 1996 | PP-ZES | PF-UES | 74±9.5 | 74.1±9.8 | 677(67.5) | 653(65.8) |
| ORIENT [106] | Kang | 2017 | RCT | south korea | 12 | 372 | PA-SES | PP-ZES | 65.2±11.9 | 64.8±11 | 180(72) | 86(70.5) |
| ORTALANI [107] | Ortalani | 2007 | RCT | Italy | 12 | 104 | PP-SES | BMS | 65.3±9.9 | 65.9±9 | 38(73) | 41(79) |
| Pache et al [108] | Pache | 2005 | RCT | Germany | 12 | 500 | PP-SES | BMS | 67.4(59-75.4) | 66.7(59.9-74.7) | 196(78) | 195(78) |
| PAINT [109] | Lemos | 2012 | RCT | Brazil | 36 | 274 | PA-PES | BMS | 60.1±10.2 | 58.5±9.6 | 68(61.3) | 38(66.7) |
| PAINT | Lemos | 2012 | RCT | Brazil | 36 | 274 | PA-SES | BMS | 59.7±10.6 | 58.5±9.7 | 71(67) | 38(66.7) |
| PANDA III [110] | Xu | 2016 | RCT | China | 12 | 2348 | PA-SES | PA-SES | 60.8±10.6 | 61.5±10.6 | 828(70.5) | 830(70.7) |
| PASEO [111] | Lorenzo | 2009 | RCT | Italy | 24 | 270 | BMS | NA-PES | 63±15 | 62±17 | 62(68.9) | 64(71.1) |
| PASEO | Lorenzo | 2009 | RCT | Italy | 24 | 270 | BMS | NA-SES | 63±15 | 62±17 | 62(68.9) | 64(71.1) |
| PASSION [112] | Laarman | 2006 | RCT | Netherlands | 12 | 619 | BMS | NA-PES | 61±12 | 61±13 | 229(73.9) | 241(78.0) |
| Petronio et al [113] | Petronio | 2007 | RCT | Italy | 9 | 101 | PP-PES | PP-SES | 61±11 | 64±10 | 36(85.7) | 34(79.1) |
| PIONEER III [114] | Lansky | 2021 | RCT | USA | 12 | 1629 | PA-SES | PP-EES | 64.53±9.83 | 63.93±10.26 | 828(76.2) | 395(72.7) |
| PLATINUM [115] | Stone | 2011 | RCT | USA | 12 | 1530 | PP-EES | PP-EES | 64.1±10.3 | 63.1±10.3 | 550(71.6) | 542(71.1) |
| PLATINUM PLUS [116] | Fajadet | 2017 | RCT | France | 12 | 2980 | PP-EES | PP-EES | 65.7±10.5 | 66.1±10.7 | 1,515(77.7) | 806(78.4) |
| Pourmoghaddas et al [117] | Pourmoghaddas | 2017 | RCT | Iran | 3 | 83 | PP-EES | BMS | 60.58±9.061 | 60.80±9.923 | 23(53.5) | 20(50.0) |
| PRAGUE-18 [118] | Hlinomaz | 2021 | RCT | Czech Republic | 12 | 1111 | NA-DES | BMS | 61.7(42.9;78.1) | 62.7(46.7;81.5) | 574(76.6) | 223(75.3) |
| PRISON II [119] | Suttorp | 2006 | RCT | Netherlands | 6 | 200 | BMS | PP-SES | 59.3±10.2 | 59.6±10.6 | 76(76.0) | 83(83.0) |
| PRISON III [120] | Van den Branden | 2013 | RCT | Netherlands | 12 | 304 | PP-ZES | PP-SES | 61.5±11.1 | 62.1±10 | 125(83.3) | 124(80.5) |
| PRISION IV [121] | Teeuwen | 2017 | RCT | Netherlands | 12 | 330 | PP-EES | PA-SES | 62.4±10.5 | 62.8±9.5 | 122(73.9) | 137(83.0) |
| PRODIGY [122] | Valgimigli | 2013 | RCT | Multiple | 24 | 2003 | BMS | NA-ZES | 69±11 | 68±11 | 369(74) | 391(78) |
| PRODIGY | Valgimigli | 2013 | RCT | Multiple | 24 | 2003 | BMS | NA-PES | 69±11 | 68±11 | 369(74) | 395(78) |
| PRODIGY | Valgimigli | 2013 | RCT | Multiple | 24 | 2003 | BMS | NA-EES | 69±11 | 68±11 | 369(74) | 383(76) |
| PROMISE [123] | Kim | 2013 | RCT | Korea | 24 | 850 | PP-EES | PP-PES | 64±8 | 63±10 | 265(62.3) | 261(61.4) |
| PROSIT [124] | Lee | 2008 | RCT | Korea | 12 | 308 | NA-PES | NA-SES | 60±11 | 60±12 | 117(76.0) | 118(76.6) |
| PROTECT [125] | Camenzind | 2012 | RCT | Multiple | 36 | 8709 | PP-ZES | PP-SES | 62.3±10.6 | 62.1±10.7 | 3340(77) | 3308(76.0) |
| RAVEL [126] | Morice | 2002 | RCT | Multiple | 12 | 238 | BMS | PP-SES | 61.8±10.7 | 59.7±10.1 | 84(70) | 96(81) |
| REALITY [127] | Morice | 2006 | RCT | France | 12 | 1353 | PP-PES | PP-SES | 62.6±10.5 | 62.6±10.0 | 507(74.1) | 482(72.0) |
| RECOVERY [128] | Tao | 2021 | RCT | China | 24 | 432 | PA-SES | PF-SES | 58.3±9.6 | 59.3±8.4 | 147(68.1) | 137(63.4) |
| RECRE8 [129] | Rozemeijer | 2019 | RCT | Netherlands | 12 | 1491 | PF-AES | PP-ZES | 65.1±10.6 | 64.7±11.3 | 577(77.6) | 565(75.6) |
| REMEDEE [130] | Haude | 2013 | RCT | Multiple | 12 | 183 | PP-PES | NA-SES | 64.20±9.48 | 64.05±10.49 | 89(71.8) | 42(71.2) |
| RESERVOIR [131] | Romaguera | 2016 | RCT | Spain | 12 | 112 | PF-AES | PP-EES | 66.7±9.8 | 67.2±8.8 | 45(80.4) | 39(69.6) |
| RESET [132] | Kimura | 2012 | RCT | Japan | 12 | 3197 | PP-EES | PP-SES | 68.9±9.7 | 69.3±9.6 | 1238(78) | 1217(76) |
| RESOLUTE ALL COMERS [133] | Serruys | 2010 | RCT | Netherlands | 12 | 2292 | PP-ZES | PP-EES | 64.4±10.9 | 64.2±10.8 | 874(76.7) | 889(77.2) |
| RESOLUTE CHINA [134] | Xu | 2013 | RCT | Multiple | 12 | 400 | PP-ZES | PP-PES | 59.7±9.9 | 59.6±10.6 | 154(77.8) | 163(80.7) |
| Sakakibara et al [135] | Sakakibara | 2011 | RCT | Japan | 12 | 100 | PP-EES | PP-SES | 65±10 | 67±10 | 38(76.0) | 32(64.0) |
| SCANDSTENT [136] | Kelbæk | 2006 | RCT | Multiple | 7 | 322 | BMS | PP-SES | 62.9±9.2 | 62.5±9.4 | 121(74) | 126(79) |
| SCORPIUS [137] | Baumgart | 2007 | RCT | Germany | 12 | 200 | BMS | PP-SES | 66±9 | 66±10 | 62(66) | 70(62) |
| SEA-SIDE [138] | Burzotta | 2011 | RCT | Italy | 18 | 150 | NA-EES | PP-SES | 65±9 | 64±10 | 56(75) | 64(85) |
| SELECTION [139] | Chechi | 2007 | RCT | Italy | 7 | 80 | BMS | PP-PES | 59.7±8.9 | 61.7±8.7 | 34(85) | 32(80) |
| SENIOR [140] | Varenne | 2018 | RCT | Multiple | 12 | 1200 | PA-EES | BMS | 81.4±4.3 | 81.4±4.2 | 368(62) | 379(63) |
| Separham et al [141] | Separham | 2011 | RCT | Iran | 12 | 200 | PA-BES | PP-EES | 60.60±9.1 | 62.38±10.2 | 66(66) | 64(64) |
| SESAMI [142] | Menichelli | 2007 | RCT | Spain | 12 | 320 | BMS | PP-SES | 63(52-72) | 62(52-72) | 128(80) | 128(80) |
| SES-SMART [143] | Ardissino | 2004 | RCT | Italy | 8 | 257 | BMS | PP-SES | 63.2±11.5 | 63.7±10.9 | 99(76.7) | 85(66.4) |
| Shen et al [144] | Shen | 2015 | RCT | China | 24 | 212 | PA-AT | PP-SES | 57.9±9.3 | 59.3±9.5 | 73(69.5) | 75(70.1) |
| SIRIUS [145] | Moses | 2003 | RCT | USA | 9 | 1058 | BMS | PP-SES | 62.1±11.2 | 62.4±11.0 | 387(73) | 366(70) |
| SIRTAX [146] | Windecker | 2005 | RCT | Switzerland | 9 | 1012 | PP-PES | PP-SES | 62±11 | 62±12 | 382(75.9) | 399(78.4) |
| SORT OUT II [147] | Galløe | 2008 | RCT | Denmark | 18 | 2098 | PP-PES | PP-SES | 64.5±10.6 | 63.6±10.9 | 789(74.1) | 780(75.5) |
| SORT OUT III [148] | Rasmussen | 2010 | RCT | Denmark | 18 | 2332 | PP-ZES | PP-SES | 64.3±10.7 | 64.3±10.8 | 852(73) | 862(74) |
| SORT OUT IV [149] | Jensen | 2012 | RCT | Denmark | 24 | 2774 | PP-EES | PP-SES | 64.2±10.9 | 64.0±10.8 | 1055(75.9) | 1041(75.2) |
| SORT OUT V [150] | Christiansen | 2013 | RCT | Denmark | 12 | 2468 | PA-BES | PP-SES | 65.0±10.6 | 65.2±10.3 | 917(74.6) | 930(75.1) |
| SORT OUT VI [151] | Raungaard | 2015 | RCT | Denmark | 12 | 2999 | PP-ZES | PA-BES | 65.7±10.7 | 65.8±10.9 | 1144(76.2) | 1135(75.8) |
| SORT OUT VII [152] | Jensen | 2016 | RCT | Denmark | 12 | 1261 | PA-BES | PA-SES | 66.1±10.7 | 64.8±10.8 | 945(74.9) | 951(75.2) |
| SORT OUT VIII [153] | Maeng | 2019 | RCT | Denmark | 12 | 2764 | PA-EES | PA-BES | 66±11 | 66±11 | 1,060(77) | 1,056(77) |
| SORT OUT IX [154] | Jensen | 2020 | RCT | Denmark | 12 | 3151 | PF-BES | PA-SES | 66.4±10.7 | 66.1±11.1 | 1219(77.5) | 1221(77.3) |
| SORT OUT X [155] | Jakobsen | 2021 | RCT | Denmark | 12 | 3146 | PA-SES | PA-SES | 67.1±10.7 | 66.7±10.9 | 1213(76.9) | 1208(77.0) |
| SPIRIT FIRST [156] | Tsuchida | 2005 | RCT | Netherlands | 12 | 56 | PP-EES | BMS | 64±10 | 61±9 | 19(70) | 22(76) |
| SPIRIT II [157] | Claessen | 2009 | RCT | Netherlands | 24 | 300 | PP-EES | PP-PES | 62±10 | 62±9 | 158(71) | 60(79) |
| SPIRIT III [158] | Stone | 2008 | RCT | USA | 12 | 1002 | PP-EES | PP-PES | 63.2±10.5 | 62.8±10.2 | 469(70.1) | 218(65.7) |
| SPIRIT IV [159] | Stone | 2010 | RCT | USA | 12 | 3687 | PP-EES | PP-PES | 63.3±10.5 | 63.3±10.2 | 1665(67.7) | 833(67.8) |
| SPIRIT V DIABETIC [160] | Grube | 2012 | RCT | Multiple | 12 | 324 | PP-EES | PP-PES | 65±10 | 66±9 | 152(70) | 71(67) |
| STRATEGY [161] | Valgimigli | 2005 | RCT | Italy | 8 | 175 | BMS | PP-SES | 62(54-72) | 63(55-72) | 67(77) | 61(69) |
| TARGET ALL COMERS [162] | Lansky | 2018 | RCT | Multiple | 12 | 1653 | PP-EES | PA-SES | 65.3±10.5 | 64.9±9.8 | 634(76.4) | 641(78.1) |
| TARGET I [163] | Run-Lin Gao | 2013 | RCT | China | 12 | 458 | PP-EES | PA-SES | 58.7±9.4 | 59.6±9.4 | 157(69.2) | 158(68.4) |
| TALENT [164] | Zaman | 2019 | RCT | Multiple | 12 | 1435 | PP-EES | PA-SES | 65(58-72) | 66(58-72) | 547(76.5) | 546(75.8) |
| TAXI [165] | Jean-Jacques Goy | 2005 | RCT | Switzerland | 6 | 202 | PP-PES | PP-SES | 65±10 | 63±10 | 79(77.5) | 83(83) |
| TAXUS I [166] | Grube | 2002 | RCT | Germany | 12 | 61 | BMS | PP-PES | 63.8±7.8 | 66±6.8 | 25(83) | 29(94) |
| TAXUS II [167] | Colombo | 2003 | RCT | Multiple | 6 | 269 | BMS | PP-PES | 59.3±10.1 | 59.3±10.0 | 103(76) | 103(77) |
| TAXUS IV [168] | Greg W Stone | 2004 | RCT | Multiple | 9 | 1314 | BMS | PP-PES | 62.1±10.9 | 62.8±11.2 | 472(72.4) | 475(71.8) |
| TAXUS V [169] | Greg W Stone | 2005 | RCT | Multiple | 9 | 1172 | BMS | PP-PES | 62.8±10.8 | 62.9±11.2 | 398(68.7) | 405(70.2) |
| TAXUS VI [170] | Dawkins | 2005 | RCT | Multiple | 9 | 446 | BMS | PP-PES | 63.4±9.9 | 61.8±9.7 | 173(76.2) | 167(76.3) |
| TUXEDO [171] | Kaul | 2017 | RCT | India | 12 | 1830 | PP-EES | PP-PES | 58.34±9.12 | 58.40±9.21 | 696(76.0) | 681(74.5) |
| TIDE [172] | Pilgrim | 2011 | RCT | Switzerland | 12 | 302 | PF-TiNO | PP-ZES | 65.9±9.0 | 63.4±10.5 | 124(81.6) | 118(78.7) |
| TIDE-ACS [173] | Tonino | 2020 | RCT | Finland | 12 | 1491 | PF-TiNO | PP-EES | 62.7±10.9 | 62.6±10.5 | 745(75.3) | 383(76.3) |
| TWENTE [174] | Birgelen | 2012 | RCT | Netherland | 12 | 1391 | PP-ZES | PP-EES | 63.9±10.9 | 64.5±10.7 | 505(72.5) | 504(72.6) |
| TWENTE II [175] | Birgelen | 2014 | RCT | Netherland | 12 | 1811 | PP-ZES | PP-EES | 63.9±10.6 | 63.9±11.0 | 665(73) | 657(73) |
| TYPHOON [176] | Spaulding | 2006 | RCT | Multiple | 12 | 715 | BMS | PP-SES | 60.5±12.4 | 58.0±11.8 | 279(78.2) | 279(78.6) |
| Yin et al [177] | Yin | 2017 | RCT | China | 108 | 2407 | PP-DES | BMS | 56.6±10.6 | 57.7±11.7 | 1370(84.6) | 670(75.1) |
| Wessely et al [178] | Wessely | 2007 | RCT | Germany | 9 | 91 | PA-PES | PA-SES | 67.3±8.6 | 66.8±9.5 | 40(89) | 39(85) |
| X-MAN [179] | Dharma | 2014 | RCT | Indonesia | 1 | 150 | PP-EES | PF-CC | 56±9.6 | 54±9.5 | 67(89) | 61(81) |
| XAMI [180] | Hofma | 2012 | RCT | Netherland | 12 | 625 | PP-EES | PP-SES | 61.2±11.3 | 62.0±11.4 | 295(73.0) | 167(75.1) |
| ZEST [181] | Park | 2010 | RCT | Korea | 12 | 2645 | PP-ZES | PP-SES | 61.7±9.3 | 61.9±9.6 | 586(66.4) | 591(67.3) |
| ZEST | Park | 2010 | RCT | Korea | 12 | 2645 | PP-ZES | PP-PES | 61.7±9.3 | 62±9.6 | 586(66.4) | 582(65.8) |
| ZEST-AMI [182] | Lee | 2009 | RCT | Korea | 12 | 328 | PP-ZES | PP-SES | 61.9±11.0 | 57.8±11.3 | 84(77.8) | 95(86.4) |
| ZEST-AMI | Lee | 2009 | RCT | Korea | 12 | 328 | PP-ZES | PP-PES | 61.9±11.0 | 59.3±11.2 | 84(77.8) | 91(82.7) |
| ZEUS [183] | Valgimigli | 2016 | RCT | Multiple | 12 | 1606 | BMS | PP-ZES | 71.8±12 | 71.8±11 | 572(71.1) | 561(70) |
| Zhang Q et al [184] | Zhang Qi | 2006 | RCT | China | 12 | 673 | PP-PES | PP-SES | 64±12 | 64±10 | 136(67) | 172(70) |
| Zhang L et al [185] | Zhang | 2012 | RCT | China | 12 | 180 | PA-R | PF-PES | 66.96±6.51 | 63.95±10.34 | 58(68.24) | 49(59.76) |
| Zhang Y et al [186] | Zhang | 2013 | RCT | China | 24 | 989 | PF-PES | PA-SES | 65.24±10.46 | 67.50±9.79 | 214(65.44) | 236(69.21) |
| Zhang Y et al | Zhang | 2013 | RCT | China | 24 | 989 | PF-PES | PP-SES | 65.24±10.46 | 65.87±11.14 | 214(65.44) | 220(68.54) |
| ZOMAXX I [187] | Chevalier | 2008 | RCT | Multiple | 9 | 396 | PP-ZES | PP-PES | 63±10 | 63±11 | 149(75) | 152(77) |
| ZOMAXX II [188] | Gray | 2012 | RCT | USA | 9 | 1099 | PP-ZES | Paclitaxel | 63±10 | 63±11 | 384(69) | 374(69) |
| XIMA [189] | Belder | 2014 | RCT | Multiple | 12 | 800 | PP-EES | BMS | 83.6±3.2 | 83.4±3.1 | 224(61.1) | 237(59.1) |
| Xu et al [190] | Xu | 2011 | RCT | China | 24 | 324 | PP-ZES | PA-SES | 60.4±10.5 | 57.0±11.4 | 111(71.2) | 118(70.2) |
| TINOX [191] | Windecker | 2005 | RCT | Multiple | 6 | 92 | PF-TiNO | BMS | 65±10 | 64±13 | 34(76) | 32(68) |
| TITAX AMI [192] | Karjalainen | 2009 | RCT | Finland | 12 | 425 | PF-TiNO | PP-PES | 64±11 | 64±11 | 162(76) | 157(74) |
| BASE ACS [193] | Karjalainen | 2012 | RCT | Finland | 12 | 827 | PF-TiNO | PP-EES | 63±12 | 63±12 | 317(76) | 312(76.1) |
| TITANIC XV [194] | López-Mínguez | 2014 | RCT | Spain | 12 | 173 | PF-TiNO | PP-EES | 66.5±8.8 | 64.5±10.1 | 60 (72.3) | 68 (75.6) |
| CIBELES [195] | Moreno | 2013 | RCT | Multiple | 12 | 207 | PP-EES | PP-SES | 65±10 | 63±11 | 85(80.2) | 85(84.1) |
| PIONEER [196] | Birgelen | 2018 | RCT | Multiple | 12 | 170 | PP-ZES | PA-SES | 62.5±9.3 | 64.3±8.9 | 64(73.6) | 62(74.7) |

**Supplemental Table 3: Detailed trial-level baseline comorbidities of the included population in intervention (i) and control (c) groups.**

| STUDY | Stent Comparison | | DM, n(%) | | HTN, n(%) | | HLD, n(%) | | Current Smoker ,n(%) | | Previous PCI,n(%) | | Previous MI,n(%) | | Previous CABG,n(%) | |
| --- | --- | --- | --- | --- | --- | --- | --- | --- | --- | --- | --- | --- | --- | --- | --- | --- |
|  | i | c | i | c | i | c | i | c | i | c | i | c | i | c | i | c |
| ACTION [1] | PF-DES | BMS | 13(10.9) | 5(4.2) | 47(39.3) | 50(42.3) | 56(46.9) | 53(44.9) | 26(21.8) | 30(25.4) |  |  | 37.5(31.4) | 41(34.7) |  |  |
| APPENDIX-AMI [2] | PP-EES | PP-SES | 50(10.4) | 70(15.2) | 208(42.8) | 238(51.0) | 249(53.7) | 251(54.9) | 133(27.4) | 107(23.2) | 83(16.8) | 108(22.5) | 109(22.1) | 108(22.7) | 50(10.1) | 72(15.0) |
| BASKET [3] | PP-SES | BMS | 41(16) | 60(21) | 169(65) | 190(68) | 196(74) | 211(75) | 76(29) | 86(31) | 44(17) | 42(15) | 73(28) | 75(27) | 37(14) | 35(12) |
| BASKET | PP-PES | BMS | 52(19) | 60(21) | 185(66) | 190(68) | 213(76) | 211(75) | 73(26) | 86(31) | 47(17) | 42(15) | 78(28) | 75(27) | 33(12) | 35(12) |
| BASKET-PROVE [4] | PP-SES | BMS | 136(18) | 108(14) | 483(62) | 485(63) | 475(61) | 495(65) | 235(30) | 261(34) | 97(13) | 88(12) | 96(12) | 103(13) | 27(3) | 20(3) |
| BASKET-PROVE | PP-EES | BMS | 119(15) | 108(14) | 469(61) | 485(63) | 498(64) | 495(65) | 267(34) | 261(34) | 93(12) | 88(12) | 82(11) | 103(13) | 20(3) | 20(3) |
| BASKET-PROVE II [5] | PA-BES | BMS | 161(21) | 141(19) | 506(66) | 510(67) | 494(65) | 471(62) | 271(35) | 280(37) | 98(13) | 115(15) | 71(9) | 75(10) | 23(3) | 14(2) |
| BASKET-PROVE II | PP-EES | BMS | 127(17) | 141(19) | 507(66) | 510(67) | 484(63) | 471(62) | 267(35) | 280(37) | 94(12) | 115(15) | 65(9) | 75(10) | 22(3) | 14(2) |
| BASKET-SMALL [6] | PP-ZES | PP-PES | 21(23.1) | 20(20) | 63(69.2) | 76(76) | 52(57.1) | 60(60) | 32(35.2) | 31(31) | 23(25.3) | 20(20) | 19(20.9) | 15(15) | 3(3.3) | 9(9) |
| BIODEGRADE [7] | PA-BES | PA-SES | 393(33.9) | 384(32.9) | 706(60.9) | 685(58.7) | 625(53.9) | 609(52.2) | 306(26.4) | 324(27.8) | 147(12.7) | 135(11.6) | 56(4.8) | 60(5.1) | 10(0.9) | 8(0.7) |
| BIOFLOW II [8] | PA-SES | PP-EES | 84(28.2) | 44(28.6) | 231(77.5) | 119(77.3) | 202(67.8) | 113(73.4) | 87(29.2) | 37(24.0) |  |  | 90(30.2) | 31(20.1) |  |  |
| BIOFLOW IV [9] | PA-SES | PP-EES | 117(30.4) | 59(31.1) | 296(76.9) | 136(71.6) | 261(67.8) | 136(71.6) | 82(21.3) | 53(27.9) | 169(43.9) | 88(46.3) | 114(29.6) | 62(32.6) |  |  |
| BIOFLOW V [10] | PA-SES | PP-EES | 300(34) | 166(37) | 696(80) | 354(80) | 695(79) | 370(82) | 209(24) | 102(23) | 323(37) | 147(33) | 238(27) | 115(26) | 62(7) | 23(5) |
| BIOFLOW VI [11] | PA-SES | PP-EES | 60(27.3) | 58(26.4) | 120(54.5) | 125(56.8) | 84(38.2) | 93(42.3) | 75(34.1) | 81(36.8) | 0(0) | 0(0) | 31(14.1) | 17(7.7) |  |  |
| BIOFREEDOM FIM [12] | PF-BES | PP-PES | 17.5(28.7) | 15(25) | 52(85.3) | 51(85) | 43(71.1) | 45(75) | 11(18.6) | 7(12.3) | 23(38) | 27(45.8) | 12.5(20.7) | 11(18.3) |  |  |
| BIOSTEMI [13] | PA-SES | PP-EES | 73(11) | 82(13) | 281(43) | 297(46) | 304(47) | 302(47) | 294(45) | 250(39) | 29(4) | 34(5) | 27(4) | 24(4) | 2(0.3) | 8(1) |
| BIONICS [14] | PP-RES | PP-ZES | 314(32.8) | 310(32.3) | 687(72.4) | 704(74) | 759(80.4) | 744(78.1) | 224(23.4) | 186(19.4) | 372(38.8) | 367(38.2) | 298(31.1) | 293(30.5) | 84(8.8) | 92(9.6) |
| BIONYX [15] | PP-ZES | PA-SES | 260(20.9) | 250(20.1) | 611(49.8) | 651(53.2) | 552(45.4) | 562(46.4) | 371(30.6) | 370(30.7) | 262(21.1) | 278(22.3) | 194(15.6) | 206(16.5) | 79(6.4) | 97(7.8) |
| BIO-RESORT [16] | PP-EES | PA-ZES | 203(17) | 210(18) | 520(44) | 554(47) | 422(36) | 450(38) | 336(30) | 354(31) | 214(18) | 198(17) | 192(16) | 248(21) | 91(8) | 96(8) |
| BIO-RESORT | PA-SES | PA-ZES | 211(18) | 210(18) | 550(47) | 554(47) | 463(40) | 450(38) | 341(30) | 354(31) | 214(18) | 198(17) | 209(18) | 248(21) | 80(7) | 96(8) |
| BIOSCIENCE [17] | PA-SES | PP-EES | 257(24.2) | 229(21.7) | 728(68.5) | 706(66.9) | 712(67) | 716(67.8) | 309(29.1) | 300(28.5) | 325(30.6) | 292(27.7) | 223(21) | 204(19.3) | 113(10.6) | 98(9.3) |
| CATOS [18] | PP-ZES | PP-SES | 28(35) | 23(29) | 52(65) | 50(63) | 21(26) | 15(19) | 28(35) | 30(38) | 11(14) | 15(19) | 9(11) | 8(10) |  |  |
| CERVINKA ET AL [19] | PP-SES | PP-PES | 15(40) | 10(30) | 21(56) | 17(50) | 26(70) | 21(63) | 23(63) | 20(60) |  |  | 15(41) | 11(33) |  |  |
| CENTURY II [20] | PA-SES | PP-EES | 176(31.9) | 170(30.9) | 404(73.3) | 373(67.8) | 387(70.3) | 383(69.6) | 122(22.2) | 131(23.9) | 205(37.2) | 193(35.0) | 156(28.3) | 152(27.6) | 25(4.5) | 20(3.7) |
| CHOICE [21] | PP-EES | PA-BES | 214(33.5) | 210(33.1) | 385(60.3) | 358(56.6) | 137(21.5) | 140(22.1) | 163(25.8) | 180(28.4) | 68(10.7) | 63(9.9) | 31(4.9) | 30(4.8) | 3(0.5) | 5(0.8) |
| CHOICE | PA-BES | PP-ZES | 210(33.1) | 215(33.6) | 358(56.6) | 379(59.3) | 140(22.1) | 139(21.8) | 180(28.4) | 188(29.5) | 63(9.9) | 64(10) | 30(4.8) | 26(4.1) | 5(0.8) | 1(0.2) |
| CORACTO [22] | BMS | PA-SES | 10(22.2) | 10(21.7) |  |  |  |  |  |  |  |  |  |  |  |  |
| COMFORTABLE AMI [23] | PA-BES | BMS | 84(14.6) | 90(15.5) | 279(48.5) | 265(45.5) | 324(56.6) | 328(56.7) | 272(47.9) | 301(52.3) | 19(3.3) | 27(4.6) | 31(5.4) | 32(5.5) | 10(1.7) | 4(0.7) |
| COMPARE [24] | PP-EES | PP-PES | 153(17) | 172(19) | 417(46) | 447(50) | 477(53) | 451(50) | 295(33) | 262(29) | 117(13) | 123(14) | 136(15) | 159(18) | 60(7) | 53(6) |
| COMPARE II [25] | PA-BES | PP-EES | 391(21.8) | 197(21.6) | 983(54.8) | 513(56.3) |  |  | 553(30.8) | 250(27.4) | 320(17.8) | 155(17) | 362(20.3) | 170(18.8) | 105(5.9) | 52(5.7) |
| COSTAR II [26] | PA-PES | PP-PES | 274(27.7) | 146(21.3) | 770(77.9) | 533(686) | 796(80.5) | 541(78.9) | 199(20.1) | 150(21.9) | 331(33.5) | 224(32.7) | 260(26.3) | 190(27.7) | 63(6.4) | 41(6) |
| CREST MI [27] | PP-ZES | PP-SES | 75(16.7) | 83(19.4) | 216(48) | 220(51.6) | 82(18.2) | 74(17.3) | 264(58.8) | 235(55.1) |  |  | 11(2.5) | 9(2) |  |  |
| CRE8 (NEXT) [28] | PF-AES | PP-PES | 48(29.7) | 39(24.2) | 104(64.2) | 104(64.6) | 102(63) | 98(60.9) | 39(24.1) | 40(24.8) | 26(16) | 23(14.3) | 14(8.6) | 15(9.3) |  |  |
| C-SIRIUS [29] | PP-SES | BMS | 12(24) | 12(24) | 28(56) | 24(48) | 42(84) | 43(86) | 18(36) | 19(38) | 4(8) | 4(8) | 24(48) | 21(42) | 3(6) | 1(2) |
| Dang et al [30] | PP-SES | PF-PES | 15(27.3) | 12(24) | 23(41.8) | 23(46) | 13(23.6) | 10(20) | 34(61.8) | 33(66) | 1(1.8) | 1(2) | 4(7.3) | 2(4) |  |  |
| DEBATER [31] | PP-SES | BMS | 47(11) | 41(9) | 124(29) | 130(29) | 119(28) | 124(28) | 286(67) | 64(14) |  |  |  |  |  |  |
| DEBFIRST [32] | PF-PES+BMS | PP-ZES | 28(31.1) | 26(28.9) | 25(27.8) | 40(44.4) | 15(16.7) | 18(20) | 27(30) | 19(21.1) | 5(5.6) | 9(10) | 3(3.3) | 4(4.4) |  |  |
| DECODE [33] | NA-SES | BMS | 48(88.9) | 28(96.6) | 36(66.7) | 22(75.9) | 40(76.9) | 20(71.4) | 19(35.2) | 12(41.4) |  |  | 20(37) | 9(31) | 3(5.6) | 2(6.9) |
| DEDICATION [34] | NA-DES | BMS | 29.1(9.3) | 36(11.5) | 101(32.3) | 106(33.9) | 58(18.5) | 67(21.4) | 165(52.7) | 171(54.7) | 14(4.4) | 17(5.4) | 19(6.1) | 22(7) |  |  |
| DES-DIABETES [35] | NA-SES | NA-PES | 182(91) | 181(90.5) | 114(57) | 124(62) | 55(27.5) | 63(31.5) | 54(27) | 57(28.5) | 25(12.5) | 25(12.5) |  |  | 4(2) | 3(1.5) |
| DESSERT [36] | PP-SES | BMS | 73(96) | 64(86) | 58(77) | 56(75) | 35(47) | 39(52) | 32(43) | 29(39) | 11(15) | 8(11) | 27(36) | 19(25) | 5(7) | 3(4) |
| DESSOLVE II [37] | PA-SES | PP-ZES | 23(19) | 12(19.7) | 87(70.5) | 42(68.9) | 89(72.7) | 50(81.7) | 26(21.5) | 15(25.4) | 38(30.9) | 14(22.9) | 28(23.1) | 10(16.4) | 5(4.1) | 2(3.3) |
| DESSOLVE III [38] | PA-SES | PP-EES | 186(26) | 187(27) | 496(72) | 517(75) | 408(61) | 393(60) | 171(27) | 168(26) | 236(34) | 247(36) | 190(27) | 192(28) | 51(7) | 66(694) |
| DIABEDES [39] | PP-SES | PP-PES | 63(83) | 67(87) | 48(63) | 58(75) | 65(86) | 70(91) | 29(38) | 18(23) | 10(13) | 19(25) |  |  | 4(5) | 6(8) |
| DIABETES [40] | PP-SES | BMS | 80(100) | 80(100) | 53(66.3) | 53(66.3) | 49(61.3) | 49(61.3) | 36(45) | 40(50) | 16(20) | 14(17.5) | 25(31.3) | 34(42.5) | 16(20) | 14(17.6) |
| Diaz de la llera et al [41] | BMS | PP-SES | 17(28.3) | 16(26.7) |  |  |  |  | 41(68.3) | 41(68.3) | 4(6.7) | 3(5) | 6(10) | 3(5) | 1(1.7) | 0(0) |
| DIRECT II [42] | PA-SES | PP-ZES | 18(16.8) | 11(21.6) | 56(52.3) | 25(49) | 22(20.6) | 6(11.8) | 61(57) | 16(31.4) | 24(22.4) | 9(17.6) | 19(17.8) | 11(21.6) |  |  |
| ENDEAVOR II [43] | PP-ZES | BMS | 107(18) | 132(22) |  |  | 484(81) | 461(77) | 209(35) | 209(35) | 131(22) | 108(18) | 239(40) | 259(42) |  |  |
| ENDEAVOR III [44] | PP-ZES | PP-SES | 96(29.7 | 32(28.3) | 227(70.7 | 84(74.3) | 268(83.5) | 98(86.7) | 212(66.5) | 85(75.2) | 73(22.6) | 19(16.8) | 64(19.9) | 23(20.7) | 17(5.3) | 9(8.0) |
| ENDEAVOR IV [45] | PP-ZES | PP-PES | 241(31.2) | 236(30.5) | 614(79.4) | 640(82.6) | 629(81.4) | 657(84.8) | 479(62.6) | 462(60.4) | 218(28.2) | 229(29.5) | 161(21.1) | 176(23.2) | 76(9.8) | 65(8.4) |
| Erglis et al [46] | BMS | NA-PES | 6(11) | 6(12) | 31(59) | 25(50) | 43(81) | 34(68) | 14(26) | 11(22) | 19(36) | 21(42) | 30(57) | 20(40) | 2(4) | 4(8) |
| ESSENCE-DIABETES [47] | PP-EES | PP-SES | 149(100) | 151(100) | 102(68.5) | 110(72.8) | 62(41.6) | 53(35.1) | 31(20.8) | 41(27.2) | 11(7.4) | 6(4.0) | 2(1.3) | 3(2.0) | 1(0.7) | 1(0.7) |
| EUCATAX [48] | PA-PES | BMS | 49(23.2) | 34(16.1) | 135(64.0) | 140(66.4) | 120(56.9) | 108(51.2) | 45(21.3) | 50(23.7) | 75(35.5) | 51(24.2) |  |  |  |  |
| EVERBIO II [49] | PA-EES | PA-BES | 13(16) | 26(33) | 51(64) | 50(63) | 50(63) | 52(65) | 30(38) | 25(31) | 25(31) | 23(29) | 14(18) | 16(20) | 11(14) | 16(20) |
| EVOLVE [50] | PP-EES | PA-EES | 22(22.4) | 16(17.2) | 68(69.4) | 58(61.3) | 69(70.4) | 64(68.5) | 27(27.8) | 20(21.7) | 32(32.7) | 31(33.3) | 34(34.4) | 30(32.3) | 1(1) | 2(2.2) |
| EVOLVE II [51] | PA-EES | PP-EES | 267(31.6) | 258(30.8) | 654(77.30 | 629(75.1) | 626(74) | 624(74.5) | 184(21.8) | 188(22.4) | 303(35.8) | 313(37.3) |  |  | 39(4.6) | 51(6.1) |
| EXAMINATION [52] | PP-EES | BMS | 137(18) | 121(16) | 347(46) | 378(51) | 354(47) | 301(40) |  |  | 29(4) | 32(4) | 33(4) | 47(6) | 3(0.4) | 7(1) |
| EXCELLA II [53] | PP-NES | PP-ZES | 33(23.7) | 17(23.9) | 106(76.3) | 51(71.8) | 118(85.5) | 54(76.1) | 22(15.9) | 16(22.5) | 47(33.8) | 25(35.2) | 37(26.6) | 22(31) | 5(3.6) | 4(5.6) |
| EXCELLENT [54] | PP-EES | PP-SES | 402(37.3) | 148(40.7) | 791(73.3) | 266(73.1) | 823(76.3) | 270(74.2) | 278(25.8) | 106(29.1) | 99(9.2) | 30(8.2) | 56(5.2) | 18(4.9) | 12(1.1) | 6(1.6) |
| E-SIRIUS [55] | PP-SES | BMS | 33(19) | 48(27) | 109(63) | 114(64) | 132(77) | 124(71) | 63(36) | 53(30) | 34(19) | 39(22) | 71(41) | 76(43) | 10(6) | 11(6) |
| FIBISTEMI [56] | NA-SES | BMS | 20(19.8) | 9(16.4) | 53(52.5) | 33(60.0) | 27(26.7) | 16(29.1) | 59(58.4) | 29(52.7) |  |  |  |  |  |  |
| GARA-GARA [57] | PP-SES | PP-PES | 178(40) | 193(41) | 334(74) | 348(74) | 271(60) | 295(63) | 167(37) | 177(38) |  |  | 137(30) | 146(31) | 15(3.3) | 17(3.6) |
| GENESIS [58] | PA-PES | NA-Pim | 18(36.7) | 32(32) | 36(73.5) | 66(66) | 36(73.5) | 82(82) | 8(16.3) | 20(20) | 13(26.5) | 33(33) | 11(22.5) | 26(26) | 3(6.1) | 2(2) |
| GENESIS | PA-PES | NA-DES | 18(36.7) | 17(17.5) | 36(73.5) | 65(67) | 36(73.5) | 69(71.1) | 8(16.3) | 35(36) | 13(26.5) | 28(28.9) | 11(22.5) | 29(29.9) | 3(6.1) | 0(0) |
| GRACIA-3 [59] | PP-PES | BMS | 44(20.27) | 36(16.6) | 95(43.7) | 93(43.0) | 97(44.7) | 89(41.2) | 99(45.6) | 111(51.3) | 4(1.8) | 9(4.1) | 13(5.9) | 12(5.5) |  |  |
| GISSOC II-GISE [60] | PP-SES | BMS | 19(25.7) | 15(19.2) | 49(66.2) | 51(65.4) | 54(73) | 59(75.6) | 45(60.8) | 41(52.6) | 11(14.9) | 15(19.2) | 24(32.4) | 24(30.8) | 5(6.7) | 4(5.1) |
| HARMONEE [61] | PA-SES | PP-EES | 117(40.7) | 93(32.6) | 218(76.0) | 220(77.2) | 225(78.4) | 227(79.6) | 191(67.7) | 175(62.5) | 72(25.1) | 83(29.1) | 45(15.7) | 45(15.8) | 4(1.4) | 5(1.8) |
| Herdeg C et al. [62] | PP-PES | BMS | 26(40) | 23(33) | 60(90) | 59(87) | 52(78) | 48(71) | 11(16) | 5(7) | 24(36) | 34(50) | 16(24) | 25(37) | 9(13) | 7(10) |
| Hong et al [63] | PP-SES | PP-PES | 85(100) | 84(100) | 56(65.9) | 65(77.4) | 29(34.1) | 33(39.3) | 9(10.6) | 14(16.7) | 11(12.9) | 9(10.7) | 13(15.3) | 12(14.3) | 0(0.0) | 3(3.6) |
| HORIZONS-AMI [64] | PP-PES | BMS | 364(16.1) | 114(15.2) | 1155(51.2) | 389(51.9) | 953(42.2) | 308(41.1) | 1041(46) | 388(51.9) | 214(9.5) | 58(7.7) | 206(9.1) | 82(10.9) | 50(2.2) | 14(1.9) |
| HOST-ASSURE [65] | NA-EES | NA-ZES | 795(31.8) | 401(32) | 1706(68.2) | 852(68.1) | 1601(64) | 822(65.7) | 823(32.9) | 369(29.5) | 247(9.9) | 120(9.6) | 116(4.6) | 49(3.9) | 16(0.6) | 10(0.8) |
| INSPIRON I [66] | NA-SES | BMS | 10(26.3) | 8(42.1) | 32(84.2) | 17(89.5) | 30(79.0) | 14(73.7) | 15(39.5) | 6(31.6) | 7(18.4) | 2(10.5) | 7(18.4) | 3(15.8) | 2(5.3) | 0 |
| ISAR-DIABETES [67] | PP-SES | PP-PES | 125(100) | 125(100) | 70(56.0) | 82(65.6) | 73(58.4) | 78(62.4) | 16(12.8) | 16(12.8) |  |  | 39(31.2) | 49(39.2) | 16(12.8) | 13(10.4) |
| ISAR-LEFT-MAIN [68] | PP-SES | PP-PES | 86(28) | 90(30) | 209(69) | 210(70) | 229(75) | 237(78) | 30(10) | 31(10) | 153(50) | 139(46) | 84(28) | 77(25) |  |  |
| ISAR-LEFT-MAIN II [69] | PP-ZES | PP-EES | 92(28.4) | 93(28.5) | 221(68.2) | 228(69.9) | 223(68.8) | 257(75.8) | 48(14.8) | 43(13.2) | 168(51.9) | 175(53.7) | 103(31.8) | 94(28.8) |  |  |
| ISAR-SMART III [70] | PP-PES | PP-SES |  |  | 120(67) | 116(64) | 99(55) | 100(56) | 22(12) | 27(15) |  |  | 56(31) | 53(29) | 29(16) | 20(11) |
| ISAR-TEST [71] | PF-SES | PP-PES | 73(32) | 58(26) | 142(63) | 155(69) | 165(73) | 170(76) | 43(19) | 39(17) |  |  | 72(32) | 71(32) | 25(11) | 25(11) |
| ISAR-TEST-2 [72] | PF-RP | PP-SES | 96(28.8) | 91(27.2) | 229(64.9) | 214(63.4) | 209(62.8) | 231(69.0) | 66(19.8) | 58(17.3) |  |  | 84(25.2) | 100(29.9) | 33(9.9) | 27(8.1) |
| ISAR-TEST-2 | PP-ZES | PP-SES | 89(26.3) | 91(27.2) | 229(67.6) | 214(63.4) | 222(65.5) | 231(69.0) | 61(18.0) | 58(17.3) |  |  | 88(26.0) | 100(29.9) | 29(8.6) | 27(8.1) |
| ISAR-TEST-4 [73] | PA-SES | PP-DES | 376(29.0) | 377(28.9) | 897(69.1) | 881(67.6) | 868(66.8) | 846(64.9) | 202(15.6) | 215(16.5) |  |  | 372(28.6) | 373(28.6) | 129(9.9) | 129(9.9) |
| ISAR-TEST-5 [74] | PF-SES+P | PP-ZES | 575(28.7) | 295(29.5) | 1336(66) | 666(66.6) | 1257(62) | 650(65.0) | 357(17.8) | 166(16.6) |  |  | 586(29.3) | 299(29.9) | 188(9.4) | 96(9.6) |
| I-LOVE-IT 2 [75] | PA-SES | PP-SES | 414(22.6) | 193(21.3) | 1150(62.9) | 559(61.6) | 445(24.3) | 204(22.5) | 685(37.5) | 335(36.9) | 137(7.5) | 64(7.1) | 301(16.5) | 151(16.6) | 8(0.4) | 6(0.7) |
| J-DESSERT [76] | NA-SES | NA-PES | 801(48.1) | 823(48.9) | 1272(76.4) | 1341(79) | 1051(63) | 1082(64.3) | 305(18.3) | 333(19.8) | 375(22.5) | 383(22.7) | 239(14.4) | 231(13.7) | 50(3) | 52(3.1) |
| Juwana et al [77] | PP-SES | PP-PES | 21(10.7) | 13(6.5) | 53(27) | 66(33) | 37(19) | 38(19) | 98(50) | 110(55) | 13(6.6) | 12(6) | 12(6.1) | 12(6) | 2(1) | 3(1,5) |
| Kamoi et al [78] | PP-SES | PP-PES | 24(48) | 27(54) | 32(64) | 33(66) | 6(12) | 3(6) |  |  |  |  |  |  |  |  |
| Kim et al [79] | PP-SES | PP-PES | 85(100) | 84(100) | 54(63.5) | 61(72.6) | 26(30.6) | 26(31) | 17(20) | 22(26,2) | 11(12.9) | 9(10.7) | 13(15.3) | 12(14,3) | 0(0) | 3(3.6) |
| KOMER [80] | NA-ZES | NA-SES | 41(20) | 47(23.0) | 79(38.5) | 86(42.2) | 63(30.7) | 70(31.3) | 104(50.7) | 117(57.4) | 8(3.9) | 6(2.9) | 5(2.4) | 4(2.0) | 0(0) | 0(0) |
| KOMER | NA-ZES | NA-PES | 41(20) | 39(19.3) | 79(38.5) | 90(44.6) | 63(30.7) | 68(33.7) | 104(50.7) | 114(56.4) | 8(3.9) | 3(1.5) | 5(2.4) | 3(1.5) | 0(0) | 0(0) |
| Laarman et al [81] | NA-PES | BMS | 31(10) | 37(12) | 95(30.6) | 98(31.7) | 72(23.2) | 86(27.8) | 165(53.2) | 154(49.80 | 14(4.5) | 13(4.2) | 14(4.5) | 18(5.8) | 2(0.6) | 2(0.6) |
| Lansky et al [82] | BMS | BMS | 88(23.2) | 84(22.4) | 221(58.2) | 225(60) | 184(48.4) | 166(44.3) |  |  |  |  |  |  |  |  |
| LEADERS [83] | PA-BES | PP-SES | 223(26.0) | 191(22.5) | 630(73.5) | 618(72.7) | 560(65.3) | 580(68.2) | 206(24.0) | 214(25.2) | 312(36.4) | 312(36.7) | 276(32.2) | 277(32.6) | 90(10.5) | 107(12.6) |
| LEADERS FREE [84] | PF-BES | BMS | 414(34) | 391(32.3) | 952(78.1) | 961(79.6) | 742(62) | 746(62.7) |  |  | 270(22.2) | 265(21.9) | 237(19.6) | 258(21.4) | 115(9.4) | 122(10.1) |
| Li et al [85] | PA-SES | PP-SES | 43(26.2) | 55(32.7) | 95(57.9) | 87(51.8) | 76(46.3) | 55(32.7) | 108(65.9) | 102(60.7) | 5(3.0) | 4(2.4) | 9(5.5) | 7(4.2) | 2(1.2) | 0(0) |
| LIPSIA YUKON [86] | PF-SES | PP-PES | 120(100) | 116(100) | 118(98) | 112(97) |  |  | 28(23) | 31(27) | 38(32) | 33(28) | 26(22) | 26(22) | 6(5) | 12(10) |
| LISAII [87] | PP-PES | PF-PES | 22(27.5) | 31(36.9) | 59(73.8) | 61(72.6) | 50(62.5) | 51(60.7) | 14(17.5) | 17(20.2) | 24(30.4) | 23(27.4) | 27(33.8) | 24(28.6) | 5(6.3) | 5(6) |
| LONG-DES II [88] | NA-SES | NA-PES | 82(32.8) | 84(33.6) | 138(55.2) | 137(54.8) |  |  | 93(37.2) | 94(37.6) | 21(8.4) | 29(11.6) |  |  | 8(3.2) | 6(2.4) |
| LONG-DES III [89] | NA-EES | NA-SES | 71(31.7) | 62(27.4) | 137(61.2) | 128(56.6) | 127(56.7) | 128(56.6) | 52(23.2) | 48(21.2) | 15(6.7) | 19(8.4) | 10(4.5) | 7(3.1) | 15(6.7) | 19(8.4) |
| LONG-DES IV [90] | NA-ZES | NA-SES | 68(27.2) | 76(30.4) | 150(60) | 135(54) | 141(56.4) | 136(54.4) | 68(27.2) | 71(28.4) | 17(6.8) | 14(5.6) | 3(1.2) | 5(2) | 4(1.6) | 4(1.6) |
| LONG-DES V [91] | PA-BES | PP-EES | 79(32.2) | 89(34.9) | 161(65.7) | 154(60.4) | 131(53.5) | 145(56.9) | 63(25.7) | 74(29) | 16(6.5) | 26(10.2) | 6(2.4) | 11(4.3) | 1(0.4) | 0(0) |
| MASTER [92] | BMS | NA-D/BMS | 26(12) | 39(18.1) | 91(42.3) | 100(47.4) | 58(27.4) | 57(27.1) | 120(55.3) | 101(46.8) | 8(3.7) | 12(5.6) | 8(3.7) | 19(8.8) |  |  |
| MASTER STUDY [93] | BMS | PA-SES | 58(15.5) | 16(12.8) | 201(53.6) | 64(51.2) | 146(39) | 45(36) | 190(50.7) | 60(48) | 5(1.3) | 4(3.2) | 13(3.5) | 4(3.2) | 1(0.3) | 0(0) |
| MERIT-V [94] | PA-SES | PP-EES | 41(24.1) | 18(20.9) | 125(73.5) | 68(79) | 118(69.4) | 59(68.6) | 71(41.7) | 41(47.6) | 31(18.2) | 14(16.2) | 37(21.7) | 13(15.1) |  |  |
| MISSION [95] | PP-SES | BMS | 20(12.7) | 10(6.6) | 48(30.4) | 39(25.7) | 37(23.4) | 25(16.4) | 84(53.2) | 85(55.9) | 4(2.5) | 1(0.7) | 7(4.4) | 5(3.3) | 1(0.6) | 1(0.7) |
| MULTISTRATEGY [96] | NA-SES | BMS | 53(14.2) | 55(14.7) | 206(55.3) | 220(59.1) | 192(51.7) | 204(54.8) | 137(36.8) | 140(37.6) | 23(6.2) | 17(4.5) | 27(7.2) | 30(8) | 3(0.8) | 5(1.3) |
| NAPLES-DIABETES [97] | NA-SES | NA-PES | 76(100) | 75(100) | 55(72.5) | 57(76) | 47(61.8) | 48(64) | 13(17) | 16(21) |  |  | 33(43.4) | 26(34.7) |  |  |
| NAPLES-DIABETES | NA-SES | NA-EES | 76(100) | 75(100) | 55(72.5) | 56(74.5) | 47(61.8) | 45(60) | 13(17) | 13(17) |  |  | 33(43.4) | 35(46.7) |  |  |
| NEVO RES-I [98] | PA-SES | PP-PES | 36(18) | 39(20) |  |  | 151(75) | 144(75) | 55(27) | 43(22) | 68(34) | 48(25) | 65(32) | 50(26) |  |  |
| NEXT [99] | PA-BES | PP-EES | 745(46) | 740(46) | 1317(81) | 1323(82) | 1265(78) | 1263(78) | 301(19) | 293(18) | 816(50) | 820(51) | 460(28) | 454(28) | 83(5.3) | 77(4.8) |
| NOBORI I [100] | PA-BES | PP-PES | 40(16.8) | 34(27.2) | 156(65.6) | 85(68) | 166(69.8) | 93(74.4) | 141(59.5) | 66(52.8) | 47(19.8) | 22(17.6) | 56(23.5) | 35(28) | 9(3.8) | 4(3.2) |
| NOBORI JAPAN [101] | PA-BES | PP-SES | 75(38.7) | 52(39.4) | 149(76.8) | 111(84.1) | 150(77.3) | 108(81.8) | 50(25.8) | 24(18.2) | 63(32.5) | 51(38.6) | 40(20.6) | 28(21.2) | 1(0.5) | 4(3) |
| NORSTENT [102] | NA-EES/ZES | BMS | 575(12.8) | 548(12.2) | 1934(42.9) | 1857(41.2) | 2413(53.6) | 2455(54.4) | 1538(34.1) | 1609(35.7) |  |  | 433(9.6) | 479(10.6) | 293(6.5) | 300(6.7) |
| OCT DES [103] | PP-EES | PP-ZES | 30(100) | 30(100) | 26(86.7) | 25(83.3) | 22(73.3) | 23(76.7) | 3(10) | 7(23.3) |  |  | 12(40) | 11(36.7) |  |  |
| OCTDESI [104] | PP-PES | PP-PES | 9(21.9) | 3(15.8) | 30(73.1) | 13(68.4) | 24(58.5) | 12(63.2) | 22(53.6) | 12(63.2) | 10(24.3) | 8(42.1) | 7(17.1) | 5(26.3) |  |  |
| ONYX ONE [105] | PP-ZES | PF-UES | 388(38.7) | 382(38.5) | 796(79.4) | 807(81.3) | 643(64.1) | 619(62.3) | 93(9.4) | 108(10.9) | 237(23.6) | 230(23.2) | 264(26.3) | 249(25.1) | 77(7.7) | 66(6.6) |
| ORIENT [106] | PA-SES | PP-ZES | 63(25.2) | 33(27) | 162(64.8) | 81(66.4) | 134(53.6) | 66(54.1) | 66(26.4) | 35(28.7) | 34(13.6) | 18(14.8) |  |  | 2(0.8) | 0(0.0) |
| ORTALANI [107] | PP-SES | BMS | 8(15) | 9(17) | 31(60) | 38(73) | 41(79) | 42(81) | 27(52) | 31(60) | 6(12) | 11(21) | 13(25) | 17(33) | 1(2) | 1(2) |
| Pache et al [108] | PP-SES | BMS | 72(29) | 82(33) | 137(55) | 154(62) | 134(54) | 128(51) | 49(20) | 40(16) |  |  | 81(320 | 76(30) | 19(8) | 18(7) |
| PAINT [109] | PA-PES | BMS | 40(28.8) | 15(26.3) | 93(83.8) | 49(86) |  |  |  |  | 17(15.3) | 10(17.5) | 33(27.9) | 22(38.6) | 10(9.0) | 2(3.5) |
| PAINT | PA-SES | BMS | 37(34.9) | 15(26.3) | 94(88.7) | 49(86) |  |  |  |  | 16(15.1) | 10(17.5) | 36(34) | 22(38.6) | 6(5.7) | 2(3.5) |
| PANDA III [110] | PA-SES | PA-SES | 275(23.4) | 295(25.1) | 724(61.7) | 723(61.6) | 368(31.4) | 364(31.0) | 437(37.2) | 442(37.7) | 122(10.4) | 160(13.6) | 464(39.5) | 483(41.1) | 3(0.3) | 4(0.3) |
| PASEO [111] | BMS | NA-PES | 21(23.3) | 23(25.6) | 24(26.7) | 22(24.4) |  |  | 22(24.4) | 24(26.7) | 3(3.3) | 4(4.4) | 13(14.4) | 11(12.2) | 7(7.8) | 6(6.7) |
| PASEO | BMS | NA-SES | 21(23.3) | 23(25.6) | 24(26.7) | 22(24.4) |  |  | 22(24.4) | 24(26.7) | 3(3.3) | 4(4.4) | 13(14.4) | 11(12.2) | 7(7.8) | 6(6.7) |
| PASSION [112] | BMS | NA-PES | 31(10.0) | 37(12.0) | 95(30.6) | 98(31.7) | 72(23.2) | 86(27.8) | 165(53.2) | 154(49.8) | 14(4.5) | 13(4.2) | 14(4.5) | 18(5.8) | 2(0.6) | 2(0.6) |
| Petronio et al [113] | PP-PES | PP-SES | 13(31.0) | 8(18.6) | 28(66.7) | 26(60.5) | 30(71.4) | 26(60.5) | 20(47.6) | 18(41.9) |  |  | 15(35.7) | 11(25.6) |  |  |
| PIONEER III [114] | PA-SES | PP-EES | 331(30.5) | 163(30.1) | 806(74.2) | 379(69.9) | 837(77.1) | 413(76.2) | 665(61.2) | 322(59.4) | 304(28.0) | 166(30.6) | 189(17.4) | 101(18.6) | 53(4.9) | 23(4.2) |
| PLATINUM [115] | PP-EES | PP-EES | 169(22.0) | 191(25.1) | 544(70.9) | 558(73.2) | 598(78.2) | 579(76.2) | 158(21.0) | 131(17.7) |  |  | 160(21.0) | 160(21.1) |  |  |
| PLATINUM PLUS [116] | PP-EES | PP-EES | 568(29.1) | 280(27.2) | 1,282(68) | 684(68.6) | 1,195(63) | 620(62.2) | 414(22.0) | 208(20.9) |  |  | 422(21.7) | 250(24.3) |  |  |
| Pourmoghaddas [117] | PP-EES | BMS | 17(39.5) | 11(27.5) | 24(55.8) | 11(27.5) | 8(18.6) | 5(12.5) | 11(25.6) | 9(23.1) |  |  | 14(32.6) | 4(10.3) |  |  |
| PRAGUE-18 [118] | NA-DES | BMS | 157(21.0) | 62(20.9) | 369(49.3) | 164(55.4) | 269(35.9) | 93(31.4) | 485(64.8) | 179(60.5) | 47(6.3) | 16(5.4) | 52(6.9) | 21(7.1) | 7(0.9) | 4(1.4) |
| PRISON II [119] | BMS | PP-SES | 16(16.0) | 10(10.0) | 46(46.0) | 45(45.0) | 90(90.0) | 90(90.0) | 40(40.0) | 34(34.0) | 16(16.0) | 18(18.0) | 51(51.0) | 47(47.0) | 2(2.0) | 3(3.0) |
| PRISON III [120] | PP-ZES | PP-SES | 32(21.3) | 23(14.9) | 73(48.6) | 77(50) | 139(92.6) | 136(88.3) | 46(30.6) | 49(31.8) | 26(17.3) | 26(16.8) |  |  | 8(5.3) | 6(3.8) |
| PRISION IV [121] | PP-EES | PA-SES | 31(18.8) | 34(20.6) | 148(89.7) | 154(93.3) | 161(97.6) | 155(93.9) | 49(29.7) | 59(35.8) | 47(28.5) | 50(30.3) | 52(31.5) | 48(29.1) | 6(3.6) | 11(6.7) |
| PRODIGY [122] | BMS | NA-ZES | 118(24) | 118(24) | 376(75) | 342(69) | 254(51) | 263(53) | 126(25) | 128(26) |  |  | 114(23) | 121(24) | 45(9) | 57(11) |
| PRODIGY | BMS | NA-PES | 118(24) | 140(28) | 376(75) | 365(73) | 254(51) | 281(56) | 126(25) | 111(22) |  |  | 114(23) | 156(31) | 45(9) | 54(11) |
| PRODIGY | BMS | NA-EES | 118(24) | 120(24) | 376(75) | 355(71) | 254(51) | 296(59) | 126(25) | 112(22) |  |  | 114(23) | 143(29) | 45(9) | 61(12) |
| PROMISE [123] | PP-EES | PP-PES | 128(30.1) | 141(33.2) | 266(62.6) | 250(58.8) | 265(62.3) | 269(63.3) | 115(27.1) | 126(29.6) |  |  | 40(9.4) | 40(9.4) |  |  |
| PROSIT [124] | NA-PES | NA-SES | 34(22.1) | 44(28.6) | 70(45.5) | 63(40.9) | 45(29.2) | 35(22.7) | 95(61.7) | 86(55.8) |  |  |  |  |  |  |
| PROTECT [125] | PP-ZES | PP-SES | 1174(27) | 1236(28) | 2814(65) | 2759(63) | 2694(62) | 2734(63) | 1084(25) | 1098(25) | 534(12) | 556(13) | 884(20) | 907(21) | 199(5) | 224(5) |
| RAVEL [126] | BMS | PP-SES | 19(16) | 25(21) | 75(62) | 72(61) | 46(38) | 51(43) | 32(27) | 39(33) |  |  | 46(38) | 40(34) |  |  |
| REALITY [127] | PP-PES | PP-SES | 187(27.3) | 192(28.7) | 448(65.5) | 452(67.6) | 497(72.7) | 468(70.0) | 138(20.2) | 147(22.0) | 159(23.2) | 136(20.3) | 289(42.3) | 258(38.6) | 54(7.9) | 46(6.9) |
| RECOVERY [128] | PA-SES | PF-SES | 43(19.9) | 46(21.3) | 116(53.7) | 130(60.2) | 27(12.5) | 37(17.1) | 97(44.9) | 93(43.1) | 21(9.7) | 20(9.3) | 29(13.4) | 28(13) | 1(0.5) | 1(0.5) |
| RECRE8 [129] | PF-AES | PP-ZES | 149(20.0) | 155(20.8) | 411(55.2) | 412(55.2) | 340(45.8 | 325(43.5) | 191(25.7 | 193(25.9) | 166(22.3) | 138(18.5) | 158(21.2) | 139(18.6) | 71(9.5) | 67(9.0) |
| REMEDEE [130] | PP-PES | NA-SES | 41(33.1) | 22(37.3) | 100(80.6) | 45(76.3) | 102(82.3) | 43(72.9) | 26(21.0) | 10(16.9) | 29(23.4) | 12(20.3) | 31(25.0) | 16(27.1) | 4(3.2) | 2(3.4) |
| RESERVOIR [131] | PF-AES | PP-EES | 56(100.0) | 56(100.0) | 46(82.1) | 49(87.5) | 45(80.4) | 47(83.9) | 30(53.6) | 35(62.5) | 22(39.3) | 19(33.9) | 13(23.2) | 17(30.4) | 1(1.8) | 1(1.8) |
| RESET [132] | PP-EES | PP-SES | 726(45) | 713(45) | 1269(79) | 1290(81) | 1189(74) | 1204(75) | 332(21) | 326(20) | 757(47) | 811(51) | 471(29) | 496(31) | 62(3.9) | 99(6.2) |
| RESOLUTE [133] | PP-ZES | PP-EES | 268(23.5) | 270(23.4) | 810(71.1) | 821(71.3) | 729(63.9) | 780(67.7) | 302(26.5) | 305(26.5) | 363(31.8) | 370(32.1) | 324(28.9) | 341(30.4) | 114(10.0) | 110(9.5) |
| RESOLUTE CHINA [134] | PP-ZES | PP-PES | 51(25.8) | 59(29.2) | 129(65.2) | 132(65.3) | 78(39.4) | 93(46.0) | 73(36.9) | 89(44.1) | 29(14.6) | 42(20.8) | 67(34.5) | 67(33.8) |  |  |
| Sakakibara et al [135] | PP-EES | PP-SES | 37(74.0) | 33(66.0) | 34(68.0) | 36(72.0) | 14(28.0) | 18(36.0) | 11(22.0) | 13(26.0) |  |  | 4(8.0) | 2(4.0) |  |  |
| SCANDSTENT [136] | BMS | PP-SES | 29(18) | 29(18) | 75(46) | 60(38) | 132(81) | 134(84) | 59(36) | 53(33) |  |  | 88(54) | 80(50) |  |  |
| SCORPIUS [137] | BMS | PP-SES | 94(100) | 96(100) | 87(93) | 90(94) | 77(82) | 76(80) | 19(20) | 17(18) |  |  | 33(35) | 32(34) | 2(2) | 6(6) |
| SEA-SIDE [138] | NA-EES | PP-SES | 25(33) | 19(25) | 52(69) | 52(69) |  |  | 9(12) | 19(25) |  |  | 10(13) | 6(8) | 4(5) | 5(7) |
| SELECTION [139] | BMS | PP-PES | 3(7.5) | 7(17.5) | 15(37.5) | 22(55) | 13(32.5) | 13(32.5) | 25(62.5) | 18(45) |  |  | 2(5) | 1(2.5) |  |  |
| SENIOR [140] | PA-EES | BMS | 158(27) | 157(26) | 427(72) | 488(81) |  |  | 43(7) | 38(6) | 139(23) | 143(24) | 109(18) | 80(13) | 36(6) | 42(7) |
| Separham et al [141] | PA-BES | PP-EES | 28(28) | 32(32) | 48(48) | 37(37) | 36(36) | 44(44) | 26(26) | 20(20) |  |  |  |  |  |  |
| SESAMI [142] | BMS | PP-SES | 28(17.5) | 37(23.7) | 87(54.3) | 98(58.7) |  |  | 91(56.8) | 83(51.7) | 15(9.4) | 17(10.6) | 9(5.6) | 20(12.5) | 1(0.6) | 1(0.6) |
| SES-SMART [143] | BMS | PP-SES | 25(19.4) | 39(29.7) | 84(65.1) | 81(64.3) | 79(61.2) | 83(64.8) | 24(18.6) | 18(14.1) | 26(20.3) | 29(22.7) | 38(29.5) | 36(28.1) | 13(10.2) | 8(6.3) |
| Shen et al [144] | PA-AT | PP-SES |  |  |  |  |  |  |  |  |  |  |  |  |  |  |
| SIRIUS [145] | BMS | PP-SES | 131(25) | 148(28) | 359(68) | 354(68) | 382(73) | 387(75) | 95(18) | 116(22) |  |  | 147(28) | 171(33) |  |  |
| SIRTAX [146] | PP-PES | PP-SES | 108(21.5) | 93(18.3) | 302(60.0) | 317(62.3) | 305(60.6) | 290(57.0) | 184(36.6) | 181(35.6) |  |  | 145(28.8) | 151(29.7) |  |  |
| SORT OUT II [147] | PP-PES | PP-SES | 162(15.2) | 152(14.7) |  |  |  |  | 420(39.4) | 405(39.2) | 167(15.7) | 185(17.4) | 282(26.4) | 268(25.2) | 52(4.9) | 57(5.5) |
| SORT OUT III [148] | PP-ZES | PP-SES | 169(15) | 168(14) | 605(52) | 569(49) |  |  | 347(30) | 341(29) | 233(20) | 195(17) | 285(25) | 302(26) | 79(7) | 73(6) |
| SORT OUT IV [149] | PP-EES | PP-SES | 194(14.0) | 196(14.2) | 689(56.7) | 649(53.8) |  |  | 344(29.2) | 353(30.4) | 264(21.5) | 250(20.6) | 276(22.6) | 259(21.3) | 118(9.6) | 97(8.0) |
| SORT OUT V [150] | PA-BES | PP-SES | 185(15.1) | 189(15.3) | 682(57.8) | 653(54.9) |  |  | 385(33.6) | 381(33.1) | 205(17.3) | 196(16.5) | 209(17.7) | 206(17.3) | 96(8.1) | 71(5.9) |
| SORT OUT VI [151] | PP-ZES | PA-BES | 265(17.6) | 270(18.0) | 872(59.7) | 850(58.1) | |  | 443(30.7) | 439(30.7) | 277(18.7) | 324(22.0) | 275(18.7) | 288(19.7) | 126(8.4) | 101(6.8) |
| SORT OUT VII [152] | PA-BES | PA-SES | 236(18.7) | 235(18.6) | 713(58.1) | 699(56.4) | 711(57.6) | 706(56.7) | 355(29.1) | 399(32.5) | 237(19.0) | 256(20.4) | 215(17.4) | 222(17.8) | 100(8.0) | 96(7.6) |
| SORT OUT VIII [153] | PA-EES | PA-BES | 250(18) | 262(19) | 777(57) | 795(58) | 748(55) | 724(53) | 418(32) | 385(29) | 246(18) | 277(20) | 241(18) | 226(17) | 144(10) | 112(8) |
| SORT OUT IX [154] | PF-BES | PA-SES | 304(19.3) | 303(19.2) | 893(59.0) | 850(56.0) | 830(55.0) | 777(51.5) | 443(29.8) | 437(29.3) | 322(20.9) | 311(20.9) | 224(14.7) | 234(15.2) | 130(8.4) | 108(7.0) |
| SORT OUT X [155] | PA-SES | PA-SES | 279(17.7) | 271(17.3) | 835(53.7) | 871(56.6) | 783(50.3) | 783(50.7) | 410(29.1) | 429(30.5) | 295(18.9) | 303(19.7) | 240(15.4) | 221(14.5) | 111(7.1) | 89(5.8) |
| SPIRIT FIRST [156] | PP-EES | BMS | 3(11) | 3(10) | 19(70) | 12(41) | 19(70) | 22(76) | 8(28) | 9(31) | 5(19) | 2(7) | 6(24) | 4(14) |  |  |
| SPIRIT II [157] | PP-EES | PP-PES | 51(23) | 18(24) | 149(67) | 50(65) | 153(69) | 57(75) | 71(32) | 23(30) | 8(4) | 3(4) | 78(35) | 19(25) |  |  |
| SPIRIT III [158] | PP-EES | PP-PES | 250(37.4) | 110(33.4) | 510(76.2) | 245(74.0) |  |  | 154(23.4) | 73(22.5) |  |  | 130(19.9) | 59(18.0) |  |  |
| SPIRIT IV [159] | PP-EES | PP-PES | 995(40.5) | 518(42.7) | 1899(77.4) | 935(76.1) |  |  | 527(21.9) | 269(22.4) |  |  | 504(21.1) | 239(19.9) |  |  |
| SPIRIT V DIABETIC [160] | PP-EES | PP-PES | 218(100) | 106(100) | 170(78) | 783(78) |  |  |  |  |  |  | 74(34) | (34(32) |  |  |
| STRATEGY [161] | BMS | PP-SES | 15(17) | 11(12) | 48(55) | 44(50) |  |  | 34(39) | 36(41) | 4(5) | 2(2) | 11(13) | 8(9) | 2(2) | 2(2) |
| TARGET ALL COMERS [162] | PP-EES | PA-SES | 191(23.0) | 197(24.0) | 519(62.5) | 429(59.9) | 425(51.2) | 435(53.0) | 533(64.2) | 488(59.5) | 262(31.6) | 236(28.7) | 206(24.8) | 178(21.7) | 62(7.5) | 69(8.4) |
| TARGET I [163] | PP-EES | PA-SES | 31(13.7) | 39(16.9) | 131(57.7) | 138(59.7) | 61(26.9) | 53(22.9) | 90(39.6) | 90(39.0) | 11(4.8) | 13(5.6) | 45(19.8) | 49(21.2) |  |  |
| TALENT [164] | PP-EES | PA-SES | 178(24.9) | 157(21.8) | 472(66.1) | 470(65.3) | 428(60.2) | 444(61.8) | 172(24.1) | 176(24.5) | 153(21.4) | 175(24.3) | 128(17.9) | 136(18.9) | 55(7.7) | 33(4.6) |
| TAXI [165] | PP-PES | PP-SES | 33(32.4) | 36(36) | 60(58.8) | 63(63) | 77(75.5) | 79(79) | 26(25.5) | 26(26) | 28(27.5) | 34(34) | 33(32.4) | 29(29) | 22(21.6) | 15(15) |
| TAXUS I [166] | BMS | PP-PES | 4(13) | 7(23) | 19(63) | 20(65) | 24(81) | 25(81) | 14(47) | 17(54) |  |  | 9(30) | 8(26) |  |  |
| TAXUS II [167] | BMS | PP-PES | 23(17) | 19(14) | 81(60) | 75(56) |  |  | 32(24) | 39(29) | 23(17) | 17(13) | 53(39) | 56(42) |  |  |
| TAXUS IV [168] | BMS | PP-PES | 220(33.8) | 206(31.1) | 450(69) | 467(70.5) | 428(65.6) | 430(65) | 131(20.1) | 155(23.4) |  |  | 195(29.9) | 202(30.5) |  |  |
| TAXUS V [169] | BMS | PP-PES | 173(29.9) | 183(31.7) | 426(73.6) | 441(76.4) | 428(73.9) | 417(72.3) | 115(19.9) | 122(21.1) |  |  | 152(26.3) | 181(31.4) |  |  |
| TAXUS VI [170] | BMS | PP-PES | 50(22) | 39(17.8) | 132(58.1) | 126(57.5) | 163(73.4) | 149(70.3) | 52(23.9) | 47(22.5) | 47(20.7) | 39(17.9) |  |  |  |  |
| TUXEDO [171] | PP-EES | PP-PES | 382(41.7) | 365(39.9) | 604(65.9) | 613(67.1) | 710(77.5) | 702(76.8) | 145(15.8) | 128(14.0) | 68(7.4) | 77(8.4) | 366(40.0) | 376(41.1) | 18(2.0) | 14(1.5) |
| TIDE [172] | PF-TiNO | PP-ZES | 30(19.7) | 28(18.7) | 105(69.1) | 113(75.3) | 115(75.7) | 122(81.3) | 53(34.9) | 43(28.7) | 39(25.7) | 38(25.3) | 42(27.6) | 32(21.3) | 12(7.9) | 4(2.7) |
| TIDE-ACS [173] | PF-TiNO | PP-EES | 140(4.2) | 63(12.5) | 463(46.8) | 219(43.6) | 410(41.5) | 202(40.2) | 309(31.2) | 180(35.9) | 69(7.0) | 33(6.6) | 75(7.6) | 45(9.0) | 6(6.0) | 6(1.2) |
| TWENTE [174] | PP-ZES | PP-EES | 158(22.7) | 143(20.6) | 386(55.4) | 387(55.8) | 392(57.0) | 411(61.4) | 176(25.3) | 164(23.6) | 139(19.9) | 149(21.5) | 213(30.6) | 237(34.1) | 68(9.8) | 80(11.5) |
| TWENTE II (DUTCH PEERS) [175] | PP-ZES | PP-EES | 167(18) | 157(17) | 500(55) | 484(53) | 418(46) | 430(48) | 213(24) | 231(26) | 182(20) | 167(18) | 207(23) | 190(21) | 84(9) | 89(10) |
| TYPHOON [176] | BMS | PP-SES | 61(17.1) | 55(15.5) | 152(42.6) | 137(38.6) | 156(43.7) | 144(40.6) | 186(52.1) | 170(47.9) | 21(5.9) | 9(2.5) |  |  |  |  |
| Yin et al [177] | PP-DES | BMS | 297(18.3) | 124(15.8) | 905(55.9) | 421(53.5) | 557(34.4) | 229(29.1) | 857(52.9) | 444(56.4) | 274(16.9) | 99(12.6) | 597(36.9) | 386(49.0) | 35(2.2) | 16(2.0) |
| Wessely et al [178] | PA-PES | PA-SES | 12(27) | 16(35) | 32(71) | 31(67) | 25(56) | 26(57) | 6(13) | 8(13) |  |  | 16(36) | 13(28) | 6(13) | 4(9) |
| X-MAN [179] | PP-EES | PF-CC | 22(29) | 17(23) | 37(84) | 37(51) | 36(48) | 37(49) | 46(61) | 48(64) |  |  |  |  |  |  |
| XAMI [180] | PP-EES | PP-SES | 36(8.9) | 25(11.3) | 119(29.5) | 66(29.9) |  |  | 220(54.5) | 122(55.2) | 17(4.2) | 6(2.8) | 23(5.7) | 14(6.2) | 1(0.2) | 4(1.8) |
| ZEST [181] | PP-ZES | PP-SES | 268(30.4) | 247(28.1) | 552(62.5) | 517(58.9) | 466(52.8) | 451(51.4) | 236(26.7) | 256(29.2) | 75(8.5) | 82(9.3) | 30(3.4) | 39(4.4) | 6(0.7) | 6(0.7) |
| ZEST | PP-ZES | PP-PES | 268(30.4) | 245(27.7) | 552(62.5) | 540(61.1) | 466(52.8) | 446(50.5) | 236(26.7) | 243(27.5) | 75(8.5) | 83(9.4) | 30(3.4) | 41(4.6) | 6(0.7) | 5(0.6) |
| ZEST-AMI [182] | PP-ZES | PP-SES | 30(27.8) | 29(26.4) | 52(48.1) | 42(38.2) | 52(48.1) | 45(40.9) | 56(51.9) | 62(56.4) | 7(6.5) | 1(0.9) |  |  |  |  |
| ZEST-AMI | PP-ZES | PP-PES | 30(27.8) | 26(23.6) | 52(48.1) | 51(53.6) | 52(48.1) | 51(46.4) | 56(51.9) | 68(61.8) | 7(6.5) | 2(1.8) |  |  |  |  |
| ZEUS [183] | BMS | PP-ZES | 205(25.5) | 215(26.8) | 605(75.2) | 612(76.3) | 399(49.6) | 381(47.5) | 169(21.0) | 167(20.8) | 149(18.5) | 155(19.3) | 190(23.6) | 194(24.2) | 59(7.3) | 54(6.7) |
| Zhang Q et al [184] | PP-PES | PP-SES | 43(21) | 76(31) | 140(69) | 180(73) | 67(33) | 76(31) | 95(47) | 145(59) |  |  |  |  |  |  |
| Zhang L et al [185] | PA-R | PF-PES | 24(28.24) | 25(30.49) | 59(69.42) | 49(59.76) | 49(57.65) | 40(48.78) | 40(47.06) | 37(45.12) | 3(3.53) | 3(3.66) | 6(7.06) | 8(9.76) | 0 | 0 |
| Zhang Y et al [186] | PF-PES | PA-SES | 83(25.38) | 110(32.2) | 211(64.53) | 232(68) | 114(34) | 197(57.77) | 134(40.9) | 151(44.28) | 26(7.95) | 30(8.80) | 16(4.89) | 20(5.87) | 0 | 1(0.29) |
| Zhang Y et al | PF-PES | PP-SES | 83(25.38) | 89(27.73) | 211(64.53) | 209(65.1) | 114(34.8) | 114(35.51) | 134(40.9) | 125(38.94) | 26(7.95) | 38(11.84) | 16(4.89) | 15(4.67) | 0 | 0 |
| ZOMAXX I [187] | PP-ZES | PP-PES | 44(22) | 51(26) | 137(69) | 132(67) | 155(78) | 142(72) | 48(24) | 37(19) | 40(20) | 49(25) | 58(29) | 57(29) | 9(4.5) | 2(1.0) |
| ZOMAXX II [188] | PP-ZES | Paclitaxel | 150(27) | 146(27) | 440(79) | 428(79) | 429(77) | 401(74) | 134(24) | 103(19) | 117(21) | 119(22) | 120(22) | 119(22) | 30(5.4) | 39(7.2) |
| XIMA [189] | PP-EES | BMS | 102(25.6) | 97(24.2) | 300(75.1) | 312(77.6) | 230(57.6) | 212(52.9) | 20(5.0) | 16(4.0) | 51(12.8) | 41(10.2) | 119(29.8) | 86(21.5) | 28(7.0) | 17(4.2) |
| Xu et al [190] | PP-ZES | PA-SES | 40(25.6) | 12(26.2) | 89(57.0) | 92(54.8) | 36(23.1) | 35(20.8) | 68(43.6) | 78(46.4) | 16(10.3) | 19(11.3) | 16(10.3) | 53(31.6) | 1(0.6) | 1(0.6) |
| TINOX [191] | PF-TiNO | BMS | 15(34) | 16(34) | 33(73) | 34(72) | 38(84) | 32(68) | 16(36) | 21(45) |  |  | 19(42) | 18(38) |  |  |
| TITAX AMI [192] | PF-TiNO | PP-PES | 48(22) | 33(16) | 122(57) | 106(50) | 141(66) | 151(72) | 113(53) | 97(46) | 22(10) | 10(5) | 33(15) | 20(9) | 16(7) | 13(6) |
| BASE ACS [193] | PF-TiNO | PP-EES | 65(15.6) | 75(18.3) | 201(48.2) | 212(51.7) | 191(45.8) | 197(48) | 144(34.5) | 134(32.7) | 40(9.6) | 43(10.5) | 56(13.4) | 40(9.8) | 20(4.8) | 17(4.1) |
| TITANIC XV [194] | PF-TiNO | PP-EES | 83(100) | 90(100) | 64 (77.1) | 65 (72.2) | 46 (55.4) | 63 (70.0) | 27 (32.5) | 38 (42.2) | 7 (8.4) | 10 (11.1) | 9 (10.8) | 14 (15.6) | 2 (2.4) | 2 (2.2) |
| CIBELES [195] | PP-EES | PP-SES | 43(40.6) | 32(31.7) | 73(68.9) | 68(67.3) | 70(66) | 78(77.2) | 53(50) | 62(61.2) | 27(25.5) | 43(42.6) | 35(106) | 43(42.6) | 5(4.7) | 4(4) |
| PIONEER [196] | PP-ZES | PA-SES | 20(23) | 24(28.9) | 57(65.5) | 47(56.6) | 53(61.6) | 54(65.1) | 22(25.6) | 21(25.6) | 16(18.4) | 18(21.7) | 15(17.2) | 15(18.1) | 3(3.4) | 4(4.8) |

**Supplemental Table 4: Detailed trial-level proportion of clinical presentation of the included population in intervention (i)and control (c) groups.**

| STUDY | Stent Comparison | | ACS, n(%) | | Silent Ischemia, n(%) | | Stable Angina, n(%) | | Unstable Angina, n(%) | | NSTEMI, n(%) | | STEMI, n(%) | |
| --- | --- | --- | --- | --- | --- | --- | --- | --- | --- | --- | --- | --- | --- | --- |
|  | i | c | i | c | i | c | i | c | i | c | i | c | i | c |
| ACTION [1] | PF-DES | BMS |  |  |  |  | 4(3.4) | 7(5.9) | 13.5(11.3) | 15(12.7) |  |  |  |  |
| APPENDIX-AMI [2] | PP-EES | PP-SES |  |  |  |  | 251(50.4) | 275(57.4) |  |  | 179(35.9) | 160(33.4) | 68(13.7) | 44(9.2) |
| BASKET [3] | PP-SES | BMS | 160(60) | 162(58) |  |  | 104(39) | 119(42) | 96(36) | 101(36) |  |  | 64(24) | 61(22) |
| BASKET | PP-PES | BMS | 155(55) | 162(58) |  |  | 126(45) | 119(42) | 104(37) | 101(36) |  |  | 51(18) | 61(22) |
| BASKET-PROVE [4] | PP-SES | BMS | 509(65) | 480(63) |  |  | 266(34) | 285(37) | 244(31) | 246(32) |  |  | 265(34) | 234(31) |
| BASKET-PROVE | PP-EES | BMS | 503(65) | 480(63) |  |  | 271(35) | 285(37) | 264(34) | 246(32) |  |  | 239(31) | 234(31) |
| BASKET-PROVE II [5] | PA-BES | BMS | 491(64) | 461(60) |  |  | 274(36) | 300(39) |  |  | 263(34) | 253(33) | 228(30) | 208(27) |
| BASKET-PROVE II | PP-EES | BMS | 494(64) | 461(60) |  |  | 271(35) | 300(39) |  |  | 271(35) | 253(33) | 223(29) | 208(27) |
| BASKET-SMALL [6] | PP-ZES | PP-PES | 48(52.8) | 58(58) |  |  | 43(47.3) | 42(42) | 30(33) | 39(39) |  |  | 18(19.8) | 19(19) |
| BIODEGRADE [7] | PA-BES | PA-SES | 782(67.4) | 783(67.1) | 65(5.6) | 55(4.7) | 313(27) | 328(28.1) | 424(36.6) | 424(36.4) | 257(22.2) | 238(20.4) | 101(8.7) | 121(10.4) |
| BIOFLOW II [8] | PA-SES | PP-EES |  |  |  |  |  |  |  |  |  |  |  |  |
| BIOFLOW IV [9] | PA-SES | PP-EES |  |  |  |  |  |  |  |  |  |  |  |  |
| BIOFLOW V [10] | PA-SES | PP-EES | 454(51) | 223(50) | 109(12) | 61(14) | 428(48) | 213(47) | 347(39) | 175(39) |  |  |  |  |
| BIOFLOW VI [11] | PA-SES | PP-EES |  |  |  |  |  |  | 161(73.2) | 184(83.6) |  |  |  |  |
| BIOFREEDOM FIM [12] | PF-BES | PP-PES |  |  | 4(6.5) | 6(10) | 48(78.8) | 46(76.7) | 7.5(12.3) | 4(6.7) |  |  |  |  |
| BIOSTEMI [13] | PA-SES | PP-EES |  |  |  |  |  |  |  |  |  |  |  |  |
| BIONICS [14] | PP-RES | PP-ZES | 390(40.7) | 372(38.7) |  |  | 568(59.3) | 589(61.3) |  |  |  |  |  |  |
| BIONYX [15] | PP-ZES | PA-SES | 880(70.8) | 885(71.1) | 363(29.2) | 360(28.9) | 363(29.2) | 360(28.9) | 254(20.4) | 236(19) | 344(27.7) | 310(24.9) | 282(22.7) | 339(27.2) |
| BIO-RESORT [16] | PP-EES | PA-ZES | 816(69) | 815(70) |  |  | 356(30) | 358(31) | 192(16) | 219(19) | 247(21) | 270(23) | 377(32) | 326(28) |
| BIO-RESORT | PA-SES | PA-ZES | 818(70) | 815(70) |  |  | 351(30) | 358(31) | 209(18) | 219(19) | 239(20) | 270(23) | 370(32) | 326(28) |
| BIOSCIENCE [17] | PA-SES | PP-EES | 577(54.2) | 554(52.4) | 161(15.1) | 171(16.2) | 325(30.6) | 331(31.3) | 78(7.3) | 74(7) | 288(27.1) | 284(26.9) | 211(19.9) | 196(18.6) |
| CATOS [18] | PP-ZES | PP-SES |  |  |  |  |  |  |  |  |  |  |  |  |
| CERVINKA ET AL [19] | PP-SES | PP-PES | 22.2(60) | 19(59) |  |  | 15(40) | 14(41) | 16(42) | 14(43) | 16(42) | 14(43) | 7(18) | 5(16) |
| CENTURY II [20] | PA-SES | PP-EES | 124(22.5) | 136(24.7) | 82(14.9) | 101(18.4) | 270(49) | 253(46) | 75(13.6) | 60(10.9) | 95(17.2) | 105(19.1) | 29(5.3) | 31(5.6) |
| CHOICE [21] | PP-EES | PA-BES | 210(32.9) | 202(31.9) |  |  | 145(22.7) | 167(26.3) |  |  | 146(22.9) | 131(20.7) | 64(10) | 71(11.2) |
| CHOICE | PA-BES | PP-ZES | 202(31.9) | 207(32.4) |  |  | 167(26.3) | 151(23.6) |  |  | 131(20.7) | 133(20.8) | 71(11.2) | 74(11.6) |
| CORACTO [22] | BMS | PA-SES |  |  |  |  |  |  |  |  |  |  |  |  |
| COMFORTABLE AMI [23] | PA-BES | BMS |  |  |  |  |  |  |  |  |  |  |  |  |
| COMPARE [24] | PP-EES | PP-PES | 541(60) | 534(59) | 23(3) | 17(2) | 331(37) | 349(39) | 107(12) | 105(12) | 194(22) | 217(24) | 240(27) | 212(23) |
| COMPARE II [25] | PA-BES | PP-EES | 1039(57.9) | 527(57.8) | 57(3.2) | 30(3.3) | 699(38.9) | 355(38.9) | 194(10.8) | 88(9.7) | 474(26.4) | 242(26.5) | 371(20.7) | 197(21.6) |
| COSTAR II [26] | PA-PES | PP-PES |  |  |  |  |  |  | 291(29.4) | 220(32.1) |  |  |  |  |
| CREST MI [27] | PP-ZES | PP-SES |  |  |  |  |  |  |  |  |  |  |  |  |
| CRE8 (NEXT) [28] | PF-AES | PP-PES |  |  |  |  |  |  |  |  |  |  |  |  |
| C-SIRIUS [29] | PP-SES | BMS |  |  |  |  | 5(10) | 7(14) | 24(48) | 27(54) |  |  |  |  |
| Dang et al [30] | PP-SES | PF-PES |  |  |  |  |  |  |  |  |  |  |  |  |
| DEBATER [31] | PP-SES | BMS |  |  |  |  |  |  |  |  |  |  |  |  |
| DEBFIRST [32] | PF-PES+BMS | PP-ZES |  |  |  |  | 42(46.7) | 43(47.8) | 20(22.2) | 28(31.1) | 28(31.1) | 19(21.1) |  |  |
| DECODE [33] | NA-SES | BMS |  |  | 5(9.3) | 1(3.4) | 35(64.8) | 20(69) | 10(18.5) | 8(27.6) |  |  |  |  |
| DEDICATION [34] | NA-DES | BMS |  |  |  |  |  |  |  |  |  |  |  |  |
| DES-DIABETES [35] | NA-SES | NA-PES |  |  |  |  | 86(43) | 82(41) | 80(40) | 67(33.5) |  |  |  |  |
| DESSERT [36] | PP-SES | BMS |  |  | 6(8) | 9(12) | 22(29) | 21(28) | 35(47) | 36(48) |  |  | 12(16) | 9(12) |
| DESSOLVE II [37] | PA-SES | PP-ZES |  |  |  |  |  |  |  |  |  |  |  |  |
| DESSOLVE III [38] | PA-SES | PP-EES |  |  |  |  | 289(41) | 287(41) | 162(23) | 166(24) | 149(21) | 133(19) | 103(15) | 109(16) |
| DIABEDES [39] | PP-SES | PP-PES | 25(33) | 25(33) |  |  |  |  |  |  |  |  |  |  |
| DIABETES [40] | PP-SES | BMS |  |  | 4(5) | 7(8.8) | 24(30) | 24(30) | 36(45) | 28(35) | 12(15) | 16(20) |  |  |
| Diaz de la llera et al [41] | BMS | PP-SES |  |  |  |  |  |  |  |  |  |  |  |  |
| DIRECT II [42] | PA-SES | PP-ZES |  |  |  |  |  |  |  |  |  |  |  |  |
| ENDEAVOR II [43] | PP-ZES | BMS |  |  |  |  |  |  | 179(30) | 180(30) |  |  |  |  |
| ENDEAVOR III [44] | PP-ZES | PP-SES |  |  |  |  |  |  | 156(59.3) | 52(55.9) |  |  |  |  |
| ENDEAVOR IV [45] | PP-ZES | PP-PES |  |  |  |  | 281(45.6) | 292(47..9) | 318(51.6) | 304(49.9) |  |  |  |  |
| Erglis et al [46] | BMS | NA-PES | 10(19) | 8(16) | 0(0) | 1(2) | 43(81) | 41(82) |  |  |  |  |  |  |
| ESSENCE-DIABETES [47] | PP-EES | PP-SES |  |  |  |  | 85(57.0) | 90(59.6) | 60(40.3) | 49(32.5) |  |  |  |  |
| EUCATAX [48] | PA-PES | BMS |  |  |  |  |  |  | 126(59.7) | 141(66.8) |  |  | 43(20.4) | 36(17.1) |
| EVERBIO II [49] | PA-EES | PA-BES |  |  | 6(8) | 15(19) | 47(59) | 27(34) | 5(6) | 9(11) | 16(20) | 21(26) | 6(8) | 8(10) |
| EVOLVE [50] | PP-EES | PA-EES |  |  |  |  |  |  | 21(21.1) | 21(22.6) |  |  |  |  |
| EVOLVE II [51] | PA-EES | PP-EES |  |  |  |  |  |  | 287(33.9) | 292(34.8) |  |  |  |  |
| EXAMINATION [52] | PP-EES | BMS |  |  |  |  |  |  |  |  |  |  | 751(100) | 747(100) |
| EXCELLA II [53] | PP-NES | PP-ZES |  |  | 15(10.8) | 3(4.2) | 89(64) | 46(64.8) | 35(25.2) | 22(31) |  |  |  |  |
| EXCELLENT [54] | PP-EES | PP-SES |  |  | 39(3.6) | 16(4.4) | 472(43.7) | 172(47.3) | 464(43.0) | 137(37.6) | 76(7.0) | 22(6.0) | 28(2.6) | 17(4.7) |
| E-SIRIUS [55] | PP-SES | BMS |  |  |  |  | 73(44) | 70(42) | 53(30) | 64(36) |  |  |  |  |
| FIBISTEMI [56] | NA-SES | BMS |  |  |  |  |  |  |  |  |  |  |  |  |
| GARA-GARA [57] | PP-SES | PP-PES | 84(19) | 80(17) |  |  |  |  |  |  |  |  |  |  |
| GENESIS [58] | PA-PES | NA-Pim |  |  |  |  |  |  | 10(20.4) | 25(25) |  |  |  |  |
| GENESIS | PA-PES | NA-DES |  |  |  |  |  |  | 10(20.4) | 34(35) |  |  |  |  |
| GRACIA-3 [59] | PP-PES | BMS |  |  |  |  |  |  |  |  |  |  |  |  |
| GISSOC II-GISE [60] | PP-SES | BMS |  |  |  |  |  |  | 17(23.0) | 15(19.2) |  |  |  |  |
| HARMONEE [61] | PA-SES | PP-EES |  |  |  |  |  |  |  |  |  |  |  |  |
| Herdeg C et al. [62] | PP-PES | BMS |  |  |  |  |  |  | 9(13) | 9(13) |  |  |  |  |
| Hong et al [63] | PP-SES | PP-PES |  |  | 2(2.4) | 5(6.0) | 29(34.1) | 30(35.7) | 54(63.5) | 49(58.3) |  |  |  |  |
| HORIZONS-AMI [64] | PP-PES | BMS |  |  |  |  |  |  |  |  |  |  |  |  |
| HOST-ASSURE [65] | NA-EES | NA-ZES |  |  | 119(4.8) | 63(5) | 746(29.8) | 367(29.3) | 903936.1) | 476(38) | 452(18.1) | 209(16.7) | 283(11.3) | 137(10.9) |
| INSPIRON I [66] | NA-SES | BMS |  |  |  |  | 29(76.3) | 15(78.9) | 9(23.7) | 4(21.1) |  |  |  |  |
| ISAR-DIABETES [67] | PP-SES | PP-PES |  |  |  |  |  |  | 56(44.8) | 43(34.4) |  |  |  |  |
| ISAR-LEFT-MAIN [68] | PP-SES | PP-PES | 121(40) | 132(44) |  |  |  |  |  |  |  |  |  |  |
| ISAR-LEFT-MAIN II [69] | PP-ZES | PP-EES | 124(38.3) | 107(32.8) |  |  |  |  |  |  |  |  |  |  |
| ISAR-SMART III [70] | PP-PES | PP-SES |  |  |  |  |  |  | 63(35) | 49(27) |  |  |  |  |
| ISAR-TEST [71] | PF-SES | PP-PES |  |  |  |  |  |  | 94(42) | 99(44) |  |  |  |  |
| ISAR-TEST-2 [72] | PF-RP | PP-SES |  |  |  |  | 192(57.7) | 205(61.2) | 101(30.3) | 85(25.4) |  |  |  |  |
| ISAR-TEST-2 | PP-ZES | PP-SES |  |  |  |  | 89(55.8) | 205(61.2) | 101(29.8) | 85(25.4) |  |  |  |  |
| ISAR-TEST-4 [73] | PA-SES | PP-DES |  |  |  |  | 758(58.4) | 785(60.2) | 374(28.8) | 379(29.1) |  |  |  |  |
| ISAR-TEST-5 [74] | PF-SES+P | PP-ZES |  |  |  |  | 1191(59.5) | 579(57.9) | 596(29.8) | 325(32.5) |  |  |  |  |
| I-LOVE-IT 2 [75] | PA-SES | PP-SES |  |  |  |  | 269(14.7) | 126(13.9) | 1330(72.7) | 691(76.1) |  |  |  |  |
| J-DESSERT [76] | NA-SES | NA-PES |  |  |  |  | 1132(68) | 1088(64.6) | 246(14.8) | 290(17.2) |  |  |  |  |
| Juwana et al [77] | PP-SES | PP-PES |  |  |  |  |  |  |  |  |  |  |  |  |
| Kamoi et al [78] | PP-SES | PP-PES |  |  |  |  |  |  |  |  |  |  |  |  |
| Kim et al [79] | PP-SES | PP-PES |  |  | 2(2.4) | 5(6) | 29(34.1) | 30(35.7) | 54(63.5) | 49(58.3) |  |  |  |  |
| KOMER [80] | NA-ZES | NA-SES |  |  |  |  |  |  |  |  |  |  |  |  |
| KOMER | NA-ZES | NA-PES |  |  |  |  |  |  |  |  |  |  |  |  |
| Laarman et al [81] | NA-PES | BMS |  |  |  |  |  |  |  |  |  |  |  |  |
| Lansky et al [82] | BMS | BMS |  |  |  |  |  |  |  |  |  |  |  |  |
| LEADERS [83] | PA-BES | PP-SES | 470(54.8) | 473(55.7) |  |  | 387(45.2) | 377(44.4) | 190(22.2) | 180(21.2) | 145(16.9) | 153(18.0) | 135(15.8) | 140(16.5) |
| LEADERS FREE [84] | PF-BES | BMS |  |  |  |  | 714(58.5) | 689(56.9) | 177(14.5) | 193(15.9) | 273(22.4) | 281(23.2) | 57(4.7) | 48(4.0) |
| Li et al [85] | PA-SES | PP-SES |  |  |  |  |  |  |  |  |  |  | 164(100) | 168(100) |
| LIPSIA YUKON [86] | PF-SES | PP-PES |  |  |  |  |  |  |  |  |  |  |  |  |
| LISAII [87] | PP-PES | PF-PES |  |  |  |  | 40(50) | 54(64.3) | 40(50) | 30(35.7) |  |  |  |  |
| LONG-DES II [88] | NA-SES | NA-PES | 138(55.2) | 135(54) |  |  | 112(44.8) | 115(46) | 92(36.8) | 84(33.6) |  |  |  |  |
| LONG-DES III [89] | NA-EES | NA-SES |  |  | 30(13.4) | 26(11.5) | 107(47.8) | 97(42.9) | 69(30.8) | 92(40.7) | 18(8) | 11(4.9) |  |  |
| LONG-DES IV [90] | NA-ZES | NA-SES |  |  |  |  | 160(64) | 160(64) | 71(28.4) | 64(25.6) | 19(7.6) | 26(10.4) |  |  |
| LONG-DES V [91] | PA-BES | PP-EES |  |  |  |  | 142(58) | 145(56.9) | 68(27.8) | 74(29) | 35(14.3) | 36(14.1) |  |  |
| MASTER [92] | BMS | NA-D/BMS |  |  |  |  |  |  |  |  |  |  |  |  |
| MASTER STUDY [93] | BMS | PA-SES |  |  |  |  |  |  |  |  |  |  |  |  |
| MERIT-V [94] | PA-SES | PP-EES |  |  | 16(9.4) | 5(5.8) | 116(68.2) | 61(70.9) | 25(14.7) | 12(13.9) | 10(5.8) | 8(9.3) | 3(1.7) | 0(0.0) |
| MISSION [95] | PP-SES | BMS |  |  |  |  |  |  |  |  |  |  |  |  |
| MULTISTRATEGY [96] | NA-SES | BMS |  |  |  |  |  |  |  |  |  |  |  |  |
| NAPLES-DIABETES [97] | NA-SES | NA-PES |  |  | 14(18.4) | 22(29.3) | 50(65.7) | 40(53.3) | 12(15.8) | 13(17.3) |  |  |  |  |
| NAPLES-DIABETES | NA-SES | NA-EES |  |  | 14(18.4) | 23(30.7) | 50(65.7) | 45(60) | 12(15.8) | 7(9.3) |  |  |  |  |
| NEVO RES-I [98] | PA-SES | PP-PES |  |  |  |  |  |  |  |  |  |  |  |  |
| NEXT [99] | PA-BES | PP-EES |  |  |  |  | 1347(83) | 1366(84) | 188(12) | 180(11) |  |  |  |  |
| NOBORI I [100] | PA-BES | PP-PES |  |  | 32(13.45) | 9(7.2) | 148(62.18) | 83(66.4) | 58(24.37) | 33(26.4) |  |  |  |  |
| NOBORI JAPAN [101] | PA-BES | PP-SES |  |  |  |  |  |  | 32(16.5) | 15(11.4) |  |  |  |  |
| NORSTENT [102] | NA-EES/ZES | BMS |  |  |  |  | 1309(29.1) | 1327(29.4) | 567(12.6) | 538(11.9) | 1404(31.2) | 1438(31.9) | 1201(26.7) | 1171(26) |
| OCT DES [103] | PP-EES | PP-ZES |  |  |  |  |  |  |  |  |  |  |  |  |
| OCTDESI [104] | PP-PES | PP-PES |  |  |  |  |  |  | 11(26.8) | 4(21.1) |  |  |  |  |
| ONYX ONE [105] | PP-ZES | PF-UES | 511(52.8) | 471(50.4) | 88(9.1) | 103(11.0) |  |  | 189(19.5) | 171(18.3) | 262(27.1) | 252(27.0) | 60(6.2) | 48(5.1) |
| ORIENT [106] | PA-SES | PP-ZES |  |  |  |  | 136(53.3) | 70(55.1) | 62(24.3) | 25(19.7) | 33(12.9) | 21(16.5) | 24(9.4) | 11(8.7) |
| ORTALANI [107] | PP-SES | BMS |  |  |  |  | 22(42) | 17(33) | 6(12) | 10(19) | 21(40) | 15(29) | 3(6) | 10(19) |
| Pache et al [108] | PP-SES | BMS |  |  |  |  |  |  | 97(39) | 112(45) |  |  |  |  |
| PAINT [109] | PA-PES | BMS |  |  | 4(3.6) | 5(8.8) | 73(65.8) | 37(64.9) | 27(24.3) | 13(22.8) |  |  |  |  |
| PAINT | PA-SES | BMS |  |  | 6(5.7) | 5(8.8) | 67(63.2) | 37(64.9) | 27(25.5) | 13(22.8) |  |  |  |  |
| PANDA III [110] | PA-SES | PA-SES |  |  | 48(4.1) | 31(2.6) | 182(15.5) | 164(14.0) | 578(49.2) | 613(52.2) | 196(16.7) | 174(14.8) | 170(14.5) | 192(16.4) |
| PASEO [111] | BMS | NA-PES |  |  |  |  |  |  |  |  |  |  |  |  |
| PASEO | BMS | NA-SES |  |  |  |  |  |  |  |  |  |  |  |  |
| PASSION [112] | BMS | NA-PES |  |  |  |  |  |  |  |  |  |  |  |  |
| Petronio et al [113] | PP-PES | PP-SES | 19(45.2) | 22(51.2) |  |  |  |  |  |  |  |  |  |  |
| PIONEER III [114] | PA-SES | PP-EES |  |  | 109(10.0) | 41(7.6) | 536(49.4) | 269(49.6) | 218(20.1) | 114(21.0) | 223(20.5) | 118(21.8) |  |  |
| PLATINUM [115] | PP-EES | PP-EES |  |  |  |  |  |  | 185(24.1) | 188(24.7) |  |  |  |  |
| PLATINUM PLUS [116] | PP-EES | PP-EES |  |  | 315(16.2) | 169(16.5) | 912(46.9) | 478(46.5) |  |  | 549(28.2) | 284(27.7) | 170(8.7) | 96(9.4) |
| Pourmoghaddas [117] | PP-EES | BMS |  |  |  |  |  |  |  |  |  |  |  |  |
| PRAGUE-18 [118] | NA-DES | BMS |  |  |  |  |  |  |  |  |  |  |  |  |
| PRISON II [119] | BMS | PP-SES |  |  |  |  |  |  |  |  |  |  |  |  |
| PRISON III [120] | PP-ZES | PP-SES |  |  |  |  |  |  |  |  |  |  |  |  |
| PRISION IV [121] | PP-EES | PA-SES | 18(10.9) | 17(10.3) |  |  | 115(69.7) | 115(69.7) | 10(6.1) | 12(7.3) |  |  |  |  |
| PRODIGY [122] | BMS | NA-ZES | 380(76) | 363(73) |  |  | 122(24) | 137(27) | 93(19) | 92(18) | 116(23) | 99(20) | 171(34) | 172(34) |
| PRODIGY | BMS | NA-PES | 380(76) | 346(69) |  |  | 122(24) | 154(31) | 93(19) | 83(17) | 116(23) | 120(24) | 171(34) | 143(29) |
| PRODIGY | BMS | NA-EES | 380(76) | 376(75) |  |  | 122(24) | 125(25) | 93(19) | 99(20) | 116(23) | 115(23) | 171(34) | 162(32) |
| PROMISE [123] | PP-EES | PP-PES |  |  | 11(2.6) | 3(0.7) | 177(41.6) | 175(41.2) | 224(52.7) | 233(54.8) | 13(3.1) | 12(2.8) |  |  |
| PROSIT [124] | NA-PES | NA-SES |  |  |  |  |  |  |  |  |  |  |  |  |
| PROTECT [125] | PP-ZES | PP-SES |  |  | 282(6) | 279(6) | 2156(49) | 2101(48) | 796(18) | 842(19) | 767(18) | 746(17.1) | 356(8) | 384(9) |
| RAVEL [126] | BMS | PP-SES |  |  | 13(11) | 13(11) | 49(41) | 44(37) | 58(48) | 61(52) |  |  |  |  |
| REALITY [127] | PP-PES | PP-SES |  |  | 89(13) | 89(13.3) | 400(58.5) | 369(55.2) | 195(28.5) | 211(31.5) |  |  |  |  |
| RECOVERY [128] | PA-SES | PF-SES |  |  |  |  |  |  |  |  |  |  |  |  |
| RECRE8 [129] | PF-AES | PP-ZES | 356(47.9) | 352(47.1) |  |  | 315(42.3) | 318(42.6) | 55(7.4) | 54(7.2) | 133(17.9) | 116(15.5) | 168(22.6) | 182(24.4) |
| REMEDEE [130] | PP-PES | NA-SES |  |  | 13(10.5) | 6(10.2) | 91(73.4) | 43(72.9) |  |  |  |  |  |  |
| RESERVOIR [131] | PF-AES | PP-EES | 25(44.6) | 34(60.7) |  |  |  |  |  |  |  |  |  |  |
| RESET [132] | PP-EES | PP-SES |  |  |  |  |  |  | 180(11) | 213(13) |  |  |  |  |
| RESOLUTE [133] | PP-ZES | PP-EES |  |  |  |  | 382(33.5) | 416(36.1) | 221(19.4) | 218(18.9) |  |  |  |  |
| RESOLUTE CHINA [134] | PP-ZES | PP-PES |  |  |  |  | 17(8.9) | 19(10.1) | 121(63.7) | 135(71.4) |  |  |  |  |
| Sakakibara et al [135] | PP-EES | PP-SES |  |  |  |  |  |  |  |  |  |  |  |  |
| SCANDSTENT [136] | BMS | PP-SES |  |  |  |  |  |  | 41(25) | 41(26) |  |  |  |  |
| SCORPIUS [137] | BMS | PP-SES |  |  |  |  |  |  | 25(27) | 23(24) |  |  |  |  |
| SEA-SIDE [138] | NA-EES | PP-SES | 29(39) | 37(49) |  |  |  |  |  |  |  |  |  |  |
| SELECTION [139] | BMS | PP-PES |  |  |  |  |  |  |  |  |  |  |  |  |
| SENIOR [140] | PA-EES | BMS |  |  | 121(20) | 119(20) | 201(34) | 215(36) | 57(10) | 52(9) | 152(26) | 156(26) | 65(11) | 62(10) |
| Separham et al [141] | PA-BES | PP-EES |  |  |  |  | 24(24) | 34(34) | 28(28) | 29(29) | 6(6) | 9(9) | 42(42) | 28(28) |
| SESAMI [142] | BMS | PP-SES |  |  |  |  | 56(43.4) | 63(49.6) |  |  |  |  | 157(98.1) | 158(98.7) |
| SES-SMART [143] | BMS | PP-SES | 63(48.8) | 46(35.8) | 10(8.0) | 19(14.6) |  |  |  |  |  |  |  |  |
| Shen et al [144] | PA-AT | PP-SES |  |  |  |  | 105(100.0) | 107(100.0) |  |  |  |  |  |  |
| SIRIUS [145] | BMS | PP-SES |  |  |  |  | 314(59) | 309(59) | 282(53) | 283(54) |  |  |  |  |
| SIRTAX [146] | PP-PES | PP-SES | 257(51.1) | 263(51.7) |  |  | 246(48.9) | 246(48.3) | 28(5.6) | 30(5.9) | 112(22.3) | 123(24.2) | 117(23.3) | 110(21.6) |
| SORT OUT II [147] | PP-PES | PP-SES |  |  |  |  | 476(44.7) | 471(45.6) |  |  |  |  | 194(18.2) | 174(16.8) |
| SORT OUT III [148] | PP-ZES | PP-SES |  |  |  |  | 614(53) | 592(51) |  |  |  |  | 70(6) | 100(9) |
| SORT OUT IV [149] | PP-EES | PP-SES |  |  |  |  | 773(55.6) | 754(54.4) |  |  |  |  | 122(8.8) | 145(10.5) |
| SORT OUT V [150] | PA-BES | PP-SES |  |  |  |  | 608(49.5) | 596(48.1) |  |  |  |  | 225(18.3) | 227(18.3) |
| SORT OUT VI [151] | PP-ZES | PA-BES |  |  |  |  | 685(45.6) | 670(44.8) |  |  |  |  | 295(19.6) | 253(16.9) |
| SORT OUT VII [152] | PA-BES | PA-SES |  |  |  |  | 559(44.3) | 555(43.9) |  |  | 388(30.7) | 412(32.6) | 268(21.2) | 262(20.7) |
| SORT OUT VIII [153] | PA-EES | PA-BES |  |  |  |  | 578(42) | 596(43) |  |  |  |  | 287(21) | 284(21) |
| SORT OUT IX [154] | PF-BES | PA-SES |  |  |  |  | 671(42.7) | 645(40.8) |  |  | 454(28.9) | 453(28.7) | 367(23.3) | 397(25.1) |
| SORT OUT X [155] | PA-SES | PA-SES |  |  |  |  | 651(41.3) | 654(41.7) |  |  | 467(29.6) | 499(31.8) | 389(24.7) | 355(22.6) |
| SPIRIT FIRST [156] | PP-EES | BMS |  |  |  |  | 21(78) | 23(79) | 5(19) | 4(14) |  |  |  |  |
| SPIRIT II [157] | PP-EES | PP-PES |  |  |  |  | 138(62) | 47(62) | 60(27) | 24(32) |  |  |  |  |
| SPIRIT III [158] | PP-EES | PP-PES |  |  |  |  |  |  | 123(18.7) | 82(25.1) |  |  |  |  |
| SPIRIT IV [159] | PP-EES | PP-PES |  |  |  |  |  |  | 669(27.7) | 347(28.9) |  |  |  |  |
| SPIRIT V DIABETIC [160] | PP-EES | PP-PES |  |  |  |  | 105(48) | 50(47) | 76(35) | 42(40) |  |  |  |  |
| STRATEGY [161] | BMS | PP-SES |  |  |  |  |  |  |  |  |  |  |  |  |
| TARGET ALL COMERS [162] | PP-EES | PA-SES |  |  | 79(9.5) | 77(9.4) | 383(46.1) | 383(46.7) | 130(15.7) | 105(12.8) | 164(19.8) | 186(22.7) | 74(8.9) | 69(8.4) |
| TARGET I [163] | PP-EES | PA-SES |  |  | 10(4.4) | 9(3.9) | 55(24.2) | 56(24.2) | 162(71.4) | 166(71.9) |  |  |  |  |
| TALENT [164] | PP-EES | PA-SES | 405(56.6) | 429(59.6) |  |  | 310(43.4) | 291(40.4) | 99(13.8) | 116(16.1) | 189(26.4) | 194(26.9) | 117(16.4) | 119(16.5) |
| TAXI [165] | PP-PES | PP-SES |  |  |  |  | 84(82.4) | 86(86) | 18(17.6) | 14(14) |  |  |  |  |
| TAXUS I [166] | BMS | PP-PES |  |  | 11(37) | 7(23) |  |  |  |  |  |  |  |  |
| TAXUS II [167] | BMS | PP-PES |  |  |  |  | 90(67) | 78(58) | 41(30) | 54(40) |  |  |  |  |
| TAXUS IV [168] | BMS | PP-PES |  |  |  |  |  |  | 213(32.7) | 237(35.8) |  |  |  |  |
| TAXUS V [169] | BMS | PP-PES |  |  |  |  |  |  | 173(29.9) | 182(31.5) |  |  |  |  |
| TAXUS VI [170] | BMS | PP-PES |  |  |  |  |  |  | 52(22.9) | 54(24.7) |  |  |  |  |
| TUXEDO [171] | PP-EES | PP-PES | 497(52.3) | 495(54.2) | 66(7.2) | 57(6.2) | 253(27.6) | 268(29.3) |  |  |  |  |  |  |
| TIDE [172] | PF-TiNO | PP-ZES |  |  |  |  | 88(57.9) | 71(47.3) | 14(9.2) | 16(10.7) | 50(32.9) | 63(42.0) |  |  |
| TIDE-ACS [173] | PF-TiNO | PP-EES |  |  |  |  |  |  | 126(12.7) | 61(12.2) | 458(46.3) | 226(45.0) | 444(44.9) | 239(47.6) |
| TWENTE [174] | PP-ZES | PP-EES |  |  |  |  | 335(48.1) | 339(48.8) | 172(24.7) | 153(22.0) | 190(27.3) | 202(29.1) |  |  |
| TWENTE II (DUTCH PEERS) [175] | PP-ZES | PP-EES | 534(59) | 528(58) |  |  | 372(41) | 377(42) | 113(12) | 132(15) | 246(27) | 201(22) | 175(19) | 195(22) |
| TYPHOON [176] | BMS | PP-SES |  |  |  |  |  |  |  |  |  |  |  |  |
| Yin et al [177] | PP-DES | BMS |  |  |  |  |  |  |  |  |  |  |  |  |
| Wessely et al [178] | PA-PES | PA-SES |  |  |  |  |  |  | 16(36) | 22(48) |  |  |  |  |
| X-MAN [179] | PP-EES | PF-CC |  |  |  |  |  |  |  |  |  |  |  |  |
| XAMI [180] | PP-EES | PP-SES |  |  |  |  |  |  |  |  | 17(4.2) | 8(3.6) | 387(95.8) | 213(96.4) |
| ZEST [181] | PP-ZES | PP-SES |  |  | 48(5.4) | 44(5.0) | 348(39.4) | 343(39.1) | 410(46.4) | 424(48.3) | 77(8.7) | 67(7.6) |  |  |
| ZEST | PP-ZES | PP-PES |  |  | 48(5.4) | 56(6.3) | 348(39.4) | 343(38.8) | 410(46.4) | 403(45.6) | 77(8.7) | 82(9.3) |  |  |
| ZEST-AMI [182] | PP-ZES | PP-SES |  |  |  |  |  |  |  |  |  |  |  |  |
| ZEST-AMI | PP-ZES | PP-PES |  |  |  |  |  |  |  |  |  |  |  |  |
| ZEUS [183] | BMS | PP-ZES |  |  |  |  | 295(36.7) | 295(36.8) | 131(16.3) | 139(17.3) | 226(28.1) | 215(26.8) | 152(18.9) | 153(19.1) |
| Zhang Q et al [184] | PP-PES | PP-SES | 104(51) | 145(59) |  |  | 93(46) | 96(39) |  |  |  |  |  |  |
| Zhang L et al [185] | PA-R | PF-PES |  |  |  |  | 42(49.41) | 43(52.44) | 23(27.06) | 22(26.83) | 2(2.35) | 4(4.88) | 18(21.18) | 12(14.63) |
| Zhang Y et al [186] | PF-PES | PA-SES |  |  |  |  | 55(16.82) | 85(24.93) | 224(68.50) | 175(51.32) | 19(5.81) | 4(1.17) | 62(18.96) | 58(17.01) |
| Zhang Y et al | PF-PES | PP-SES |  |  |  |  | 55(16.82) | 39(12.15) | 224(68.50) | 230(71.65) | 19(5.81) | 16(4.98) | 62(18.96) | 78(24.30) |
| ZOMAXX I [187] | PP-ZES | PP-PES |  |  |  |  |  |  |  |  |  |  |  |  |
| ZOMAXX II [188] | PP-ZES | Paclitaxel |  |  |  |  |  |  |  |  |  |  |  |  |
| XIMA [189] | PP-EES | BMS |  |  |  |  |  |  |  |  |  |  |  |  |
| Xu et al [190] | PP-ZES | PA-SES |  |  |  |  |  |  | 110(70.5) | 135(80.4) |  |  |  |  |
| TINOX [191] | PF-TiNO | BMS |  |  |  |  |  |  |  |  |  |  |  |  |
| TITAX AMI [192] | PF-TiNO | PP-PES |  |  |  |  |  |  |  |  | 131(61) | 114(54) | 83(39) | 97(46) |
| BASE ACS [193] | PF-TiNO | PP-EES |  |  |  |  |  |  | 49(11.8) | 64(15.6) | 206(49.4) | 187(45.6) | 162(38.8) | 159(38.8) |
| TITANIC XV [194] | PF-TiNO | PP-EES |  |  |  |  |  |  |  |  |  |  |  |  |
| CIBELES [195] | PP-EES | PP-SES |  |  |  |  |  |  |  |  |  |  |  |  |
| PIONEER [196] | PP-ZES | PA-SES |  |  | 20(23.0) | 21(25.3) | 52(59.8) | 42(50.6) | 15(17.2) | 20(24.1) |  |  |  |  |

**Supplemental Table 5: Net-league estimates of MACE and Mortality over 6 months across different treatment strategies showing risk ratio with its 95% confidence interval**

**Mortality 6 Months**

| **BMS** | 1.37 (0.35; 5.39) | 1.30 (0.84; 2.02) | 1.69 (0.45; 6.36) | - | 2.00 (0.18; 21.95) | 1.54 (0.49; 4.82) | 1.06 (0.27; 4.22) | 1.48 (0.51; 4.32) | 1.31 (0.32; 5.47) |
| --- | --- | --- | --- | --- | --- | --- | --- | --- | --- |
| - | **PA-BES** | 0.95 (0.23; 3.95) | 1.23 (0.37; 4.14) | - | 1.46 (0.09; 22.99) | 1.12 (0.38; 3.30) | 0.77 (0.18; 3.33) | 1.08 (0.46; 2.55) | 0.96 (0.46; 2.00) |
| 1.52 (0.97; 2.38) | - | **PA-EES** | 1.30 (0.33; 5.16) | - | 1.53 (0.13; 17.50) | 1.18 (0.35; 3.93) | 0.81 (0.19; 3.43) | 1.14 (0.37; 3.55) | 1.01 (0.23; 4.42) |
| 2.24 (1.46; 3.43) | - | 1.47 (0.81; 2.70) | **PA- SES** | - | 1.18 (0.08; 18.23) | 0.91 (0.33; 2.53) | 0.62 (0.17; 2.34) | 0.88 (0.37; 2.06) | 0.78 (0.22; 2.78) |
| 2.80 (0.12; 67.00) | - | 1.85 (0.07; 45.57) | 1.25 (0.05; 30.79) | **PF-TiNO** | **-** | - | - | - | - |
| - | - | - | - | **-** | **PP- DES** | 0.77 (0.05; 10.97) | 0.53 (0.03; 8.44) | 0.74 (0.05; 10.26) | 0.66 (0.04; 10.72) |
| 1.49 (0.58; 3.80) | - | 0.98 (0.39; 2.48) | 0.67 (0.26; 1.71) | 0.53 (0.02; 14.52) | - | **PP- EES** | 0.69 (0.18; 2.57) | 0.97 (0.50; 1.86) | 0.85 (0.27; 2.70) |
| 1.75 (1.31; 2.34) | - | 1.15 (0.68; 1.96) | 0.78 (0.50; 1.21) | 0.62 (0.03; 15.09) | - | 1.17 (0.45; 3.08) | **PP- PES** | 1.40 (0.43; 4.60) | 1.24 (0.27; 5.67) |
| 1.95 (1.37; 2.77) | - | 1.28 (0.73; 2.25) | 0.87 (0.65; 1.17) | 0.70 (0.03; 16.94) | - | 1.31 (0.51; 3.36) | 1.12 (0.76; 1.64) | **PP- SES** | 0.88 (0.34; 2.28) |
| 1.62 (0.73; 3.61) | - | 1.07 (0.43; 2.63) | 0.72 (0.33; 1.58) | 0.58 (0.02; 15.24) | - | 1.09 (0.36; 3.32) | 0.93 (0.41; 2.10) | 0.83 (0.40; 1.72) | **PP- ZES** |

**MACE 6 months**

**Supplemental Table 6: Net-league estimates of MI and ST over 6 months across different treatment strategies showing risk ratio with its 95% confidence interval**

**ST 6 Months**

| **BMS** | 0.44 (0.07; 2.84) | 2.91 (1.09; 7.78) | 4.52 (0.40; 51.06) | 0.98 (0.03; 32.51) | 4.96 (0.58; 42.32) | 2.91 (0.68; 12.41) | 1.51 (0.36; 6.26) | 0.98 (0.25; 3.77) | 0.35 (0.07; 1.91) |
| --- | --- | --- | --- | --- | --- | --- | --- | --- | --- |
| 0.74 (0.25; 2.19) | **PA-BES** | 6.64 (0.84; 52.45) | 10.3 (0.84; 126.88) | 2.24 (0.06; 79.40) | 11.31(0.66; 194.35) | 6.63 (1.32; 33.17) | 3.44 (0.74; 16.06) | 2.23 (0.61; 8.11) | 0.80 (0.20; 3.31) |
| 1.09 (0.64; 1.84) | 1.47 (0.44; 4.90) | **PA-EES** | 1.55 (0.12; 20.34) | 0.34 (0.01; 12.32) | 1.70 (0.16; 18.00) | 1.00 (0.19; 5.33) | 0.52 (0.10; 2.78) | 0.34 (0.07; 1.68) | 0.12 (0.02; 0.81) |
| 2.02 (1.09; 3.76) | 2.74 (1.00; 7.49) | 1.86 (0.83; 4.16) | **PA- SES** | 0.22 (0.00; 9.95) | 1.10 (0.04; 27.96) | 0.64 (0.08; 5.35) | 0.33 (0.04; 2.86) | 0.22 (0.03; 1.86) | 0.08 (0.01; 0.84) |
| - | - | - | - | **PF- Cobalt Chromium** | 5.05 (0.08; 306.29) | 2.96 (0.12; 71.55) | 1.54 (0.05; 46.43) | 1.00 (0.04; 27.74) | 0.36 (0.01; 11.64) |
| 2.99 (0.31; 28.63) | 4.05 (0.33; 49.72) | 2.75 (0.27; 28.03) | 1.48 (0.14; 15.39) | **-** | **PP- DES** | 0.59 (0.04; 7.80) | 0.30 (0.02; 3.98) | 0.20 (0.02; 2.48) | 0.07 (0.00; 1.09) |
| 1.99 (0.87; 4.55) | 2.70 (0.88; 8.30) | 1.83 (0.70; 4.80) | 0.98 (0.50; 1.96) | **-** | 0.67 (0.06; 7.39) | **PP- EES** | 0.52 (0.15; 1.75) | 0.34 (0.13; 0.88) | 0.12 (0.03; 0.49) |
| 1.39 (0.89; 2.19) | 1.89 (0.65; 5.51) | 1.28 (0.64; 2.56) | 0.69 (0.39; 1.23) | **-** | 0.47 (0.05; 4.68) | 0.70 (0.31; 1.58) | **PP- PES** | 0.65 (0.28; 1.50) | 0.23 (0.06; 0.87) |
| 1.73 (1.01; 2.97) | 2.35 (0.91; 6.05) | 1.60 (0.76; 3.36) | 0.86 (0.60; 1.22) | **-** | 0.58 (0.06; 5.92) | 0.87 (0.45; 1.70) | 1.24 (0.75; 2.05) | **PP- SES** | 0.36 (0.13; 0.99) |
| 0.71 (0.26; 1.98) | 0.97 (0.43; 2.17) | 0.66 (0.21; 2.06) | 0.35 (0.14; 0.90) | **-** | 0.24 (0.02; 2.85) | 0.36 (0.13; 1.01) | 0.51 (0.19; 1.40) | 0.41 (0.17; 0.99) | **PP- ZES** |

**MI 6 months**

**Supplemental Table 7: Net-league estimates of TLR and TVR over 6 months across different treatment strategies showing risk ratio with its 95% confidence interval**

**TVR 6 months**

| **BMS** | 1.45 (0.65; 3.23) | 4.23 (0.89; 19.99) | 2.49 (1.26; 4.91) | 1.99 (0.50; 7.91) | 2.27 (1.07; 4.82) | 1.91 (1.35; 2.72) | 2.04 (1.34; 3.09) | 1.26 (0.55; 2.85) |
| --- | --- | --- | --- | --- | --- | --- | --- | --- |
| 1.71 (0.67; 4.38) | **PA-BES** | 2.92 (0.56; 15.22) | 1.72 (0.69; 4.30) | 1.37 (0.28; 6.79) | 1.57 (0.61; 4.00) | 1.32 (0.59; 2.97) | 1.41 (0.71; 2.79) | 0.87 (0.49; 1.54) |
| 3.05 (1.48; 6.31) | 1.79 (0.56; 5.70) | **PA-EES** | 0.59 (0.12; 2.94) | 0.47 (0.06; 3.76) | 0.54 (0.14; 2.08) | 0.45 (0.10; 2.14) | 0.48 (0.11; 2.16) | 0.30 (0.06; 1.56) |
| 4.30 (1.86; 9.94) | 2.51 (0.94; 6.72) | 1.41 (0.48; 4.15) | **PA- SES** | 0.80 (0.17; 3.72) | 0.91 (0.39; 2.14) | 0.77 (0.40; 1.47) | 0.82 (0.45; 1.50) | 0.50 (0.20; 1.28) |
| 1.33 (0.30; 5.92) | 0.78 (0.13; 4.54) | 0.44 (0.08; 2.29) | 0.31 (0.06; 1.72) | **PP- DES** | 1.14 (0.24; 5.50) | 0.96 (0.23; 4.00) | 1.02 (0.24; 4.33) | 0.63 (0.13; 3.14) |
| 2.68 (1.06; 6.75) | 1.57 (0.54; 4.52) | 0.88 (0.29; 2.63) | 0.62 (0.23; 1.67) | 2.01 (0.35; 11.64) | **PP- EES** | 0.84 (0.40; 1.80) | 0.90 (0.47; 1.70) | 0.55 (0.21; 1.43) |
| 2.48 (1.56; 3.93) | 1.45 (0.59; 3.56) | 0.81 (0.35; 1.89) | 0.58 (0.27; 1.25) | 1.86 (0.39; 8.87) | 0.93 (0.37; 2.29) | **PP- PES** | 1.06 (0.70; 1.63) | 0.66 (0.29; 1.49) |
| 2.87 (1.57; 5.25) | 1.68 (0.82; 3.45) | 0.94 (0.38; 2.33) | 0.67 (0.34; 1.30) | 2.16 (0.43; 10.79) | 1.07 (0.49; 2.34) | 1.16 (0.68; 1.99) | **PP- SES** | 0.62 (0.31; 1.25) |
| 1.77 (0.70; 4.45) | 1.03 (0.56; 1.91) | 0.58 (0.18; 1.82) | 0.41 (0.16; 1.08) | 1.33 (0.23; 7.67) | 0.66 (0.23; 1.88) | 0.71 (0.30; 1.72) | 0.62 (0.31; 1.24) | **PP- ZES** |

**TLR 6 months**

**Supplemental Table 8: Net-league estimates of CVD over 6 months across different treatment strategies showing risk ratio with its 95% confidence interval**

| **BMS** |  |  |  |  |  |  |  |
| --- | --- | --- | --- | --- | --- | --- | --- |
| 1.69 (0.45; 6.31) | **PA-BES** |  |  |  |  |  |  |
| 1.73 (1.04; 2.88) | 1.02 (0.25; 4.17) | **PA-EES** |  |  |  |  |  |
| 2.12 (0.46; 9.74) | 1.26 (0.31; 5.05) | 1.23 (0.25; 6.06) | **PA- SES** |  |  |  |  |
| 2.21 (0.69; 7.02) | 1.31 (0.46; 3.75) | 1.28 (0.37; 4.45) | 1.04 (0.30; 3.63) | **PP- EES** |  |  |  |
| 0.96 (0.36; 2.55) | 0.57 (0.12; 2.62) | 0.56 (0.18; 1.67) | 0.45 (0.08; 2.49) | 0.43 (0.11; 1.76) | **PP- PES** |  |  |
| 1.84 (0.65; 5.20) | 1.09 (0.48; 2.46) | 1.06 (0.34; 3.34) | 0.87 (0.28; 2.67) | 0.83 (0.43; 1.62) | 1.92 (0.53; 6.98) | **PP- SES** |  |
| 1.61 (0.47; 5.47) | 0.95 (0.45; 2.04) | 0.93 (0.25; 3.47) | 0.76 (0.21; 2.78) | 0.73 (0.29; 1.85) | 1.67 (0.39; 7.12) | 0.87 (0.46; 1.67) | **PP- ZES** |

**CVD 6 Months**

**Supplemental Table 9: Net-league estimates of MACE and Mortality over 1-Year across different treatment strategies showing risk ratio with its 95% confidence interval**

**Mortality 1 Year**

| **BMS** | 1.07 (0.85; 1.34) | 1.11 (0.83; 1.48) | 1.28 (0.46; 3.59) | 0.95 (0.75; 1.19) | 1.12 (0.59; 2.12) | 1.30 (0.78; 2.17) | 1.33 (0.88; 2.00) | 1.32 (0.77; 2.25) | 1.19 (0.83; 1.70) | 1.04 (0.86; 1.26) | 0.97 (0.77; 1.21) | 0.92 (0.74; 1.14) | 1.01 (0.84; 1.23) |
| --- | --- | --- | --- | --- | --- | --- | --- | --- | --- | --- | --- | --- | --- |
| 1.60 (1.41; 1.81) | **PA- BES** | 1.04 (0.78; 1.39) | 1.19 (0.42; 3.43) | 0.89 (0.70; 1.12) | 1.04 (0.54; 2.00) | 1.22 (0.73; 2.03) | 1.24 (0.81; 1.90) | 1.23 (0.72; 2.12) | 1.11 (0.76; 1.61) | 0.98 (0.79; 1.20) | 0.90 (0.69; 1.18) | 0.86 (0.68; 1.08) | 0.95 (0.76; 1.19) |
| 1.77 (1.49; 2.12) | 1.11 (0.94; 1.31) | **PA- EES** | 1.15 (0.39; 3.36) | 0.85 (0.63; 1.16) | 1.00 (0.51; 1.99) | 1.17 (0.68; 2.03) | 1.20 (0.74; 1.93) | 1.19 (0.66; 2.12) | 1.07 (0.69; 1.65) | 0.94 (0.70; 1.26) | 0.87 (0.62; 1.22) | 0.83 (0.60; 1.14) | 0.91 (0.67; 1.24) |
| 1.46 (1.01; 2.10) | 0.91 (0.62; 1.34) | 0.82 (0.55; 1.23) | **PA-PES** | 0.74 (0.26; 2.14) | 0.87 (0.26; 2.94) | 1.02 (0.32; 3.22) | 1.04 (0.34; 3.16) | 1.03 (0.32; 3.29) | 0.93 (0.31; 2.77) | 0.82 (0.29; 2.33) | 0.76 (0.26; 2.18) | 0.72 (0.25; 2.06) | 0.79 (0.28; 2.27) |
| 1.84 (1.63; 2.07) | 1.15 (1.02; 1.29) | 1.04 (0.87; 1.23) | 1.26 (0.86; 1.85) | **PA- SES** | 1.18 (0.61; 2.25) | 1.37 (0.87; 2.16) | 1.40 (0.92; 2.14) | 1.39 (0.81; 2.37) | 1.25 (0.86; 1.82) | 1.10 (0.91; 1.33) | 1.02 (0.78; 1.33) | 0.97 (0.76; 1.24) | 1.07 (0.86; 1.33) |
| 1.48 (0.99; 2.21) | 0.93 (0.62; 1.38) | 0.84 (0.55; 1.27) | 1.02 (0.59; 1.75) | 0.81 (0.54; 1.20) | **PF- AES** | 1.17 (0.53; 2.58) | 1.19 (0.58; 2.43) | 1.18 (0.53; 2.66) | 1.06 (0.54; 2.11) | 0.94 (0.49; 1.77) | 0.87 (0.45; 1.66) | 0.82 (0.43; 1.58) | 0.91 (0.49; 1.68) |
| 1.78 (1.36; 2.32) | 1.11 (0.85; 1.44) | 1.00 (0.75; 1.34) | 1.22 (0.78; 1.92) | 0.97 (0.76; 1.23) | 1.20 (0.75; 1.91) | **PF- BES** | 1.02 (0.55; 1.91) | 1.01 (0.50; 2.05) | 0.91 (0.51; 1.65) | 0.80 (0.49; 1.31) | 0.74 (0.44; 1.26) | 0.71 (0.42; 1.19) | 0.78 (0.47; 1.30) |
| 1.68 (1.39; 2.02) | 1.05 (0.87; 1.27) | 0.95 (0.75; 1.19) | 1.15 (0.76; 1.74) | 0.91 (0.76; 1.10) | 1.13 (0.75; 1.72) | 0.94 (0.70; 1.28) | **PF- SES + Probucol** | 0.99 (0.52; 1.89) | 0.89 (0.56; 1.43) | 0.78 (0.52; 1.18) | 0.73 (0.47; 1.12) | 0.69 (0.45; 1.06) | 0.76 (0.53; 1.09) |
| 1.90 (1.50; 2.41) | 1.19 (0.94; 1.51) | 1.07 (0.82; 1.41) | 1.31 (0.84; 2.02) | 1.04 (0.82; 1.31) | 1.28 (0.82; 2.00) | 1.07 (0.77; 1.49) | 1.13 (0.86; 1.49) | **PF- TiNO** | 0.90 (0.49; 1.66) | 0.79 (0.48; 1.31) | 0.73 (0.43; 1.25) | 0.70 (0.41; 1.20) | 0.77 (0.45; 1.31) |
| 1.53 (1.25; 1.87) | 0.96 (0.78; 1.17) | 0.86 (0.68; 1.10) | 1.05 (0.69; 1.59) | 0.83 (0.68; 1.02) | 1.03 (0.68; 1.58) | 0.86 (0.63; 1.17) | 0.91 (0.73; 1.15) | 0.80 (0.61; 1.07) | **PF- UES** | 0.88 (0.62; 1.25) | 0.82 (0.55; 1.20) | 0.77 (0.53; 1.13) | 0.85 (0.63; 1.15) |
| 1.78 (1.60; 1.97) | 1.11 (1.01; 1.23) | 1.00 (0.85; 1.18) | 1.22 (0.83; 1.78) | 0.97 (0.89; 1.05) | 1.20 (0.81; 1.78) | 1.00 (0.78; 1.29) | 1.06 (0.89; 1.26) | 0.93 (0.75; 1.17) | 1.16 (0.96; 1.40) | **PP- EES** | 0.93 (0.75; 1.15) | 0.88 (0.72; 1.08) | 0.97 (0.80; 1.18) |
| 1.26 (1.13; 1.41) | 0.79 (0.70; 0.89) | 0.71 (0.59; 0.85) | 0.87 (0.59; 1.27) | 0.69 (0.61; 0.78) | 0.85 (0.57; 1.27) | 0.71 (0.54; 0.93) | 0.75 (0.62; 0.91) | 0.66 (0.52; 0.84) | 0.82 (0.68; 1.01) | 0.71 (0.64; 0.78) | **PP- PES** | 0.95 (0.75; 1.20) | 1.05 (0.82; 1.34) |
| 1.70 (1.53; 1.89) | 1.06 (0.95; 1.19) | 0.96 (0.80; 1.14) | 1.17 (0.80; 1.71) | 0.93 (0.83; 1.04) | 1.15 (0.77; 1.71) | 0.96 (0.74; 1.24) | 1.01 (0.84; 1.22) | 0.89 (0.71; 1.13) | 1.11 (0.91; 1.35) | 0.96 (0.87; 1.05) | 1.35 (1.21; 1.50) | **PP- SES** | 1.10 (0.88; 1.39) |
| 1.66 (1.49; 1.85) | 1.03 (0.92; 1.16) | 0.93 (0.78; 1.11) | 1.14 (0.78; 1.66) | 0.90 (0.81; 1.00) | 1.12 (0.76; 1.65) | 0.93 (0.72; 1.21) | 0.99 (0.85; 1.15) | 0.87 (0.69; 1.09) | 1.08 (0.91; 1.28) | 0.93 (0.85; 1.02) | 1.31 (1.18; 1.46) | 0.97 (0.88; 1.08) | **PP- ZES** |

**MACE 1 Year**

**Supplemental Table 10: Net-league estimates of Myocardial Infarction over 1-Year across different treatment strategies showing risk ratio with its 95% confidence interval**

| **BMS** |  |  |  |  |  |  |  |  |  |  |  |  |  |
| --- | --- | --- | --- | --- | --- | --- | --- | --- | --- | --- | --- | --- | --- |
| 1.47 (1.17; 1.85) | **PA-BES** |  |  |  |  |  |  |  |  |  |  |  |  |
| 1.81 (1.35; 2.41) | 1.23 (0.94; 1.62) | **PA-EES** |  |  |  |  |  |  |  |  |  |  |  |
| 1.85 (1.49; 2.30) | 1.26 (1.03; 1.54) | 1.02 (0.78; 1.34) | **PA- SES** |  |  |  |  |  |  |  |  |  |  |
| 1.57 (0.91; 2.71) | 1.06 (0.62; 1.84) | 0.87 (0.48; 1.55) | 0.85 (0.49; 1.45) | **PF- AES** |  |  |  |  |  |  |  |  |  |
| 1.99 (1.21; 3.25) | 1.35 (0.83; 2.20) | 1.10 (0.66; 1.85) | 1.07 (0.69; 1.67) | 1.27 (0.63; 2.55) | **PF- BES** |  |  |  |  |  |  |  |  |
| 1.73 (1.13; 2.66) | 1.18 (0.77; 1.81) | 0.96 (0.60; 1.53) | 0.94 (0.62; 1.42) | 1.11 (0.58; 2.09) | 0.87 (0.47; 1.60) | **PF-SES + Probucol** |  |  |  |  |  |  |  |
| 3.70 (2.44; 5.63) | 2.52 (1.67; 3.81) | 2.05 (1.30; 3.23) | 2.00 (1.34; 2.99) | 2.37 (1.24; 4.50) | 1.86 (1.03; 3.38) | 2.14 (1.24; 3.69) | **PF- TiNO** |  |  |  |  |  |  |
| 1.64 (1.22; 2.20) | 1.11 (0.83; 1.50) | 0.91 (0.64; 1.29) | 0.88 (0.67; 1.17) | 1.04 (0.60; 1.83) | 0.82 (0.49; 1.39) | 0.94 (0.61; 1.47) | 0.44 (0.28; 0.69) | **PF- UES** |  |  |  |  |  |
| 1.91 (1.26; 2.88) | 1.30 (0.86; 1.96) | 1.06 (0.68; 1.65) | 1.03 (0.72; 1.48) | 1.22 (0.64; 2.32) | 0.96 (0.54; 1.70) | 1.10 (0.64; 1.91) | 0.51 (0.30; 0.88) | 1.17 (0.74; 1.84) | **PP- DES** |  |  |  |  |
| 1.79 (1.48; 2.17) | 1.22 (1.02; 1.45) | 0.99 (0.77; 1.28) | 0.97 (0.84; 1.11) | 1.14 (0.68; 1.94) | 0.90 (0.57; 1.43) | 1.03 (0.69; 1.55) | 0.48 (0.33; 0.70) | 1.10 (0.85; 1.42) | 0.94 (0.64; 1.38) | **PP- EES** |  |  |  |
| 1.18 (0.96; 1.45) | 0.80 (0.64; 1.00) | 0.65 (0.49; 0.87) | 0.64 (0.52; 0.78) | 0.75 (0.44; 1.29) | 0.59 (0.36; 0.96) | 0.68 (0.45; 1.04) | 0.32 (0.21; 0.48) | 0.72 (0.54; 0.96) | 0.62 (0.41; 0.93) | 0.66 (0.56; 0.78) | **PP- PES** |  |  |
| 1.52 (1.25; 1.86)) | 1.04 (0.85; 1.27) | 0.84 (0.64; 1.12) | 0.82 (0.68; 0.99) | 0.97 (0.57; 1.67) | 0.77 (0.47; 1.24) | 0.88 (0.58; 1.34) | 0.41 (0.27; 0.62) | 0.93 (0.70; 1.23) | 0.80 (0.54; 1.19) | 0.85 (0.72; 1.00) | 1.29 (1.08; 1.55) | **PP- SES** |  |
| 1.77 (1.46; 2.16) | 1.21 (0.99; 1.47) | 0.98 (0.74; 1.29) | 0.96 (0.81; 1.14) | 1.13 (0.68; 1.89) | 0.89 (0.56; 1.43) | 1.02 (0.70; 1.50) | 0.48 (0.32; 0.71) | 1.08 (0.87; 1.35) | 0.93 (0.63; 1.38) | 0.99 (0.86; 1.13) | 1.50 (1.26; 1.79) | 1.16 (0.98; 1.38) | **PP- ZES** |

**MI 1-Year**

**Supplemental Table 11: Net-league estimates of TLR and TVR over 1-Year across different treatment strategies showing risk ratio with its 95% confidence interval**

**TVR 1 Year**

| **BMS** | 2.11 (1.76; 2.51) | 2.32 (1.71; 3.13) | 1.93 (1.22; 3.06) | 2.35 (1.98; 2.79) | 2.06 (0.79; 5.35) | 1.74 (1.19; 2.55) | 2.04 (1.60; 2.60) | 1.66 (1.23; 2.24) | 1.40 (0.89; 2.18) | 2.30 (1.81; 2.92) | 2.28 (1.97; 2.65) | 1.53 (1.31; 1.79) | 2.25 (1.95; 2.60) | 1.99 (1.70; 2.32) |
| --- | --- | --- | --- | --- | --- | --- | --- | --- | --- | --- | --- | --- | --- | --- |
| 2.41 (1.96; 2.97) | **PA-BES** | 1.10 (0.84; 1.44) | 0.92 (0.56; 1.50) | 1.11 (0.94; 1.32) | 0.98 (0.38; 2.54) | 0.83 (0.57; 1.21) | 0.97 (0.76; 1.24) | 0.79 (0.58; 1.06) | 0.66 (0.42; 1.04) | 1.09 (0.86; 1.39) | 1.08 (0.94; 1.25) | 0.73 (0.60; 0.87) | 1.07 (0.91; 1.25) | 0.94 (0.80; 1.11) |
| 2.54 (1.83; 3.53) | 1.05 (0.77; 1.45) | **PA-EES** | 0.83 (0.48; 1.44) | 1.01 (0.76; 1.35) | 0.89 (0.33; 2.38) | 0.75 (0.48; 1.17) | 0.88 (0.62; 1.25) | 0.72 (0.49; 1.05) | 0.60 (0.36; 1.01) | 0.99 (0.71; 1.39) | 0.99 (0.75; 1.30) | 0.66 (0.49; 0.89) | 0.97 (0.73; 1.30) | 0.86 (0.64; 1.15) |
| 2.09 (1.28; 3.41) | 0.87 (0.51; 1.48) | 0.82 (0.46; 1.49) | **PA- PES** | 1.22 (0.75; 1.99) | 1.07 (0.37; 3.08) | 0.90 (0.50; 1.64) | 1.06 (0.63; 1.78) | 0.86 (0.50; 1.49) | 0.72 (0.38; 1.37) | 1.19 (0.71; 2.00) | 1.18 (0.73; 1.92) | 0.79 (0.49; 1.29) | 1.17 (0.72; 1.89) | 1.03 (0.63; 1.67) |
| 2.50 (2.05; 3.04) | 1.04 (0.85; 1.27) | 0.98 (0.71; 1.36) | 1.20 (0.71; 2.02) | **PA- SES** | 0.88 (0.34; 2.28) | 0.74 (0.53; 1.04) | 0.87 (0.68; 1.11) | 0.71 (0.53; 0.95) | 0.59 (0.38; 0.93) | 0.98 (0.81; 1.18) | 0.97 (0.86; 1.11) | 0.65 (0.55; 0.78) | 0.96 (0.81; 1.13) | 0.85 (0.72; 0.99) |
| 2.13 (1.29; 3.51) | 0.88 (0.53; 1.47) | 0.84 (0.47; 1.49) | 1.02 (0.51; 2.05) | 0.85 (0.51; 1.41) | **PF- AES** | 0.85 (0.31; 2.33) | 0.99 (0.38; 2.62) | 0.81 (0.30; 2.15) | 0.68 (0.24; 1.92) | 1.12 (0.42; 2.95) | 1.11 (0.43; 2.86) | 0.74 (0.29; 1.91) | 1.09 (0.42; 2.83) | 0.96 (0.37; 2.50) |
| 0.92 (0.54; 1.59) | 0.38 (0.22; 0.66) | 0.36 (0.20; 0.67) | 0.44 (0.21; 0.92) | 0.37 (0.22; 0.61) | 0.43 (0.21; 0.89) | **PF- BES** | 1.17 (0.77; 1.78) | 0.95 (0.61; 1.49) | 0.80 (0.46; 1.40) | 1.32 (0.90; 1.94) | 1.31 (0.91; 1.88) | 0.88 (0.60; 1.28) | 1.29 (0.89; 1.88) | 1.14 (0.78; 1.66) |
| 2.03 (1.52; 2.70) | 0.84 (0.63; 1.13) | 0.80 (0.53; 1.19) | 0.97 (0.55; 1.71) | 0.81 (0.61; 1.09) | 0.95 (0.56; 1.62) | 2.19 (1.22; 3.94) | **PF-SES + Probucol** | 0.81 (0.58; 1.14) | 0.68 (0.43; 1.08) | 1.13 (0.83; 1.53) | 1.12 (0.89; 1.41) | 0.75 (0.59; 0.95) | 1.10 (0.87; 1.40) | 0.97 (0.81; 1.17) |
| 1.75 (1.24; 2.46) | 0.72 (0.51; 1.02) | 0.69 (0.44; 1.06) | 0.83 (0.46; 1.52) | 0.70 (0.50; 0.98) | 0.82 (0.46; 1.45) | 1.89 (1.03; 3.47) | 0.86 (0.58; 1.28) | **PF- TiNO** | 0.84 (0.51; 1.39) | 1.39 (0.98; 1.96) | 1.38 (1.05; 1.80) | 0.92 (0.68; 1.24) | 1.36 (1.01; 1.82) | 1.20 (0.90; 1.59) |
| 1.44 (0.87; 2.40) | 0.60 (0.36; 1.00) | 0.57 (0.32; 1.02) | 0.69 (0.34; 1.40) | 0.58 (0.35; 0.96) | 0.68 (0.34; 1.34) | 1.56 (0.76; 3.21) | 0.71 (0.42; 1.21) | 0.83 (0.47; 1.47) | **PF- UES** | 1.65 (1.02; 2.67) | 1.64 (1.05; 2.54) | 1.09 (0.70; 1.71) | 1.61 (1.03; 2.52) | 1.42 (0.94; 2.16) |
| 1.88 (1.45; 2.46) | 0.78 (0.59; 1.04) | 0.74 (0.51; 1.09) | 0.90 (0.52; 1.57) | 0.75 (0.60; 0.94) | 0.89 (0.51; 1.52) | 2.04 (1.17; 3.55) | 0.93 (0.65; 1.32) | 1.08 (0.73; 1.60) | 1.30 (0.75; 2.26) | **PP- DES** | 0.99 (0.80; 1.24) | 0.66 (0.52; 0.85) | 0.98 (0.77; 1.24) | 0.86 (0.68; 1.10) |
| 2.66 (2.24; 3.17) | 1.10 (0.93; 1.31) | 1.05 (0.76; 1.44) | 1.27 (0.76; 2.14) | 1.07 (0.91; 1.24) | 1.25 (0.77; 2.04) | 2.88 (1.69; 4.89) | 1.31 (1.00; 1.73) | 1.53 (1.12; 2.07) | 1.84 (1.12; 3.04) | 1.41 (1.09; 1.83) | **PP- EES** | 0.67 (0.58; 0.77) | 0.99 (0.86; 1.13) | 0.87 (0.76; 1.00) |
| 1.71 (1.44; 2.03) | 0.71 (0.58; 0.87) | 0.67 (0.48; 0.94) | 0.82 (0.49; 1.37) | 0.68 (0.56; 0.83) | 0.80 (0.49; 1.31) | 1.84 (1.07; 3.18) | 0.84 (0.63; 1.11) | 0.98 (0.70; 1.37) | 1.18 (0.71; 1.96) | 0.91 (0.69; 1.20) | 0.64 (0.55; 0.75) | **PP- PES** | 1.47 (1.26; 1.72) | 1.30 (1.11; 1.52) |
| 2.79 (2.35; 3.32) | 1.16 (0.97; 1.39) | 1.10 (0.79; 1.53) | 1.34 (0.79; 2.24) | 1.12 (0.93; 1.35) | 1.31 (0.80; 2.15) | 3.02 (1.76; 5.18) | 1.38 (1.04; 1.82) | 1.60 (1.15; 2.24) | 1.93 (1.17; 3.20) | 1.48 (1.13; 1.95) | 1.05 (0.90; 1.22) | 1.64 (1.40; 1.91) | **PP- SES** | 0.88 (0.75; 1.03) |
| 2.03 (1.71; 2.41) | 0.84 (0.70; 1.02) | 0.80 (0.57; 1.11) | 0.97 (0.58; 1.63) | 0.81 (0.67; 0.97) | 0.95 (0.59; 1.54) | 2.19 (1.28; 3.76) | 1.00 (0.79; 1.26) | 1.16 (0.84; 1.60) | 1.40 (0.87; 2.26) | 1.08 (0.82; 1.41) | 0.76 (0.66; 0.88) | 1.19 (1.01; 1.40) | 0.73 (0.62; 0.85) | **PP- ZES** |

**TLR 1 Year**

**Supplemental Table 12: Net-league estimates of CVD and Stroke over 1 year across different treatment strategies showing risk ratio with its 95% confidence interval**

**Stroke 1 Year**

| **BMS** | 0.94 (0.45; 1.97) | 0.42 (0.15; 1.17) | 2.99 (0.12; 72.88) | 0.94 (0.37; 2.39) | 1.47 (0.41; 5.32) | - | - | - | 1.44 (0.61; 3.37) | 0.96 (0.51; 1.83) | 0.76 (0.36; 1.62) | 1.39 (0.71; 2.71) | 1.46 (0.79; 2.72) |
| --- | --- | --- | --- | --- | --- | --- | --- | --- | --- | --- | --- | --- | --- |
| 1.09 (0.81; 1.45) | **PA-BES** | 0.44 (0.12; 1.59) | 3.19 (0.12; 84.81) | 1.01 (0.42; 2.41) | 1.57 (0.37; 6.69) | - | - | - | 1.53 (0.52; 4.53) | 1.03 (0.61; 1.75) | 0.81 (0.32; 2.08) | 1.48 (0.71; 3.08) | 1.56 (0.63; 3.89) |
| 1.30 (0.90; 1.88) | 1.20 (0.81; 1.77) | **PA-EES** | 7.18 (0.25; 206.46) | 2.27 (0.56; 9.12) | 3.53 (0.68; 18.44) | - | - | - | 3.45 (0.90; 13.21) | 2.32 (0.68; 7.84) | 1.83 (0.51; 6.59) | 3.34 (0.97; 11.46) | 3.52 (1.05; 11.78) |
| 0.93 (0.26; 3.30) | 0.86 (0.24; 3.14) | 0.72 (0.19; 2.68) | **PA- PES** | 0.32 (0.01; 8.80) | 0.49 (0.02; 15.41) | - | - | - | 0.48 (0.02; 13.13) | 0.32 (0.01; 8.39) | 0.25 (0.01; 6.78) | 0.46 (0.02; 12.16) | 0.49 (0.02; 12.70) |
| 1.12 (0.83; 1.51) | 1.03 (0.75; 1.41) | 0.86 (0.57; 1.30) | 1.20 (0.33; 4.37) | **PA- SES** | 1.56 (0.34; 7.25) | - | - | - | 1.52 (0.46; 5.05) | 1.02 (0.51; 2.05) | 0.81 (0.27; 2.37) | 1.47 (0.61; 3.56) | 1.55 (0.55; 4.42) |
| 0.99 (0.44; 2.26) | 0.91 (0.39; 2.12) | 0.76 (0.32; 1.85) | 1.06 (0.24; 4.79) | 0.89 (0.38; 2.06) | **PF- AES** | - | - | - | 0.98 (0.27; 3.48) | 0.66 (0.16; 2.64) | 0.52 (0.12; 2.26) | 0.94 (0.23; 3.91) | 1.00 (0.32; 3.07) |
| 2.00 (1.02; 3.93) | 1.84 (0.93; 3.65) | 1.54 (0.74; 3.20) | 2.14 (0.51; 8.95) | 1.79 (0.98; 3.28) | 2.02 (0.72; 5.68) | **PF- BES** | - | - | - | - | - | - | - |
| 1.13 (0.61; 2.07) | 1.04 (0.55; 1.95) | 0.87 (0.43; 1.73) | 1.21 (0.30; 4.89) | 1.01 (0.54; 1.90) | 1.14 (0.43; 3.00) | 0.56 (0.23; 1.35) | **PF- SES + Probucol** | - | - | - | - | - | - |
| 1.68 (0.78; 3.63) | 1.55 (0.71; 3.38) | 1.29 (0.56; 2.96) | 1.80 (0.41; 7.87) | 1.50 (0.69; 3.26) | 1.69 (0.56; 5.08) | 0.84 (0.31; 2.24) | 1.49 (0.58; 3.86) | **PF- TiNO** | - | - | - | - | - |
| 1.38 (0.84; 2.26) | 1.27 (0.75; 2.14) | 1.06 (0.58; 1.92) | 1.47 (0.38; 5.71) | 1.23 (0.73; 2.08) | 1.39 (0.56; 3.42) | 0.69 (0.31; 1.53) | 1.22 (0.60; 2.47) | 0.82 (0.34; 1.98) | **PF- UES** | 0.67 (0.25; 1.83) | 0.53 (0.17; 1.62) | 0.97 (0.34; 2.74) | 1.02 (0.57; 1.83) |
| 1.14 (0.90; 1.46) | 1.05 (0.80; 1.38) | 0.88 (0.59; 1.30) | 1.22 (0.34; 4.42) | 1.02 (0.80; 1.31) | 1.15 (0.51; 2.62) | 0.57 (0.30; 1.10) | 1.01 (0.55; 1.87) | 0.68 (0.33; 1.42) | 0.83 (0.50; 1.37) | **PP- EES** | 0.79 (0.34; 1.82) | 1.44 (0.83; 2.49) | 1.52 (0.67; 3.44) |
| 1.03 (0.76; 1.41) | 0.95 (0.66; 1.36) | 0.79 (0.50; 1.25) | 1.10 (0.30; 4.04) | 0.92 (0.64; 1.32) | 1.04 (0.45; 2.41) | 0.51 (0.25; 1.04) | 0.91 (0.48; 1.75) | 0.61 (0.28; 1.34) | 0.75 (0.43; 1.29) | 0.90 (0.67; 1.22) | **PP- PES** | 1.83 (0.86; 3.86) | 1.93 (0.74; 5.00) |
| 0.90 (0.66; 1.22) | 0.83 (0.61; 1.13) | 0.69 (0.45; 1.07) | 0.96 (0.26; 3.52) | 0.80 (0.58; 1.12) | 0.91 (0.39; 2.11) | 0.45 (0.23; 0.90) | 0.80 (0.42; 1.51) | 0.54 (0.24; 1.17) | 0.65 (0.38; 1.11) | 0.79 (0.59; 1.05) | 0.87 (0.62; 1.23) | **PP- SES** | 1.05 (0.44; 2.50) |
| 1.16 (0.91; 1.48) | 1.07 (0.79; 1.43) | 0.89 (0.59; 1.34) | 1.24 (0.34; 4.47) | 1.03 (0.77; 1.39) | 1.16 (0.53; 2.58) | 0.58 (0.29; 1.14) | 1.03 (0.59; 1.79) | 0.69 (0.32; 1.49) | 0.84 (0.55; 1.29) | 1.01 (0.79; 1.30) | 1.12 (0.81; 1.56) | 1.29 (0.94; 1.76) | **PP- ZES** |

**CVD 1 Year**

**Supplemental Table 13: Net-league estimates of MACE and Mortality over 5-years across different treatment strategies showing risk ratio with its 95% confidence interval**

**Mortality 5 Years**

| **BMS** | 1.13 (0.94; 1.36) | 1.16 (0.78; 1.70) | - | 1.06 (0.86; 1.31) | 1.38 (0.58; 3.31) | 0.78 (0.26; 2.36) | 1.30 (0.83; 2.03) | 1.34 (1.02; 1.75) | 1.14 (0.79; 1.66) | 1.17 (0.99; 1.38) | 1.29 (0.44; 3.80) | 1.11 (0.92; 1.35) | 1.04 (0.88; 1.23) | 1.20 (0.97; 1.48) |
| --- | --- | --- | --- | --- | --- | --- | --- | --- | --- | --- | --- | --- | --- | --- |
| 1.34 (1.21; 1.50) | **PA- BES** | 1.02 (0.70; 1.48) | - | 0.93 (0.78; 1.11) | 1.22 (0.51; 2.91) | 0.69 (0.23; 2.08) | 1.15 (0.74; 1.78) | 1.18 (0.92; 1.51) | 1.01 (0.71; 1.45) | 1.03 (0.91; 1.16) | 1.14 (0.39; 3.34) | 0.98 (0.82; 1.18) | 0.92 (0.81; 1.04) | 1.06 (0.88; 1.27) |
| 1.39 (1.14; 1.69) | 1.03 (0.85; 1.25) | **PA- EES** | - | 0.92 (0.63; 1.34) | 1.19 (0.47; 3.04) | 0.67 (0.21; 2.14) | 1.12 (0.65; 1.96) | 1.16 (0.77; 1.75) | 0.99 (0.61; 1.62) | 1.01 (0.71; 1.43) | 1.12 (0.36; 3.44) | 0.96 (0.66; 1.41) | 0.90 (0.62; 1.30) | 1.03 (0.71; 1.51) |
| 1.51 (0.86; 2.65) | 1.12 (0.63; 1.99) | 1.09 (0.60; 1.97) | **PA- PES** | - | - | - | - | - | - | - | - | - | - | - |
| 1.42 (1.25; 1.61) | 1.05 (0.94; 1.18) | 1.02 (0.84; 1.24) | 0.94 (0.53; 1.67) | **PA- SES** | 1.30 (0.54; 3.13) | 0.73 (0.24; 2.23) | 1.23 (0.78; 1.93) | 1.26 (0.97; 1.64) | 1.08 (0.75; 1.56) | 1.10 (0.95; 1.28) | 1.22 (0.42; 3.58) | 1.05 (0.86; 1.29) | 0.98 (0.85; 1.14) | 1.13 (0.92; 1.38) |
| 1.64 (0.99; 2.73) | 1.22 (0.73; 2.04) | 1.18 (0.69; 2.02) | 1.09 (0.51; 2.32) | 1.16 (0.69; 1.94) | **PF- AES** | 0.56 (0.14; 2.25) | 0.94 (0.37; 2.42) | 0.97 (0.40; 2.35) | 0.83 (0.33; 2.08) | 0.85 (0.36; 2.01) | 0.94 (0.24; 3.69) | 0.81 (0.34; 1.89) | 0.75 (0.32; 1.79) | 0.87 (0.36; 2.07) |
| - | - | - | - | - | - | **PF- BES** | 1.67 (0.52; 5.35) | 1.72 (0.56; 5.27) | 1.47 (0.47; 4.63) | 1.50 (0.50; 4.52) | 1.66 (0.36; 7.68) | 1.43 (0.48; 4.27) | 1.33 (0.44; 4.03) | 1.54 (0.51; 4.65) |
| 1.19 (0.88; 1.60) | 0.88 (0.65; 1.19) | 0.85 (0.61; 1.21) | 0.79 (0.42; 1.49) | 0.84 (0.61; 1.14) | 0.72 (0.41; 1.29) | - | **PF-SES** | 1.03 (0.64; 1.65) | 0.88 (0.52; 1.50) | 0.90 (0.58; 1.38) | 1.00 (0.32; 3.13) | 0.86 (0.57; 1.28) | 0.80 (0.52; 1.24) | 0.92 (0.59; 1.43) |
| 1.45 (1.22; 1.71) | 1.08 (0.92; 1.27) | 1.04 (0.83; 1.31) | 0.96 (0.53; 1.73) | 1.02 (0.86; 1.21) | 0.88 (0.52; 1.49) | - | 1.22 (0.88; 1.69) | **PF-SES**  **+ Probucol** | 0.86 (0.58; 1.27) | 0.87 (0.70; 1.08) | 0.97 (0.33; 2.82) | 0.83 (0.65; 1.07) | 0.78 (0.61; 0.98) | 0.89 (0.76; 1.05) |
| 1.89 (1.47; 2.43) | 1.40 (1.09; 1.81) | 1.36 (1.01; 1.83) | 1.25 (0.68; 2.32) | 1.33 (1.03; 1.72) | 1.15 (0.66; 2.01) | - | 1.59 (1.09; 2.31) | 1.30 (1.00; 1.70) | **PF-TiNO** | 1.02 (0.72; 1.43) | 1.13 (0.37; 3.45) | 0.97 (0.69; 1.38) | 0.91 (0.64; 1.29) | 1.04 (0.73; 1.49) |
| 1.44 (1.31; 1.59) | 1.07 (0.99; 1.17) | 1.04 (0.87; 1.24) | 0.96 (0.54; 1.69) | 1.02 (0.93; 1.12) | 0.88 (0.53; 1.46) | - | 1.22 (0.90; 1.64) | 1.00 (0.86; 1.15) | 0.76 (0.60; 0.97) | **PP- EES** | 1.11 (0.38; 3.22) | 0.96 (0.82; 1.11) | 0.89 (0.80; 0.99) | 1.02 (0.89; 1.18) |
| - | - | - | - | - | - | - | - | - | - | - | **PP- NES** | - | - | - |
| 1.17 (1.06; 1.29) | 0.87 (0.78; 0.97) | 0.84 (0.69; 1.02) | 0.77 (0.44; 1.37) | 0.82 (0.73; 0.93) | 0.71 (0.43; 1.17) | - | 0.98 (0.74; 1.31) | 0.81 (0.69; 0.94) | 0.62 (0.48; 0.79) | 0.81 (0.74; 0.88) | - | **PP- PES** | 0.93 (0.79; 1.10) | 1.07 (0.89; 1.29) |
| 1.28 (1.16; 1.41) | 0.95 (0.88; 1.03) | 0.92 (0.76; 1.12) | 0.85 (0.48; 1.50) | 0.90 (0.81; 1.00) | 0.78 (0.47; 1.30) | - | 1.08 (0.80; 1.46) | 0.88 (0.75; 1.04) | 0.68 (0.53; 0.87) | 0.89 (0.82; 0.96) | - | 1.10 (1.00; 1.21) | **PP- SES** | 1.15 (0.97; 1.37) |
| 1.44 (1.28; 1.64) | 1.07 (0.96; 1.21) | 1.04 (0.86; 1.26) | 0.96 (0.54; 1.70) | 1.02 (0.90; 1.16) | 0.88 (0.53; 1.47) | - | 1.22 (0.90; 1.65) | 1.00 (0.89; 1.12) | 0.76 (0.60; 0.97) | 1.00 (0.92; 1.10) | - | 1.24 (1.11; 1.38) | 1.13 (1.01; 1.26) | **PP- ZES** |

**MACE 5 Years**

**Supplemental Table 14: Net-league estimates of MI and ST over 5-years across different treatment strategies showing risk ratio with its 95% confidence interval**

**ST 5 Years**

| **BMS** | 1.31 (0.95; 1.80) | 1.71 (0.57; 5.09) | 1.77 (1.21; 2.57) | 1.28 (0.28; 5.90) | - | 1.20 (0.56; 2.58) | 1.33 (0.64; 2.78) | 2.87 (0.12; 68.58) | 1.48 (1.08; 2.01) | 1.58 (0.49; 5.14) | 0.94 (0.65; 1.37) | 1.08 (0.80; 1.46) | 1.15 (0.79; 1.69) |
| --- | --- | --- | --- | --- | --- | --- | --- | --- | --- | --- | --- | --- | --- |
| 1.16 (0.94; 1.44) | **PA-BES** | 1.30 (0.44; 3.83) | 1.35 (0.97; 1.88) | 0.98 (0.21; 4.49) | - | 0.91 (0.43; 1.96) | 1.02 (0.49; 2.09) | 2.19 (0.09; 53.14) | 1.13 (0.87; 1.46) | 1.21 (0.38; 3.88) | 0.72 (0.50; 1.04) | 0.83 (0.65; 1.04) | 0.88 (0.62; 1.25) |
| 1.07 (0.74; 1.54) | 0.92 (0.66; 1.29) | **PA-EES** | 1.03 (0.35; 3.04) | 0.75 (0.12; 4.70) | - | 0.70 (0.20; 2.51) | 0.78 (0.22; 2.71) | 1.68 (0.06; 48.15) | 0.86 (0.30; 2.46) | 0.93 (0.20; 4.36) | 0.55 (0.19; 1.63) | 0.63 (0.22; 1.85) | 0.68 (0.23; 1.98) |
| 1.34 (1.04; 1.72) | 1.15 (0.92; 1.43) | 1.25 (0.88; 1.77) | **PA- SES** | 0.72 (0.16; 3.34) | - | 0.68 (0.31; 1.46) | 0.75 (0.36; 1.55) | 1.62 (0.07; 39.65) | 0.84 (0.65; 1.08) | 0.90 (0.28; 2.88) | 0.53 (0.36; 0.78) | 0.61 (0.46; 0.81) | 0.65 (0.46; 0.93) |
| 1.89 (0.72; 4.97) | 1.63 (0.62; 4.28) | 1.77 (0.65; 4.83) | 1.42 (0.54; 3.74) | **PF- AES** | - | 0.94 (0.18; 4.75) | 1.04 (0.20; 5.35) | 2.24 (0.07; 75.82) | 1.15 (0.26; 5.21) | 1.24 (0.19; 8.09) | 0.74 (0.17; 3.23) | 0.84 (0.18; 3.86) | 0.90 (0.20; 4.09) |
| 0.73 (0.14; 3.71) | 0.63 (0.12; 3.19) | 0.68 (0.13; 3.55) | 0.55 (0.11; 2.78) | 0.39 (0.06; 2.49) | **PF- BES** | - | - | - | - | - | - | - | - |
| 0.63 (0.34; 1.15) | 0.54 (0.30; 0.99) | 0.59 (0.30; 1.14) | 0.47 (0.26; 0.87) | 0.33 (0.11; 1.00) | 0.86 (0.16; 4.77) | **PF- SES** | 1.11 (0.42; 2.93) | 2.40 (0.09; 62.74) | 1.23 (0.59; 2.56) | 1.32 (0.35; 5.03) | 0.79 (0.40; 1.54) | 0.90 (0.42; 1.92) | 0.96 (0.46; 2.02) |
| 1.43 (0.92; 2.23) | 1.23 (0.80; 1.89) | 1.34 (0.81; 2.20) | 1.07 (0.69; 1.66) | 0.76 (0.27; 2.12) | 1.96 (0.37; 10.37) | 2.27 (1.12; 4.60) | **PF-SES + Probucol** | 2.16 (0.08; 56.08) | 1.11 (0.56; 2.19) | 1.19 (0.33; 4.28) | 0.71 (0.35; 1.43) | 0.81 (0.40; 1.66) | 0.87 (0.46; 1.63) |
| 1.94 (1.32; 2.85) | 1.67 (1.14; 2.43) | 1.81 (1.15; 2.87) | 1.45 (0.98; 2.14) | 1.02 (0.37; 2.80) | 2.66 (0.51; 13.84) | 3.08 (1.58; 5.99) | 1.36 (0.81; 2.27) | **PF-TiNO** | 0.51 (0.02; 12.49) | 0.55 (0.02; 16.30) | 0.33 (0.01; 8.02) | 0.38 (0.02; 9.14) | 0.40 (0.02; 9.84) |
| 1.25 (1.01; 1.53) | 1.07 (0.91; 1.26) | 1.17 (0.87; 1.57) | 0.93 (0.78; 1.12) | 0.66 (0.25; 1.72) | 1.71 (0.34; 8.65) | 1.98 (1.09; 3.58) | 0.87 (0.58; 1.30) | 0.64 (0.45; 0.91) | **PP- EES** | 1.07 (0.34; 3.36) | 0.64 (0.48; 0.85) | 0.73 (0.58; 0.92) | 0.78 (0.61; 1.00) |
| 3.39 (1.08; 10.65) | 2.91 (0.93; 9.12) | 3.17 (0.98; 10.19) | 2.53 (0.81; 7.95) | 1.79 (0.41; 7.84) | 4.64 (0.64; 33.36) | 5.37 (1.51; 19.14) | 2.37 (0.74; 7.61) | 1.75 (0.54; 5.66) | 2.71 (0.88; 8.40) | **PP- NES** | 0.59 (0.19; 1.89) | 0.68 (0.21; 2.18) | 0.73 (0.24; 2.22) |
| 0.89 (0.72; 1.10) | 0.77 (0.62; 0.95) | 0.83 (0.59; 1.18) | 0.67 (0.52; 0.85) | 0.47 (0.18; 1.21) | 1.22 (0.24; 6.11) | 1.41 (0.80; 2.49) | 0.62 (0.41; 0.95) | 0.46 (0.32; 0.65) | 0.71 (0.60; 0.85) | 0.26 (0.08; 0.82) | **PP- PES** | 1.15 (0.81; 1.63) | 1.23 (0.90; 1.67) |
| 1.09 (0.89; 1.34) | 0.94 (0.80; 1.11) | 1.02 (0.73; 1.43) | 0.82 (0.68; 0.98) | 0.58 (0.22; 1.51) | 1.50 (0.30; 7.61) | 1.74 (0.95; 3.16) | 0.77 (0.50; 1.17) | 0.56 (0.39; 0.82) | 0.88 (0.75; 1.02) | 0.32 (0.10; 1.01) | 1.23 (1.00; 1.50) | **PP- SES** | 1.07 (0.77; 1.49) |
| 1.31 (1.00; 1.72) | 1.13 (0.88; 1.44) | 1.22 (0.86; 1.75) | 0.98 (0.76; 1.27) | 0.69 (0.26; 1.83) | 1.79 (0.35; 9.15) | 2.08 (1.13; 3.83) | 0.92 (0.64; 1.30) | 0.68 (0.46; 0.98) | 1.05 (0.87; 1.27) | 0.39 (0.13; 1.18) | 1.47 (1.16; 1.86) | 1.20 (0.94; 1.52) | **PP- ZES** |

**MI 5 Years**

**Supplemental Table 15: Net-league estimates of TLR and TVR over 5-years across different treatment strategies showing risk ratio with its 95% confidence interval**

**TVR 5 years**

| **BMS** | - | 1.41 (0.88; 2.26) | 1.69 (1.44; 1.98) | 1.79 (1.32; 2.43) | 1.91 (1.04; 3.51) | 1.67 (1.37; 2.03) | 1.76 (0.84; 3.68) | 1.11 (0.54; 2.28) | 1.70 (1.00; 2.89) | 1.71 (1.35; 2.16) | 1.66 (1.03; 2.68) | 1.82 (1.58; 2.10) | 3.64 (1.84; 7.20) | 1.40 (1.23; 1.59) | 1.59 (1.38; 1.83) | 1.73 (1.44; 2.07) |
| --- | --- | --- | --- | --- | --- | --- | --- | --- | --- | --- | --- | --- | --- | --- | --- | --- |
| 1.34 (0.79; 2.27) | **NA- PES** | - | - | - | - | - | - | - | - | - | - | - | - | - | - | - |
| 1.82 (1.03; 3.22) | 1.36 (0.62; 2.97) | **NA- SES** | 1.20 (0.73; 1.97) | 1.27 (0.73; 2.22) | 1.35 (0.62; 2.93) | 1.19 (0.71; 1.97) | 1.25 (0.52; 2.98) | 0.79 (0.33; 1.86) | 1.21 (0.60; 2.44) | 1.21 (0.72; 2.05) | 1.18 (0.60; 2.30) | 1.29 (0.79; 2.11) | 2.59 (1.13; 5.90) | 0.99 (0.61; 1.60) | 1.13 (0.69; 1.84) | 1.23 (0.74; 2.02) |
| 2.35 (1.96; 2.82) | 1.76 (1.01; 3.08) | 1.29 (0.72; 2.34) | **PA-BES** | 1.06 (0.79; 1.42) | 1.13 (0.60; 2.12) | 0.99 (0.82; 1.19) | 1.04 (0.49; 2.19) | 0.66 (0.32; 1.36) | 1.01 (0.59; 1.72) | 1.01 (0.80; 1.27) | 0.98 (0.61; 1.58) | 1.08 (0.95; 1.22) | 2.15 (1.09; 4.25) | 0.83 (0.71; 0.97) | 0.94 (0.83; 1.06) | 1.02 (0.86; 1.21) |
| 2.02 (1.33; 3.09) | 1.52 (0.77; 2.98) | 1.11 (0.55; 2.25) | 0.86 (0.57; 1.30) | **PA-EES** | 1.06 (0.54; 2.11) | 0.93 (0.69; 1.27) | 0.98 (0.45; 2.16) | 0.62 (0.29; 1.34) | 0.95 (0.53; 1.72) | 0.96 (0.68; 1.34) | 0.93 (0.54; 1.58) | 1.02 (0.78; 1.33) | 2.04 (0.99; 4.19) | 0.78 (0.58; 1.05) | 0.89 (0.66; 1.19) | 0.96 (0.72; 1.30) |
| 2.19 (1.13; 4.25) | 1.64 (0.70; 3.83) | 1.20 (0.50; 2.89) | 0.93 (0.47; 1.85) | 1.08 (0.49; 2.37) | **PA- PES** | 0.88 (0.46; 1.66) | 0.92 (0.35; 2.41) | 0.58 (0.23; 1.50) | 0.89 (0.40; 2.00) | 0.90 (0.47; 1.73) | 0.87 (0.40; 1.89) | 0.96 (0.51; 1.79) | 1.91 (0.77; 4.77) | 0.73 (0.39; 1.37) | 0.83 (0.45; 1.56) | 0.91 (0.48; 1.71) |
| 2.27 (1.87; 2.76) | 1.70 (0.97; 2.99) | 1.25 (0.69; 2.27) | 0.96 (0.80; 1.16) | 1.12 (0.74; 1.70) | 1.04 (0.52; 2.07) | **PA- SES** | 1.05 (0.50; 2.23) | 0.66 (0.32; 1.38) | 1.02 (0.59; 1.76) | 1.02 (0.80; 1.31) | 0.99 (0.61; 1.62) | 1.09 (0.93; 1.27) | 2.18 (1.10; 4.33) | 0.84 (0.69; 1.01) | 0.95 (0.80; 1.13) | 1.03 (0.85; 1.26) |
| 2.60 (1.30; 5.17) | 1.94 (0.82; 4.64) | 1.43 (0.59; 3.46) | 1.10 (0.55; 2.21) | 1.28 (0.58; 2.82) | 1.18 (0.46; 3.08) | 1.14 (0.57; 2.30) | **PF- AES** | 0.63 (0.23; 1.74) | 0.97 (0.40; 2.36) | 0.97 (0.46; 2.08) | 0.94 (0.40; 2.24) | 1.04 (0.50; 2.17) | 2.07 (0.77; 5.59) | 0.80 (0.38; 1.64) | 0.90 (0.43; 1.89) | 0.98 (0.47; 2.06) |
| 1.59 (0.63; 4.00) | 1.19 (0.41; 3.45) | 0.87 (0.30; 2.57) | 0.67 (0.27; 1.71) | 0.78 (0.29; 2.13) | 0.72 (0.23; 2.26) | 0.70 (0.28; 1.77) | 0.61 (0.20; 1.90) | **PF- BES** | 1.54 (0.64; 3.69) | 1.54 (0.73; 3.24) | 1.49 (0.64; 3.51) | 1.64 (0.80; 3.38) | 3.29 (1.23; 8.76) | 1.26 (0.62; 2.56) | 1.43 (0.69; 2.95) | 1.56 (0.75; 3.22) |
| 1.79 (1.21; 2.65) | 1.34 (0.69; 2.59) | 0.98 (0.50; 1.94) | 0.76 (0.51; 1.14) | 0.88 (0.51; 1.53) | 0.82 (0.38; 1.76) | 0.79 (0.52; 1.18) | 0.69 (0.32; 1.47) | 1.13 (0.42; 2.99) | **PF- SES** | 1.00 (0.58; 1.75) | 0.97 (0.48; 1.95) | 1.07 (0.63; 1.81) | 2.14 (0.92; 4.99) | 0.82 (0.49; 1.37) | 0.93 (0.55; 1.58) | 1.01 (0.59; 1.73) |
| 2.05 (1.52; 2.75) | 1.53 (0.84; 2.81) | 1.12 (0.60; 2.12) | 0.87 (0.65; 1.16) | 1.01 (0.63; 1.61) | 0.93 (0.45; 1.93) | 0.90 (0.67; 1.21) | 0.79 (0.38; 1.63) | 1.29 (0.50; 3.33) | 1.14 (0.73; 1.80) | **PF-SES + Probucol** | 0.97 (0.61; 1.55) | 1.06 (0.87; 1.30) | 2.13 (1.09; 4.18) | 0.82 (0.66; 1.02) | 0.93 (0.74; 1.16) | 1.01 (0.87; 1.18) |
| 1.71 (0.93; 3.14) | 1.28 (0.57; 2.87) | 0.94 (0.41; 2.15) | 0.73 (0.40; 1.33) | 0.85 (0.42; 1.72) | 0.78 (0.32; 1.92) | 0.75 (0.41; 1.39) | 0.66 (0.27; 1.62) | 1.08 (0.36; 3.20) | 0.96 (0.48; 1.92) | 0.84 (0.46; 1.52) | **PF-TiNO** | 1.10 (0.69; 1.75) | 2.20 (0.99; 4.86) | 0.84 (0.53; 1.35) | 0.96 (0.60; 1.54) | 1.04 (0.67; 1.63) |
| 2.44 (2.07; 2.87) | 1.82 (1.05; 3.17) | 1.34 (0.75; 2.41) | 1.04 (0.90; 1.19) | 1.20 (0.82; 1.78) | 1.11 (0.56; 2.20) | 1.07 (0.92; 1.25) | 0.94 (0.47; 1.87) | 1.53 (0.61; 3.86) | 1.36 (0.92; 2.01) | 1.19 (0.92; 1.55) | 1.42 (0.79; 2.57) | **PP- EES** | 2.00 (1.02; 3.91) | 0.77 (0.67; 0.87) | 0.87 (0.77; 0.98) | 0.95 (0.83; 1.08) |
| 5.08 (1.78; 14.47) | 3.80 (1.18; 12.29) | 2.79 (0.85; 9.16) | 2.16 (0.76; 6.14) | 2.51 (0.83; 7.60) | 2.32 (0.67; 8.00) | 2.24 (0.79; 6.38) | 1.96 (0.57; 6.76) | 3.20 (0.80; 12.76) | 2.84 (0.94; 8.56) | 2.48 (0.88; 7.03) | 2.97 (0.92; 9.54) | 2.09 (0.74; 5.89) | **PP- NES** | 0.38 (0.20; 0.75) | 0.44 (0.22; 0.86) | 0.47 (0.25; 0.91) |
| 1.80 (1.53; 2.11) | 1.35 (0.78; 2.34) | 0.99 (0.55; 1.76) | 0.76 (0.64; 0.91) | 0.89 (0.59; 1.35) | 0.82 (0.42; 1.62) | 0.79 (0.65; 0.96) | 0.69 (0.35; 1.36) | 1.13 (0.46; 2.81) | 1.01 (0.70; 1.44) | 0.88 (0.67; 1.16) | 1.05 (0.58; 1.91) | 0.74 (0.64; 0.86) | 0.35 (0.12; 1.00) | **PP- PES** | 1.14 (0.99; 1.30) | 1.23 (1.05; 1.45) |
| 2.08 (1.78; 2.43) | 1.56 (0.90; 2.71) | 1.15 (0.64; 2.06) | 0.89 (0.77; 1.02) | 1.03 (0.68; 1.55) | 0.95 (0.48; 1.88) | 0.92 (0.79; 1.06) | 0.80 (0.40; 1.60) | 1.31 (0.52; 3.30) | 1.17 (0.79; 1.72) | 1.02 (0.77; 1.35) | 1.22 (0.67; 2.22) | 0.85 (0.76; 0.97) | 0.41 (0.14; 1.16) | 1.16 (0.99; 1.35) | **PP- SES** | 1.09 (0.92; 1.28) |
| 2.08 (1.66; 2.60) | 1.55 (0.88; 2.76) | 1.14 (0.62; 2.09) | 0.88 (0.71; 1.10) | 1.03 (0.67; 1.57) | 0.95 (0.47; 1.91) | 0.92 (0.73; 1.15) | 0.80 (0.40; 1.61) | 1.31 (0.52; 3.32) | 1.16 (0.77; 1.75) | 1.02 (0.84; 1.23) | 1.21 (0.69; 2.13) | 0.85 (0.71; 1.02) | 0.41 (0.15; 1.14) | 1.15 (0.95; 1.41) | 1.00 (0.81; 1.22) | **PP- ZES** |

**TLR 5 years**

**Supplemental Table 16: Net-league estimates of CVD over 5-years across different treatment strategies showing risk ratio with its 95% confidence interval**

| **BMS** |  |  |  |  |  |  |  |  |  |  |  |  |  |  |
| --- | --- | --- | --- | --- | --- | --- | --- | --- | --- | --- | --- | --- | --- | --- |
| 1.06 (0.82; 1.37) | **PA-BES** |  |  |  |  |  |  |  |  |  |  |  |  |  |
| 1.31 (0.76; 2.25) | 1.24 (0.73; 2.09) | **PA-EES** |  |  |  |  |  |  |  |  |  |  |  |  |
| 0.18 (0.01; 3.26) | 0.17 (0.01; 3.12) | 0.14 (0.01; 2.62) | **PA- PES** |  |  |  |  |  |  |  |  |  |  |  |
| 1.09 (0.81; 1.46) | 1.03 (0.79; 1.34) | 0.83 (0.49; 1.42) | 5.94 (0.33; 107.16) | **PA- SES** |  |  |  |  |  |  |  |  |  |  |
| 1.05 (0.26; 4.24) | 1.00 (0.25; 4.01) | 0.80 (0.19; 3.50) | 5.74 (0.23; 140.56) | 0.97 (0.24; 3.92) | **PF- AES** |  |  |  |  |  |  |  |  |  |
| 0.20 (0.01; 3.53) | 0.18 (0.01; 3.33) | 0.15 (0.01; 2.79) | 1.07 (0.02; 63.07) | 0.18 (0.01; 3.25) | 0.19 (0.01; 4.50) | **PF- BES** |  |  |  |  |  |  |  |  |
| 1.19 (0.67; 2.13) | 1.12 (0.63; 2.01) | 0.91 (0.43; 1.91) | 6.49 (0.34; 122.27) | 1.09 (0.60; 1.98) | 1.13 (0.26; 4.87) | 6.08 (0.33; 113.32) | **PF- SES** |  |  |  |  |  |  |  |
| 1.14 (0.77; 1.68) | 1.07 (0.74; 1.56) | 0.87 (0.48; 1.56) | 6.20 (0.34; 113.20) | 1.04 (0.71; 1.54) | 1.08 (0.26; 4.45) | 5.81 (0.32; 105.95) | 0.96 (0.51; 1.80) | **PF-SES + Probucol** |  |  |  |  |  |  |
| 1.56 (0.84; 2.90) | 1.48 (0.80; 2.72) | 1.19 (0.56; 2.56) | 8.53 (0.45; 161.95) | 1.44 (0.77; 2.67) | 1.49 (0.33; 6.61) | 8.00 (0.42; 151.37) | 1.31 (0.60; 2.90) | 1.38 (0.72; 2.64) | **PF- TiNO** |  |  |  |  |  |
| 1.14 (0.91; 1.43) | 1.08 (0.89; 1.31) | 0.87 (0.53; 1.42) | 6.23 (0.35; 111.73) | 1.05 (0.85; 1.30) | 1.08 (0.27; 4.33) | 5.84 (0.33; 104.81) | 0.96 (0.55; 1.68) | 1.00 (0.73; 1.39) | 0.73 (0.41; 1.31) | **PP- EES** |  |  |  |  |
| 1.66 (0.37; 7.43) | 1.56 (0.35; 6.99) | 1.26 (0.26; 6.04) | 9.03 (0.35; 231.87) | 1.52 (0.34; 6.82) | 1.57 (0.21; 11.93) | 8.46 (0.33; 217.06) | 1.39 (0.29; 6.77) | 1.46 (0.33; 6.47) | 1.06 (0.22; 5.18) | 1.45 (0.33; 6.40) | **PP- NES** |  |  |  |
| 1.03 (0.78; 1.34) | 0.97 (0.74; 1.27) | 0.78 (0.46; 1.34) | 5.59 (0.31; 100.67) | 0.94 (0.70; 1.27) | 0.97 (0.25; 3.82) | 5.24 (0.29; 93.29) | 0.86 (0.51; 1.44) | 0.90 (0.62; 1.31) | 0.66 (0.36; 1.19) | 0.90 (0.72; 1.12) | 0.62 (0.14; 2.76) | **PP- PES** |  |  |
| 1.00 (0.79; 1.26) | 0.94 (0.78; 1.14) | 0.76 (0.45; 1.28) | 5.43 (0.30; 97.43) | 0.91 (0.73; 1.14) | 0.94 (0.24; 3.79) | 5.09 (0.28; 91.51) | 0.84 (0.47; 1.48) | 0.87 (0.61; 1.26) | 0.64 (0.35; 1.16) | 0.87 (0.73; 1.03) | 0.60 (0.13; 2.68) | 0.97 (0.76; 1.25) | **PP- SES** |  |
| 1.14 (0.85; 1.54) | 1.08 (0.82; 1.43) | 0.87 (0.51; 1.48) | 6.23 (0.35; 112.49) | 1.05 (0.78; 1.41) | 1.08 (0.27; 4.37) | 5.84 (0.32; 105.29) | 0.96 (0.54; 1.72) | 1.00 (0.78; 1.29) | 0.73 (0.40; 1.33) | 1.00 (0.81; 1.23) | 0.69 (0.16; 3.00) | 1.11 (0.85; 1.46) | 1.15 (0.88; 1.49) | **PP- ZES** |

**CVD 5 years**

**Supplemental Table 17: Proportion and comparison of contribution of direct and indirect evidence for the mixed treatment loop for MACE at 1 and 5 years.**

| Comparison | k | prop | nma | direct | indirect | RoR | z | p-value | Comparison | k | prop | nma | direct | indirect | RoR | z | p-value |
| --- | --- | --- | --- | --- | --- | --- | --- | --- | --- | --- | --- | --- | --- | --- | --- | --- | --- |
| 1-year | | | | | | | | | 5 year | | | | | | | | |
| BMS vs. PA-Biolimus | 1 | 0.11 | 1.6004 | 1.9065 | 1.5654 | 1.2179 | 0.97 | 0.3306 | BMS vs. PA-Biolimus | 1 | 0.23 | 1.3441 | 1.369 | 1.3369 | 1.024 | 0.18 | 0.8557 |
| BMS vs. PA-Everolimus | 1 | 0.22 | 1.7721 | 1.302 | 1.9321 | 0.6739 | -1.79 | 0.073 | BMS vs. PA-Everolimus | 0 | 0 | 1.3876 | . | 1.3876 | . | . | . |
| BMS vs. PA-Paclitaxel | 2 | 1 | 1.4566 | 1.4566 | . | . | . | . | BMS vs. PA-Paclitaxel | 1 | 1 | 1.5086 | 1.5086 | . | . | . | . |
| BMS vs. PA-Sirolimus | 2 | 0.08 | 1.8375 | 1.928 | 1.8295 | 1.0538 | 0.23 | 0.818 | BMS vs. PA-Sirolimus | 1 | 0.05 | 1.416 | 1.5789 | 1.4083 | 1.1212 | 0.38 | 0.7049 |
| BMS vs. PF-Amphilimus | 0 | 0 | 1.4821 | . | 1.4821 | . | . | . | BMS vs. PF-Amphilimus | 0 | 0 | 1.6403 | . | 1.6403 | . | . | . |
| BMS vs. PF-Biolimus | 0 | 0 | 1.7774 | . | 1.7774 | . | . | . | BMS vs. PF-Sirolimus | 0 | 0 | 1.1862 | . | 1.1862 | . | . | . |
| BMS vs. PF-Sirolimus+Probucol | 0 | 0 | 1.6778 | . | 1.6778 | . | . | . | BMS vs. PF-Sirolimus+Probucol | 0 | 0 | 1.4485 | . | 1.4485 | . | . | . |
| BMS vs. PF-TiNO | 0 | 0 | 1.902 | . | 1.902 | . | . | . | BMS vs. PF-TiNO | 1 | 0.01 | 1.8882 | 2.8085 | 1.8784 | 1.4952 | 0.35 | 0.7249 |
| BMS vs. PF-Umirolimus | 0 | 0 | 1.5305 | . | 1.5305 | . | . | . | BMS vs. PP-Everolimus | 2 | 0.22 | 1.4421 | 1.1727 | 1.5301 | 0.7664 | -2.22 | 0.0265 |
| BMS vs. PP-Everolimus | 3 | 0.2 | 1.7786 | 1.582 | 1.8315 | 0.8637 | -1.09 | 0.2754 | BMS vs. PP-Paclitaxel | 3 | 0.56 | 1.1675 | 1.2276 | 1.0945 | 1.1216 | 1.16 | 0.2465 |
| BMS vs. PP-Paclitaxel | 4 | 0.38 | 1.2624 | 1.3719 | 1.2006 | 1.1427 | 1.12 | 0.2614 | BMS vs. PP-Sirolimus | 6 | 0.4 | 1.2801 | 1.3102 | 1.2601 | 1.0397 | 0.39 | 0.6992 |
| BMS vs. PP-Sirolimus | 9 | 0.4 | 1.6997 | 1.8486 | 1.6073 | 1.1501 | 1.24 | 0.2144 | BMS vs. PP-Zotarolimus | 0 | 0 | 1.4442 | . | 1.4442 | . | . | . |
| BMS vs. PP-Zotarolimus | 2 | 0.31 | 1.656 | 1.495 | 1.7341 | 0.8621 | -1.22 | 0.2226 | PA-Biolimus vs. PA-Everolimus | 0 | 0 | 1.0324 | . | 1.0324 | . | . | . |
| PA-Biolimus vs. PA-Everolimus | 1 | 0.42 | 1.1073 | 1.2021 | 1.0445 | 1.151 | 0.81 | 0.4192 | PA-Biolimus vs. PA-Paclitaxel | 0 | 0 | 1.1223 | . | 1.1223 | . | . | . |
| PA-Biolimus vs. PA-Paclitaxel | 0 | 0 | 0.9101 | . | 0.9101 | . | . | . | PA-Biolimus vs. PA-Sirolimus | 0 | 0 | 1.0535 | . | 1.0535 | . | . | . |
| PA-Biolimus vs. PA-Sirolimus | 1 | 0.19 | 1.1481 | 1.2682 | 1.1223 | 1.13 | 0.8 | 0.4251 | PA-Biolimus vs. PF-Amphilimus | 0 | 0 | 1.2204 | . | 1.2204 | . | . | . |
| PA-Biolimus vs. PF-Amphilimus | 0 | 0 | 0.9261 | . | 0.9261 | . | . | . | PA-Biolimus vs. PF-Sirolimus | 0 | 0 | 0.8825 | . | 0.8825 | . | . | . |
| PA-Biolimus vs. PF-Biolimus | 0 | 0 | 1.1106 | . | 1.1106 | . | . | . | PA-Biolimus vs. PF-Sirolimus+Probucol | 0 | 0 | 1.0777 | . | 1.0777 | . | . | . |
| PA-Biolimus vs. PF-Sirolimus+Probucol | 0 | 0 | 1.0484 | . | 1.0484 | . | . | . | PA-Biolimus vs. PF-TiNO | 0 | 0 | 1.4048 | . | 1.4048 | . | . | . |
| PA-Biolimus vs. PF-TiNO | 0 | 0 | 1.1884 | . | 1.1884 | . | . | . | PA-Biolimus vs. PP-Everolimus | 2 | 0.57 | 1.0729 | 1.1079 | 1.0291 | 1.0766 | 0.87 | 0.3851 |
| PA-Biolimus vs. PF-Umirolimus | 0 | 0 | 0.9563 | . | 0.9563 | . | . | . | PA-Biolimus vs. PP-Paclitaxel | 1 | 0.05 | 0.8686 | 0.9123 | 0.8662 | 1.0532 | 0.21 | 0.8308 |
| PA-Biolimus vs. PP-Everolimus | 4 | 0.42 | 1.1113 | 1.0904 | 1.1269 | 0.9676 | -0.32 | 0.7499 | PA-Biolimus vs. PP-Sirolimus | 2 | 0.59 | 0.9524 | 0.9254 | 0.9921 | 0.9327 | -0.82 | 0.411 |
| PA-Biolimus vs. PP-Paclitaxel | 0 | 0 | 0.7888 | . | 0.7888 | . | . | . | PA-Biolimus vs. PP-Zotarolimus | 0 | 0 | 1.0745 | . | 1.0745 | . | . | . |
| PA-Biolimus vs. PP-Sirolimus | 2 | 0.38 | 1.062 | 1.0319 | 1.0808 | 0.9548 | -0.39 | 0.6941 | PA-Everolimus vs. PA-Paclitaxel | 0 | 0 | 1.0872 | . | 1.0872 | . | . | . |
| PA-Biolimus vs. PP-Zotarolimus | 1 | 0.22 | 1.0347 | 1.057 | 1.0284 | 1.0278 | 0.19 | 0.8455 | PA-Everolimus vs. PA-Sirolimus | 0 | 0 | 1.0205 | . | 1.0205 | . | . | . |
| PA-Everolimus vs. PA-Paclitaxel | 0 | 0 | 0.822 | . | 0.822 | . | . | . | PA-Everolimus vs. PF-Amphilimus | 0 | 0 | 1.1821 | . | 1.1821 | . | . | . |
| PA-Everolimus vs. PA-Sirolimus | 1 | 0.25 | 1.0369 | 0.8952 | 1.0876 | 0.8231 | -0.95 | 0.3402 | PA-Everolimus vs. PF-Sirolimus | 0 | 0 | 0.8549 | . | 0.8549 | . | . | . |
| PA-Everolimus vs. PF-Amphilimus | 0 | 0 | 0.8364 | . | 0.8364 | . | . | . | PA-Everolimus vs. PF-Sirolimus+Probucol | 0 | 0 | 1.0439 | . | 1.0439 | . | . | . |
| PA-Everolimus vs. PF-Biolimus | 0 | 0 | 1.003 | . | 1.003 | . | . | . | PA-Everolimus vs. PF-TiNO | 0 | 0 | 1.3608 | . | 1.3608 | . | . | . |
| PA-Everolimus vs. PF-Sirolimus+Probucol | 0 | 0 | 0.9468 | . | 0.9468 | . | . | . | PA-Everolimus vs. PP-Everolimus | 2 | 1 | 1.0393 | 1.0393 | . | . | . | . |
| PA-Everolimus vs. PF-TiNO | 0 | 0 | 1.0733 | . | 1.0733 | . | . | . | PA-Everolimus vs. PP-Paclitaxel | 0 | 0 | 0.8414 | . | 0.8414 | . | . | . |
| PA-Everolimus vs. PF-Umirolimus | 0 | 0 | 0.8637 | . | 0.8637 | . | . | . | PA-Everolimus vs. PP-Sirolimus | 0 | 0 | 0.9225 | . | 0.9225 | . | . | . |
| PA-Everolimus vs. PP-Everolimus | 1 | 0.3 | 1.0036 | 1.0319 | 0.9919 | 1.0404 | 0.22 | 0.8294 | PA-Everolimus vs. PP-Zotarolimus | 0 | 0 | 1.0408 | . | 1.0408 | . | . | . |
| PA-Everolimus vs. PP-Paclitaxel | 0 | 0 | 0.7124 | . | 0.7124 | . | . | . | PA-Paclitaxel vs. PA-Sirolimus | 0 | 0 | 0.9387 | . | 0.9387 | . | . | . |
| PA-Everolimus vs. PP-Sirolimus | 0 | 0 | 0.9591 | . | 0.9591 | . | . | . | PA-Paclitaxel vs. PF-Amphilimus | 0 | 0 | 1.0873 | . | 1.0873 | . | . | . |
| PA-Everolimus vs. PP-Zotarolimus | 0 | 0 | 0.9345 | . | 0.9345 | . | . | . | PA-Paclitaxel vs. PF-Sirolimus | 0 | 0 | 0.7863 | . | 0.7863 | . | . | . |
| PA-Paclitaxel vs. PA-Sirolimus | 0 | 0 | 1.2615 | . | 1.2615 | . | . | . | PA-Paclitaxel vs. PF-Sirolimus+Probucol | 0 | 0 | 0.9602 | . | 0.9602 | . | . | . |
| PA-Paclitaxel vs. PF-Amphilimus | 0 | 0 | 1.0175 | . | 1.0175 | . | . | . | PA-Paclitaxel vs. PF-TiNO | 0 | 0 | 1.2517 | . | 1.2517 | . | . | . |
| PA-Paclitaxel vs. PF-Biolimus | 0 | 0 | 1.2202 | . | 1.2202 | . | . | . | PA-Paclitaxel vs. PP-Everolimus | 0 | 0 | 0.956 | . | 0.956 | . | . | . |
| PA-Paclitaxel vs. PF-Sirolimus+Probucol | 0 | 0 | 1.1518 | . | 1.1518 | . | . | . | PA-Paclitaxel vs. PP-Paclitaxel | 0 | 0 | 0.7739 | . | 0.7739 | . | . | . |
| PA-Paclitaxel vs. PF-TiNO | 0 | 0 | 1.3057 | . | 1.3057 | . | . | . | PA-Paclitaxel vs. PP-Sirolimus | 0 | 0 | 0.8486 | . | 0.8486 | . | . | . |
| PA-Paclitaxel vs. PF-Umirolimus | 0 | 0 | 1.0507 | . | 1.0507 | . | . | . | PA-Paclitaxel vs. PP-Zotarolimus | 0 | 0 | 0.9573 | . | 0.9573 | . | . | . |
| PA-Paclitaxel vs. PP-Everolimus | 0 | 0 | 1.221 | . | 1.221 | . | . | . | PA-Sirolimus vs. PF-Amphilimus | 0 | 0 | 1.1584 | . | 1.1584 | . | . | . |
| PA-Paclitaxel vs. PP-Paclitaxel | 0 | 0 | 0.8666 | . | 0.8666 | . | . | . | PA-Sirolimus vs. PF-Sirolimus | 0 | 0 | 0.8377 | . | 0.8377 | . | . | . |
| PA-Paclitaxel vs. PP-Sirolimus | 0 | 0 | 1.1669 | . | 1.1669 | . | . | . | PA-Sirolimus vs. PF-Sirolimus+Probucol | 0 | 0 | 1.0229 | . | 1.0229 | . | . | . |
| PA-Paclitaxel vs. PP-Zotarolimus | 0 | 0 | 1.1369 | . | 1.1369 | . | . | . | PA-Sirolimus vs. PF-TiNO | 0 | 0 | 1.3334 | . | 1.3334 | . | . | . |
| PA-Sirolimus vs. PF-Amphilimus | 0 | 0 | 0.8066 | . | 0.8066 | . | . | . | PA-Sirolimus vs. PP-Everolimus | 3 | 0.68 | 1.0184 | 0.9768 | 1.1138 | 0.877 | -1.25 | 0.2115 |
| PA-Sirolimus vs. PF-Biolimus | 1 | 1 | 0.9673 | 0.9673 | . | . | . | . | PA-Sirolimus vs. PP-Paclitaxel | 0 | 0 | 0.8245 | . | 0.8245 | . | . | . |
| PA-Sirolimus vs. PF-Sirolimus+Probucol | 0 | 0 | 0.9131 | . | 0.9131 | . | . | . | PA-Sirolimus vs. PP-Sirolimus | 2 | 0.46 | 0.904 | 0.98 | 0.8443 | 1.1607 | 1.4 | 0.1615 |
| PA-Sirolimus vs. PF-TiNO | 0 | 0 | 1.0351 | . | 1.0351 | . | . | . | PA-Sirolimus vs. PP-Zotarolimus | 0 | 0 | 1.0199 | . | 1.0199 | . | . | . |
| PA-Sirolimus vs. PF-Umirolimus | 0 | 0 | 0.8329 | . | 0.8329 | . | . | . | PF-Amphilimus vs. PF-Sirolimus | 0 | 0 | 0.7232 | . | 0.7232 | . | . | . |
| PA-Sirolimus vs. PP-Everolimus | 14 | 0.64 | 0.9679 | 0.9479 | 1.0052 | 0.943 | -0.63 | 0.5258 | PF-Amphilimus vs. PF-Sirolimus+Probucol | 0 | 0 | 0.8831 | . | 0.8831 | . | . | . |
| PA-Sirolimus vs. PP-Paclitaxel | 0 | 0 | 0.687 | . | 0.687 | . | . | . | PF-Amphilimus vs. PF-TiNO | 0 | 0 | 1.1511 | . | 1.1511 | . | . | . |
| PA-Sirolimus vs. PP-Sirolimus | 2 | 0.19 | 0.925 | 0.996 | 0.9086 | 1.0961 | 0.63 | 0.5292 | PF-Amphilimus vs. PP-Everolimus | 0 | 0 | 0.8792 | . | 0.8792 | . | . | . |
| PA-Sirolimus vs. PP-Zotarolimus | 5 | 0.25 | 0.9012 | 0.9469 | 0.8863 | 1.0683 | 0.52 | 0.6032 | PF-Amphilimus vs. PP-Paclitaxel | 1 | 1 | 0.7118 | 0.7118 | . | . | . | . |
| PF-Amphilimus vs. PF-Biolimus | 0 | 0 | 1.1992 | . | 1.1992 | . | . | . | PF-Amphilimus vs. PP-Sirolimus | 0 | 0 | 0.7804 | . | 0.7804 | . | . | . |
| PF-Amphilimus vs. PF-Sirolimus+Probucol | 0 | 0 | 1.132 | . | 1.132 | . | . | . | PF-Amphilimus vs. PP-Zotarolimus | 0 | 0 | 0.8804 | . | 0.8804 | . | . | . |
| PF-Amphilimus vs. PF-TiNO | 0 | 0 | 1.2832 | . | 1.2832 | . | . | . | PF-Sirolimus vs. PF-Sirolimus+Probucol | 0 | 0 | 1.2211 | . | 1.2211 | . | . | . |
| PF-Amphilimus vs. PF-Umirolimus | 0 | 0 | 1.0326 | . | 1.0326 | . | . | . | PF-Sirolimus vs. PF-TiNO | 0 | 0 | 1.5918 | . | 1.5918 | . | . | . |
| PF-Amphilimus vs. PP-Everolimus | 1 | 0.12 | 1.2 | 0.5333 | 1.3374 | 0.3988 | -1.47 | 0.1405 | PF-Sirolimus vs. PP-Everolimus | 0 | 0 | 1.2158 | . | 1.2158 | . | . | . |
| PF-Amphilimus vs. PP-Paclitaxel | 1 | 0.15 | 0.8517 | 1.4713 | 0.7706 | 1.9093 | 1.15 | 0.2484 | PF-Sirolimus vs. PP-Paclitaxel | 2 | 1 | 0.9843 | 0.9843 | . | . | . | . |
| PF-Amphilimus vs. PP-Sirolimus | 0 | 0 | 1.1468 | . | 1.1468 | . | . | . | PF-Sirolimus vs. PP-Sirolimus | 0 | 0 | 1.0792 | . | 1.0792 | . | . | . |
| PF-Amphilimus vs. PP-Zotarolimus | 1 | 0.74 | 1.1173 | 1.1354 | 1.0671 | 1.064 | 0.14 | 0.891 | PF-Sirolimus vs. PP-Zotarolimus | 0 | 0 | 1.2175 | . | 1.2175 | . | . | . |
| PF-Biolimus vs. PF-Sirolimus+Probucol | 0 | 0 | 0.944 | . | 0.944 | . | . | . | PF-Sirolimus vs. Probucol+PF-TiNO | 0 | 0 | 1.3035 | . | 1.3035 | . | . | . |
| PF-Biolimus vs. PF-TiNO | 0 | 0 | 1.0701 | . | 1.0701 | . | . | . | PF-Sirolimus vs. Probucol+PP-Everolimus | 0 | 0 | 0.9956 | . | 0.9956 | . | . | . |
| PF-Biolimus vs. PF-Umirolimus | 0 | 0 | 0.8611 | . | 0.8611 | . | . | . | PF-Sirolimus vs. Probucol+PP-Paclitaxel | 0 | 0 | 0.806 | . | 0.806 | . | . | . |
| PF-Biolimus vs. PP-Everolimus | 0 | 0 | 1.0007 | . | 1.0007 | . | . | . | PF-Sirolimus vs. Probucol+PP-Sirolimus | 0 | 0 | 0.8837 | . | 0.8837 | . | . | . |
| PF-Biolimus vs. PP-Paclitaxel | 0 | 0 | 0.7102 | . | 0.7102 | . | . | . | PF-Sirolimus vs. Probucol+PP-Zotarolimus | 1 | 1 | 0.997 | 0.997 | . | . | . | . |
| PF-Biolimus vs. PP-Sirolimus | 0 | 0 | 0.9563 | . | 0.9563 | . | . | . | PF-TiNO vs. PP-Everolimus | 1 | 0.32 | 0.7638 | 0.6557 | 0.8207 | 0.7989 | -0.86 | 0.3925 |
| PF-Biolimus vs. PP-Zotarolimus | 0 | 0 | 0.9317 | . | 0.9317 | . | . | . | PF-TiNO vs. PP-Paclitaxel | 1 | 0.27 | 0.6183 | 0.4866 | 0.676 | 0.7198 | -1.17 | 0.2414 |
| PF-Sirolimus + Probucol vs. PF-TiNO | 0 | 0 | 1.1336 | . | 1.1336 | . | . | . | PF-TiNO vs. PP-Sirolimus | 0 | 0 | 0.678 | . | 0.678 | . | . | . |
| PF-Sirolimus + Probucol vs. PF-Umirolimus | 0 | 0 | 0.9122 | . | 0.9122 | . | . | . | PF-TiNO vs. PP-Zotarolimus | 1 | 0.45 | 0.7649 | 0.9899 | 0.6186 | 1.6002 | 1.91 | 0.0565 |
| PF-Sirolimus + Probucol vs. PP-Everolimus | 0 | 0 | 1.06 | . | 1.06 | . | . | . | PP-Everolimus vs. PP-Paclitaxel | 2 | 0.39 | 0.8096 | 0.7559 | 0.8462 | 0.8933 | -1.22 | 0.2219 |
| PF-Sirolimus + Probucol vs. PP-Paclitaxel | 0 | 0 | 0.7524 | . | 0.7524 | . | . | . | PP-Everolimus vs. PP-Sirolimus | 2 | 0.31 | 0.8877 | 0.8405 | 0.9096 | 0.924 | -0.92 | 0.3598 |
| PF-Sirolimus + Probucol vs. PP-Sirolimus | 0 | 0 | 1.013 | . | 1.013 | . | . | . | PP-Everolimus vs. PP-Zotarolimus | 3 | 0.76 | 1.0014 | 1.0025 | 0.998 | 1.0045 | 0.04 | 0.9662 |
| PF-Sirolimus + Probucol vs. PP-Zotarolimus | 1 | 1 | 0.987 | 0.987 | . | . | . | . | PP-Paclitaxel vs. PP-Sirolimus | 1 | 0.27 | 1.0964 | 1.1247 | 1.0863 | 1.0354 | 0.31 | 0.755 |
| PF-TiNO vs. PF-Umirolimus | 0 | 0 | 0.8047 | . | 0.8047 | . | . | . | PP-Paclitaxel vs. PP-Zotarolimus | 2 | 0.35 | 1.237 | 1.1547 | 1.283 | 0.9 | -0.89 | 0.3708 |
| PF-TiNO vs. PP-Everolimus | 3 | 0.64 | 0.9351 | 0.8701 | 1.0656 | 0.8165 | -0.86 | 0.3917 | PP-Zotarolimus vs. PP-Sirolimus | 1 | 0.06 | 0.8864 | 0.9116 | 0.8847 | 1.0304 | 0.13 | 0.8997 |
| PF-TiNO vs. PP-Paclitaxel | 1 | 0.1 | 0.6637 | 0.4932 | 0.687 | 0.7179 | -0.84 | 0.4005 |  |  |  |  |  |  |  |  |  |
| PF-TiNO vs. PP-Sirolimus | 0 | 0 | 0.8937 | . | 0.8937 | . | . | . |  |  |  |  |  |  |  |  |  |
| PF-TiNO vs. PP-Zotarolimus | 1 | 0.3 | 0.8707 | 1.1323 | 0.7788 | 1.4538 | 1.47 | 0.1424 |  |  |  |  |  |  |  |  |  |
| PF-Umirolimus vs. PP-Everolimus | 0 | 0 | 1.1621 | . | 1.1621 | . | . | . |  |  |  |  |  |  |  |  |  |
| PF-Umirolimus vs. PP-Paclitaxel | 0 | 0 | 0.8248 | . | 0.8248 | . | . | . |  |  |  |  |  |  |  |  |  |
| PF-Umirolimus vs. PP-Sirolimus | 0 | 0 | 1.1105 | . | 1.1105 | . | . | . |  |  |  |  |  |  |  |  |  |
| PF-Umirolimus vs. PP-Zotarolimus | 1 | 1 | 1.082 | 1.082 | . | . | . | . |  |  |  |  |  |  |  |  |  |
| PP-Everolimus vs. PP-Paclitaxel | 5 | 0.43 | 0.7098 | 0.618 | 0.7877 | 0.7845 | -2.36 | 0.0183 |  |  |  |  |  |  |  |  |  |
| PP-Everolimus vs. PP-Sirolimus | 6 | 0.28 | 0.9557 | 0.9023 | 0.9772 | 0.9234 | -0.73 | 0.4632 |  |  |  |  |  |  |  |  |  |
| PP-Everolimus vs. PP-Zotarolimus | 4 | 0.39 | 0.9311 | 0.965 | 0.9097 | 1.0608 | 0.64 | 0.5254 |  |  |  |  |  |  |  |  |  |
| PP-Paclitaxel vs. PP-Sirolimus | 7 | 0.34 | 1.3464 | 1.2106 | 1.4219 | 0.8514 | -1.36 | 0.1724 |  |  |  |  |  |  |  |  |  |
| PP-Paclitaxel vs. PP-Zotarolimus | 4 | 0.34 | 1.3118 | 1.2888 | 1.3236 | 0.9737 | -0.23 | 0.8177 |  |  |  |  |  |  |  |  |  |
| PP-Zotarolimus vs. PP-Sirolimus | 4 | 0.2 | 1.0264 | 1.1263 | 1.0027 | 1.1233 | 0.86 | 0.3905 |  |  |  |  |  |  |  |  |  |

**Supplemental Table 18: Q-statistics, degree of freedom (df), and p-value for inconsistency model at different follow up durations**

| 6-Months | Q | df | p-value | Q | df | p-value | Q | df | p-value | Q | df | p-value |
| --- | --- | --- | --- | --- | --- | --- | --- | --- | --- | --- | --- | --- |
| Inconsistency | MACE | | | Mortality | | | Cardiovascular Death | | | Stent Thrombosis | | |
| Total | 4.28 | 9 | 0.8921 | 8.97 | 15 | 0.8791 | 4.22 | 12 | 0.979 | 7.22 | 12 | 0.8425 |
| Within designs | 3.19 | 5 | 0.6714 | 5.32 | 9 | 0.806 | 3.36 | 7 | 0.8502 | 3.3 | 7 | 0.8558 |
| Between designs | 1.09 | 4 | 0.8953 | 3.65 | 6 | 0.7233 | 0.87 | 5 | 0.9725 | 3.92 | 5 | 0.5607 |
| Inconsistency | Myocardial Infarction | | | Target Lesion Revascularization | | | Target Vessel Revascularization | | |  | | |
| Total | 13.92 | 20 | 0.8344 | 5.25 | 16 | 0.9943 | 4.77 | 13 | 0.9799 |  |  |  |
| Within designs | 12.24 | 13 | 0.5078 | 3.52 | 10 | 0.9663 | 2.7 | 8 | 0.9519 |  |  |  |
| Between designs | 1.68 | 7 | 0.9755 | 1.73 | 6 | 0.9428 | 2.07 | 5 | 0.8389 |  |  |  |
| 1-Year | Q | df | p-value | Q | df | p-value | Q | df | p-value | Q | df | p-value |
| Inconsistency | MACE | | | Mortality | | | Cardiovascular Death | | | Myocardial Infarction | | |
| Total | 84.89 | 84 | 0.4524 | 51.87 | 81 | 0.9951 | 36.2 | 71 | 0.9998 | 59.17 | 81 | 0.9675 |
| Within designs | 63.89 | 64 | 0.4802 | 44.5 | 62 | 0.9543 | 28.13 | 51 | 0.9962 | 33.28 | 61 | 0.9985 |
| Between designs | 20.99 | 20 | 0.3977 | 7.38 | 19 | 0.9919 | 8.07 | 20 | 0.9914 | 25.89 | 20 | 0.1695 |
| Inconsistency | TLR | | | TVR | | | Stent Thrombosis | | | Stroke | | |
| Total | 130.75 | 73 | 0.01 | 79.68 | 70 | 0.2008 | 42.03 | 44 | 0.5565 | 5.17 | 8 | 0.7397 |
| Within designs | 98.02 | 53 | 0.0002 | 64.7 | 52 | 0.1112 | 26.34 | 27 | 0.4999 | 3.38 | 3 | 0.3369 |
| Between designs | 32.72 | 20 | 0.0362 | 14.98 | 18 | 0.6636 | 15.69 | 17 | 0.546 | 1.79 | 5 | 0.8777 |
| 5-Year | Q | df | p-value | Q | df | p-value | Q | df | p-value | Q | df | p-value |
| Inconsistency | Mortality | | | Cardiovascular Death | | | Myocardial Infarction | | |  | | |
| Total | 37.92 | 29 | 0.124 | 25.62 | 32 | 0.7801 | 22.51 | 32 | 0.8931 |  |  |  |
| Within designs | 25.08 | 17 | 0.0929 | 14.38 | 20 | 0.8109 | 8.05 | 20 | 0.9915 |  |  |  |
| Between designs | 12.84 | 12 | 0.3807 | 11.25 | 12 | 0.5079 | 14.46 | 12 | 0.2724 |  |  |  |
| Inconsistency | TLR | | | TVR | | | Stent Thrombosis | | |  | | |
| Total | 23.9 | 30 | 0.7763 | 19.29 | 25 | 0.783 | 20.55 | 29 | 0.875 |  |  |  |
| Within designs | 12.53 | 19 | 0.8619 | 11.91 | 14 | 0.6136 | 11.96 | 20 | 0.9176 |  |  |  |
| Between designs | 11.38 | 11 | 0.4121 | 7.38 | 11 | 0.7674 | 8.6 | 9 | 0.4755 |  |  |  |

**Supplemental Table 19: Previous meta-analysis on the efficacy of different stent comparisons and their limitations [Trial sequential analysis (TSA), Heterogeneity (H), Network (NW), Meta-Regression (MR), Subgroups (SG)]**

| **Author** | **Country** | **Year** | **Comparison arms** | **Studies** | **Model** | **SG** | **NW** | **MR** | **H** | **TSA** | **Limitation** |
| --- | --- | --- | --- | --- | --- | --- | --- | --- | --- | --- | --- |
| Ouyang (197) | China | 2021 | EES vs. SES or PES | 8 | Random | No | No | No | Yes | No | <10 Studies, No funnel plot |
| Deng (198) | China | 2020 | SES vs. EES | 5 | Both | No | No | No | No | No | Late stent thrombosis could not be assessed. No data on antiplatelet therapy |
| Monjur (199) | Australia | 2020 | PA vs. PP | 10 | Random | No | No | Yes | No | Yes | Only 1-year follow-up. No ACS subgroup. Underpowered. |
| Ke (200) | China | 2020 | Scaffolds vs. DES | 6 | Random | No | No | No | No | No | No data on device success. Only 1 type of bioresorbable scaffold data |
| Ni (201) | China | 2020 | BVS vs DES | 10 | Fixed | No | No | No | No | No | Limited no. of BVS studies in outcome analysis. Many studies included ‘Absorb BVS’ device |
| Chen (202) | China | 2019 | PF-DES vs. PP-DES | 17 | Both | Yes | No | No | Yes | No | Not performed at individual level. |
| Wu (203) | Australia | 2019 | PF-DES vs. PP-DES | 13 | Random | Yes | No | No | No | No | No description of randomization techniques-difficult to access the risk of bias. |
| Shah (204) | USA | 2018 | DES vs BMS | 3 | Random | No | No | No | Yes | No | <10 studies, Only PF or PA-DES. |
| Wu (205) | Australia | 2018 | PA vs. PP | 19 | Fixed | No | No | No | No | No | Median follow up of 12 months. |
| Zhu (206) | China | 2018 | PA vs. PP | 6 | Random | Yes | No | No | No | Yes | <10 studies, Only 1-year follow-up, No data on antiplatelet therapy. |
| Kheiri (207) | USA | 2018 | DES vs. BMS | 6 | Random | No | No | No | Yes | No | <10 studies, small sample size in 3 studies, No consistency on MACE definition |
| Felix (208) | Netherlands | 2018 | BVS vs. DES | 10 | Random | No | No | No | No | Yes | Unpublished data included, confounding bias, median follow up is <2years |
| Mahmoud (209) | USA | 2018 | DES vs. BMS | 9 | Random | No | No | Yes | Yes | No | Inconsistency of MACE definition, confound bias due to differential duration of DAPT. |
| Bundhun (210) | China | 2017 | PA vs. PP | 12 | Both | No | No | No | Yes | No | Small sample size, Lack of heterogeneity. |
| Lu (211) | China | 2017 | PA-BES vs. PP-DES | 5 | Fixed | No | No | No | No | No | <10 studies, heterogeneity and confounding bias |
| Bundhun (212) | China | 2017 | ZES vs. SES, PES and EES | 12 | Both | No | No | No | Yes | Yes | Selection bias, publication bias. |
| Gao (213) | China | 2017 | PF vs. PP - DES | 11 | Both | No | No | No | Yes | No | Underpowered, heterogeneity, small sample size. |
| Bundhun (214) | China | 2017 | PA-DES vs PP-EES | 10 | Fixed | No | No | No | No | No | Small sample size in each study, no subgroup analysis. |
| Bundhun (215) | China | 2017 | ZES vs. EES | 6 | Fixed | No | No | No | No | No | <10 studies, small sample size, inconsistent followup period, heterogeneity, |
| Lu (216) | China | 2016 | DES vs. BMS | 38 | Random | No | No | Yes | Yes | No | Only 1 true RCT was included, publication bias, heterogeneity. |
| Ferko (217) | Canada | 2017 | EES vs. BMS |  |  | No | No |  |  |  | Uncertainty over duration of DAPT for 12 months. |
| Bundhun (218) | China | 2016 | SES vs non-SE DES | 29 | Both | No | No | No | Yes | No | Observational studies included, no subgroup analysis. |
| Bundhun (219) | China | 2016 | DES vs. BMS | 10 | Fixed | No | No | No | No | No | Small sample size, fewer outcomes, shorter follow up |
| Yan (220) | China | 2016 | DES vs. BMS | 49 | Random | No | No | No | No | No | Selection bias, confounding bias related to duration of DAPT. |
| Pandya (221) | USA | 2016 | PA-DES vs. PP-DES | 11 | Random | No | No | No | No | No | Variable followup periods, no subgroup analysis, short follow-up |
| Wu (222) | China | 2016 | PF-DES vs. PP-DES | 10 | Both | No | No | No | No | No | No subgroup analysis, heterogeneity, variable follow-up |
| Meng (223) | China | 2016 | EES vs. PES | 3 | Both | No | No | No | Yes | No | <10 studies, heterogeneity, study bias |
| Lv (224) | China | 2015 | PA-DES vs. PP-DES | 16 | Both | No | No | No | Yes | No | Broad range of followup periods. |
| Wang (225) | China | 2015 | PA-DES vs. PP-DES | 16 | Both | Yes | No | No | Yes | No | Lacked specific targets, variable followup periods. |
| Wu (226) | China | 2015 | 1st gen DES vs. 2nd gen DES | 5 | Random | No | No | No | No | No | <10 studies, underpowered subgroup analysis. |
| Navarese (227) | Italy, Germany | 2014 | 1st gen DES vs. 2nd gen DES | 33 | Both | No | No | No | Yes | No | Heterogeneous definitions of MI across various studies, variable duration of DAPT. |
| Yin (228) | China | 2014 | PA-DES vs BMS | 7 | Fixed | No | No | No | Yes | No | <10 studies, underpowered. |
| Zhang (229) | China | 2014 | SES vs. PES | 76 | Both | Yes | No | No |  | No | selective reporting bias, variable MACE definition, observational studies were included. |
| Yan (230) | China | 2014 | 1st gen DES vs. 2nd gen DES | 10 | Random | No | No | No | Yes | No | Small sample size, underpowered subgroup analysis. |
| Kwong (231) | Hong Kong | 2014 | PA-DES vs. PP-DES | 20 | Random | No | No | No | No | No | Publication bias, no subgroup analysis. |
| Ye (232) | China | 2013 | PA-BES vs. PP-DES | 8 | Both | No | No | Yes |  | No | <10 studies, underpowered subgroup analysis. |
| Bangalore (233) | USA | 2013 | PA-DES vs. BMS vs. PP-DES | 126 | Random | No | Yes | No |  | No | Indirect comparison only. Different sample sizes of comparison groups |
| Navarese (234) | Poland | 2013 | PP-DES vs. PA-BES | 60 | Random | No | Yes | Yes |  | No | Variable duration of DAPT, limited studies with ZES |
| Ullah (235) | USA | 2021 | PF-DES vs. PC-DES | 28 | Both | Yes | No | Yes | No | No | No subgroup analysis on strut thickness of stents and PA-DES vs PP-DES |

**Supplemental Figure 1: PRISMA flow diagram of the included studies**

**
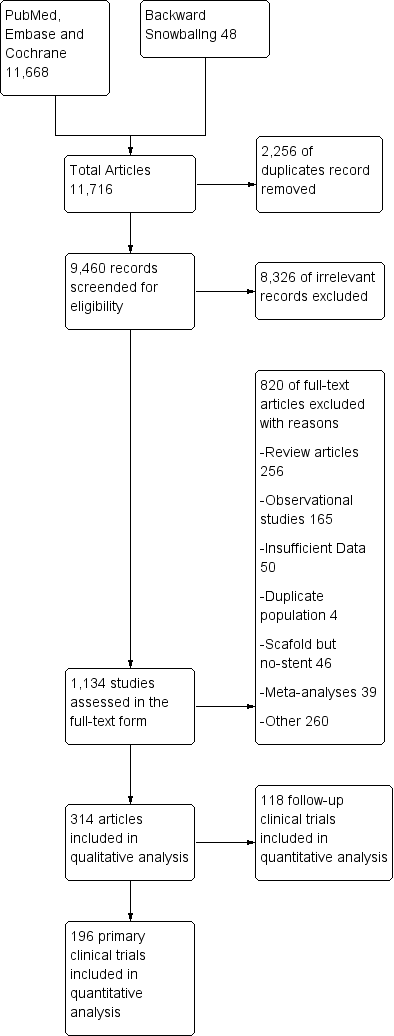
**

**Supplemental Figure 2: Flow diagram of the contribution of different stent design comparisons to the pooled network analysis.**

**
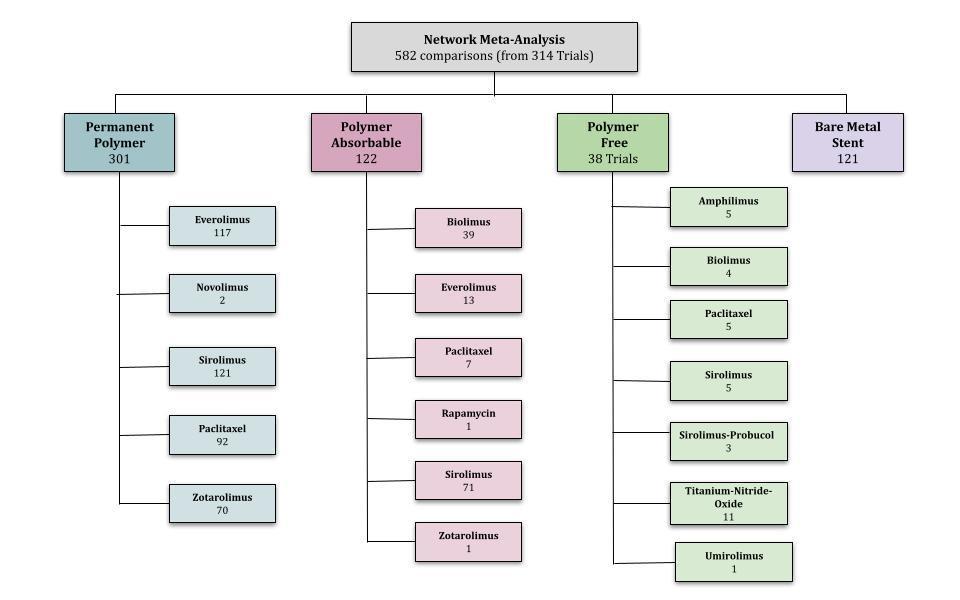
**

**Supplemental Figure 3: Splitwise interval analysis showing the contribution of direct and indirect estimates of MACE at the level of major comparisons, indicating that network estimates were not influenced by indirect comparisons (not significant).**

**
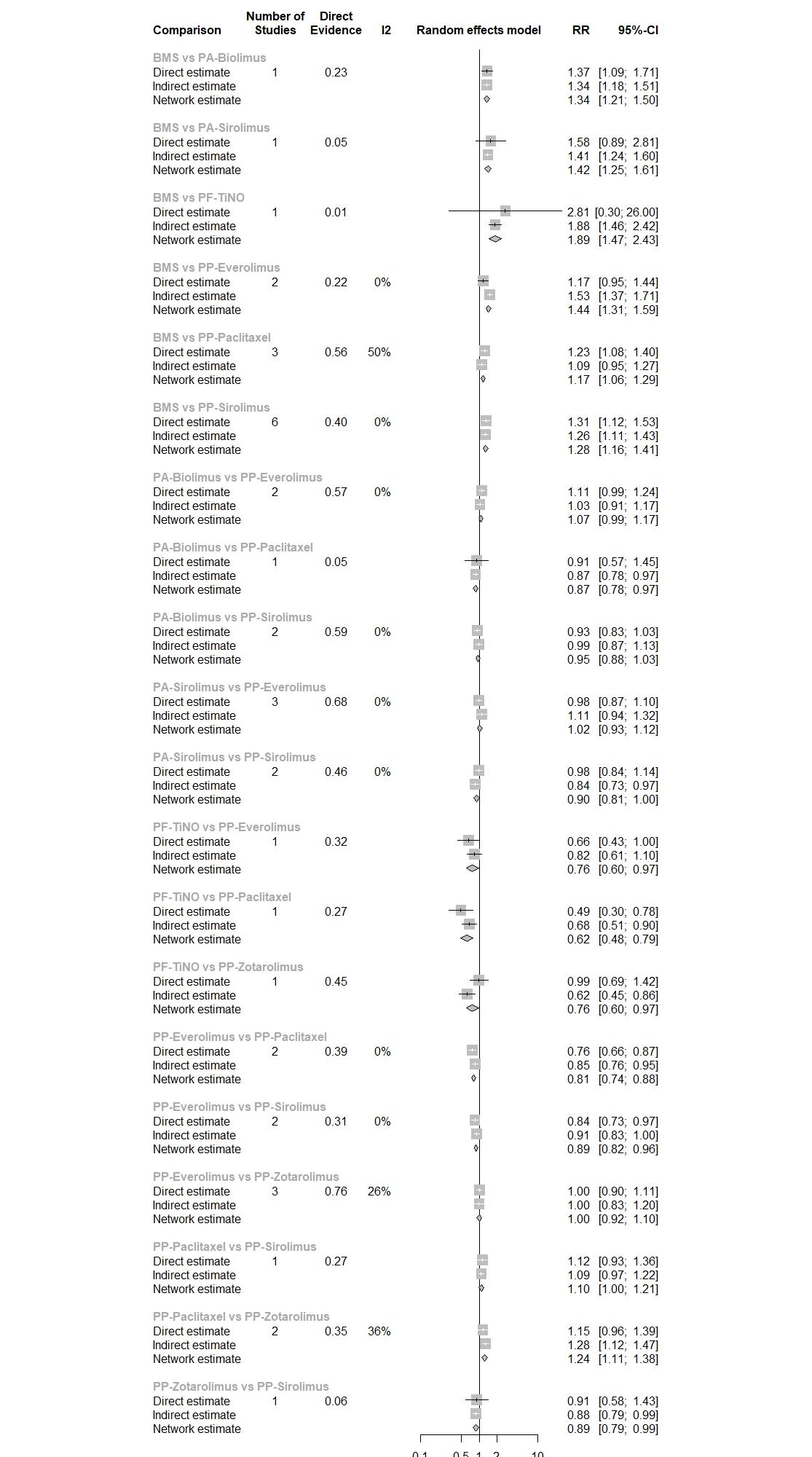
**

**Supplemental Figure 4: Direct evidence plot shows that several estimates in our network model for MACE were inferred by indirect evidence. The minimal parallelism indicates the number of different sources that contribute in parallel to the net effect size and mean path length of 2 means that indirect evidence had an important role.**

**
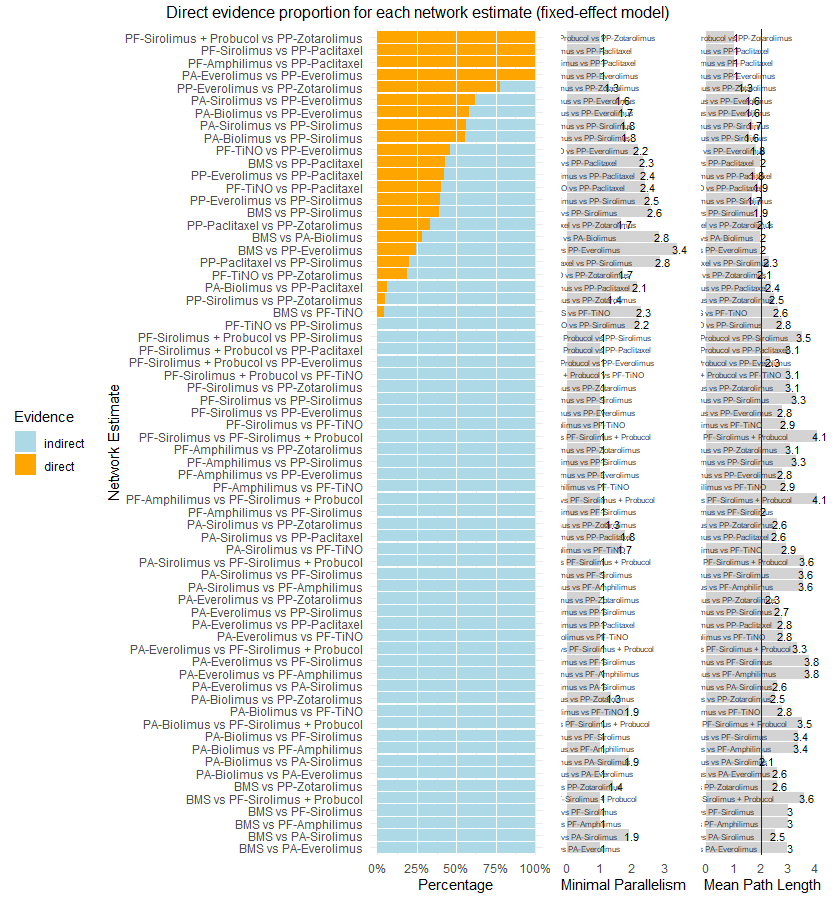
**

**Supplemental Figure S5: Network forest plots for comparison of all treatment strategies in comparison with PP-SES in all-comers at 6-months.**

**
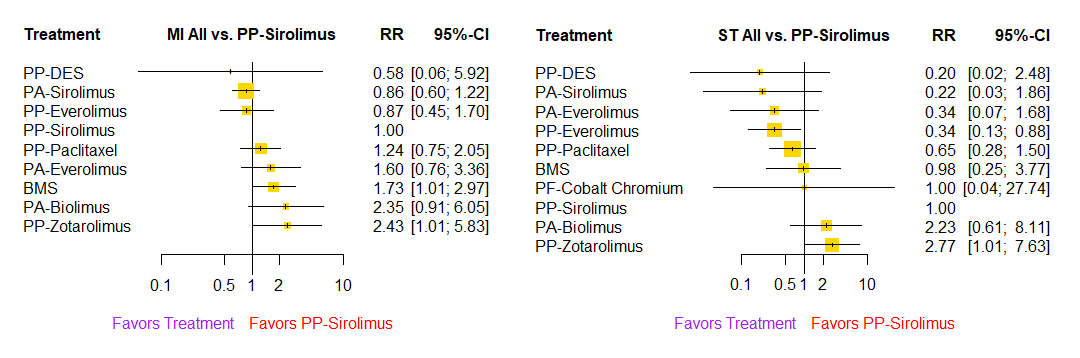
**

**
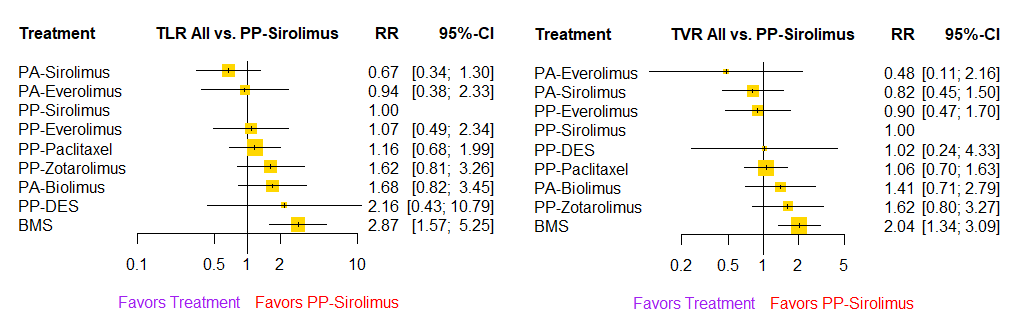
**

**
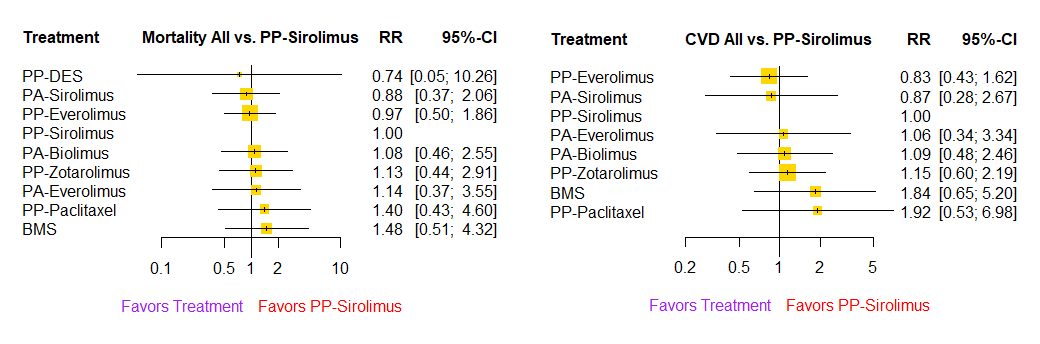
**

**Supplemental Figure 6: Network forest plots for comparison of all treatment strategies in comparison with PP-SES in all-comers at 12-months.**

**
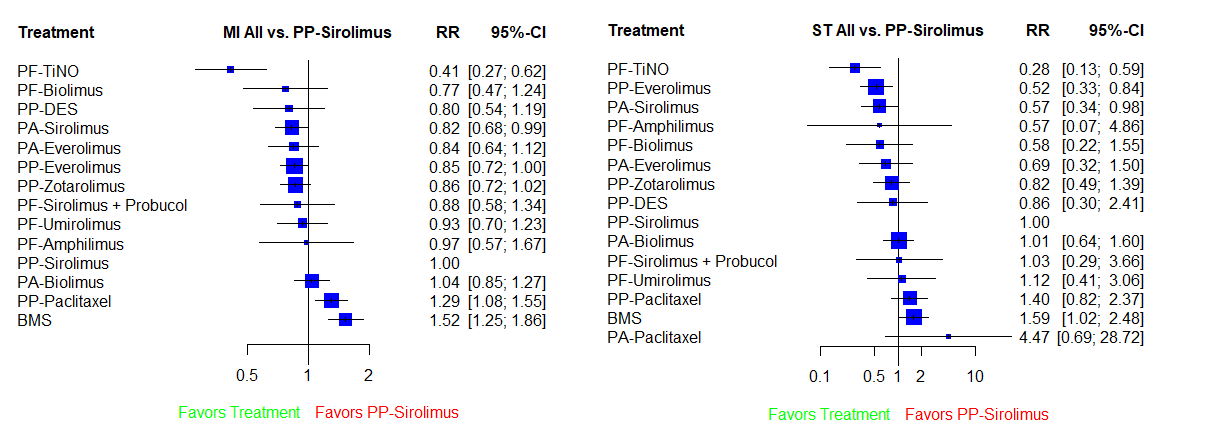
**

**
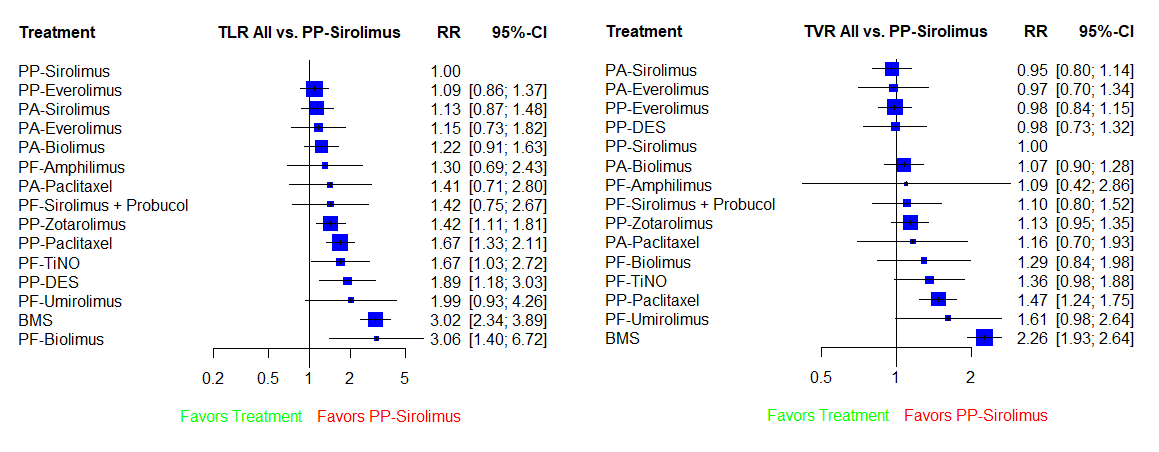
**

**
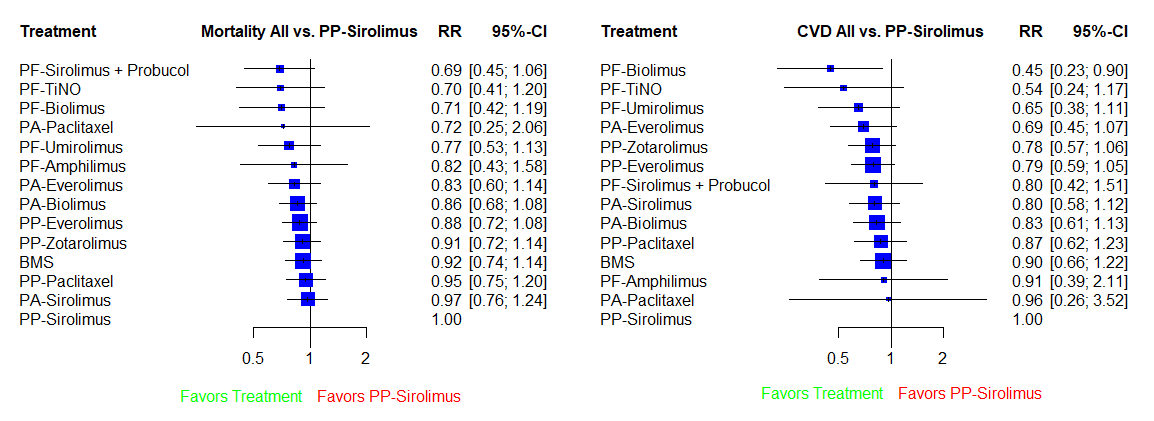
**

**
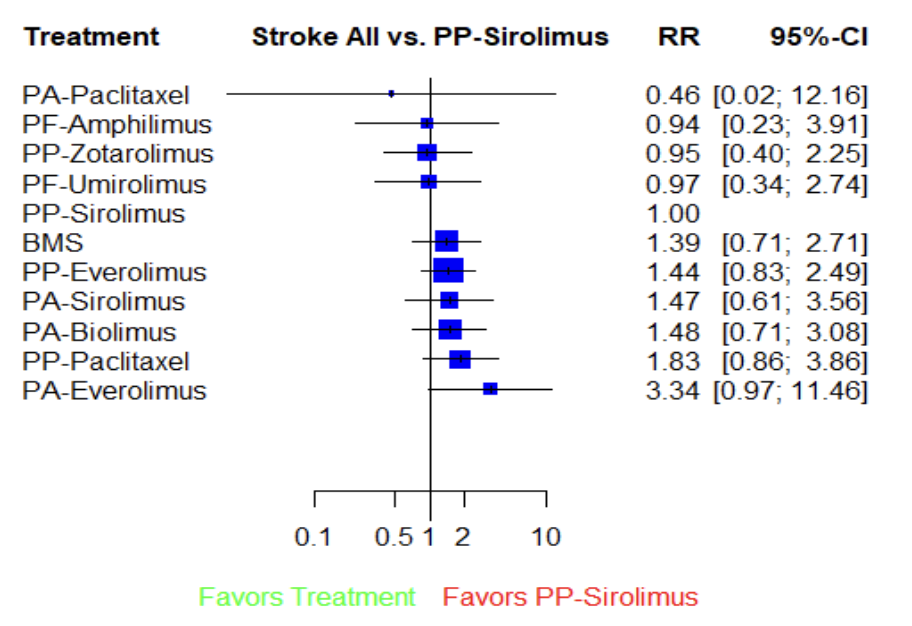
**

**Supplemental Figure 7: Network forest plots for comparison of all treatment strategies in comparison with PP-SES in all-comers at 5-years.**

**
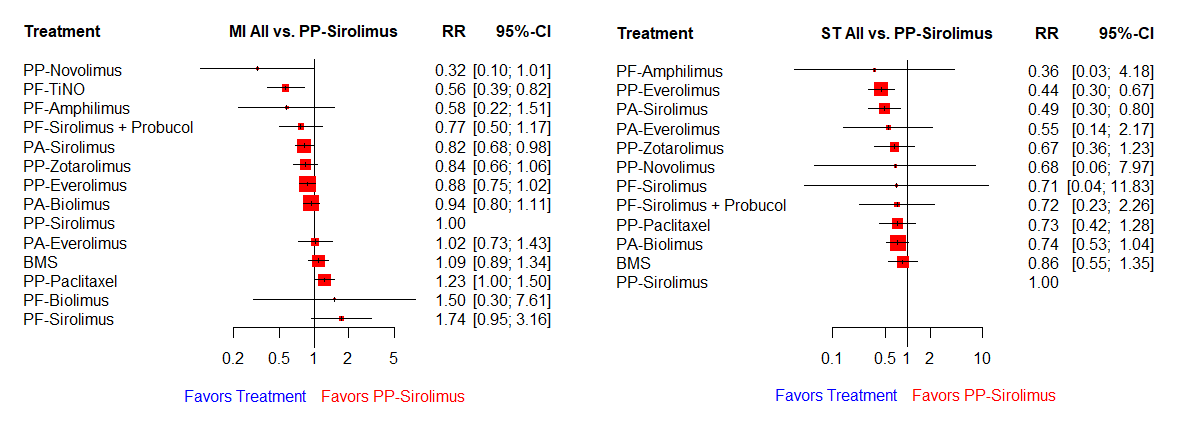
**

**
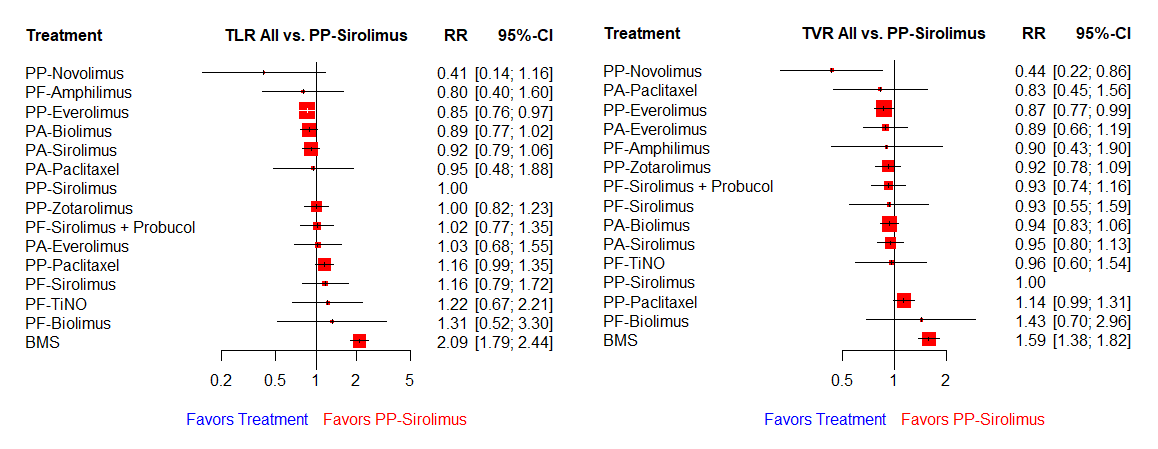
**

**
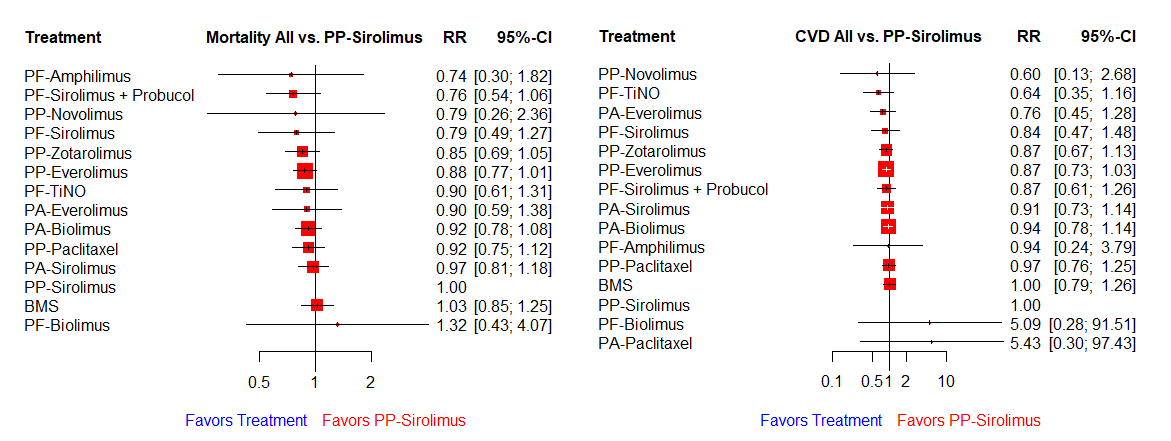
**

**Supplemental Figure 8: Network forest plots for comparison of all treatment strategies in comparison with PP-SES in patients receiving DAPT for 6-months.**

**
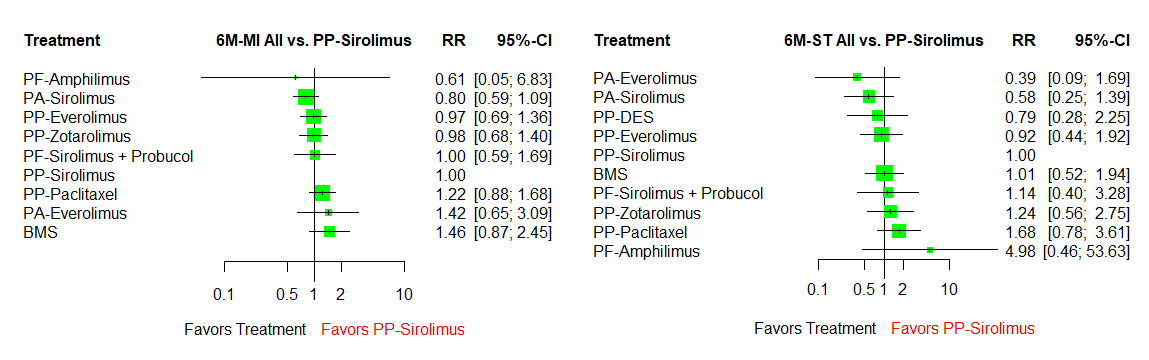
**

**
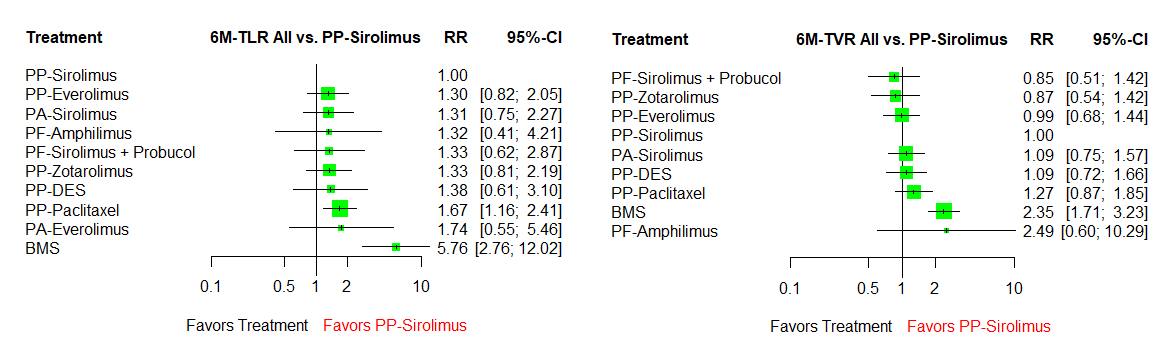
**

**
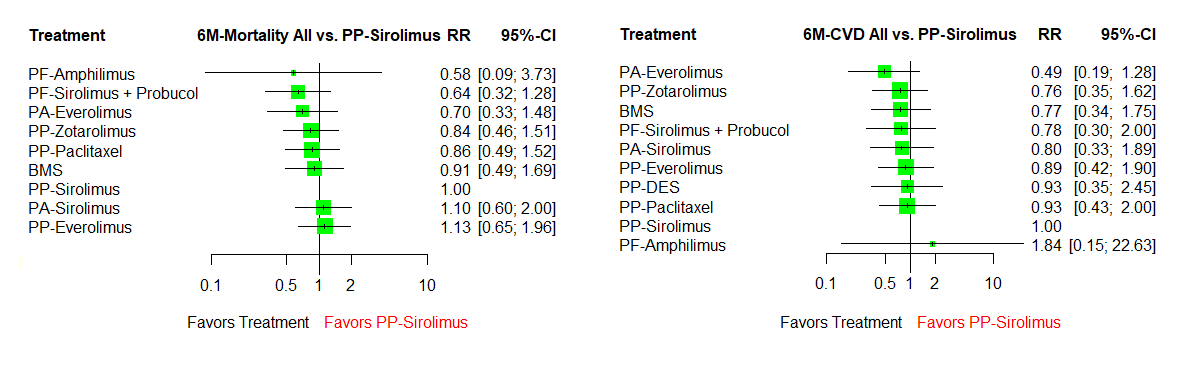
**

**Supplemental Figure 9: Network forest plots for comparison of all treatment strategies in comparison with PP-SES in patients receiving DAPT for 12-months.**

**
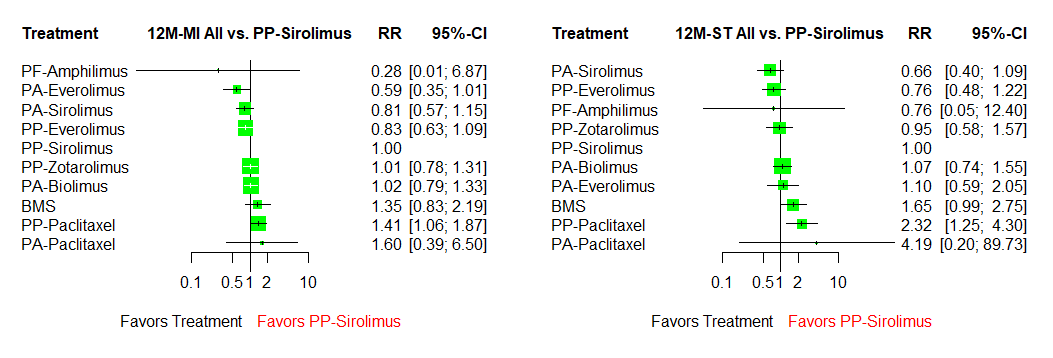
**

**
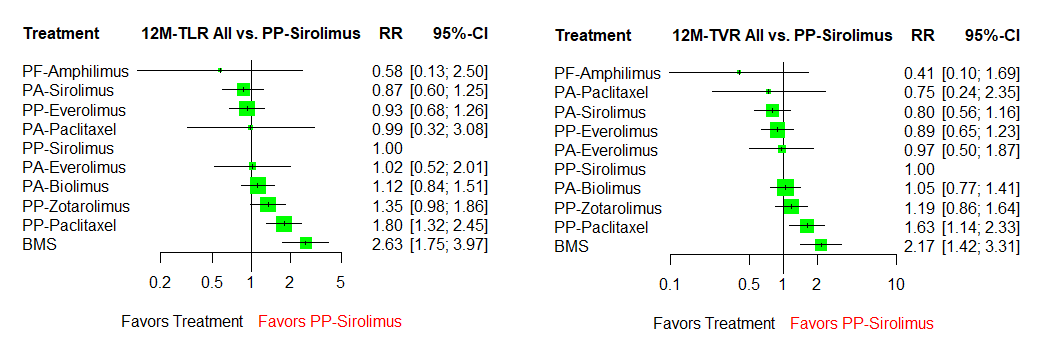
**

**
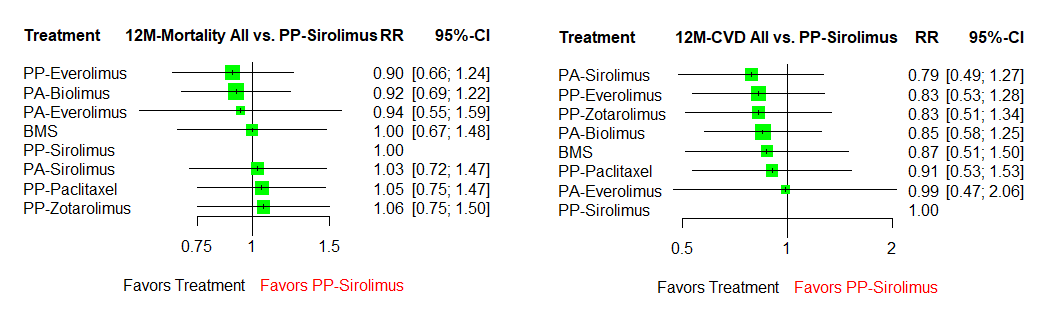
**

**Supplemental Figure 10: Network forest plots for comparison of all treatment strategies at 12-months, in comparison with PP-SES in patients presenting with ACS.**

**
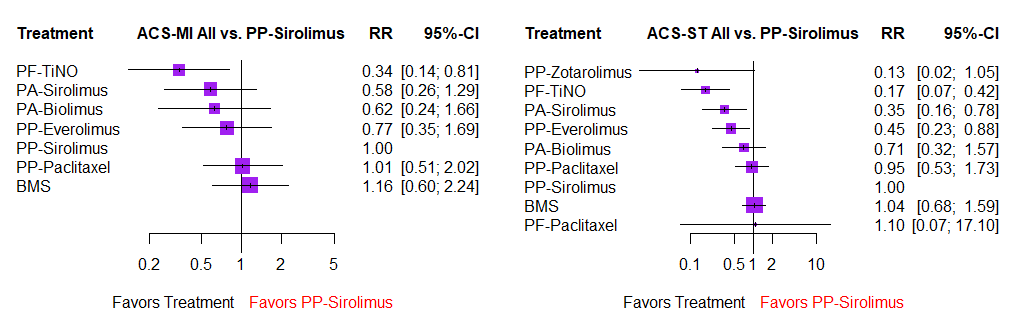
**

**
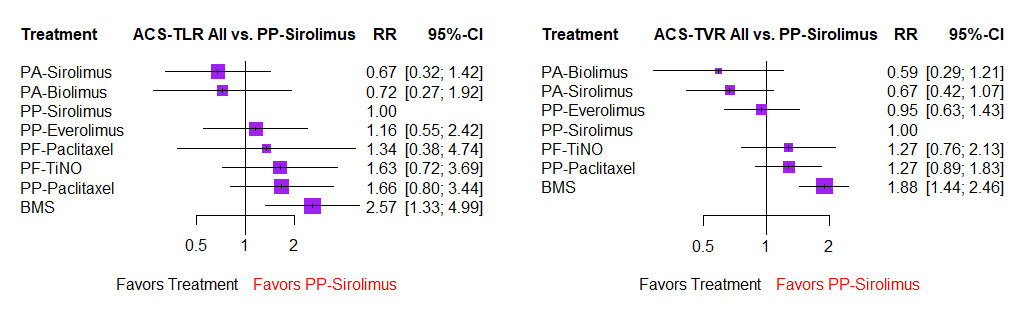
**

**
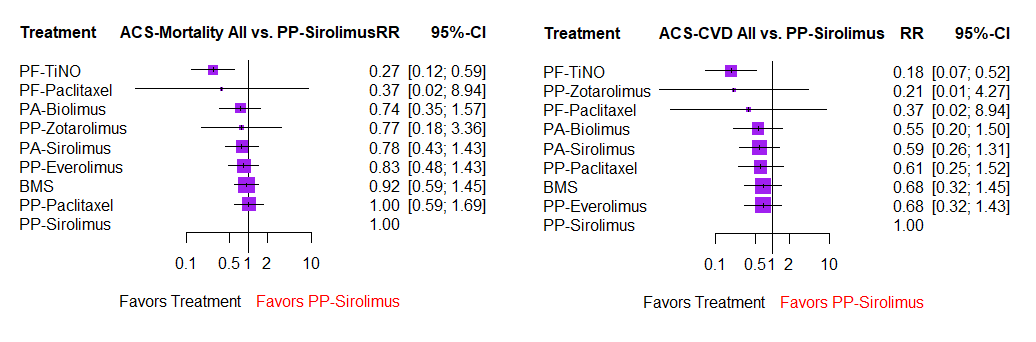
**

**Supplemental Figure 11: Rankograms showing the performance of different treatment strategies at a follow-up of 12 months. A p-score of closer to 1 indicates the best performance.**

**
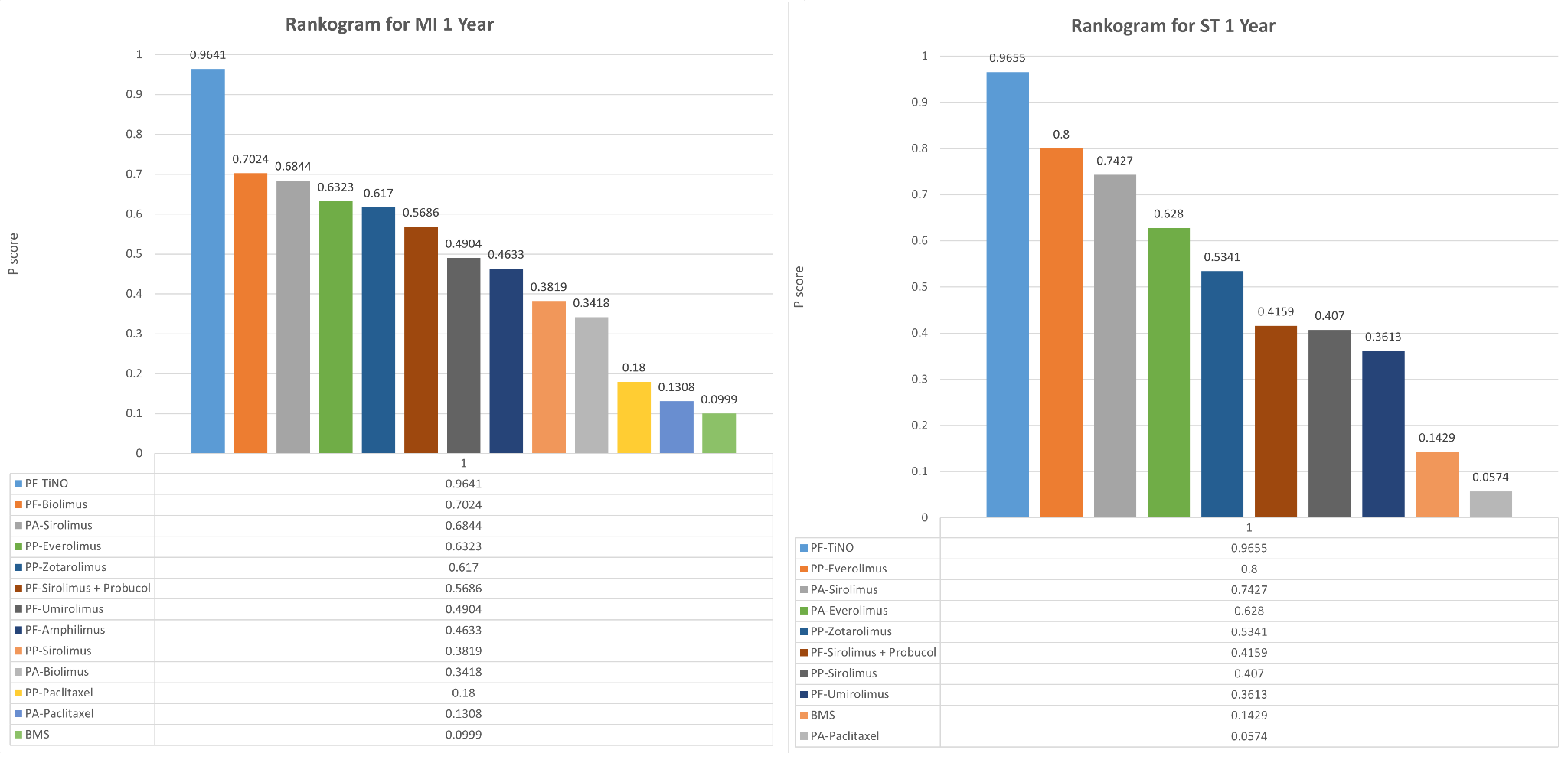
**

**
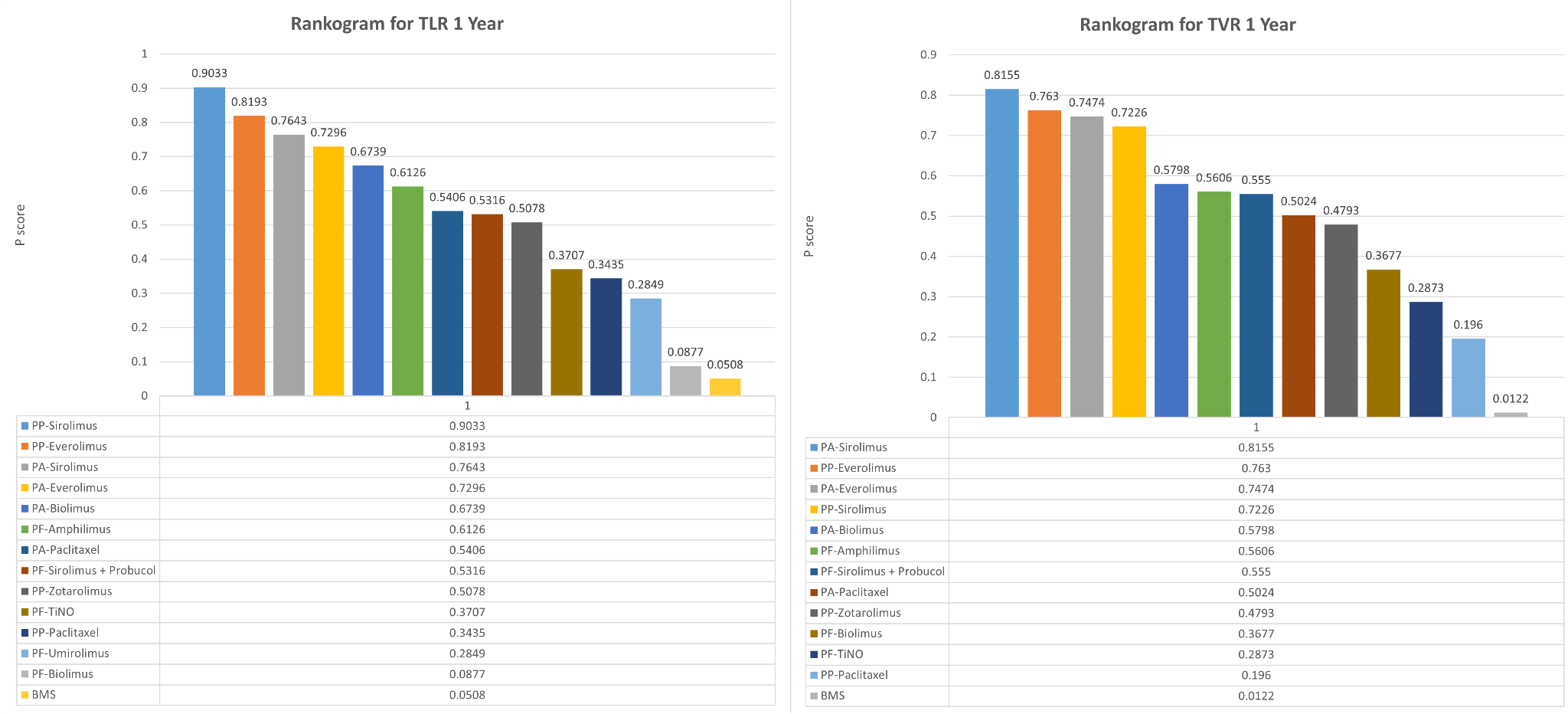
**

**
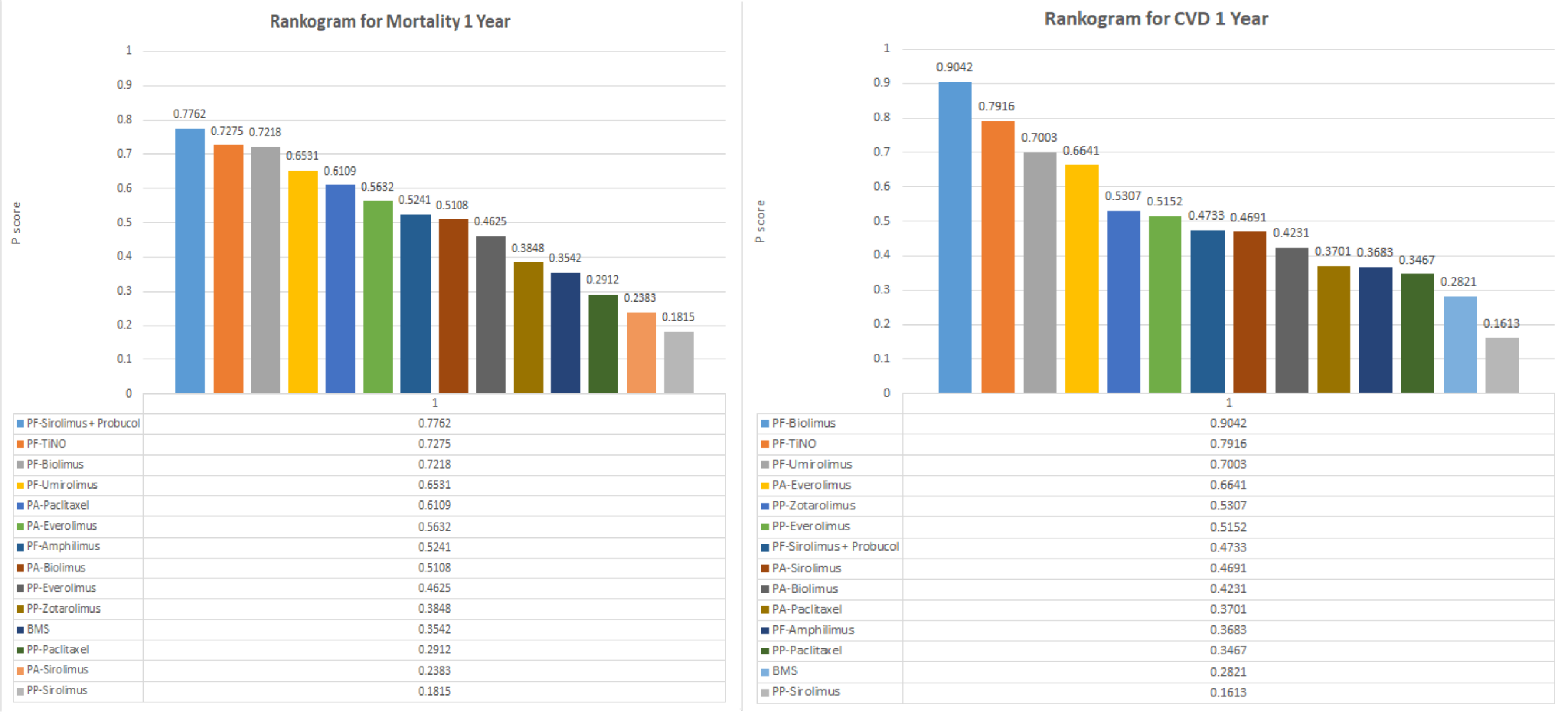
**

**
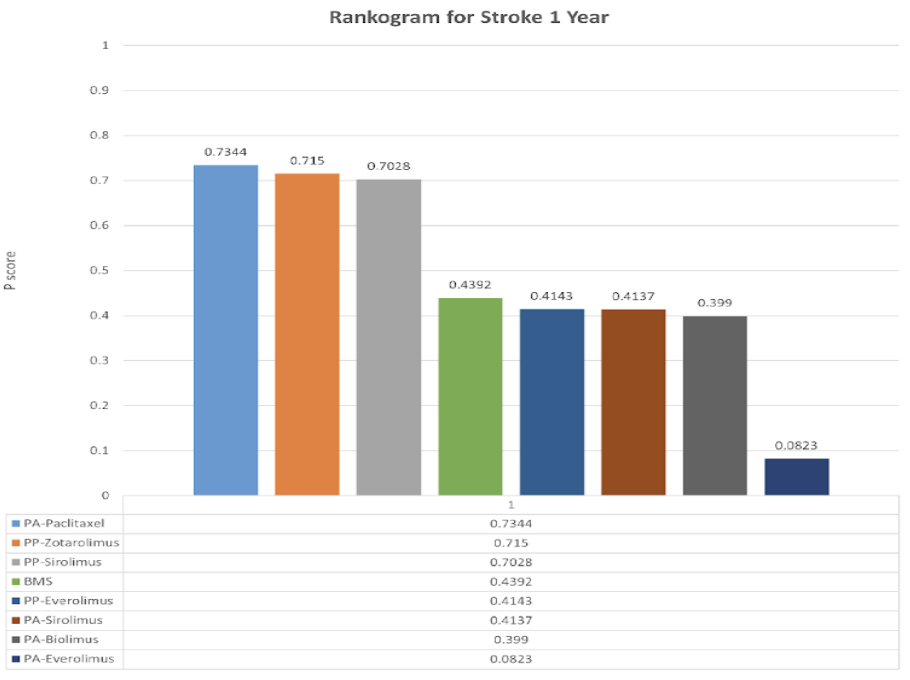
**

**Supplemental Figure 12: Rankograms showing the performance of different treatment strategies at a follow-up of 5-years. A p-score of closer to 1 indicates the best performance.**

**
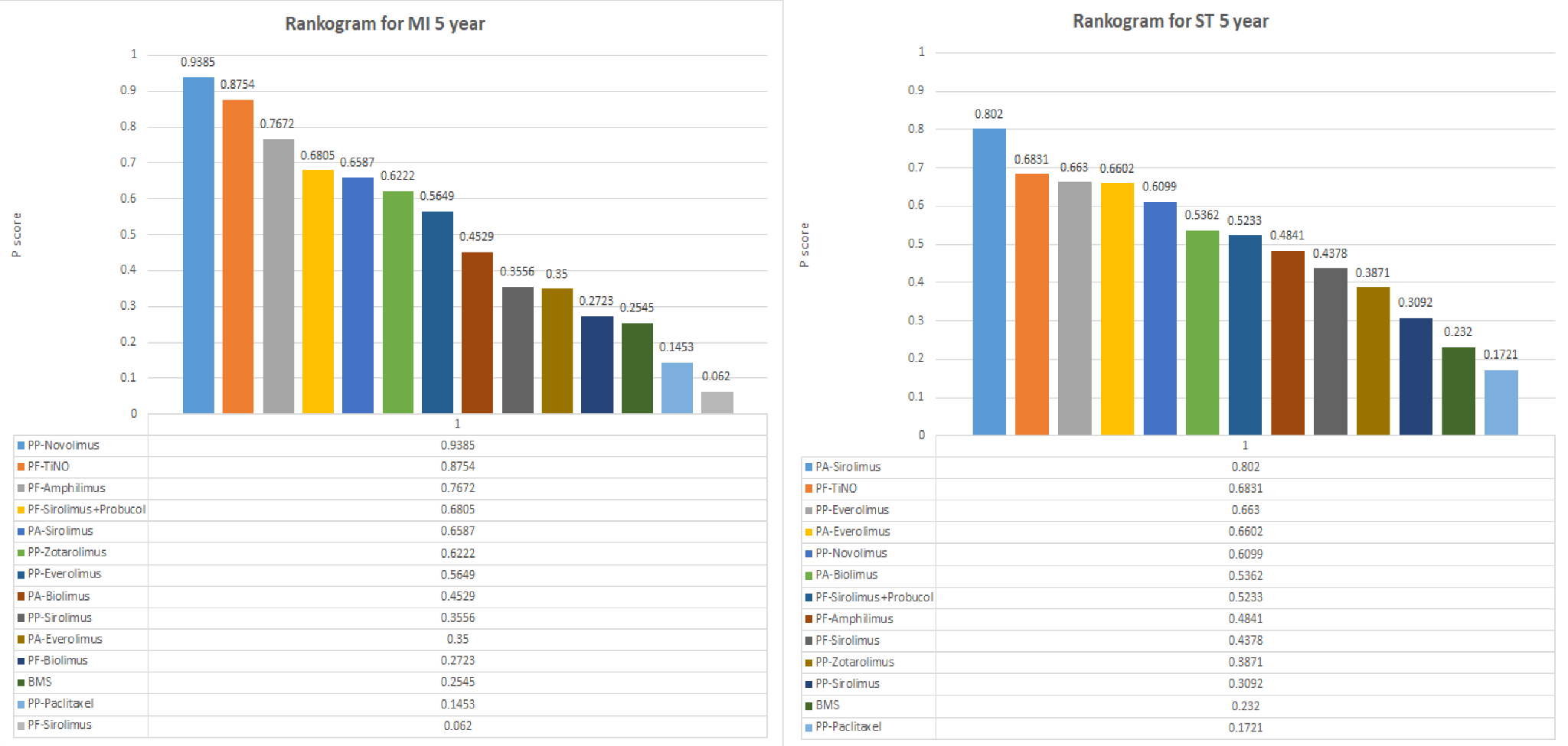
**

**
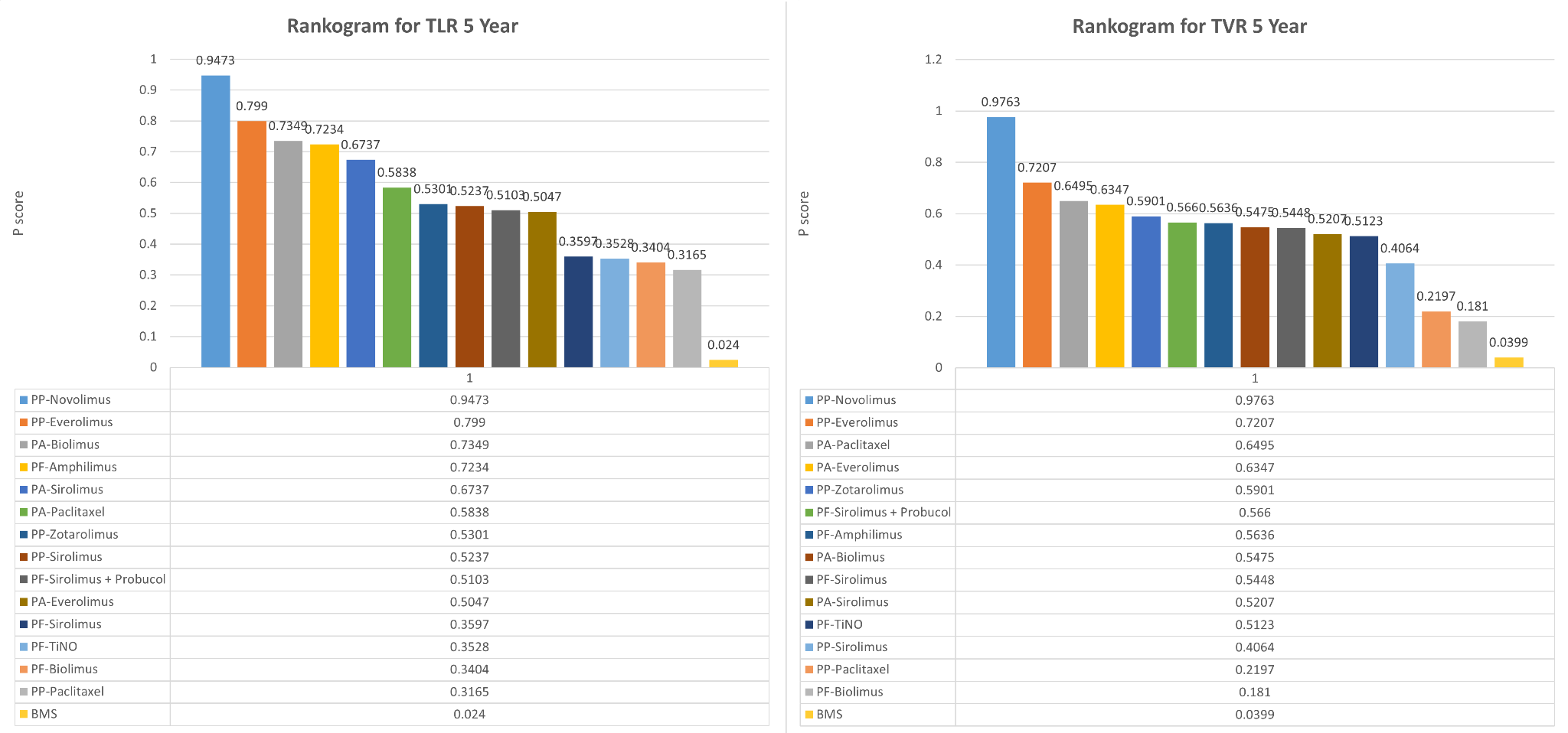
**

**
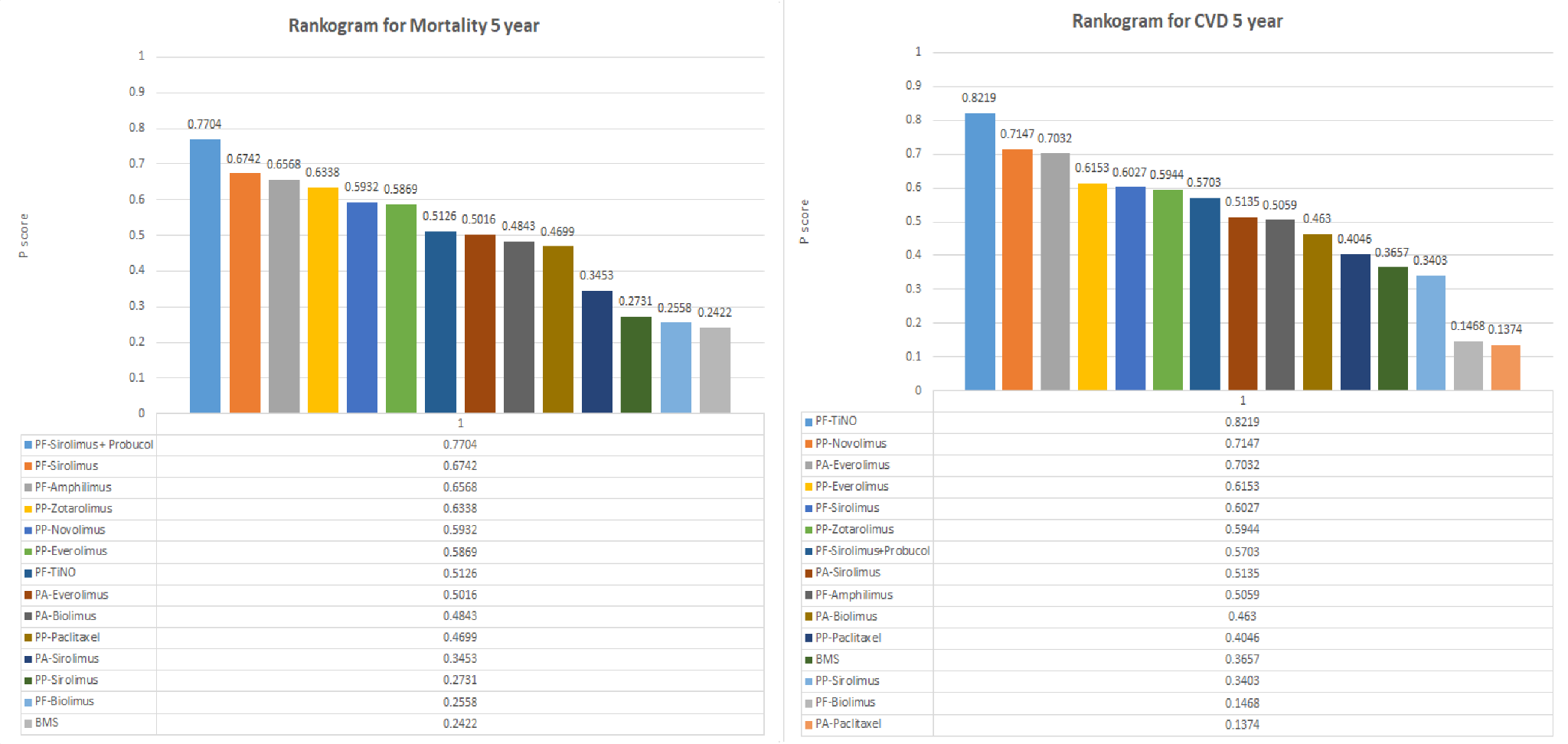
**

**Supplemental Figure 13: L’Abbe plot showing no evidence of heterogeneity in comparison of MACE and mortality at 1-year indicated by close distribution of studies to the equality line.**

**
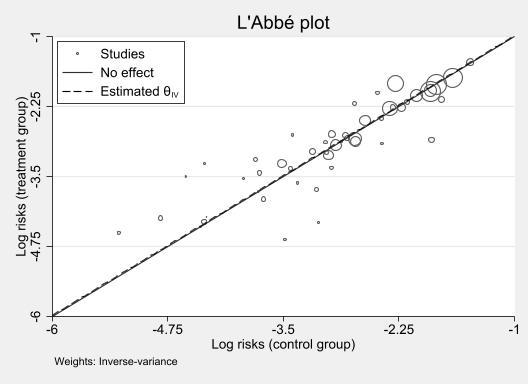

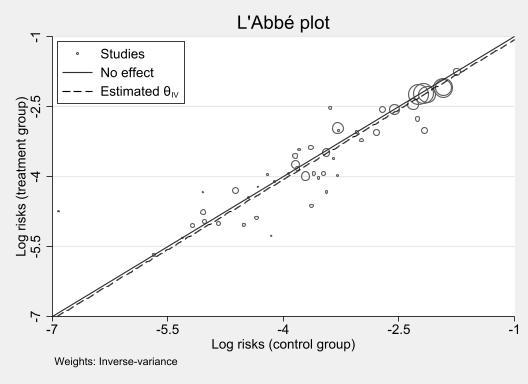
**

**Supplemental Figure 14: Net heatmap showing minimal inconsistency in the network estimates of MACE as indicated by predominantly yellow and gray boxes (red indicates higher inconsistency) and large gray boxes in the diagonal of the heatmap means that direct evidence was appropriately used to assess design-level estimates.**

**
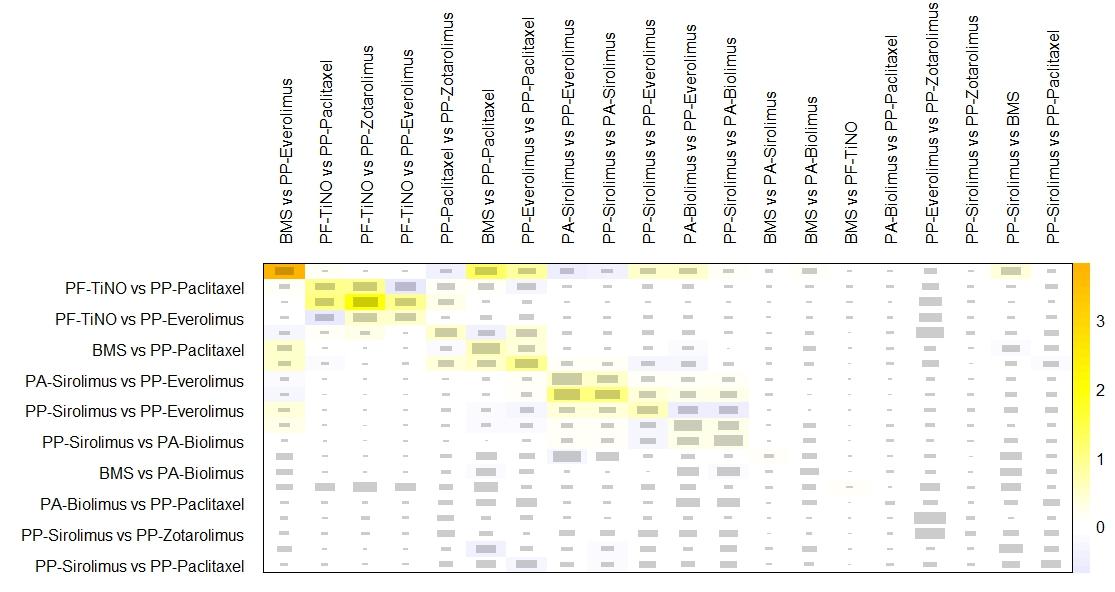
**

**Supplemental Figure 15: Sensitivity analysis showing no evidence of influence of any one trial in comparison of MACE and mortality at 1-year.**

**MACE at 1-year**

**
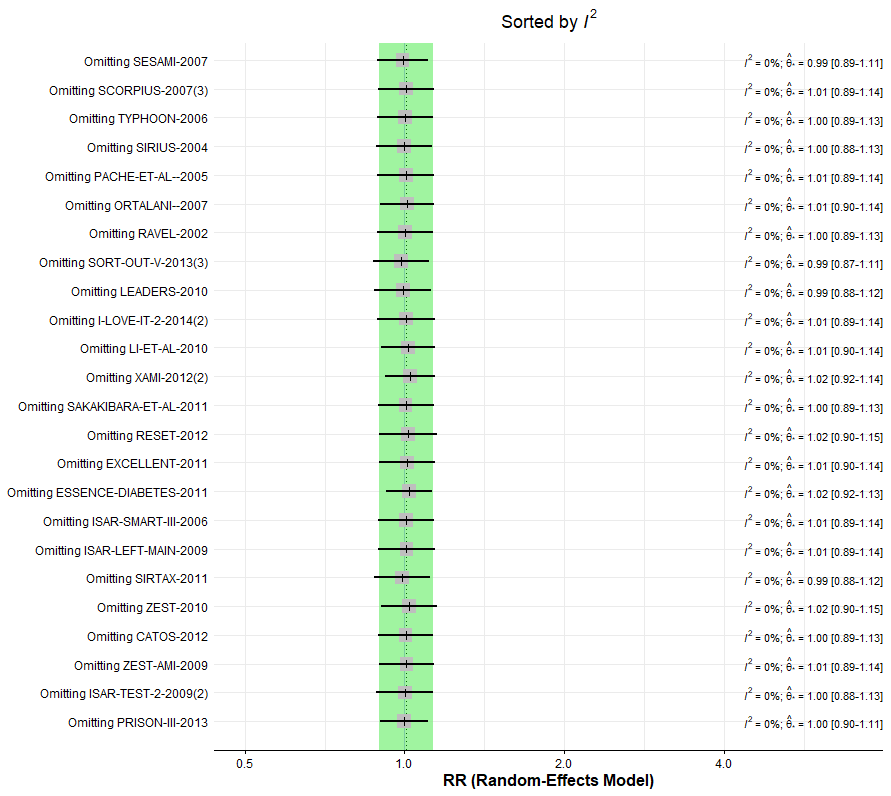
**

**Mortality at 1-year**

**
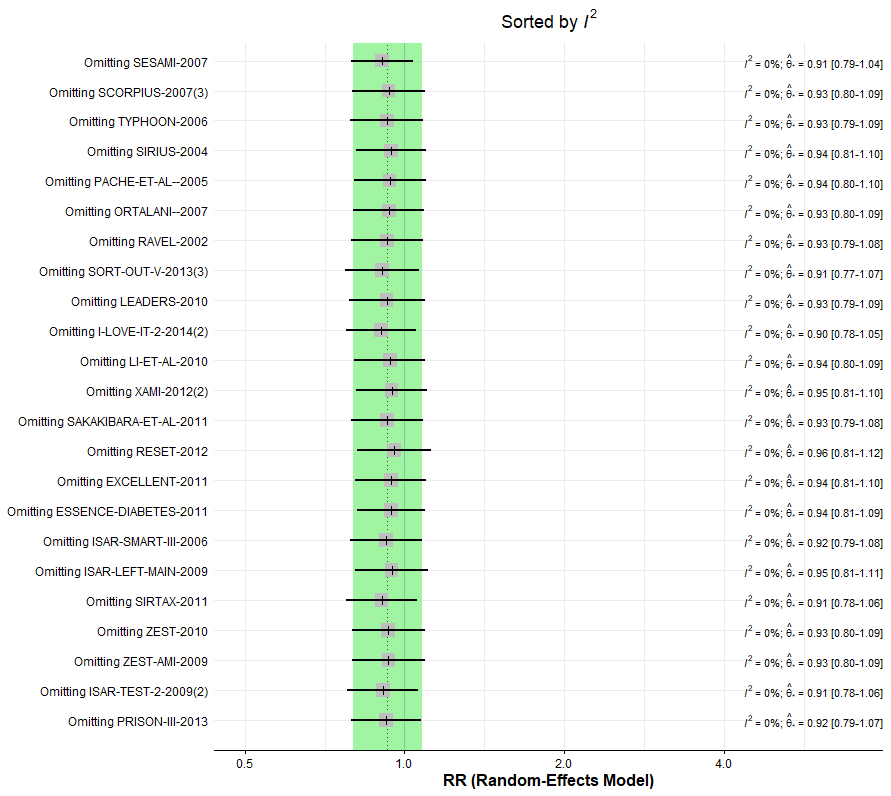
**

**Supplemental Figure 16: Funnel plot showing no evidence of publication bias in comparison of MACE and mortality at 1-year.**


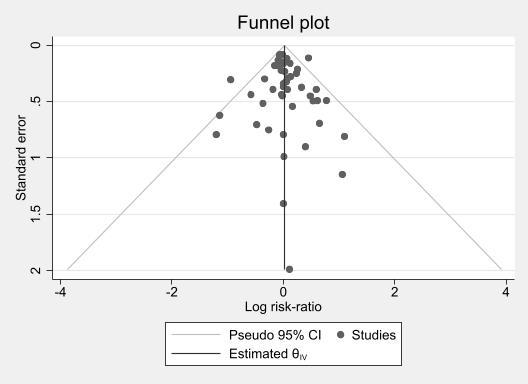

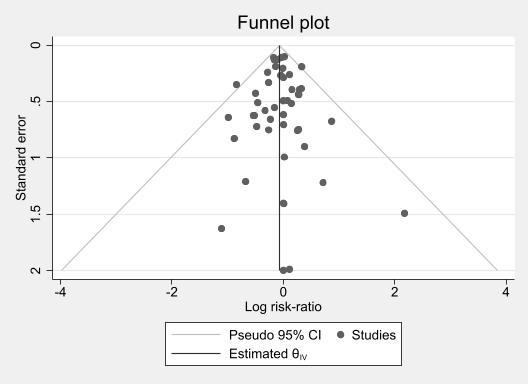


**References:**

1. Serruys PW, Ormiston JA, Sianos G, et al. Actinomycin-eluting stent for coronary revascularization: a randomized feasibility and safety study: the ACTION trial. Journal of the American College of Cardiology. 2004 Oct 6;44(7):1363-7.
2. Velders MA, Hofma SH, Brouwer J, et al. Two-year results of an open-label randomized comparison of everolimus-eluting stents and sirolimus-eluting stents. PLoS One. 2013 Jun 3;8(6):e64424.
3. Kaiser C, Brunner-La Rocca HP, Buser PT, et al. Incremental cost-effectiveness of drug-eluting stents compared with a third-generation bare-metal stent in a real-world setting: randomised Basel Stent Kosten Effektivitäts Trial (BASKET). The Lancet. 2005 Sep 10;366(9489):921-9.
4. Abe D, Sato A, Hoshi T,et al. Drug-eluting versus bare-metal stents in large coronary arteries of patients with ST-segment elevation myocardial infarction: findings from the ICAS registry. Journal of cardiology. 2014 Nov 1;64(5):377-83.
5. Kaiser C, Galatius S, Jeger R, et al. Long-term efficacy and safety of biodegradable-polymer biolimus-eluting stents: main results of the Basel Stent Kosten-Effektivitäts Trial–PROspective Validation Examination II (BASKET-PROVE II), a randomized, controlled noninferiority 2-year outcome trial. Circulation. 2015 Jan 6;131(1):74-81.
6. Jeger R, Pfisterer M, Pfister O, et al. First-generation paclitaxel-vs. second-generation zotarolimus-eluting stents in small coronary arteries: the BASKET-SMALL Pilot Study. Postępy w Kardiologii Interwencyjnej= Advances in Interventional Cardiology. 2016;12(4):314.
7. Yoon CH, Choi YJ, Park JJ, et al. BioMatrix versus Orsiro biodegradable polymer stents in all-comer patients with coronary artery disease: The multicentre, randomized BIODEGRADE trial. EuroIntervention. 2021 Apr;16(17):1404-12.
8. Windecker S, Haude M, Neumann FJ, et al. Comparison of a novel biodegradable polymer sirolimus-eluting stent with a durable polymer everolimus-eluting stent: results of the randomized BIOFLOW-II trial. Circulation: Cardiovascular Interventions. 2015 Feb;8(2):e001441.
9. Saito S, Toelg R, Witzenbichler B, et al. BIOFLOW-IV, a randomised, intercontinental, multicentre study to assess the safety and effectiveness of the Orsiro sirolimus-eluting stent in the treatment of subjects with de novo coronary artery lesions: primary outcome target vessel failure at 12 months. EuroIntervention: journal of EuroPCR in collaboration with the Working Group on Interventional Cardiology of the European Society of Cardiology. 2019 Dec 6;15(11):e1006-13.
10. Kandzari DE, Mauri L, Koolen JJ, et al. Ultrathin, bioresorbable polymer sirolimus-eluting stents versus thin, durable polymer everolimus-eluting stents in patients undergoing coronary revascularisation (BIOFLOW V): a randomised trial. The Lancet. 2017 Oct 21;390(10105):1843-52.
11. Li C, Yang Y, Han Y, et al. Comparison of the ultrathin strut, biodegradable polymer sirolimus-eluting stent with a durable polymer everolimus-eluting stent in a Chinese population: the randomized BIOFLOW VI trial. Clinical therapeutics. 2020 Apr 1;42(4):649-60.
12. Costa RA, Abizaid A, Mehran R, et al. Polymer-free biolimus A9-coated stents in the treatment of de novo coronary lesions: 4-and 12-month angiographic follow-up and final 5-year clinical outcomes of the prospective, multicenter BioFreedom FIM clinical trial. JACC: Cardiovascular Interventions. 2016 Jan 11;9(1):51-64.
13. Iglesias JF, Muller O, Heg D, et al. Biodegradable polymer sirolimus-eluting stents versus durable polymer everolimus-eluting stents in patients with ST-segment elevation myocardial infarction (BIOSTEMI): a single-blind, prospective, randomised superiority trial. The Lancet. 2019 Oct 5;394(10205):1243-53.
14. Kandzari DE, Smits PC, Love MP, et al. Randomized comparison of ridaforolimus-and zotarolimus-eluting coronary stents in patients with coronary artery disease: primary results from the BIONICS Trial (BioNIR Ridaforolimus-Eluting Coronary Stent System in Coronary Stenosis). Circulation. 2017 Oct 3;136(14):1304-14.
15. von Birgelen C, Zocca P, Buiten RA, et al. Thin composite wire strut, durable polymer-coated (Resolute Onyx) versus ultrathin cobalt–chromium strut, bioresorbable polymer-coated (Orsiro) drug-eluting stents in allcomers with coronary artery disease (BIONYX): an international, single-blind, randomised non-inferiority trial. The Lancet. 2018 Oct 6;392(10154):1235-45.
16. von Birgelen C, Kok MM, van der Heijden LC, et al. Very thin strut biodegradable polymer everolimus-eluting and sirolimus-eluting stents versus durable polymer zotarolimus-eluting stents in allcomers with coronary artery disease (BIO-RESORT): a three-arm, randomised, non-inferiority trial. The Lancet. 2016 Nov 26;388(10060):2607-17.
17. Pilgrim T, Heg D, Roffi M, et al. Ultrathin strut biodegradable polymer sirolimus-eluting stent versus durable polymer everolimus-eluting stent for percutaneous coronary revascularisation (BIOSCIENCE): a randomised, single-blind, non-inferiority trial. The Lancet. 2014 Dec 13;384(9960):2111-22.
18. Park HJ, Kim HY, Lee JM, et al. Randomized Comparison of the Efficacy and Safety of Zotarolimus-Eluting Stents vs. Sirolimus-Eluting Stents for Percutaneous Coronary Intervention in Chronic Total Occlusion–CAtholic Total Occlusion Study (CATOS) Trial–. Circulation Journal. 2012;76(4):868-75.
19. Červinka P, Costa MA, Angiolillo DJ, et al. Head‐to‐head comparison between sirolimus‐eluting and paclitaxel‐eluting stents in patients with complex coronary artery disease: An intravascular ultrasound study. Catheterization and cardiovascular interventions. 2006 Jun;67(6):846-51.
20. Saito S, Valdes-Chavarri M, Richardt G, et al. A randomized, prospective, intercontinental evaluation of a bioresorbable polymer sirolimus-eluting coronary stent system: the CENTURY II (Clinical Evaluation of New Terumo Drug-Eluting Coronary Stent System in the Treatment of Patients with Coronary Artery Disease) trial. European heart journal. 2014 Aug 7;35(30):2021-31.
21. Youn YJ, Lee JW, Ahn SG, et al. Randomized comparison of everolimus-and zotarolimus-eluting coronary stents with biolimus-eluting stents in all-comer patients. Circulation: Cardiovascular Interventions. 2020 Mar;13(3):e008525.
22. Reifart N, Hauptmann KE, Rabe A, et al. Short and long term comparison (24 months) of an alternative sirolimus-coated stent with bioabsorbable polymer and a bare metal stent of similar design in chronic coronary occlusions: the CORACTO trial. EuroIntervention. 2010 Aug 1;6(3):356-60.
23. Räber L, Kelbaek H, Ostojic M, et al. Effect of biolimus-eluting stents with biodegradable polymer vs bare-metal stents on cardiovascular events among patients with acute myocardial infarction: the COMFORTABLE AMI randomized trial. Jama. 2012 Aug 22;308(8):777-87.
24. Kedhi E, Joesoef KS, McFadden E, et al. Second-generation everolimus-eluting and paclitaxel-eluting stents in real-life practice (COMPARE): a randomised trial. The Lancet. 2010 Jan 16;375(9710):201-9.
25. Smits PC, Hofma S, Togni M, et al. Abluminal biodegradable polymer biolimus-eluting stent versus durable polymer everolimus-eluting stent (COMPARE II): a randomised, controlled, non-inferiority trial. The Lancet. 2013 Feb 23;381(9867):651-60.
26. Krucoff MW, Kereiakes DJ, Petersen JL, et al. A novel bioresorbable polymer paclitaxel-eluting stent for the treatment of single and multivessel coronary disease: primary results of the COSTAR (Cobalt Chromium Stent With Antiproliferative for Restenosis) II study. Journal of the American College of Cardiology. 2008 Apr 22;51(16):1543-52.
27. Wang L, Zhang D, Yang X, et al. Comparison of Safety and Efficacy of CYPHER (R) Stent and ENDEAVOR (R) Stent in Patients with Acute ST Elevation Myocardial Infarction (STEMI) undergoing emergency PCI and Analysis of Current Status of Emergency PCI Green Channel in China. InJournal of the American College of Cardiology 2011 Nov 8 (Vol. 58, No. 20, pp. B216-B216). 360 PARK AVE SOUTH, NEW YORK, NY 10010-1710 USA: ELSEVIER SCIENCE INC.
28. Carrié D, Berland J, Verheye S,et al. A multicenter randomized trial comparing amphilimus-with paclitaxel-eluting stents in de novo native coronary artery lesions. Journal of the American College of Cardiology. 2012 Apr 10;59(15):1371-6.
29. Schampaert E, Cohen EA, Schlüter M, et al. The Canadian study of the sirolimus-eluting stent in the treatment of patients with long de novo lesions in small native coronary arteries (C-SIRIUS). Journal of the American College of Cardiology. 2004 Mar 17;43(6):1110-5.
30. Qun DA, Li YJ, Lu GA, et al. Six-month angiographic and one-year clinical outcomes of polymer free paclitaxel-eluting stent in patients with ST-segment elevation myocardial infarction: a comparison with permanent polymer sirolimus-eluting stent. Chinese medical journal. 2012 Oct 1;125(19):3393-7.
31. Wijnbergen I, Helmes H, Tijssen J, et al. Comparison of drug-eluting and bare-metal stents for primary percutaneous coronary intervention with or without abciximab in ST-segment elevation myocardial infarction: DEBATER: the Eindhoven reperfusion study. JACC: Cardiovascular Interventions. 2012 Mar;5(3):313-22.
32. Chae IH, Yoon CH, Park JJ, et al. Comparison of drug-eluting balloon followed by bare metal stent with drug-eluting stent for treatment of de novo lesions: randomized, controlled, single-center clinical trial. Journal of Korean medical science. 2017 Jun;32(6):933.
33. Chan C, Zambahari R, Kaul U, et al. A randomized comparison of sirolimus‐eluting versus bare metal stents in the treatment of diabetic patients with native coronary artery lesions: The DECODE study. Catheterization and Cardiovascular Interventions. 2008 Nov 1;72(5):591-600.
34. Kelbæk H, Thuesen L, Helqvist S, et al. Drug-Eluting Versus Bare Metal Stents in Patients With ST-Segment–Elevation Myocardial Infarction: Eight-Month Follow-Up in the Drug Elution and Distal Protection in Acute Myocardial Infarction (DEDICATION) Trial. Circulation. 2008 Sep 9;118(11):1155-62.
35. Lee SW, Park SW, Kim YH, et al. A randomized comparison of sirolimus-versus paclitaxel-eluting stent implantation in patients with diabetes mellitus. Journal of the American College of Cardiology. 2008 Aug 26;52(9):727-33.
36. Maresta A, Varani E, Balducelli M, et al. Comparison of effectiveness and safety of sirolimus-eluting stents versus bare-metal stents in patients with diabetes mellitus (from the Italian Multicenter Randomized DESSERT Study). The American journal of cardiology. 2008 Jun 1;101(11):1560-6.
37. Wijns W, Vrolix M, Verheye S, et al. Randomised study of a bioabsorbable polymer-coated sirolimus-eluting stent: results of the DESSOLVE II trial. EuroIntervention: journal of EuroPCR in collaboration with the Working Group on Interventional Cardiology of the European Society of Cardiology. 2015 Apr 1;10(12):1383-90.
38. de Winter RJ, Katagiri Y, Asano T, et al. A sirolimus-eluting bioabsorbable polymer-coated stent (MiStent) versus an everolimus-eluting durable polymer stent (Xience) after percutaneous coronary intervention (DESSOLVE III): a randomised, single-blind, multicentre, non-inferiority, phase 3 trial. The Lancet. 2018 Feb 3;391(10119):431-40.
39. Maeng M, Jensen LO, Galloe AM, et al. Comparison of the sirolimus-eluting versus paclitaxel-eluting coronary stent in patients with diabetes mellitus: the diabetes and drug-eluting stent (DiabeDES) randomized angiography trial. The American journal of cardiology. 2009 Feb 1;103(3):345-9.
40. Sabaté M, Jiménez-Quevedo P, Angiolillo DJ, et al. Randomized comparison of sirolimus-eluting stent versus standard stent for percutaneous coronary revascularization in diabetic patients: the diabetes and sirolimus-eluting stent (DIABETES) trial. Circulation. 2005 Oct 4;112(14):2175-83.
41. de la Llera LS, Ballesteros S, Nevado J, et al. Sirolimus-eluting stents compared with standard stents in the treatment of patients with primary angioplasty. American heart journal. 2007 Jul 1;154(1):164-e1.
42. Verheye S, Khattab AA, Carrie D, et al. Direct implantation of rapamycin-eluting stents with bioresorbable drug carrier technology utilising the Svelte coronary stent-on-a-wire: the DIRECT II study. EuroIntervention. 2016 Aug 5;12(5):e615-22.
43. Fajadet J, Wijns W, Laarman GJ, et al. Randomized, double-blind, multicenter study of the Endeavor zotarolimus-eluting phosphorylcholine-encapsulated stent for treatment of native coronary artery lesions: clinical and angiographic results of the ENDEAVOR II trial. Circulation. 2006 Aug 22;114(8):798-806.
44. Kandzari DE, Leon MB, Popma JJ, et al. Comparison of zotarolimus-eluting and sirolimus-eluting stents in patients with native coronary artery disease: a randomized controlled trial. Journal of the American College of Cardiology. 2006 Dec 19;48(12):2440-7.
45. Leon MB, Mauri L, Popma JJ, et al. A randomized comparison of the ENDEAVOR zotarolimus-eluting stent versus the TAXUS paclitaxel-eluting stent in de novo native coronary lesions: 12-month outcomes from the ENDEAVOR IV trial. Journal of the American College of Cardiology. 2010 Feb 9;55(6):543-54.
46. Erglis A, Narbute I, Kumsars I, et al. A randomized comparison of paclitaxel-eluting stents versus bare-metal stents for treatment of unprotected left main coronary artery stenosis. Journal of the American College of Cardiology. 2007 Aug 7;50(6):491-7.
47. Kim WJ, Lee SW, Park SW, et al. Randomized Comparison of Everolimus-Eluting Stent Versus Sirolimus-Eluting Stent Implantation for De Novo Coronary Artery Disease in Patients With Diabetes Mellitus (ESSENCE-DIABETES) Results From the ESSENCE-DIABETES Trial. Circulation. 2011 Aug 23;124(8):886-92.
48. Rodriguez AE, Vigo CF, Delacasa A, et al. Efficacy and safety of a double‐coated paclitaxel‐eluting coronary stent: The EUCATAX trial. Catheterization and Cardiovascular Interventions. 2011 Feb 15;77(3):335-42.
49. Puricel S, Arroyo D, Corpataux N, et al. Comparison of everolimus-and biolimus-eluting coronary stents with everolimus-eluting bioresorbable vascular scaffolds. Journal of the American College of Cardiology. 2015 Mar 3;65(8):791-801.
50. Meredith IT, Verheye S, Dubois CL, et al.Primary endpoint results of the EVOLVE trial: a randomized evaluation of a novel bioabsorbable polymer-coated, everolimus-eluting coronary stent. Journal of the American College of Cardiology. 2012 Apr 10;59(15):1362-70.
51. Kereiakes DJ, Meredith IT, Windecker S, et al. Efficacy and safety of a novel bioabsorbable polymer-coated, everolimus-eluting coronary stent: the EVOLVE II Randomized Trial. Circulation: Cardiovascular Interventions. 2015 Apr;8(4):e002372.
52. Brugaletta S, Gomez-Lara J, Ortega-Paz L, et al. 10-year follow-up of patients with everolimus-eluting versus bare-metal stents after ST-segment elevation myocardial infarction. Journal of the American College of Cardiology. 2021 Mar 9;77(9):1165-78.
53. Serruys PW, Garg S, Abizaid A, et al. A randomised comparison of novolimus-eluting and zotarolimus-eluting coronary stents: 9-month follow-up results of the EXCELLA II study. EuroIntervention. 2010 Jun 1;6(2):195-205.
54. Park KW, Chae IH, Lim DS, et al. Everolimus-eluting versus sirolimus-eluting stents in patients undergoing percutaneous coronary intervention: the EXCELLENT (Efficacy of Xience/Promus Versus Cypher to Reduce Late Loss After Stenting) randomized trial. Journal of the American College of Cardiology. 2011 Oct 25;58(18):1844-54.
55. Schofer J, Schlüter M, Gershlick AH, et al. Sirolimus-eluting stents for treatment of patients with long atherosclerotic lesions in small coronary arteries: double-blind, randomised controlled trial (E-SIRIUS). The Lancet. 2003 Oct 4;362(9390):1093-9.
56. Hai GA, Yan HB, Zhu XL, et al. Firebird sirolimus eluting stent versus bare mental stent in patients with ST-segment elevation myocardial infarction. Chinese medical journal. 2007 May 2;120(10):863-7.
57. Fukumoto A, Otsuji S, Takiuchi S, et al. Comparison of real-world clinical outcomes between Cypher-and Taxus-eluting stents: the GARA–GARA study. Cardiovascular intervention and therapeutics. 2011 Sep 1;26(3):202.
58. Verheye S, Agostoni P, Dawkins KD, et al. The GENESIS (randomized, multicenter study of the pimecrolimus-eluting and pimecrolimus/paclitaxel-eluting coronary stent system in patients with de novo lesions of the native coronary arteries) trial. JACC: Cardiovascular Interventions. 2009 Mar;2(3):205-14.
59. Sánchez PL, Gimeno F, Ancillo P, et al. Role of the paclitaxel-eluting stent and tirofiban in patients with ST-elevation myocardial infarction undergoing postfibrinolysis angioplasty: the GRACIA-3 randomized clinical trial. Circulation: Cardiovascular Interventions. 2010 Aug;3(4):297-307.
60. Rubartelli P, Petronio AS, Guiducci V,et al. Comparison of sirolimus-eluting and bare metal stent for treatment of patients with total coronary occlusions: results of the GISSOC II-GISE multicentre randomized trial. European heart journal. 2010 Aug 1;31(16):2014-20.
61. Saito S, Krucoff MW, Nakamura S, et al. Japan-United States of America Harmonized Assessment by Randomized Multicentre Study of OrbusNEich’s Combo StEnt (Japan-USA HARMONEE) study: primary results of the pivotal registration study of combined endothelial progenitor cell capture and drug-eluting stent in patients with ischaemic coronary disease and non-ST-elevation acute coronary syndrome. European heart journal. 2018 Jul 7;39(26):2460-8.
62. Herdeg C, Göhring-Frischholz K, Haase KK, et al. Catheter-based delivery of fluid paclitaxel for prevention of restenosis in native coronary artery lesions after stent implantation. Circulation: Cardiovascular Interventions. 2009 Aug;2(4):294-301.
63. Hong SJ, Kim MH, Cha KS, et al. Comparison of three‐year clinical outcomes between sirolimus‐versus paclitaxel‐eluting stents in diabetic patients: Prospective randomized multicenter trial. Catheterization and Cardiovascular Interventions. 2010 Dec 1;76(7):924-33.
64. Stone GW, Lansky AJ, Pocock SJ, et al. Paclitaxel-eluting stents versus bare-metal stents in acute myocardial infarction. New England Journal of Medicine. 2009 May 7;360(19):1946-59.
65. Park KW, Kang SH, Kang HJ, et al. A Randomized Comparison of Platinum Chromium-Based Everolimus-Eluting Stents Versus Cobalt Chromium-Based Zotarolimus-Eluting Stents in All-Comers Receiving Percutaneous Coronary Intervention: HOST–ASSURE (Harmonizing Optimal Strategy for Treatment of Coronary Artery Stenosis–Safety & Effectiveness of Drug-Eluting Stents & Anti-platelet Regimen), a Randomized, Controlled, Noninferiority Trial. Journal of the American College of Cardiology. 2014 Jul 1;63(25 Part A):2805-16.
66. Oliveira MD, Ribeiro EE, Campos CM, et al. Four-year clinical follow-up of the first-in-man randomized comparison of a novel sirolimus eluting stent with abluminal biodegradable polymer and ultra-thin strut cobalt-chromium alloy: the INSPIRON-I trial. Cardiovascular diagnosis and therapy. 2015 Aug;5(4):264.
67. Dibra A, Kastrati A, Mehilli J, et al. Paclitaxel-eluting or sirolimus-eluting stents to prevent restenosis in diabetic patients. New England Journal of Medicine. 2005 Aug 18;353(7):663-70.
68. Mehilli J, Kastrati A, Byrne RA,et al. Paclitaxel-versus sirolimus-eluting stents for unprotected left main coronary artery disease. Journal of the American College of Cardiology. 2009 May 12;53(19):1760-8.
69. Mehilli J, Richardt G, Valgimigli M, et al. Zotarolimus-versus everolimus-eluting stents for unprotected left main coronary artery disease. Journal of the American College of Cardiology. 2013 Dec 3;62(22):2075-82.
70. Mehilli J, Dibra A, Kastrati A, et al. Randomized trial of paclitaxel-and sirolimus-eluting stents in small coronary vessels. European heart journal. 2006 Feb 1;27(3):260-6.
71. Mehilli J, Kastrati A, Wessely R, et al. Randomized trial of a nonpolymer-based rapamycin-eluting stent versus a polymer-based paclitaxel-eluting stent for the reduction of late lumen loss. Circulation. 2006 Jan 17;113(2):273-9.
72. Byrne RA, Mehilli J, Iijima R, et al. A polymer-free dual drug-eluting stent in patients with coronary artery disease: a randomized trial vs. polymer-based drug-eluting stents. European heart journal. 2009 Apr 1;30(8):923-31.
73. Byrne RA, Kastrati A, Kufner S, et al. Randomized, non-inferiority trial of three limus agent-eluting stents with different polymer coatings: the Intracoronary Stenting and Angiographic Results: Test Efficacy of 3 Limus-Eluting Stents (ISAR-TEST-4) Trial. European heart journal. 2009 Oct 1;30(20):2441-9.
74. Massberg S, Byrne RA, Kastrati A, et al. Polymer-free sirolimus-and probucol-eluting versus new generation zotarolimus-eluting stents in coronary artery disease: the Intracoronary Stenting and Angiographic Results: Test Efficacy of Sirolimus-and Probucol-Eluting versus Zotarolimus-eluting Stents (ISAR-TEST 5) trial. Circulation. 2011 Aug 2;124(5):624-32.
75. Han Y, Xu B, Jing Q, et al. A randomized comparison of novel biodegradable polymer-and durable polymer–coated cobalt-chromium sirolimus-eluting stents. JACC: Cardiovascular Interventions. 2014 Dec;7(12):1352-60.
76. Nakamura M, Muramatsu T, Yokoi H, et al. Outcomes of the largest multi-center trial stratified by the presence of diabetes mellitus comparing sirolimus-eluting stents (SES) and paclitaxel-eluting stents (PES) in patients with coronary artery disease. The Japan drug-eluting stents evaluation: a randomized trial (J-DESsERT). Cardiovascular intervention and therapeutics. 2015 Apr 1;30(2):103-14.
77. Juwana YB, Suryapranata H, Ottervanger JP, et al. Comparison of rapamycin-and paclitaxel-eluting stents in patients undergoing primary percutaneous coronary intervention for ST-elevation myocardial infarction. The American journal of cardiology. 2009 Jul 15;104(2):205-9.
78. Kamoi D, Ishii H, Takahashi H, et al. Sirolimus-vs. paclitaxel-eluting stent to coronary intervention in dialysis patients. International journal of cardiology. 2013 May 25;165(3):533-6.
79. Kim MH, Hong SJ, Cha KS, et al. Effect of paclitaxel‐eluting versus sirolimus‐eluting stents on coronary restenosis in Korean diabetic patients. Journal of interventional cardiology. 2008 Jun;21(3):225-31.
80. Kang WC, Ahn T, Lee K, et al. Comparison of zotarolimus-eluting stents versus sirolimus-eluting stents versus paclitaxel-eluting stents for primary percutaneous coronary intervention in patients with ST-elevation myocardial infarction: results from the Korean Multicentre Endeavor (KOMER) acute myocardial infarction (AMI) trial. EuroIntervention: journal of EuroPCR in collaboration with the Working Group on Interventional Cardiology of the European Society of Cardiology. 2011 Dec 1;7(8):936-43.
81. Laarman GJ, Suttorp MJ, Dirksen MT, et al. Paclitaxel-eluting versus uncoated stents in primary percutaneous coronary intervention. New England Journal of Medicine. 2006 Sep 14;355(11):1105-13.
82. Lansky AJ, Roubin GS, O’Shaughnessy CD, et al. Randomized comparison of GR-II stent and Palmaz-Schatz stent for elective treatment of coronary stenoses. Circulation. 2000 Sep 19;102(12):1364-8.
83. Windecker S, Serruys PW, Wandel S, et al. Biolimus-eluting stent with biodegradable polymer versus sirolimus-eluting stent with durable polymer for coronary revascularisation (LEADERS): a randomised non-inferiority trial. The Lancet. 2008 Sep 27;372(9644):1163-73.
84. Urban P, Meredith IT, Abizaid A, et al. Polymer-free drug-coated coronary stents in patients at high bleeding risk. New England Journal of Medicine. 2015 Nov 19;373(21):2038-47.
85. Li Q, Tong Z, Wang L, et al. Efficacy and safety of a biodegradable polymer sirolimus-eluting stent in primary percutaneous coronary intervention: a randomized controlled trial. Archives of medical science: AMS. 2013 Dec 30;9(6):1040.
86. Desch S, Schloma D, Möbius-Winkler S, et al. Randomized comparison of a polymer-free sirolimus-eluting stent versus a polymer-based paclitaxel-eluting stent in patients with diabetes mellitus: the LIPSIA Yukon trial. JACC: Cardiovascular Interventions. 2011 Apr;4(4):452-9.
87. Shiratori Y, Cola C, Brugaletta S, et al. Randomized comparison between polymer-free versus polymer-based paclitaxel-eluting stent: two-year final clinical results. Circulation: Cardiovascular Interventions. 2014 Jun;7(3):312-21.
88. Kim YH, Park SW, Lee SW, et al. Sirolimus-eluting stent versus paclitaxel-eluting stent for patients with long coronary artery disease. Circulation. 2006 Nov 14;114(20):2148-53.
89. Park DW, Kim YH, Song HG, et al. Comparison of everolimus-and sirolimus-eluting stents in patients with long coronary artery lesions: a randomized LONG-DES-III (Percutaneous Treatment of LONG Native Coronary Lesions With Drug-Eluting Stent-III) Trial. JACC: Cardiovascular interventions. 2011 Oct;4(10):1096-103.
90. Ahn JM, Park DW, Kim YH, et al. Comparison of resolute zotarolimus-eluting stents and sirolimus-eluting stents in patients with de novo long coronary artery lesions: a randomized LONG-DES IV trial. Circulation: Cardiovascular Interventions. 2012 Oct;5(5):633-40.
91. Lee JY, Park DW, Kim YH, et al. Comparison of Biolimus A9–Eluting (Nobori) and Everolimus-Eluting (Promus Element) Stents in Patients With De Novo Native Long Coronary Artery Lesions: A Randomized Long Drug-Eluting Stent V Trial. Circulation: Cardiovascular Interventions. 2014 Jun;7(3):322-9.
92. Stone GW, Abizaid A, Silber S, et al. Prospective, Randomized, Multicenter Evaluation of a Polyethylene Terephthalate Micronet Mesh–Covered Stent (MGuard) in ST-Segment Elevation Myocardial Infarction: The MASTER Trial. Journal of the American College of Cardiology. 2012 Nov 6;60(19):1975-84.
93. Valdes-Chavarri M, Kedev S, Neskovic AN, et al. Randomised evaluation of a novel biodegradable polymer-based sirolimus-eluting stent in ST-segment elevation myocardial infarction: the MASTER study. EuroIntervention: journal of EuroPCR in collaboration with the Working Group on Interventional Cardiology of the European Society of Cardiology. 2019 Apr 5;14(18):e1836-42.
94. Abizaid A, Kedev S, Kedhi E, et al. Randomised comparison of a biodegradable polymer ultra-thin sirolimus-eluting stent versus a durable polymer everolimus-eluting stent in patients with de novo native coronary artery lesions: the meriT-V trial. EuroIntervention: journal of EuroPCR in collaboration with the Working Group on Interventional Cardiology of the European Society of Cardiology. 2018 Dec 7;14(11):e1207-14.
95. van der Hoeven BL, Liem SS, Jukema JW, et al. Sirolimus-eluting stents versus bare-metal stents in patients with ST-segment elevation Myocardial infarction: 9-month angiographic and intravascular ultrasound results and 12-month clinical outcome: results from the MISSION! Intervention study. Journal of the American College of Cardiology. 2008 Feb 12;51(6):618-26.
96. Valgimigli M, Campo G, Percoco G, et al. Comparison of angioplasty with infusion of tirofiban or abciximab and with implantation of sirolimus-eluting or uncoated stents for acute myocardial infarction: the MULTISTRATEGY randomized trial. Jama. 2008 Apr 16;299(15):1788-99.
97. Briguori C, Airoldi F, Visconti G, et al. Novel approaches for preventing or limiting events in diabetic patients (Naples-diabetes) trial: a randomized comparison of 3 drug-eluting stents in diabetic patients. Circulation: Cardiovascular Interventions. 2011 Apr;4(2):121-9.
98. Ormiston JA, Abizaid A, Spertus J, et al. Six-month results of the NEVO Res-Elution I (NEVO RES-I) trial: a randomized, multicenter comparison of the NEVO sirolimus-eluting coronary stent with the TAXUS Liberte paclitaxel-eluting stent in de novo native coronary artery lesions. Circulation: Cardiovascular Interventions. 2010 Dec;3(6):556-64.
99. Natsuaki M, Kozuma K, Morimoto T, et al. Biodegradable polymer biolimus-eluting stent versus durable polymer everolimus-eluting stent: a randomized, controlled, noninferiority trial. Journal of the American College of Cardiology. 2013 Jul 16;62(3):181-90.
100. Chevalier B, Wijns W, Silber S, et al. Five-year clinical outcome of the Nobori drug-eluting coronary stent system in the treatment of patients with coronary artery disease: final results of the NOBORI 1 trial. EuroIntervention. 2015 Sep 1;11(5):549-.
101. Kadota K, Muramatsu T, Iwabuchi M, et al. Randomized comparison of the Nobori biolimus A9‐eluting stent with the sirolimus‐eluting stent in patients with stenosis in native coronary arteries. Catheterization and Cardiovascular Interventions. 2012 Nov 1;80(5):789-96.
102. Bønaa KH, Mannsverk J, Wiseth R,et al. Drug-eluting or bare-metal stents for coronary artery disease. New England Journal of Medicine. 2016 Sep 29;375(13):1242-52.
103. Hamshere S, Byrne A, Choudhury T, et al. Randomised trial of the comparison of drug-eluting stents in patients with diabetes: OCT DES trial. Open heart. 2018 Apr 1;5(1):e000705.
104. Guagliumi G, Sirbu V, Musumeci G,et al. Strut coverage and vessel wall response to a new-generation paclitaxel-eluting stent with an ultrathin biodegradable abluminal polymer: Optical Coherence Tomography Drug-Eluting Stent Investigation (OCTDESI). Circulation: Cardiovascular Interventions. 2010 Aug;3(4):367-75.
105. Windecker S, Latib A, Kedhi E, et al. Polymer-based or polymer-free stents in patients at high bleeding risk. New England Journal of Medicine. 2020 Mar 26;382(13):1208-18.
106. Kang SH, Chung WY, Lee JM,et al. Angiographic outcomes of Orsiro biodegradable polymer sirolimus-eluting stents and Resolute Integrity durable polymer zotarolimus-eluting stents: results of the ORIENT trial. EuroIntervention. 2017 Jan 1;12(13):1623-31.
107. Ortolani P, Marzocchi A, Marrozzini C, et al. Randomized comparative trial of a thin‐strut bare metal cobalt‐chromium stent versus a sirolimus‐eluting stent for coronary revascularization. Catheterization and Cardiovascular Interventions. 2007 May 1;69(6):790-8.
108. Pache J, Dibra A, Mehilli J, et al. Drug-eluting stents compared with thin-strut bare stents for the reduction of restenosis: a prospective, randomized trial. European heart journal. 2005 Jul 1;26(13):1262-8.
109. Lemos PA, Moulin B, Perin MA, et al. Late clinical outcomes after implantation of drug-eluting stents coated with biodegradable polymers: 3-year follow-up of the PAINT randomised trial. EuroIntervention. 2012 May 1;8(1):117-9.
110. Xu B, Gao R, Yang Y, et al. Biodegradable polymer-based sirolimus-eluting stents with differing elution and absorption kinetics: the PANDA III trial. Journal of the American College of Cardiology. 2016 May 17;67(19):2249-58.
111. Di Lorenzo E, De Luca G, Sauro R, et al. The PASEO (paclitaxel or sirolimus-eluting stent versus bare metal stent in primary angioplasty) randomized trial. JACC: Cardiovascular Interventions. 2009 Jun;2(6):515-23.
112. Laarman GJ, Suttorp MJ, Dirksen MT, et al. Paclitaxel-eluting versus uncoated stents in primary percutaneous coronary intervention. New England Journal of Medicine. 2006 Sep 14;355(11):1105-13.
113. Petronio AS, De Carlo M, Branchitta G, et al. Randomized comparison of sirolimus and paclitaxel drug-eluting stents for long lesions in the left anterior descending artery: an intravascular ultrasound study. Journal of the American College of Cardiology. 2007 Feb 6;49(5):539-46.
114. Lansky AJ, Kereiakes DJ, Baumbach A, et al. Novel supreme drug-eluting stents with early synchronized antiproliferative drug delivery to inhibit smooth muscle cell proliferation after drug-eluting stents implantation in coronary artery disease: results of the PIONEER III randomized clinical trial. Circulation. 2021 Jun 1;143(22):2143-54.
115. Stone GW, Teirstein PS, Meredith IT, et al. A prospective, randomized evaluation of a novel everolimus-eluting coronary stent: the PLATINUM (a Prospective, Randomized, Multicenter Trial to Assess an Everolimus-Eluting Coronary Stent System [PROMUS Element] for the Treatment of Up to Two de Novo Coronary Artery Lesions) trial. Journal of the American College of Cardiology. 2011 Apr 19;57(16):1700-8.
116. Fajadet J, Neumann FJ, Hildick-Smith D, et al. Twelve-month results of a prospective, multicentre trial to assess the everolimus-eluting coronary stent system (PROMUS Element): the PLATINUM PLUS all-comers randomised trial. EuroIntervention. 2017 Jan 20;12(13):1595-604.
117. Pourmoghaddas M, Rohani HR, Sanei H, et al. Undesired Outcomes of the Catania Stent Compared to the Xience Stent in Patients Undergoing Angioplasty: A Double-Blind Randomized Controlled Trial. Advanced biomedical research. 2017;6.
118. Hlinomaz O, Motovska Z, Knot J,et al. Stent Selection for Primary Angioplasty and Outcomes in the Era of Potent Antiplatelets. Data from the Multicenter Randomized Prague-18 Trial. Journal of Clinical Medicine. 2021 Jan;10(21):5103.
119. Suttorp MJ, Laarman GJ, Rahel BM, et al. Primary stenting of totally occluded native coronary arteries II (PRISON II) a randomized comparison of bare metal stent implantation with sirolimus-eluting stent implantation for the treatment of total coronary occlusions. Circulation. 2006 Aug 29;114(9):921-8.
120. Van den Branden BJ, Teeuwen K, Koolen JJ, et al. Primary Stenting of Totally Occluded Native Coronary Arteries III (PRISON III): a randomised comparison of sirolimus-eluting stent implantation with zotarolimus-eluting stent implantation for the treatment of total coronary occlusions. EuroIntervention: journal of EuroPCR in collaboration with the Working Group on Interventional Cardiology of the European Society of Cardiology. 2013 Nov 1;9(7):841-53.
121. Teeuwen K, van der Schaaf RJ, Adriaenssens T, et al. Randomized multicenter trial investigating angiographic outcomes of hybrid sirolimus-eluting stents with biodegradable polymer compared with everolimus-eluting stents with durable polymer in chronic total occlusions: the PRISON IV trial. JACC: Cardiovascular Interventions. 2017 Jan 23;10(2):133-43.
122. Valgimigli M, Tebaldi M, Borghesi M, et al. Two-year outcomes after first-or second-generation drug-eluting or bare-metal stent implantation in all-comer patients undergoing percutaneous coronary intervention: a pre-specified analysis from the PRODIGY study (PROlonging Dual Antiplatelet Treatment After Grading stent-induced Intimal hyperplasia studY). JACC: Cardiovascular interventions. 2014 Jan;7(1):20-8.
123. Kim U, Lee CH, Jo JH, et al. A prospective, randomized comparison of promus everolimus-eluting and TAXUS Liberte paclitaxel-eluting stent systems in patients with coronary artery disease eligible for percutaneous coronary intervention: the PROMISE study. Journal of Korean medical science. 2013 Nov 1;28(11):1609-14.
124. Lee JH, Kim HS, Lee SW, et al. Prospective randomized comparison of sirolimus‐versus paclitaxel‐eluting stents for the treatment of acute ST‐elevation myocardial infarction: pROSIT trial. Catheterization and Cardiovascular Interventions. 2008 Jul 1;72(1):25-32.
125. Camenzind E, Wijns W, Mauri L, et al. Stent thrombosis and major clinical events at 3 years after zotarolimus-eluting or sirolimus-eluting coronary stent implantation: a randomised, multicentre, open-label, controlled trial. The Lancet. 2012 Oct 20;380(9851):1396-405.
126. Morice MC, Serruys PW, Sousa JE, et al. A randomized comparison of a sirolimus-eluting stent with a standard stent for coronary revascularization. New England Journal of Medicine. 2002 Jun 6;346(23):1773-80.
127. Morice MC, Colombo A, Meier B, et al. Sirolimus-vs paclitaxel-eluting stents in de novo coronary artery lesions: the REALITY trial: a randomized controlled trial. Jama. 2006 Feb 22;295(8):895-904.
128. Tao L, Li Z, Yin Z, et al. Nine‐month angiographic and 2‐year clinical outcomes of the RECOVERY trial: A randomized study of the biodegradable polymer sirolimus‐eluting COMBO dual‐therapy stent versus a polymer‐free sirolimus‐eluting stent in Chinese patients. Catheterization and Cardiovascular Interventions. 2021 May 1;97:966-75.
129. Rozemeijer R, Stein M, Voskuil M, et al. Randomized all-comers evaluation of a permanent polymer zotarolimus-eluting stent versus a polymer-free amphilimus-eluting stent: multicenter, noninferiority trial (ReCre8). Circulation. 2019 Jan 2;139(1):67-77.
130. Haude M, Lee SW, Worthley SG,et al. The REMEDEE trial: a randomized comparison of a combination sirolimus-eluting endothelial progenitor cell capture stent with a paclitaxel-eluting stent. JACC: Cardiovascular interventions. 2013 Apr;6(4):334-43.
131. Romaguera R, Gómez-Hospital JA, Gomez-Lara J, et al. A randomized comparison of reservoir-based polymer-free amphilimus-eluting stents versus everolimus-eluting stents with durable polymer in patients with diabetes mellitus: the RESERVOIR clinical trial. JACC: Cardiovascular Interventions. 2016 Jan 11;9(1):42-50.
132. Kimura T, Morimoto T, Natsuaki M, et al. Comparison of everolimus-eluting and sirolimus-eluting coronary stents: 1-year outcomes from the Randomized Evaluation of Sirolimus-eluting Versus Everolimus-eluting stent Trial (RESET). Circulation. 2012 Sep 4;126(10):1225-36.
133. Serruys PW, Silber S, Garg S, et al. Comparison of zotarolimus-eluting and everolimus-eluting coronary stents. New England Journal of Medicine. 2010 Jul 8;363(2):136-46.
134. Xu B, Yang Y, Yuan Z, et al. Zotarolimus-and paclitaxel-eluting stents in an all-comer population in China: the RESOLUTE China randomized controlled trial. JACC: Cardiovascular Interventions. 2013 Jul;6(7):664-70.
135. Sakakibara T, Ishii H, Toriyama T,et al. Sirolimus-eluting stent vs. everolimus-eluting stent for coronary intervention in patients on chronic hemodialysis. Circulation Journal. 2011:1111221481-.
136. Kelbæk H, Thuesen L, Helqvist S, et al. The stenting coronary arteries in non-stress/benestent disease (SCANDSTENT) trial. Journal of the American College of Cardiology. 2006 Jan 17;47(2):449-55.
137. Baumgart D, Klauss V, Baer F, et al. One-year results of the SCORPIUS study: a German multicenter investigation on the effectiveness of sirolimus-eluting stents in diabetic patients. Journal of the American College of Cardiology. 2007 Oct 23;50(17):1627-34.
138. Burzotta F, Trani C, Todaro D, et al. Prospective randomized comparison of sirolimus-or everolimus-eluting stent to treat bifurcated lesions by provisional approach. JACC: Cardiovascular Interventions. 2011 Mar;4(3):327-35.
139. Chechi T, Vittori G, BIONDI ZOCCAI GG, et al. Single‐center randomized evaluation of paclitaxel‐eluting versus conventional stent in acute myocardial infarction (SELECTION). Journal of interventional cardiology. 2007 Aug;20(4):282-91.
140. Varenne O, Cook S, Sideris G, et al. Drug-eluting stents in elderly patients with coronary artery disease (SENIOR): a randomised single-blind trial. The Lancet. 2018 Jan 6;391(10115):41-50.
141. Separham A, Sohrabi B, Aslanabadi N, et al. The twelve-month outcome of biolimus eluting stent with biodegradable polymer compared with an everolimus eluting stent with durable polymer. Journal of cardiovascular and thoracic research. 2011;3(4):113.
142. Menichelli M, Parma A, Pucci E, et al. Randomized trial of sirolimus-eluting stent versus bare-metal stent in acute myocardial infarction (SESAMI). Journal of the American College of Cardiology. 2007 May 15;49(19):1924-30.
143. Ardissino D, Cavallini C, Bramucci E, et al. Sirolimus-eluting vs uncoated stents for prevention of restenosis in small coronary arteries: a randomized trial. Jama. 2004 Dec 8;292(22):2727-34.
144. Shen L, Yang W, Yin JS,et al. Nine-month angiographic and two-year clinical follow-up of novel biodegradable-polymer arsenic trioxide-eluting stent versus durable-polymer sirolimus-eluting stent for coronary artery disease. Chinese medical journal. 2015 Mar 20;128(6):768.
145. Moses JW, Leon MB, Popma JJ, et al. Sirolimus-eluting stents versus standard stents in patients with stenosis in a native coronary artery. New England Journal of Medicine. 2003 Oct 2;349(14):1315-23.
146. Windecker S, Remondino A, Eberli FR, et al. Sirolimus-eluting and paclitaxel-eluting stents for coronary revascularization. New England Journal of Medicine. 2005 Aug 18;353(7):653-62.
147. Galløe AM, Thuesen L, Kelbæk H, et al. Comparison of paclitaxel-and sirolimus-eluting stents in everyday clinical practice: the SORT OUT II randomized trial. Jama. 2008 Jan 30;299(4):409-16.
148. Rasmussen K, Maeng M, Kaltoft A, et al. Efficacy and safety of zotarolimus-eluting and sirolimus-eluting coronary stents in routine clinical care (SORT OUT III): a randomised controlled superiority trial. The Lancet. 2010 Mar 27;375(9720):1090-9.
149. Okkels Jensen L, Thayssen P, Hansen HS, et al. Randomized comparison of everolimus-eluting and sirolimus-eluting stents in patients treated with percutaneous coronary intervention: the Scandinavian Organization for Randomized Trials with Clinical Outcome IV (SORT OUT IV). Circulation. 2012 Mar 13;125(10):1246-55.
150. Christiansen EH, Jensen LO, Thayssen P, et al. Biolimus-eluting biodegradable polymer-coated stent versus durable polymer-coated sirolimus-eluting stent in unselected patients receiving percutaneous coronary intervention (SORT OUT V): a randomised non-inferiority trial. The Lancet. 2013 Feb 23;381(9867):661-9.
151. Raungaard B, Jensen LO, Tilsted HH, et al. Zotarolimus-eluting durable-polymer-coated stent versus a biolimus-eluting biodegradable-polymer-coated stent in unselected patients undergoing percutaneous coronary intervention (SORT OUT VI): a randomised non-inferiority trial. The Lancet. 2015 Apr 18;385(9977):1527-35.
152. Jensen LO, Thayssen P, Maeng M, et al. Randomized comparison of a biodegradable polymer ultrathin strut sirolimus-eluting stent with a biodegradable polymer biolimus-eluting stent in patients treated with percutaneous coronary intervention: the SORT OUT VII trial. Circulation: Cardiovascular Interventions. 2016 Jul;9(7):e003610.
153. Maeng M, Christiansen EH, Raungaard B, et al. Everolimus-eluting versus biolimus-eluting stents with biodegradable polymers in unselected patients undergoing percutaneous coronary intervention: A randomized noninferiority trial with 1-year follow-up (SORT OUT VIII Trial). JACC: Cardiovascular Interventions. 2019 Apr 8;12(7):624-33.
154. Jensen LO, Maeng M, Raungaard B, et al. Randomized comparison of the polymer-free biolimus-coated biofreedom stent with the ultrathin strut biodegradable polymer sirolimus-eluting orsiro stent in an all-comers population treated with percutaneous coronary intervention: the SORT OUT IX trial. Circulation. 2020 Jun 23;141(25):2052-63.
155. Jakobsen L, Christiansen EH, Freeman P, et al. Randomized clinical comparison of the dual-therapy CD34 antibody-covered sirolimus-eluting Combo stent with the sirolimus-eluting Orsiro stent in patients treated with percutaneous coronary intervention: the SORT OUT X trial. Circulation. 2021 Jun 1;143(22):2155-65.
156. Tsuchida K, Piek JJ, Neumann FJ, et al. One-year results of a durable polymer everolimus-eluting stent in de novo coronary narrowings (The SPIRIT FIRST Trial). EuroIntervention: journal of EuroPCR in collaboration with the Working Group on Interventional Cardiology of the European Society of Cardiology. 2005 Nov 1;1(3):266-72.
157. Claessen BE, Beijk MA, Legrand V, et al. Two-year clinical, angiographic, and intravascular ultrasound follow-up of the XIENCE V everolimus-eluting stent in the treatment of patients with de novo native coronary artery lesions: the SPIRIT II trial. Circulation: Cardiovascular Interventions. 2009 Aug;2(4):339-47.
158. Stone GW, Midei M, Newman W, et al. Comparison of an everolimus-eluting stent and a paclitaxel-eluting stent in patients with coronary artery disease: a randomized trial. Jama. 2008 Apr 23;299(16):1903-13.
159. Stone GW, Rizvi A, Newman W, et al. Everolimus-eluting versus paclitaxel-eluting stents in coronary artery disease. New England Journal of Medicine. 2010 May 6;362(18):1663-74.
160. Grube E, Chevalier B, Guagliumi G, et al. The SPIRIT V diabetic study: a randomized clinical evaluation of the XIENCE V everolimus-eluting stent vs the TAXUS Liberté paclitaxel-eluting stent in diabetic patients with de novo coronary artery lesions. American heart journal. 2012 May 1;163(5):867-75.
161. Valgimigli M, Percoco G, Malagutti P, et al. Tirofiban and sirolimus-eluting stent vs abciximab and bare-metal stent for acute myocardial infarction: a randomized trial. Jama. 2005 May 4;293(17):2109-17.
162. Lansky A, Wijns W, Xu B, et al. Targeted therapy with a localised abluminal groove, low-dose sirolimus-eluting, biodegradable polymer coronary stent (TARGET All Comers): a multicentre, open-label, randomised non-inferiority trial. The Lancet. 2018 Sep 29;392(10153):1117-26.
163. Gao RL, Xu B, Lansky AJ, et al. A randomised comparison of a novel abluminal groove-filled biodegradable polymer sirolimus-eluting stent with a durable polymer everolimus-eluting stent: clinical and angiographic follow-up of the TARGET I trial. EuroIntervention. 2013 May 1;9(1):75-83.
164. Zaman A, de Winter RJ, Kogame N, et al. Safety and efficacy of a sirolimus-eluting coronary stent with ultra-thin strut for treatment of atherosclerotic lesions (TALENT): a prospective multicentre randomised controlled trial. The Lancet. 2019 Mar 9;393(10175):987-97.
165. Goy JJ, Stauffer JC, Siegenthaler M, et al. A prospective randomized comparison between paclitaxel and sirolimus stents in the real world of interventional cardiology: the TAXi trial. Journal of the American College of Cardiology. 2005 Jan 18;45(2):308-11.
166. Grube E, Silber S, Hauptmann KE, et al. TAXUS I: six-and twelve-month results from a randomized, double-blind trial on a slow-release paclitaxel-eluting stent for de novo coronary lesions. Circulation. 2003 Jan 7;107(1):38-42.
167. Colombo A, Drzewiecki J, Banning A, et al. Randomized study to assess the effectiveness of slow-and moderate-release polymer-based paclitaxel-eluting stents for coronary artery lesions. Circulation. 2003 Aug 19;108(7):788-94.
168. Stone GW, Ellis SG, Cox DA, et al. A polymer-based, paclitaxel-eluting stent in patients with coronary artery disease. New England Journal of Medicine. 2004 Jan 15;350(3):221-31.
169. Stone GW, Ellis SG, Cannon L, et al. Comparison of a polymer-based paclitaxel-eluting stent with a bare metal stent in patients with complex coronary artery disease: a randomized controlled trial. Jama. 2005 Sep 14;294(10):1215-23.
170. Dawkins KD, Grube E, Guagliumi G, et al. Clinical efficacy of polymer-based paclitaxel-eluting stents in the treatment of complex, long coronary artery lesions from a multicenter, randomized trial: support for the use of drug-eluting stents in contemporary clinical practice. Circulation. 2005 Nov 22;112(21):3306-13.
171. Kaul U, Bhagwat A, Pinto B, et al. Paclitaxel-eluting stents versus everolimus-eluting coronary stents in a diabetic population: two-year follow-up of the TUXEDO-India trial. EuroIntervention: journal of EuroPCR in collaboration with the Working Group on Interventional Cardiology of the European Society of Cardiology. 2017 Nov 1;13(10):1194-201.
172. Pilgrim T, Räber L, Limacher A,et al. Comparison of Titanium-Nitride-Oxide–Coated Stents With Zotarolimus-Eluting Stents for Coronary Revascularization: A Randomized Controlled Trial. JACC: Cardiovascular Interventions. 2011 Jun;4(6):672-82.
173. Tonino PA, Pijls NH, Collet C, et al. Titanium-nitride-oxide–coated versus everolimus-eluting stents in acute coronary syndrome: The randomized TIDES-ACS trial. Cardiovascular Interventions. 2020 Jul 27;13(14):1697-705.
174. von Birgelen C, Basalus MW, Tandjung K, et al. A randomized controlled trial in second-generation zotarolimus-eluting Resolute stents versus everolimus-eluting Xience V stents in real-world patients: the TWENTE trial. Journal of the American College of Cardiology. 2012 Apr 10;59(15):1350-61.
175. von Birgelen C, Sen H, Lam MK, et al. Third-generation zotarolimus-eluting and everolimus-eluting stents in all-comer patients requiring a percutaneous coronary intervention (DUTCH PEERS): a randomised, single-blind, multicentre, non-inferiority trial. The Lancet. 2014 Feb 1;383(9915):413-23.
176. Spaulding C, Henry P, Teiger E, et al. Sirolimus-eluting versus uncoated stents in acute myocardial infarction. New England Journal of Medicine. 2006 Sep 14;355(11):1093-104.
177. Yin D, Li J, Yang YJ,et al. Nine-year clinical outcomes of drug-eluting stents vs. bare metal stents for large coronary vessel lesions. Journal of geriatric cardiology: JGC. 2017 Jan;14(1):35.
178. Wessely R, Kastrati A, Mehilli J, et al. Randomized trial of rapamycin-and paclitaxel-eluting stents with identical biodegradable polymeric coating and design. European heart journal. 2007 Nov 1;28(22):2720-5.
179. Dharma S, Wardeh AJ, Soerianata S,et al. A Randomized Comparison between Everolimus-Eluting Stent and Cobalt Chromium Stent in Patients with Acute ST-Elevation Myocardial Infarction Undergoing Primary Percutaneous Coronary Intervention Using Routine Intravenous Eptifibatide: The X-MAN (Xience vs. Multi-Link Stent in Acute Myocardial Infarction) Trial, A Pilot Study. International Journal of Angiology. 2014 Jun;23(02):093-100.
180. Hofma SH, Brouwer J, Velders MA, et al. Second-generation everolimus-eluting stents versus first-generation sirolimus-eluting stents in acute myocardial infarction: 1-year results of the randomized XAMI (XienceV Stent vs. Cypher Stent in Primary PCI for acute myocardial infarction) trial. Journal of the American College of Cardiology. 2012 Jul 31;60(5):381-7.
181. Park DW, Kim YH, Yun SC, et al. Comparison of zotarolimus-eluting stents with sirolimus-and paclitaxel-eluting stents for coronary revascularization: the ZEST (comparison of the efficacy and safety of zotarolimus-eluting stent with sirolimus-eluting and paclitaxel-eluting stent for coronary lesions) randomized trial. Journal of the American College of Cardiology. 2010 Oct 5;56(15):1187-95.
182. Lee CW, Park DW, Lee SH, et al. Comparison of the efficacy and safety of zotarolimus-, sirolimus-, and paclitaxel-eluting stents in patients with ST-elevation myocardial infarction. The American journal of cardiology. 2009 Nov 15;104(10):1370-6.
183. Valgimigli M, Patialiakas A, Thury A, et al. Zotarolimus-eluting versus bare-metal stents in uncertain drug-eluting stent candidates. Journal of the American College of Cardiology. 2015 Mar 3;65(8):805-15.
184. Zhang Q, Zhang RY, Zhang JS, et al. One-year clinical outcomes of Chinese sirolimus-eluting stent in the treatment of unselected patients with coronary artery disease. Chinese medical journal. 2006 Jan 1;119(2):165-8.
185. Zhang L, Yuan J, Liu G, et al. One‐year clinical outcome of a randomized trial of polymer‐free paclitaxel‐eluting stents versus biodegradable polymer‐based rapamycin‐eluting stents in patients with coronary heart disease. Journal of interventional cardiology. 2012 Dec;25(6):604-10.
186. Zhang Y, Shen J, Li Z, et al. Two-year clinical outcomes of different drug-eluting stents with different polymer coating strategies in coronary artery heart disease: a multi-centre, randomised, controlled clinical trial. International journal of cardiology. 2013 Oct 3;168(3):2646-52.
187. Chevalier B, Di Mario C, Neumann FJ, et al. A randomized, controlled, multicenter trial to evaluate the safety and efficacy of zotarolimus-versus paclitaxel-eluting stents in de novo occlusive lesions in coronary arteries: the ZoMaxx I trial. JACC: Cardiovascular Interventions. 2008 Oct;1(5):524-32.
188. Gray WA, Yeung AC, Cutlip DE, et al. A randomized, controlled, multi-center trial comparing the safety and efficacy of zotarolimus-eluting and paclitaxel-eluting stents in de novo lesions in coronary arteries: Final results of the ZoMaxx II trial. International journal of cardiology. 2012 May 17;157(1):96-101.
189. de Belder A, de la Torre Hernandez JM, et al. A prospective randomized trial of everolimus-eluting stents versus bare-metal stents in octogenarians: the XIMA Trial (Xience or Vision Stents for the Management of Angina in the Elderly). Journal of the American College of Cardiology. 2014 Apr 15;63(14):1371-5.
190. Bo XU, Ke-fei DO, Ya-ling HA, et al. A prospective multicenter parallel-controlled trial of TIVOLI biodegradable-polymer-based sirolimus-eluting stent compared to ENDEAVOR zotarolimus-eluting stent for the treatment of coronary artery disease: 8-month angiographic and 2-year clinical follow-up results. Chinese medical journal. 2011 Mar 1;124(6):811-6.
191. Windecker S, Simon R, Lins M, et al. Randomized comparison of a titanium-nitride-oxide-coated stent with a stainless steel stent for coronary revascularization: the TiNOX trial. Circulation: AHA. 2005 May 24;111(20):2617-22.
192. Karjalainen PP, Ylitalo A, Niemelä M, et al. Titanium-nitride-oxide coated stents versus paclitaxel-eluting stents in acute myocardial infarction: a 12-months follow-up report from the TITAX AMI trial. EuroIntervention. 2008 Aug;4(2):234-41.
193. Karjalainen PP, Niemelä M, Airaksinen JK, et al. A prospective randomized comparison of titanium-nitride-oxide-coated bioactive stents with everolimus-eluting stents in acute coronary syndrome: the BASE-ACS trial. EuroIntervention. 2012 Jul 20;8(3):306-15.
194. López-Mínguez JR, Nogales-Asensio JM, Doncel-Vecino LJ, et al. A randomized study to compare bioactive titanium stents and everolimus-eluting stents in diabetic patients (TITANIC XV): 1-year results. Rev Esp Cardiol (Engl Ed). 2014 Jul;67(7):522-30.
195. Raul Moreno, Eulogio Garcia, Rui Teles, et al. Randomized Comparison of Sirolimus-Eluting and Everolimus-Eluting Coronary Stents in the Treatment of Total Coronary Occlusions. Results From the Chronic Coronary Occlusion Treated by Everolimus-eluting Stent Randomized Trial. Circulation: Cardiovascular Interventions. Feb 2013;6:21–28.
196. Clemens Von Birgelen, Taku Asano, Giovanni Amoroso, et al. First-in-man randomized comparison of the BuMA Supreme biodegradable polymer sirolimus-eluting stent versus a durable polymer zotarolimus-eluting coronary stent: the PIONEER trial. EuroIntervention 2018; 13:2026-2035.
197. Ouyang H, Zeng X, Zhang C, Song L, Xu J, Hou Z, Xie S, Tao Z, He J. A meta-analysis of everolimus-eluting stents versus sirolimus-eluting stents and paclitaxel-eluting stents in diabetic patients. Journal of Cardiothoracic Surgery. 2021 Dec;16(1):1-1.
198. Deng S, Yi X, Tian Z. Cardiovascular outcomes associated with Ultrathin bioresorbable polymer sirolimus eluting stents versus thin, durable polymer everolimus eluting stents following percutaneous coronary intervention in patients with type 2 diabetes mellitus: A meta-analysis of published studies. Medicine. 2020 Dec 24;99(52).
199. Monjur MR, Said CF, Bamford P, Parkinson M, Szirt R, Ford T. Ultrathin-strut biodegradable polymer versus durable polymer drug-eluting stents: a meta-analysis. Open heart. 2020 Oct 1;7(2):e001394.
200. Ke J, Zhang H, Huang J, Lv P, Chen Y, Xu K, Yang W, Tu B. Three-year outcomes of bioresorbable vascular scaffolds versus second-generation drug-eluting stents: Meta-analysis of randomized trials. Medicine. 2020 Jul 31;99(31).
201. Ni L, Chen H, Luo Z, Yu Y. Bioresorbable vascular stents and drug-eluting stents in treatment of coronary heart disease: a meta-analysis. Journal of cardiothoracic surgery. 2020 Dec;15(1):1-7.
202. Chen YL, Fan J, Chen G, Cao L, Lu L, Xu Y, Yin Y. Polymer-free drug-eluting stents versus permanent polymer drug-eluting stents: An updated meta-analysis. Medicine. 2019 Apr;98(15).
203. Wu JJ, Way JA, Kritharides L, Brieger D. Polymer-free versus durable polymer drug-eluting stents in patients with coronary artery disease: A meta-analysis. Annals of medicine and surgery. 2019 Feb 1;38:13-21.
204. Shah R, Rao SV, Latham SB, Kandzari DE. Efficacy and safety of drug-eluting stents optimized for biocompatibility vs bare-metal stents with a single month of dual antiplatelet therapy: a meta-analysis. JAMA cardiology. 2018 Nov 1;3(11):1050-9.
205. Wu JJ, Way JA, Roy P, Yong A, Lowe H, Kritharides L, Brieger D. Biodegradable polymer versus second‐generation durable polymer drug‐eluting stents in patients with coronary artery disease: A meta‐analysis. Health science reports. 2018 Nov;1(11):e93.
206. Zhu P, Zhou X, Zhang C, Li H, Zhang Z, Song Z. Safety and efficacy of ultrathin strut biodegradable polymer sirolimus-eluting stent versus durable polymer drug-eluting stents: a meta-analysis of randomized trials. BMC Cardiovascular Disorders. 2018 Dec;18(1):1-1.
207. Kheiri B, Osman M, Abdalla A, Ahmed S, Bachuwa G, Hassan M. The short‐and long‐term outcomes of percutaneous intervention with drug‐eluting stent vs bare‐metal stent in saphenous vein graft disease: an updated meta‐analysis of all randomized clinical trials. Clinical Cardiology. 2018 May;41(5):685-92.
208. Felix CM, van den Berg VJ, Hoeks SE, Fam JM, Lenzen M, Boersma E, Smits PC, Serruys PW, Onuma Y, van Geuns RJ. Mid-term outcomes of the Absorb BVS versus second-generation DES: A systematic review and meta-analysis. PloS one. 2018 May 9;13(5):e0197119.
209. Mahmoud AN, Shah NH, Elgendy IY, Agarwal N, Elgendy AY, Mentias A, Barakat AF, Mahtta D, David Anderson R, Bavry AA. Safety and efficacy of second‐generation drug‐eluting stents compared with bare‐metal stents: An updated meta‐analysis and regression of 9 randomized clinical trials. Clinical cardiology. 2018 Jan;41(1):151-8.
210. Bundhun PK, Pursun M, Huang F. Biodegradable polymer drug-eluting stents versus first-generation durable polymer drug-eluting stents: A systematic review and meta-analysis of 12 randomized controlled trials. Medicine. 2017 Nov;96(47).
211. Lu P, Lu S, Li Y, Deng M, Wang Z, Mao X. A comparison of the main outcomes from BP-BES and DP-DES at five years of follow-up: A systematic review and meta-analysis. Scientific Reports. 2017 Nov 3;7(1):1-9.
212. Bundhun PK, Bhurtu A, Pursun M, Soogund MZ, Teeluck AR, Huang WQ. Long-term (2–5 years) adverse clinical outcomes associated with ZES versus SES, PES and EES: A Meta-Analysis. Scientific Reports. 2017 Jul 25;7(1):1-8.
213. Gao K, Sun Y, Yang M, Han L, Chen L, Hu W, Chen P, Li X. Efficacy and safety of polymer-free stent versus polymer-permanent drug-eluting stent in patients with acute coronary syndrome: a meta-analysis of randomized control trials. BMC cardiovascular disorders. 2017 Dec;17(1):1-9.
214. Bundhun PK, Janoo G, Yanamala CM, Huang F. Adverse cardiovascular events associated with biodegradable polymer drug-eluting stents and durable polymer everolimus-eluting stents: A systematic review and meta-analysis of 10 randomized controlled trials. Medicine. 2017 Jul;96(28).
215. Bundhun PK, Yanamala CM, Huang WQ. Comparing Stent Thrombosis associated with Zotarolimus Eluting Stents versus Everolimus Eluting Stents at 1 year follow up: a systematic review and meta-analysis of 6 randomized controlled trials. BMC cardiovascular disorders. 2017 Dec;17(1):1-2.
216. Lu R, Tang F, Zhang Y, Zhu X, Zhu S, Wang G, Jiang Y, Fan Z. Comparison of drug‐eluting and bare metal stents in patients with chronic kidney disease: an updated systematic review and meta‐analysis. Journal of the American Heart Association. 2016 Nov 7;5(11):e003990.
217. Ferko N, Ferrante G, Hasegawa JT, Schikorr T, Soleas IM, Hernandez JB, Sabaté M, Kaiser C, Brugaletta S, De La Torre Hernandez JM, Galatius S. Cost‐effectiveness of percutaneous coronary intervention with cobalt‐chromium everolimus eluting stents versus bare metal stents: Results from a patient level meta‐analysis of randomized trials. Catheterization and cardiovascular interventions. 2017 May;89(6):994-1002.
218. Bundhun PK, Soogund MZ, Pursun M, Chen MH. Stent thrombosis and adverse cardiovascular outcomes observed between six months and five years with sirolimus-eluting stents and other drug-eluting stents in patients with Type 2 diabetes mellitus complicated by coronary artery disease: a systematic review and meta-analysis. Medicine. 2016 Jul;95(27).
219. Bundhun PK, Bhurtu A, Soogund MZ, Long MY. Comparing the clinical outcomes between drug eluting stents and bare metal stents in patients with insulin-treated type 2 diabetes mellitus: a systematic review and meta-analysis of 10 randomized controlled trials. PLoS One. 2016 Apr 25;11(4):e0154064.
220. Yan YF, Jiang L, Zhang MD, Li XH, Nie MX, Feng TT, Zhao X, Wang LY, Zhao QM. Can Platforms Affect the Safety and Efficacy of Drug-Eluting Stents in the Era of Biodegradable Polymers?: A Meta-Analysis of 34,850 Randomized Individuals. PloS one. 2016 Mar 31;11(3):e0151259.
221. Pandya B, Gaddam S, Raza M, Asti D, Nalluri N, Vazzana T, Kandov R, Lafferty J. Biodegradable polymer stents vs second generation drug eluting stents: a meta-analysis and systematic review of randomized controlled trials. World Journal of Cardiology. 2016 Feb 26;8(2):240.
222. Wu DW, Yu MY, Gao HY, Zhang L, Song F, Zhang XY, Wu YJ. Polymer-free versus permanent polymer drug eluting stents in coronary artery disease: a meta-analysis of 10 RCTs with 6575 patients. Chronic Diseases and Translational Medicine. 2015 Dec 1;1(04):221-30.
223. Meng M, Gao B, Wang X, Bai ZG, Sa RN, Ge B. Long-term clinical outcomes of everolimus-eluting stent versus paclitaxel-eluting stent in patients undergoing percutaneous coronary interventions: a meta-analysis. BMC cardiovascular disorders. 2016 Dec;16(1):1-1.
224. Lv J, Wu Y, Zhang X, Jing T, Zhang L, Tong S, Song Z, Wang M, Wang G, Chi L. Comparison of the safety and efficacy of biodegradable polymer drug-eluting stents versus durable polymer drug-eluting stents: a meta-analysis. European Journal of Medical Research. 2015 Dec;20(1):1-2.
225. Wang QI, Zhou YU, Qiao T, Zhou M. Clinical performance of biodegradable versus permanent polymer drug‑eluting stents: A meta‑analysis of randomized clinical trials at long‑term follow‑up. Experimental and Therapeutic Medicine. 2015 Apr 1;9(4):1545-56.
226. Wu G, Sun G, Zhao R, Sun M. Clinical outcomes of second-versus first-generation drug-eluting stents in patients with acute myocardial infarction: a meta-analysis of randomized controlled trials. Archives of Medical Science: AMS. 2014 Aug 29;10(4):643.
227. Navarese EP, Kowalewski M, Kandzari D, Lansky A, Górny B, Kołtowski Ł, Waksman R, Berti S, Musumeci G, Limbruno U, van der Schaaf RJ. First-generation versus second-generation drug-eluting stents in current clinical practice: updated evidence from a comprehensive meta-analysis of randomised clinical trials comprising 31 379 patients. Open heart. 2014 Aug 1;1(1):e000064.
228. Yin Y, Zhang Y, Zhao X. Safety and efficacy of biodegradable drug-eluting vs. bare metal stents: a meta-analysis from randomized trials. PloS one. 2014 Jun 19;9(6):e99648.
229. Zhang X, Xie J, Li G, Chen Q, Xu B. Head-to-head comparison of sirolimus-eluting stents versus paclitaxel-eluting stents in patients undergoing percutaneous coronary intervention: a meta-analysis of 76 studies. PloS one. 2014 May 20;9(5):e97934.
230. Yan P, Dong P, Li Z. Second-versus first-generation drug-eluting stents for diabetic patients: a meta-analysis. Archives of Medical Science: AMS. 2014 May 12;10(2):213.
231. Kwong JS, Yu CM. Clinical Outcomes of Biodegradable Polymer Drug‐Eluting Stents for Percutaneous Coronary Intervention: An Updated Meta‐analysis of Randomized Controlled Trials. Clinical cardiology. 2014 Jul;37(7):440-53.
232. Ye Y, Xie H, Zeng Y, Zhao X, Tian Z, Zhang S. Efficacy and safety of biodegradable polymer biolimus-eluting stents versus durable polymer drug-eluting stents: a meta-analysis. PloS one. 2013 Nov 11;8(11):e78667.
233. Bangalore S, Toklu B, Amoroso N, Fusaro M, Kumar S, Hannan EL, Faxon DP, Feit F. Bare metal stents, durable polymer drug eluting stents, and biodegradable polymer drug eluting stents for coronary artery disease: mixed treatment comparison meta-analysis. Bmj. 2013 Nov 8;347.
234. Navarese EP, Tandjung K, Claessen B, Andreotti F, Kowalewski M, Kandzari DE, Kereiakes DJ, Waksman R, Mauri L, Meredith IT, Finn AV. Safety and efficacy outcomes of first and second generation durable polymer drug eluting stents and biodegradable polymer biolimus eluting stents in clinical practice: comprehensive network meta-analysis. Bmj. 2013 Nov 6;347.
235. Ullah W, Zghouzi M, Ahmad B, Suleiman AR, Zahid S, Faisaluddin M, Alabdalrazzak M, Sattar Y, Kalra A, Kapadia S, Fischman DL. Safety and efficacy of the polymer‐free and polymer‐coated drug‐eluting stents in patients undergoing percutaneous coronary intervention. Catheterization and Cardiovascular Interventions. 2021 Nov 15;98(6):E802-13.
